# Correction: Early Refill of an Opioid Medication: Recognizing Personal Biases Through Clinical Vignettes and OSCEs

**DOI:** 10.15766/mep_2374-8265.11374

**Published:** 2023-12-13

## Correction

In the publication “Early Refill of an Opioid Medication: Recognizing Personal Biases Through Clinical Vignettes and OSCEs,” the following sentence should be added to the Funding/Support field:

This publication was supported by the Cooperative Agreement Number 5 NU36OE000007-03, funded by the Centers for Disease Control and Prevention.

Additionally, the following field should be added:

### Disclaimer

The contents of this publication are solely the responsibility of the authors and do not necessarily represent the official views of the Centers for Disease Control and Prevention or the Department of Health and Human Services.

## References

[R1] Lu WH, Baldelli P, Migdal P, Iuli R, Strano-Paul L, Zacharoff KL. Early refill of an opioid medication: recognizing personal biases through clinical vignettes and OSCEs. MedEdPORTAL. 2022;18:11234. 10.15766/mep_2374-8265.11234 35497675 PMC8986891

