# Supplementary material for: Early Refill of an Opioid Medication: Recognizing Personal Biases Through Clinical Vignettes and OSCEs
Source: MedEdPORTAL. 2022 Apr 7;18:11234. doi: 10.15766/mep_2374-8265.11234 (PMC8986891; doi:10.15766/mep_2374-8265.11234)
Supplement: Supplementary file 1 — MS 1 Clinical Vignettes & Follow-Up.pptxMS 1 Debrief.pptxSP James Spiegel - Case 1.docxSP Darryl Whitcomb - Case 2.docxSP Helen Morgan - Case 3.docxDoor Notes.docxLogistical Flow.docxFaculty Post-OSCE Debrief Discussion Guide.docxSP Encounter Checklist.docxSP Responses for Checklist Items.docxMS 3 Post-OSCE Survey.docx [file mep_2374-8265.11234-s001.zip › F. Door Notes.docx]

**Name of Case: Opioid Use and Implicit Bias Case 1**

**Door Note for Learner**

Patient Name: James Spiegel

Chief Complaint: Back Pain

James Spiegel is a 39-year-old male patient who presents today for a virtual telehealth visit requesting an early refill of his monthly prescribed opioid analgesic**.**

Vital Signs

Temperature (98.6)

Blood Pressure (130/85)

Pulse (85)

Respiration (14)

### EXAMINEE TASKS

- Obtain a history pertinent to the patient’s problem.
- Complete a focused telehealth physical exam that includes the localization of the pain, range of motion of the back and motor strength of the lower extremities.
- Discuss your initial impressions and plans with respect to the patient’s request for an early refill of their opioid analgesic medication.

You will have 20 minutes to perform these tasks.

You will receive a 5-minute warning 15 minutes after the encounter has begun and a warning at the end of the encounter.

**Name of Case: Opioid Use and Implicit Bias Case 1**

**Door Note Copy for SP**

Patient Name: James Spiegel

Chief Complaint: Back Pain

James Spiegel is a 39-year-old male patient who presents today for a virtual telehealth visit requesting an early refill of his monthly prescribed opioid analgesic**.**

Vital Signs

Temperature (98.6)

Blood Pressure (130/85)

Pulse (85)

Respiration (14)

### EXAMINEE TASKS

- Obtain a history pertinent to the patient’s problem.
- Complete a focused telehealth physical exam that includes the localization of the pain, range of motion of the back and motor strength of the lower extremities.
- Discuss your initial impressions and plans with respect to the patient’s request for an early refill of their opioid analgesic medication.

You will have 20 minutes to perform these tasks.

You will receive a 5-minute warning 15 minutes after the encounter has begun and a warning at the end of the encounter.

*How to direct the conversation:*

You are James Spiegel, who is a 39-year-old patient who has been treated with opioid analgesics for chronic recurring back pain for the past 5 years in this practice. Today you are seeing a physician who is new to this practice. You are here to request an early refill of your prescribed opioid analgesic. You are feeling rushed and anxious as you want to make this quick as you have an important meeting you need to get to. You convey to the Doctor that your pain management is very stable and that you are able to control your back pain with this medication. However, if you cannot get a refill you fear that it will become very difficult for you to manage your pain.

You are assertive and manipulative just shy to the point of being pushy and overanxious. You *want to be believed*, but also *want to leave with an early refill* of your prescription pain medication.

**Name of Case: Opioid Use and Implicit Bias Case 2**

**Door Note for Learner**

Patient Name: Darryl Whitcomb

Chief Complaint: Back Pain

Darryl Whitcomb is a 56-year-old male patient who presents today for a virtual telehealth visit requesting an early refill of his monthly prescribed opioid analgesic.

Vital Signs

Temperature (98.4)

Blood Pressure (140/90)

Pulse (90)

Respiration (12)

**EXAMINEE TASKS**

- Obtain a history pertinent to the patient’s problem.
- Complete a focused telehealth physical exam that includes the localization of the pain, range of motion of the back and motor strength of the lower extremities.
- Discuss your initial impressions and plans with respect to the patient’s request for an early refill of their opioid analgesic medication.

You will have 20 minutes to perform these tasks.

You will receive a 5-minute warning 15 minutes after the encounter has begun and a warning at the end of the encounter.

**Name of Case: Opioid Use and Implicit Bias Case 2**

**Door Note Copy for SP**

Patient Name: Darryl Whitcomb

Chief Complaint: Back Pain

Darryl Whitcomb is a 56-year-old male patient who presents today for a virtual telehealth visit requesting an early refill of his monthly prescribed opioid analgesic.

Vital Signs

Temperature (98.4)

Blood Pressure (140/90)

Pulse (90)

Respiration (12)

**EXAMINEE TASKS**

- Obtain a history pertinent to the patient’s problem.
- Complete a focused telehealth physical exam that includes the localization of the pain, range of motion of the back and motor strength of the lower extremities.
- Discuss your initial impressions and plans with respect to the patient’s request for an early refill of their opioid analgesic medication.

You will have 20 minutes to perform these tasks.

You will receive a 5-minute warning 15 minutes after the encounter has begun and a warning at the end of the encounter.

*How to direct the conversation:*

You are Darryl Whitcomb, who is a 56-year-old patient who has been treated with opioid analgesics for chronic recurring back pain for the past 7 years in this practice. Today you are seeing a physician who is new to this practice. You are presenting to request an early refill of your prescribed opioid analgesic. You convey to the Doctor that your pain control is normally tolerable on your medication, but you have been miserable for the past 4 days. You are lackadaisical, laid-back behavior just shy of not taking things seriously including responsible use/storage of your opioid medication. You portray *a somewhat indignant* attitude, but you also *wans to leave with an early refill* of your prescription pain medication.

**Name of Case: Opioid Use and Implicit Bias Case 3**

**Door Note for Learner**

Patient Name: Helen Morgan

Chief Complaint: Back Pain

Helen Morgan is a 77-year-old female patient who presents today for a virtual telehealth visit requesting an early refill of her monthly prescribed opioid analgesic.

Vital Signs

Temperature (97.9)

Blood Pressure (148/90)

Pulse (82)

Respiration (12)

### EXAMINEE TASKS

- Obtain a history pertinent to the patient’s problem.
- Complete a focused telehealth physical exam that includes the localization of the pain, range of motion of the back and motor strength of the lower extremities.
- Discuss your initial impressions and plans with respect to the patient’s request for an early refill of their opioid analgesic medication.

You will have 20 minutes to perform these tasks.

You will receive a 5-minute warning 15 minutes after the encounter has begun and a warning at the end of the encounter.

**Name of Case: Opioid Use and Implicit Bias Case 3**

**Door Note Copy for SP**

Patient Name: Helen Morgan

Chief Complaint: Back Pain

Helen Morgan is a 77-year-old female patient who presents today for a virtual telehealth visit requesting an early refill of her monthly prescribed opioid analgesic.

Vital Signs

Temperature (97.9)

Blood Pressure (148/90)

Pulse (82)

Respiration (12)

### EXAMINEE TASKS

- Obtain a history pertinent to the patient’s problem.
- Complete a focused telehealth physical exam that includes the localization of the pain, range of motion of the back and motor strength of the lower extremities.
- Discuss your initial impressions and plans with respect to the patient’s request for an early refill of their opioid analgesic medication.

You will have 20 minutes to perform these tasks.

You will receive a 5-minute warning 15 minutes after the encounter has begun and a warning at the end of the encounter.

*How to direct the conversation:*

You are Helen Morgan, who is a 77-year-old patient who has been treated with opioid analgesics for chronic recurring back pain for the past 15 years in this practice. Today you are seeing a physician who is new to this practice. You are presenting to request an early refill of their prescribed opioid analgesic.

You convey to the Doctor that you just want to make sure that you have your pain medication on hand, and that you are fearful that without it, you might not be able to take care of yourself and retain your independence if your pain should flare and worsen and become disabling to you. You are polite, mild-mannered, soft-spoken, proper, calm, and endearing. You portray a respectful and attentive attitude, but you also want to leave with an early *refill* of your prescription pain medication.
