# Supplementary material for: Early Refill of an Opioid Medication: Recognizing Personal Biases Through Clinical Vignettes and OSCEs
Source: MedEdPORTAL. 2022 Apr 7;18:11234. doi: 10.15766/mep_2374-8265.11234 (PMC8986891; doi:10.15766/mep_2374-8265.11234)
Supplement: Supplementary file 1 — MS 1 Clinical Vignettes & Follow-Up.pptxMS 1 Debrief.pptxSP James Spiegel - Case 1.docxSP Darryl Whitcomb - Case 2.docxSP Helen Morgan - Case 3.docxDoor Notes.docxLogistical Flow.docxFaculty Post-OSCE Debrief Discussion Guide.docxSP Encounter Checklist.docxSP Responses for Checklist Items.docxMS 3 Post-OSCE Survey.docx [file mep_2374-8265.11234-s001.zip › G. Logistical Flow.docx]

We implemented the iPAC curriculum **for Phase II students during the Transition to Clinical Care Medical (TCC)** course. Using the same three patient cases from the TMS course, we converted the case vignettes presented at TMS into (Tele)OSCE cases for the TCC course. This was a full day event starting from 8:45AM and continued until 4:30PM. Groups of 18 students would rotate through our virtual TeleOSCE every 30 minutes until all 7 groups consisting of 124 students completed the (Tele)OSCE case. This logistical flow is applicable for both in person and virtual sessions.

**Transition to Clinical Care (One-Day)**

James Spiegel

(n=6), 20 mins

Darryl Whitcomb

(n=6), 20 mins

Helen Morgan

(n=6), 20 mins

**Group of 18 students**

Randomly assigned to one of the 3 pt cases

Debrief Rm 1

(n=9), 30 mins

Debrief Rm 2

(n=9), 30 mins

Next Group of 18 students start

- Students complete a post- OSCE survey before debrief
- Gave a brief 10-minute orientation before the (Tele)OSCE
