# Supplementary material for: Early Refill of an Opioid Medication: Recognizing Personal Biases Through Clinical Vignettes and OSCEs
Source: MedEdPORTAL. 2022 Apr 7;18:11234. doi: 10.15766/mep_2374-8265.11234 (PMC8986891; doi:10.15766/mep_2374-8265.11234)
Supplement: Supplementary file 1 — MS 1 Clinical Vignettes & Follow-Up.pptxMS 1 Debrief.pptxSP James Spiegel - Case 1.docxSP Darryl Whitcomb - Case 2.docxSP Helen Morgan - Case 3.docxDoor Notes.docxLogistical Flow.docxFaculty Post-OSCE Debrief Discussion Guide.docxSP Encounter Checklist.docxSP Responses for Checklist Items.docxMS 3 Post-OSCE Survey.docx [file mep_2374-8265.11234-s001.zip › H. Faculty Post-OSCE Debrief Discussion Guide.docx]

**Faculty Post-OSCE Debrief Discussion Guide**

**Course: Transition to Clinical Care (TCC, early stage clinical clerkship trainees)**

**Topic of the OSCE Case: Request for Early Refill**

**Patient Names: James Spiegel, Darryl Whitcomb, and Helen Morgan**

**The purpose of this OSCE is to evaluate the students’ ability to:**

- Perform a (virtual) history and physical to make/confirm a diagnosis.
- Demonstrate knowledge and competency for assessing pain and substance use disorder (SUD).
- Assess appropriately a request for early refill of a prescription opioid analgesic.
- Recognize the potential impact of biases on clinical decision-making and the assessment and treatment of pain in the potential context of aberrant drug-related behaviors (e.g., misuse, abuse, diversion, etc.), and/or substance use disorders.

**Critical Actions**

- **Case presentation**
  - Performed by one member from each group of three students assigned to each of the three cases
  - 5 minutes
- **Feedback on case presentation**
  - **Opening Statements**
    - Name
    - Age
    - Gender
    - Chief complaint (cc)
    - Relevant historical information
    - Vital signs
- **HPI**
  - **Contains the cardinal features of the presenting symptom(s).** In this case, pain:
    - Pain rating (0-10, 0 being no pain, 10 being the worst it could be)
    - What makes it worse?
    - What makes it better?
    - Duration
    - Location
    - Quality (numbness, sharp, burning, etc.)
  - Events leading up to visit follow a temporal sequence.
  - **Integration of PMH, FH, SH, and/or ROS relevant to HPI**
- **Past Medical History**
  - Relevant medical history.
  - Relevant surgical history.
- **Medications**
  - All prescription medications with dose and frequency.
  - Important OTC and/or herbal meds
- **Allergies**
  - All drug allergies and reactions
- **Family History**
  - Relevant conditions in first-degree relatives (parents, sibs, and children)
- **Social History**
  - Quantify and detail tobacco, alcohol, and recreational drug use
  - Relevant social history which could include marital/relationship status, living arrangements, finances, housing, work, and/or major stressors in the last year
- **ROS**
  - ROS reflects on all relevant systems
  - Does not repeat information that has previously been presented
- **Physical Exam**
  - General appearance and vital signs are presented first
  - Physical exam is presented in a systematic, head-to-toe manner
  - Appropriate level of detail for each component (positive and negative findings)
  - All pertinent organ systems described, and irrelevant systems excluded
- **Assessment/Plan**
  - Synthesize all the relevant elements of the patient’s history and physical exam into a formulation statement/assessment
  - ****Bonus: Describe an appropriate differential diagnosis:**
    - **Spiegel**
      - R/O Discogenic back pain
      - R/O Musculoskeletal pain
      - Insomnia
        - Role of insomnia in chronic pain
      - R/O Medication-seeking behavior
      - R/O Aberrant Drug-related behavior
    - **Whitcomb**
      - R/O Discogenic back pain
      - R/O Musculoskeletal pain
      - Hypertension
        - Impact of uncontrolled pain on comorbid hypertension
      - Elevated Liver Enzymes
        - Etiology unknown
      - R/O Medication-seeking behavior
      - R/O Aberrant Drug-related behavior
    - **Morgan**
      - R/O Spinal Stenosis
      - R/O Vertebral Compression
      - R/O Osteoarthritis/Degenerative Disc – related back pain
      - Type II Diabetes
        - Impact of comorbid diabetes on pain-related symptoms
      - Hypertension
      - Osteoarthritis
      - Chronic Atrial Fibrillation
      - R/O Medication-seeking behavior
      - R/O Aberrant Drug-related behavior
  - ****Bonus:** **Provide an accurate argument for and against items on the differential using supportive data from the history and physical**
- **Presentation Style**
  - Clear and concise delivery style without filler words
  - Information follows a logical sequence
  - Eye contact is established during the presentation even though notes may be used
- **Discuss SP Encounter Checklist-Communication Skills Items (Appendix I)**
- **Poll for decisions about providing early refill for each of the three patients with the appropriate students (see Appendix A for the student poll questions)**
- **Discuss concerns about prescribing opioids in the context of today’s opioid epidemic**
  - **The media**
  - **Public opinion**
  - **The role of tools to assist the decision-making process**
    - Urine Drug Screens
    - Prescription Drug Monitoring Programs (e.g., I-STOP)
    - Pill Counts
- **Discuss how we typically make decisions**
  - What informs the process?
    - Experiential thinking
    - Empirical thinking
    - Evidence basis
    - Judgment
    - Fear of regulatory scrutiny
    - Personal feelings
    - Biases
- **Discuss how heuristics (mental shortcuts) may lead to cognitive biases that influence decision-making**
  - **Factors that may increase the reliance on cognitive biases**

Include: cognitive overload, fatigue, time constraints

- - **Some examples of cognitive bias:**
    - **Anchoring bias**
      - Focusing on one sign or symptom (usually the first). Hanging onto one diagnosis without taking into consideration other possibilities, or discounting and/or ignoring them.
    - **Attribution bias**
      - Explaining a patient’s condition on the basis of their disposition and/or character/characteristic rather than seeking a valid medical explanation
    - **Confirmation bias**
      - Confirmation bias is the tendency to search for, interpret, favor, and recall information in a way that confirms or supports one's prior beliefs or values
    - **Expectancy bias**
      - Making clinical decisions based on expectations or prior experiences instead of individual context and or circumstances
