# Supplementary material for: Early Refill of an Opioid Medication: Recognizing Personal Biases Through Clinical Vignettes and OSCEs
Source: MedEdPORTAL. 2022 Apr 7;18:11234. doi: 10.15766/mep_2374-8265.11234 (PMC8986891; doi:10.15766/mep_2374-8265.11234)
Supplement: Supplementary file 1 — MS 1 Clinical Vignettes & Follow-Up.pptxMS 1 Debrief.pptxSP James Spiegel - Case 1.docxSP Darryl Whitcomb - Case 2.docxSP Helen Morgan - Case 3.docxDoor Notes.docxLogistical Flow.docxFaculty Post-OSCE Debrief Discussion Guide.docxSP Encounter Checklist.docxSP Responses for Checklist Items.docxMS 3 Post-OSCE Survey.docx [file mep_2374-8265.11234-s001.zip › J. SP Responses for Checklist Items.docx]

**Standardized Patient Responses to History Checklist Items**

**Case: James Spiegel**

| **History Checklist Items** |
| --- |
| 1. Do you use any recreational/illicit/illegal drugs?   **SP response: I use marijuana and a little cocaine.** |
| 1. Do you use any drugs not prescribed to you?   **SP response: Sometimes I use some of my fiancé’s pain medication if I am having a bad day.** |
| 1. Have you ever needed an early refill before? Or Do you find you often run out of your medication early?   **SP response: Yes.** |
| 1. How many times have you needed an early refill?   **SP response: I don’t know, maybe 10 times over the past 5 years.** |
| 1. Have you ever taken you prescription pain medication in a way other than prescribed or taken extra doses?   **SP response: Yes, when I need to.** |
| 1. How long has it been since someone has performed testing to re-evaluate your back pain? Or When was the last time you had a diagnostic test (xray, MRI, etc) to evaluate your back pain?   **SP response: It’s been at least 5 years.** |
| 1. Does anyone in your family have a history of substance abuse (problem with alcohol or drugs)?   **SP response: My father was an alcoholic.** |

**Standardized Patient Responses to History Checklist Items**

**Case: Darryl Whitcomb**

| **History Checklist Items** |
| --- |
| 1. Do you use any recreational/illicit/illegal drugs?   **SP response: No. I like my beer and cigarettes but don’t touch anything else.** |
| 1. Do you use any drugs not prescribed to you?   **SP response: Nothing other than some Advil or Tylenol.** |
| 1. Have you ever needed an early refill before? Or Do you find you often run out of your medication early?   **SP response: Yes.** |
| 1. How many times have you needed an early refill?   **SP response: I don’t know, maybe 2 times over the past 7 years.** |
| 1. Have you ever taken you prescription pain medication in a way other than prescribed or taken extra doses?   **SP response: Nope.** |
| 1. How long has it been since someone has performed testing to re-evaluate your back pain? Or When was the last time you had a diagnostic test (xray, MRI, etc) to evaluate your back pain?   **SP response: It’s been at least 5 years.** |
| 1. Does anyone in your family have a history of substance abuse (problem with alcohol or drugs)?   **SP response: My father was an alcoholic.** |

**Standardized Patient Responses to History Checklist Items**

**Case: Helen Morgan**

| **History Checklist Items** |
| --- |
| 1. Do you use any recreational/illicit/illegal drugs?   **SP response: Oh my, no. I have a rare drink on special occasions.** |
| 1. Do you use any drugs not prescribed to you?   **SP response: No Doctor, I do not think that would be safe, especially since I’m taking so many different medicines.** |
| 1. Have you ever needed an early refill before? Or Do you find you often run out of your medication early?   **SP response: Yes.** |
| 1. How many times have you needed an early refill?   **SP response: Every so often, when the pills get “lost” maybe once a year.** |
| 1. Have you ever taken you prescription pain medication in a way other than prescribed or taken extra doses?   **SP response: Oh no, never.** |
| 1. How long has it been since someone has performed testing to re-evaluate your back pain? Or When was the last time you had a diagnostic test (xray, MRI, etc) to evaluate your back pain?   **SP response: It’s been at least 10 years.** |
| 1. Does anyone in your family have a history of substance abuse (problem with alcohol or drugs)?   **SP response: My father did like his scotch and sodas.** |
