# Supplementary material for: Early Refill of an Opioid Medication: Recognizing Personal Biases Through Clinical Vignettes and OSCEs
Source: MedEdPORTAL. 2022 Apr 7;18:11234. doi: 10.15766/mep_2374-8265.11234 (PMC8986891; doi:10.15766/mep_2374-8265.11234)
Supplement: Supplementary file 1 — MS 1 Clinical Vignettes & Follow-Up.pptxMS 1 Debrief.pptxSP James Spiegel - Case 1.docxSP Darryl Whitcomb - Case 2.docxSP Helen Morgan - Case 3.docxDoor Notes.docxLogistical Flow.docxFaculty Post-OSCE Debrief Discussion Guide.docxSP Encounter Checklist.docxSP Responses for Checklist Items.docxMS 3 Post-OSCE Survey.docx [file mep_2374-8265.11234-s001.zip › A. MS 1 Clinical Vignettes & Follow-Up.pptx]

## Slide 1
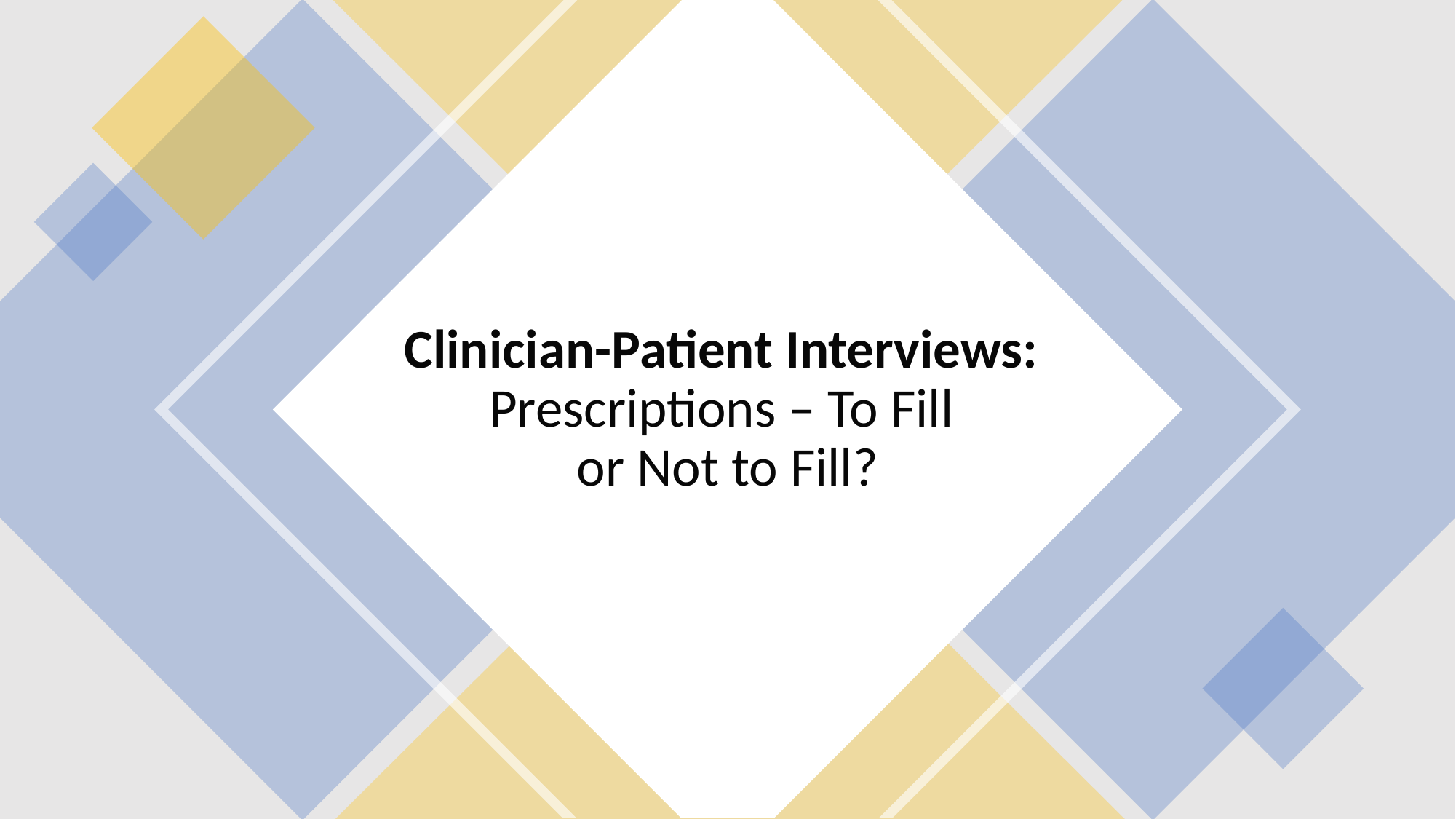

# Clinician-Patient Interviews: Prescriptions – To Fill or Not to Fill?

## Slide 2
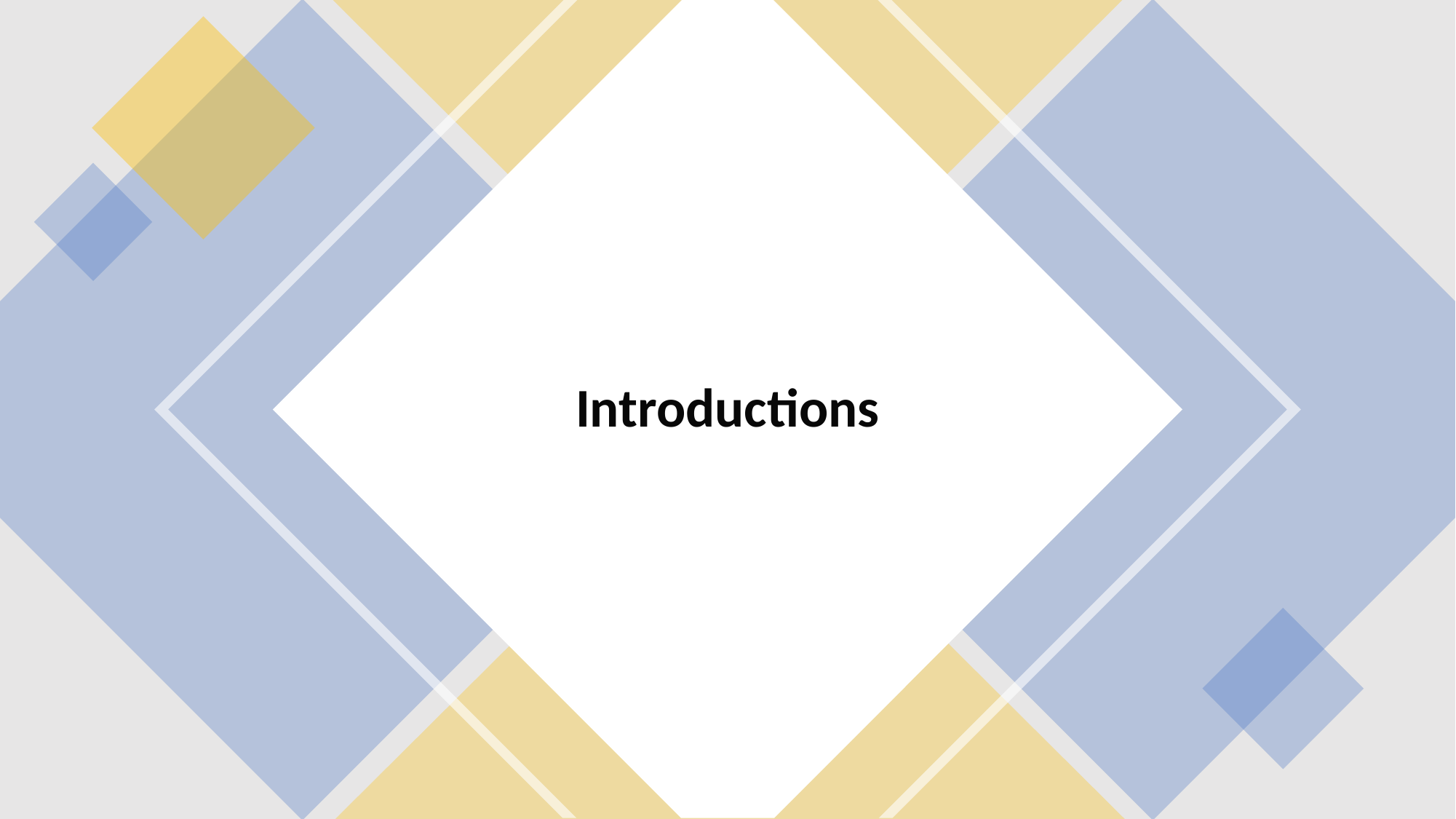

# Introductions

## Slide 3
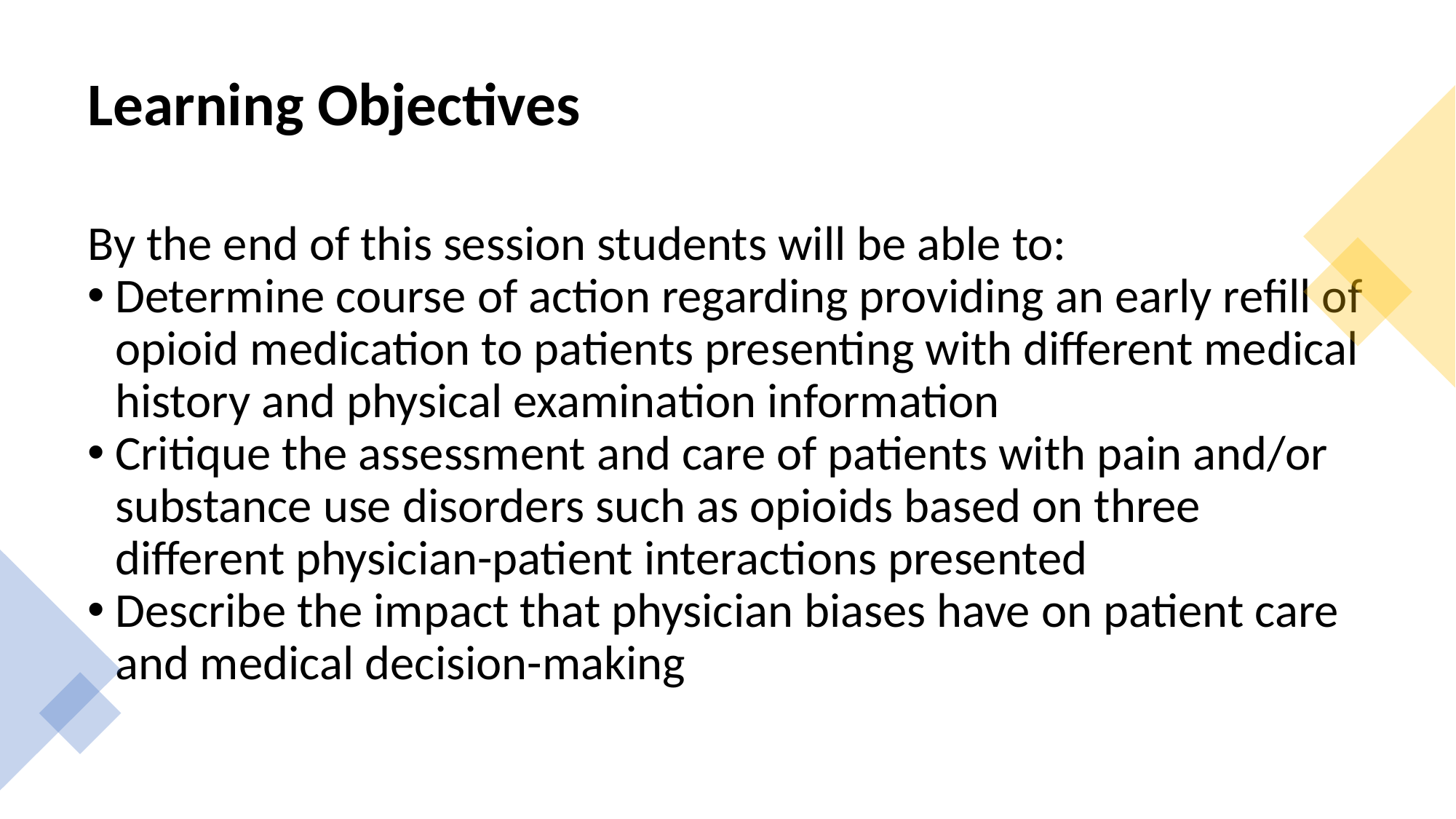

# Learning Objectives
By the end of this session students will be able to:
Determine course of action regarding providing an early refill of opioid medication to patients presenting with different medical history and physical examination information
Critique the assessment and care of patients with pain and/or substance use disorders such as opioids based on three different physician-patient interactions presented
Describe the impact that physician biases have on patient care and medical decision-making

## Slide 4
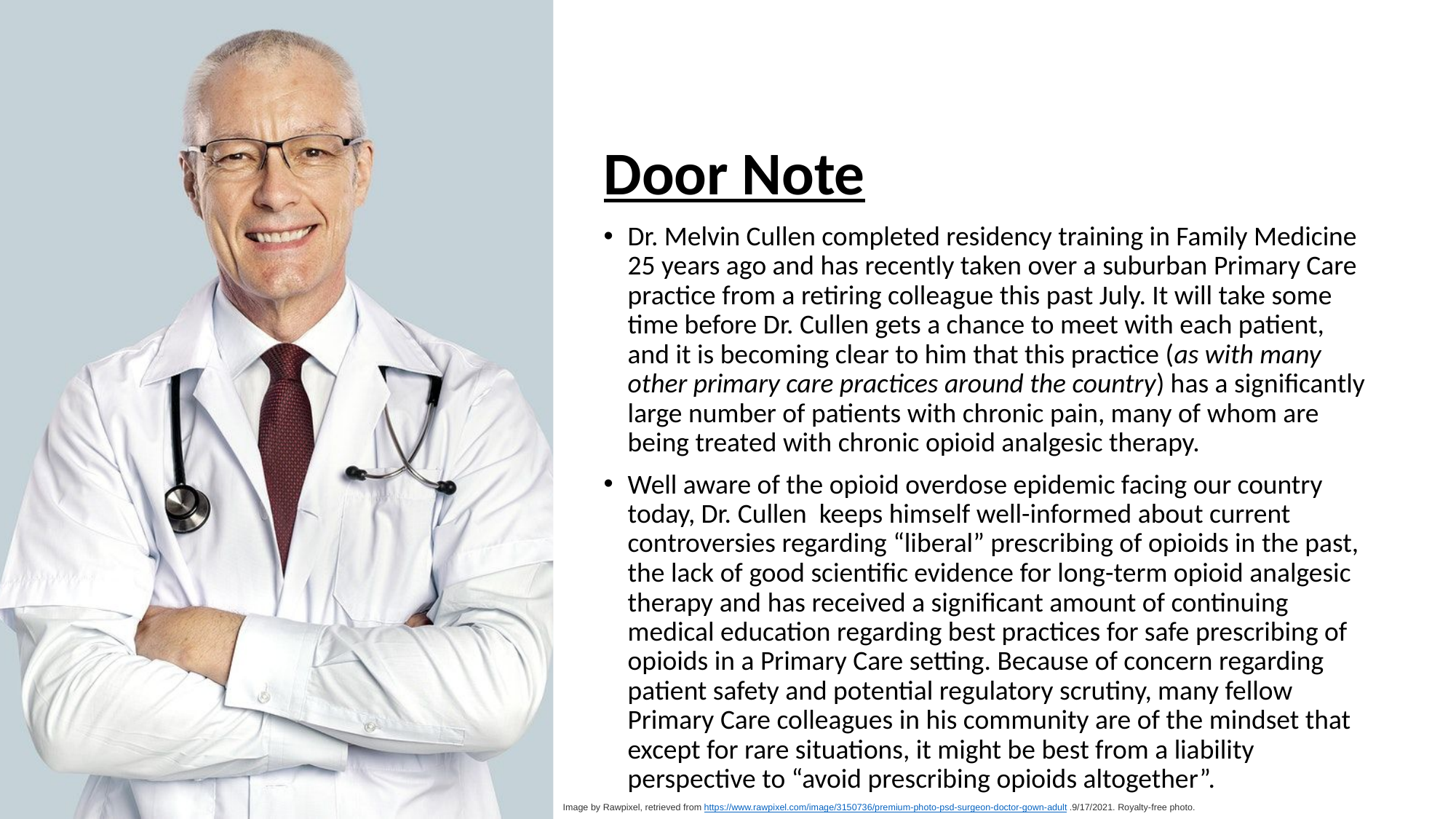

# Door Note
Dr. Melvin Cullen completed residency training in Family Medicine 25 years ago and has recently taken over a suburban Primary Care practice from a retiring colleague this past July. It will take some time before Dr. Cullen gets a chance to meet with each patient, and it is becoming clear to him that this practice (as with many other primary care practices around the country) has a significantly large number of patients with chronic pain, many of whom are being treated with chronic opioid analgesic therapy.
Well aware of the opioid overdose epidemic facing our country today, Dr. Cullen keeps himself well-informed about current controversies regarding “liberal” prescribing of opioids in the past, the lack of good scientific evidence for long-term opioid analgesic therapy and has received a significant amount of continuing medical education regarding best practices for safe prescribing of opioids in a Primary Care setting. Because of concern regarding patient safety and potential regulatory scrutiny, many fellow Primary Care colleagues in his community are of the mindset that except for rare situations, it might be best from a liability perspective to “avoid prescribing opioids altogether”.
Image by Rawpixel, retrieved from https://www.rawpixel.com/image/3150736/premium-photo-psd-surgeon-doctor-gown-adult .9/17/2021. Royalty-free photo.

## Slide 5
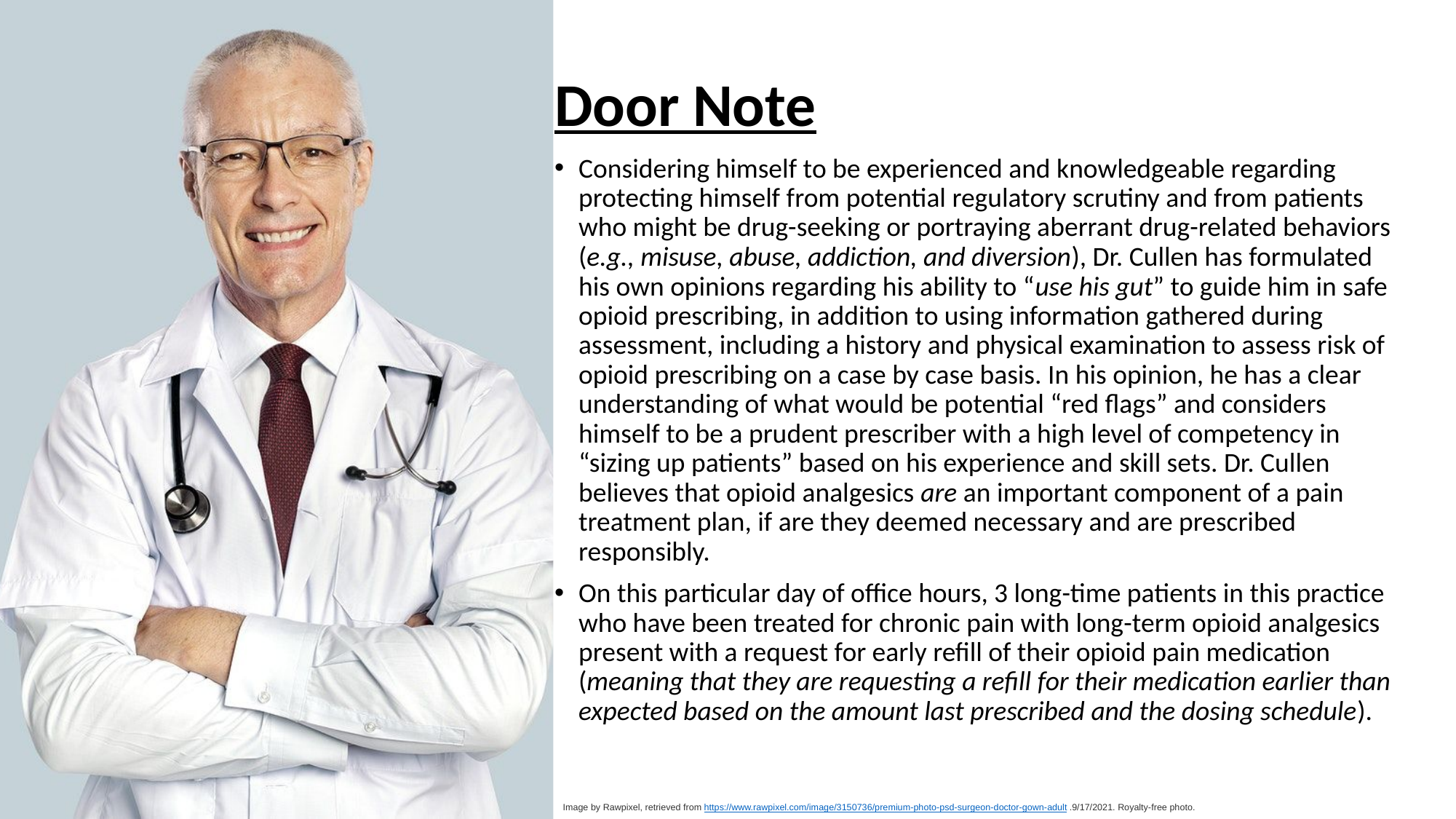

Door Note
Considering himself to be experienced and knowledgeable regarding protecting himself from potential regulatory scrutiny and from patients who might be drug-seeking or portraying aberrant drug-related behaviors (e.g., misuse, abuse, addiction, and diversion), Dr. Cullen has formulated his own opinions regarding his ability to “use his gut” to guide him in safe opioid prescribing, in addition to using information gathered during assessment, including a history and physical examination to assess risk of opioid prescribing on a case by case basis. In his opinion, he has a clear understanding of what would be potential “red flags” and considers himself to be a prudent prescriber with a high level of competency in “sizing up patients” based on his experience and skill sets. Dr. Cullen believes that opioid analgesics are an important component of a pain treatment plan, if are they deemed necessary and are prescribed responsibly.
On this particular day of office hours, 3 long-time patients in this practice who have been treated for chronic pain with long-term opioid analgesics present with a request for early refill of their opioid pain medication (meaning that they are requesting a refill for their medication earlier than expected based on the amount last prescribed and the dosing schedule).
Image by Rawpixel, retrieved from https://www.rawpixel.com/image/3150736/premium-photo-psd-surgeon-doctor-gown-adult .9/17/2021. Royalty-free photo.

## Slide 6
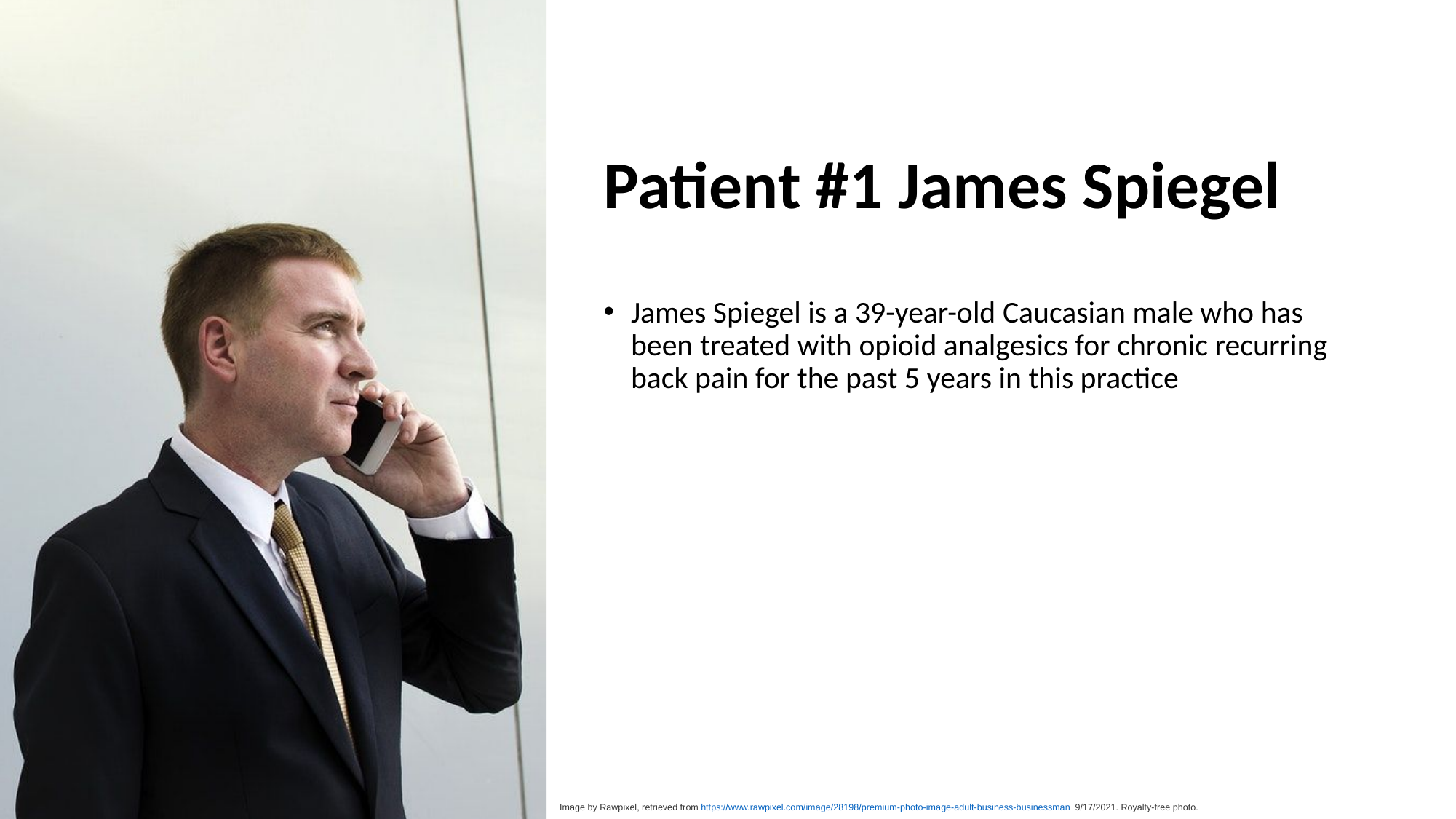

# Patient #1 James Spiegel
James Spiegel is a 39-year-old Caucasian male who has been treated with opioid analgesics for chronic recurring back pain for the past 5 years in this practice
Image by Rawpixel, retrieved from https://www.rawpixel.com/image/28198/premium-photo-image-adult-business-businessman 9/17/2021. Royalty-free photo.

## Slide 7
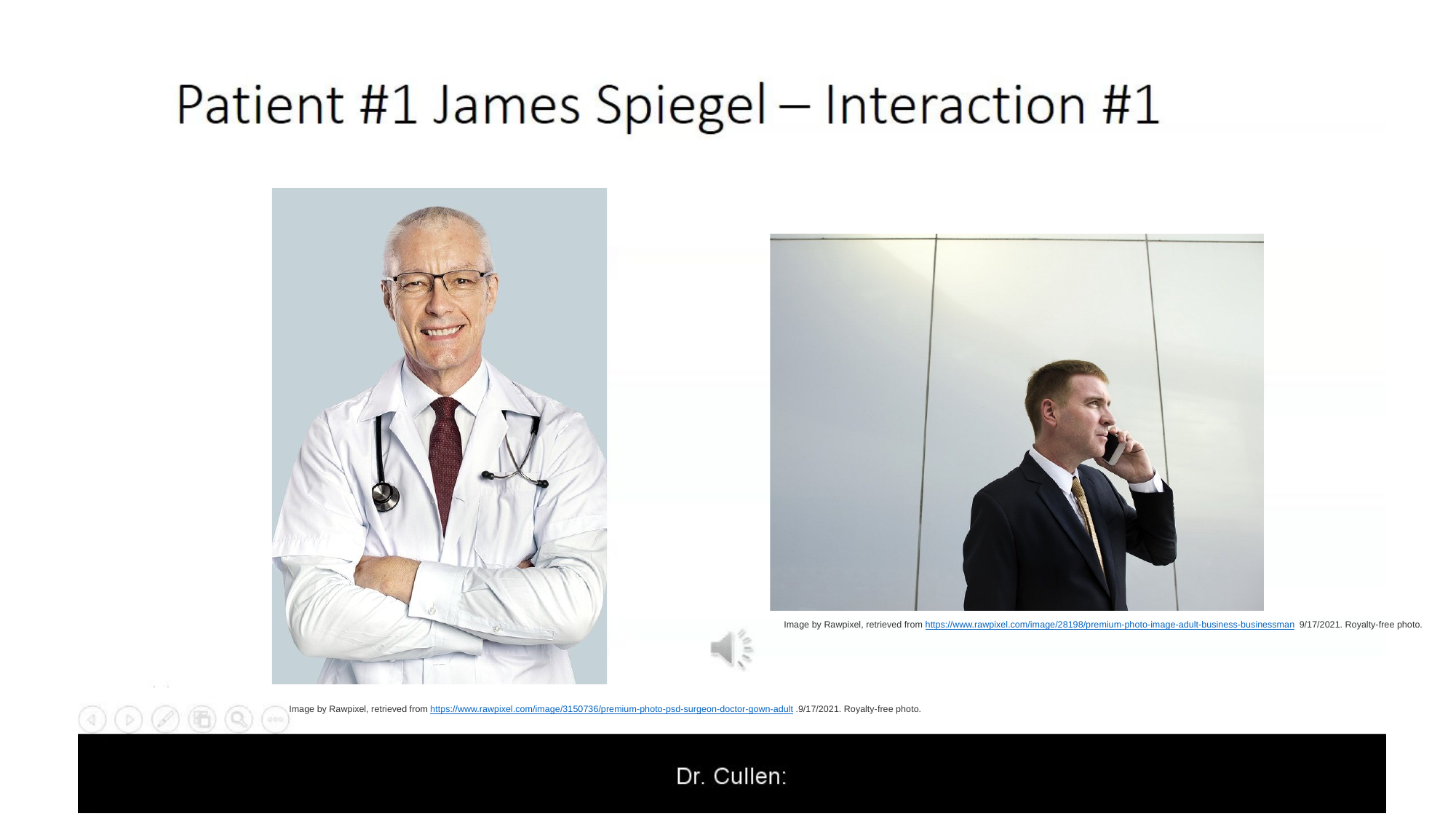

Image by Rawpixel, retrieved from https://www.rawpixel.com/image/28198/premium-photo-image-adult-business-businessman 9/17/2021. Royalty-free photo.
Image by Rawpixel, retrieved from https://www.rawpixel.com/image/3150736/premium-photo-psd-surgeon-doctor-gown-adult .9/17/2021. Royalty-free photo.

## Slide 8
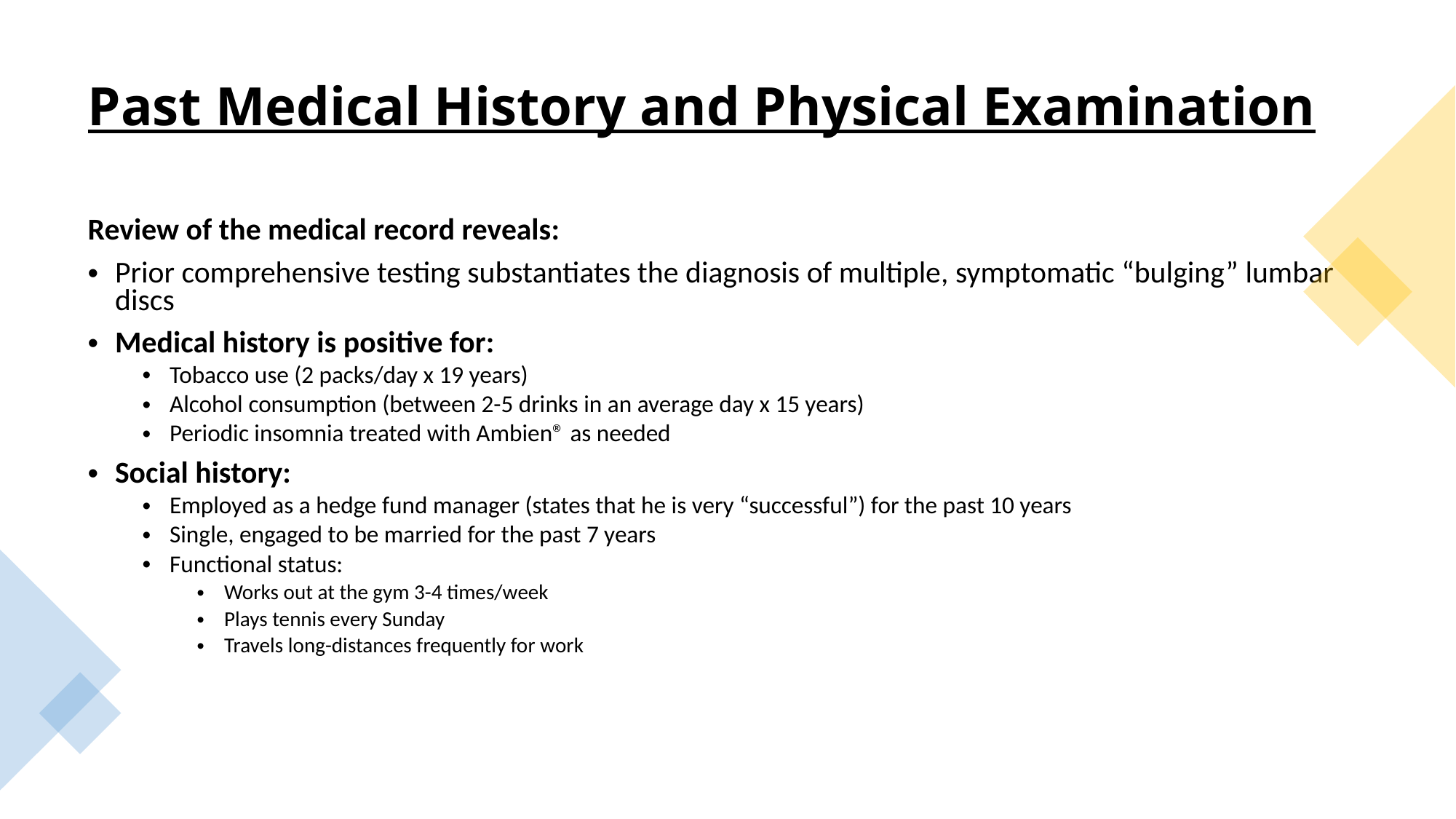

# Past Medical History and Physical Examination
Review of the medical record reveals:
Prior comprehensive testing substantiates the diagnosis of multiple, symptomatic “bulging” lumbar discs
Medical history is positive for:
Tobacco use (2 packs/day x 19 years)
Alcohol consumption (between 2-5 drinks in an average day x 15 years)
Periodic insomnia treated with Ambien® as needed
Social history:
Employed as a hedge fund manager (states that he is very “successful”) for the past 10 years
Single, engaged to be married for the past 7 years
Functional status:
Works out at the gym 3-4 times/week
Plays tennis every Sunday
Travels long-distances frequently for work

## Slide 9
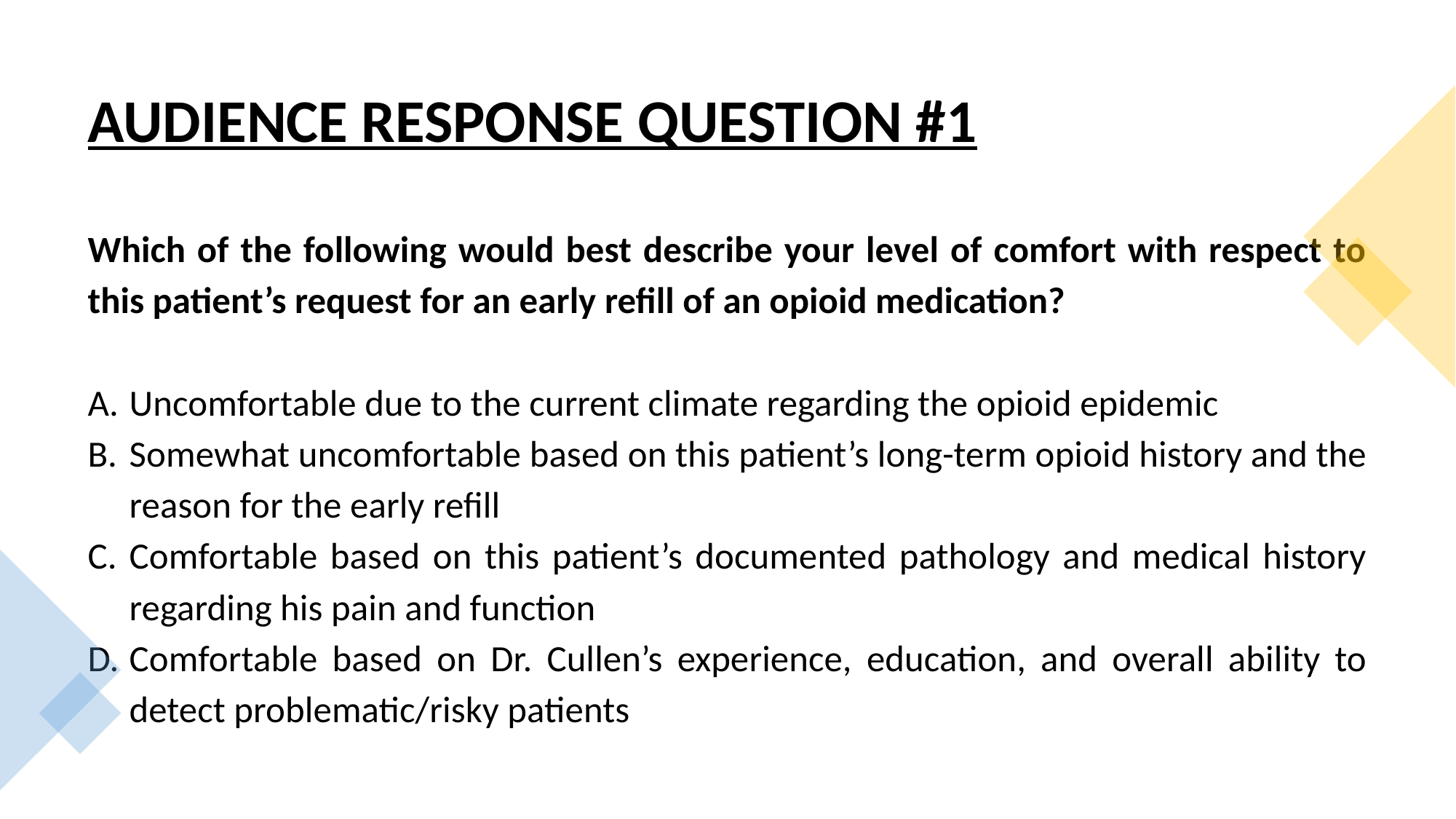

# AUDIENCE RESPONSE QUESTION #1
Which of the following would best describe your level of comfort with respect to this patient’s request for an early refill of an opioid medication?
Uncomfortable due to the current climate regarding the opioid epidemic
Somewhat uncomfortable based on this patient’s long-term opioid history and the reason for the early refill
Comfortable based on this patient’s documented pathology and medical history regarding his pain and function
Comfortable based on Dr. Cullen’s experience, education, and overall ability to detect problematic/risky patients

## Slide 10
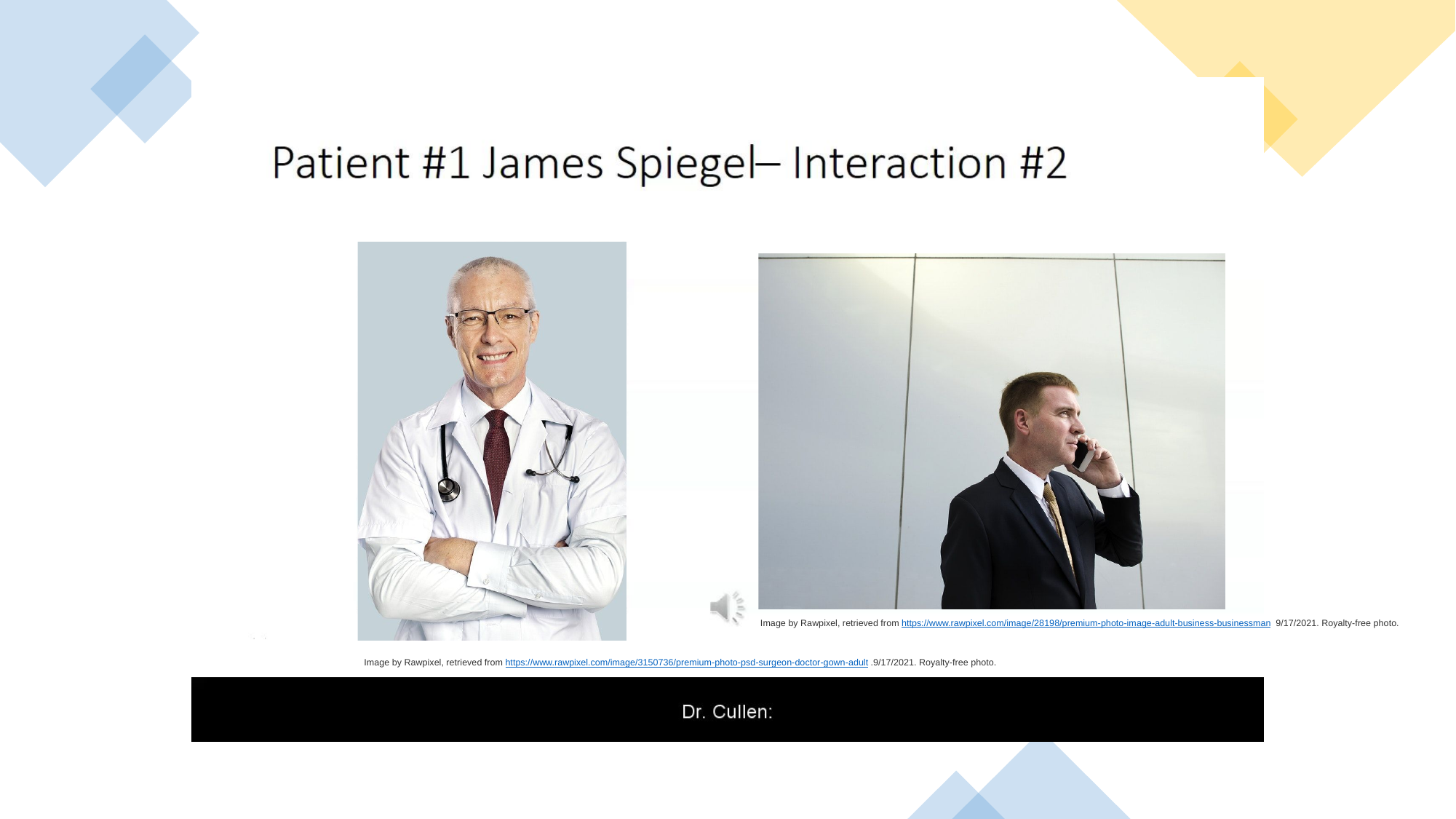

Image by Rawpixel, retrieved from https://www.rawpixel.com/image/28198/premium-photo-image-adult-business-businessman 9/17/2021. Royalty-free photo.
Image by Rawpixel, retrieved from https://www.rawpixel.com/image/3150736/premium-photo-psd-surgeon-doctor-gown-adult .9/17/2021. Royalty-free photo.

## Slide 11
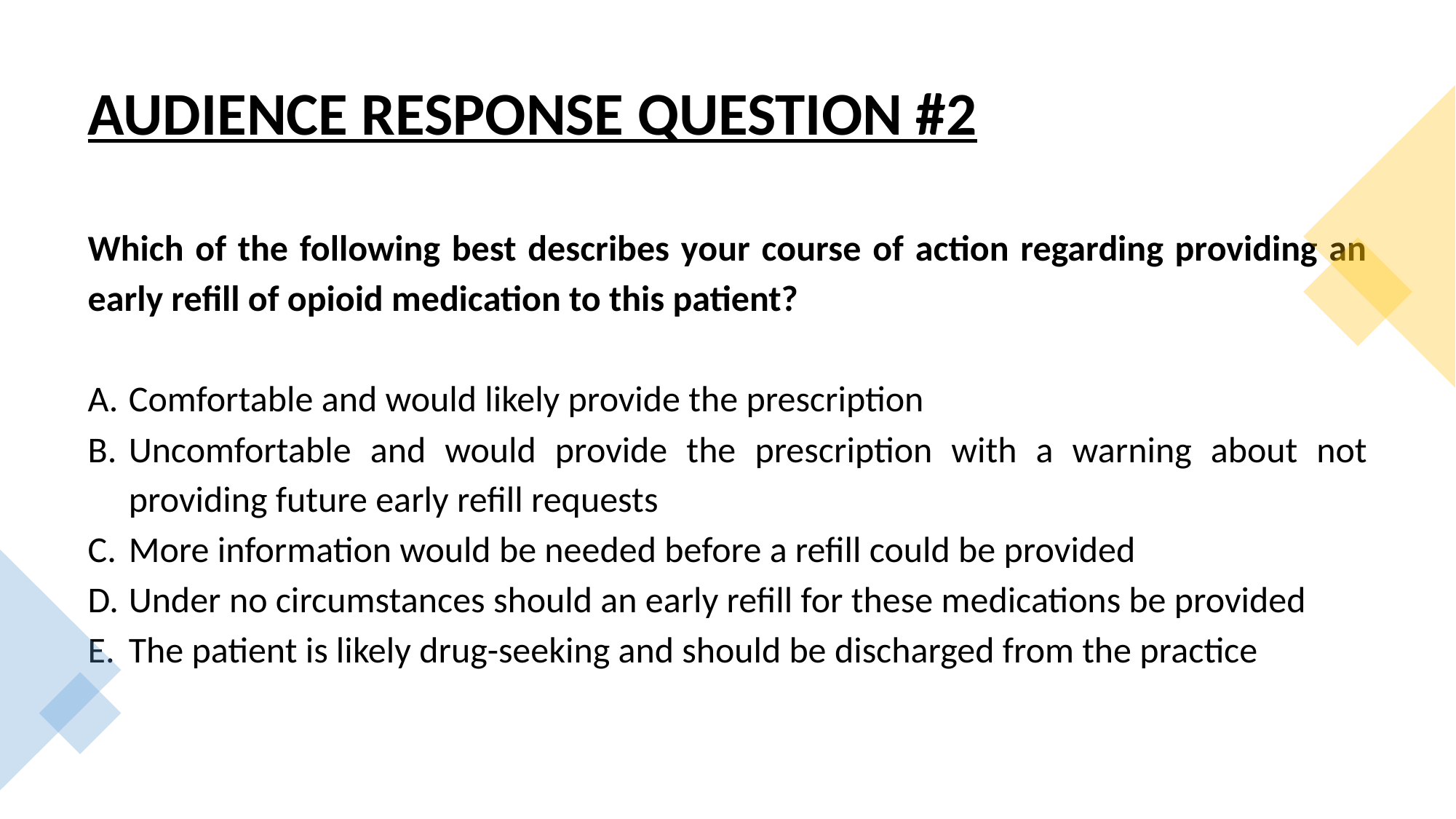

# AUDIENCE RESPONSE QUESTION #2
Which of the following best describes your course of action regarding providing an early refill of opioid medication to this patient?
Comfortable and would likely provide the prescription
Uncomfortable and would provide the prescription with a warning about not providing future early refill requests
More information would be needed before a refill could be provided
Under no circumstances should an early refill for these medications be provided
The patient is likely drug-seeking and should be discharged from the practice

## Slide 12
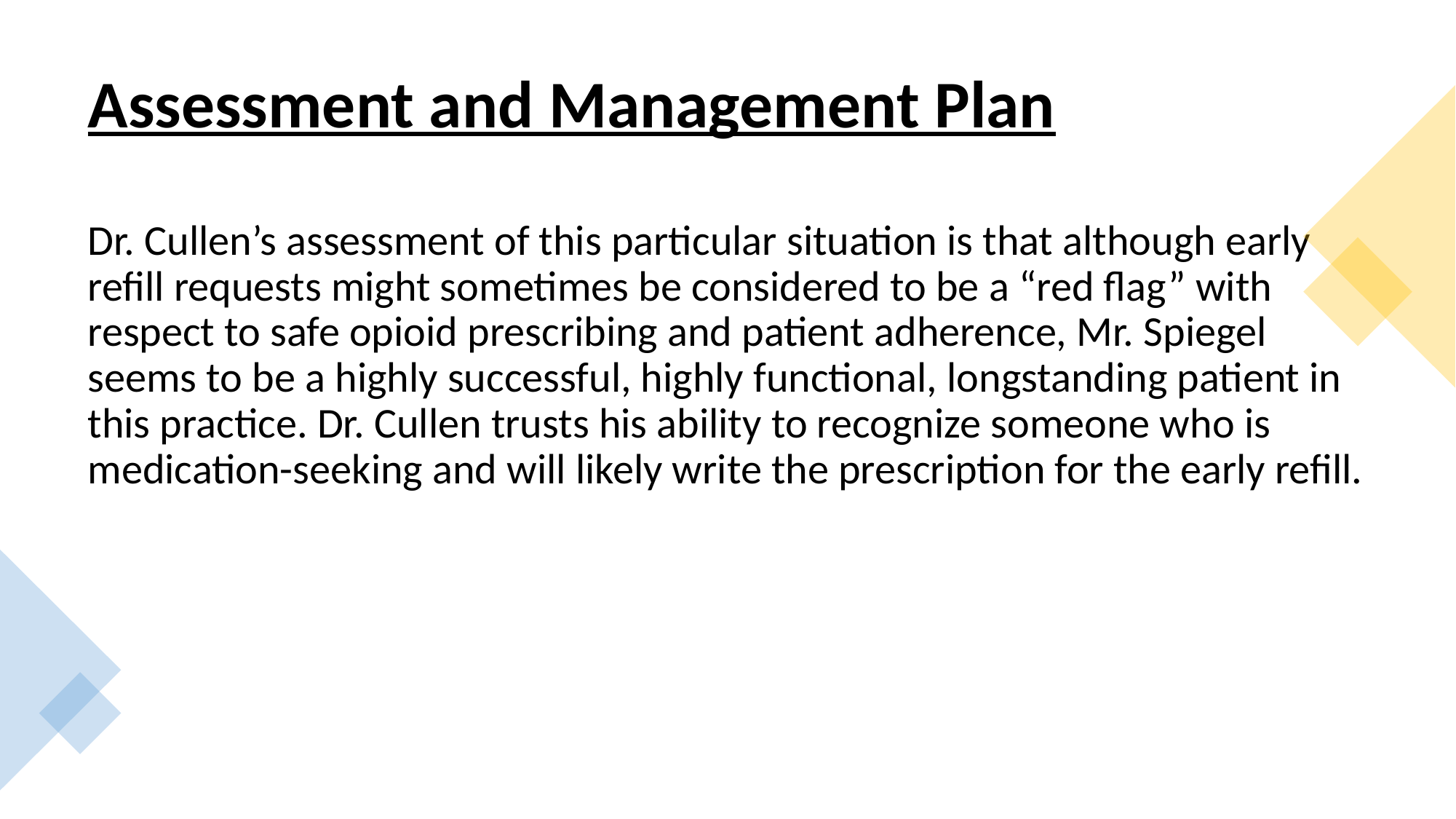

# Assessment and Management Plan
Dr. Cullen’s assessment of this particular situation is that although early refill requests might sometimes be considered to be a “red flag” with respect to safe opioid prescribing and patient adherence, Mr. Spiegel seems to be a highly successful, highly functional, longstanding patient in this practice. Dr. Cullen trusts his ability to recognize someone who is medication-seeking and will likely write the prescription for the early refill.

## Slide 13
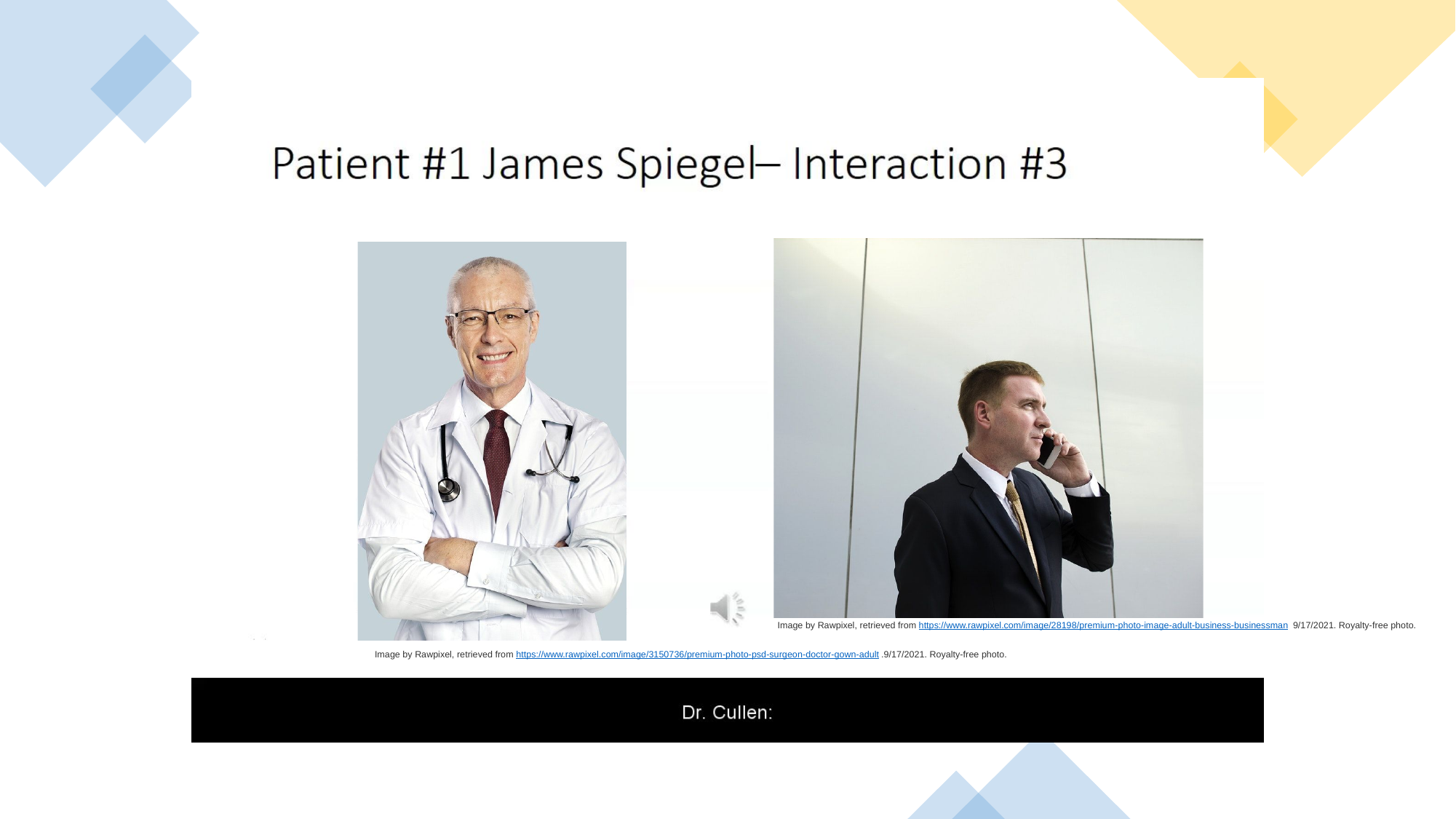

Image by Rawpixel, retrieved from https://www.rawpixel.com/image/28198/premium-photo-image-adult-business-businessman 9/17/2021. Royalty-free photo.
Image by Rawpixel, retrieved from https://www.rawpixel.com/image/3150736/premium-photo-psd-surgeon-doctor-gown-adult .9/17/2021. Royalty-free photo.

## Slide 14
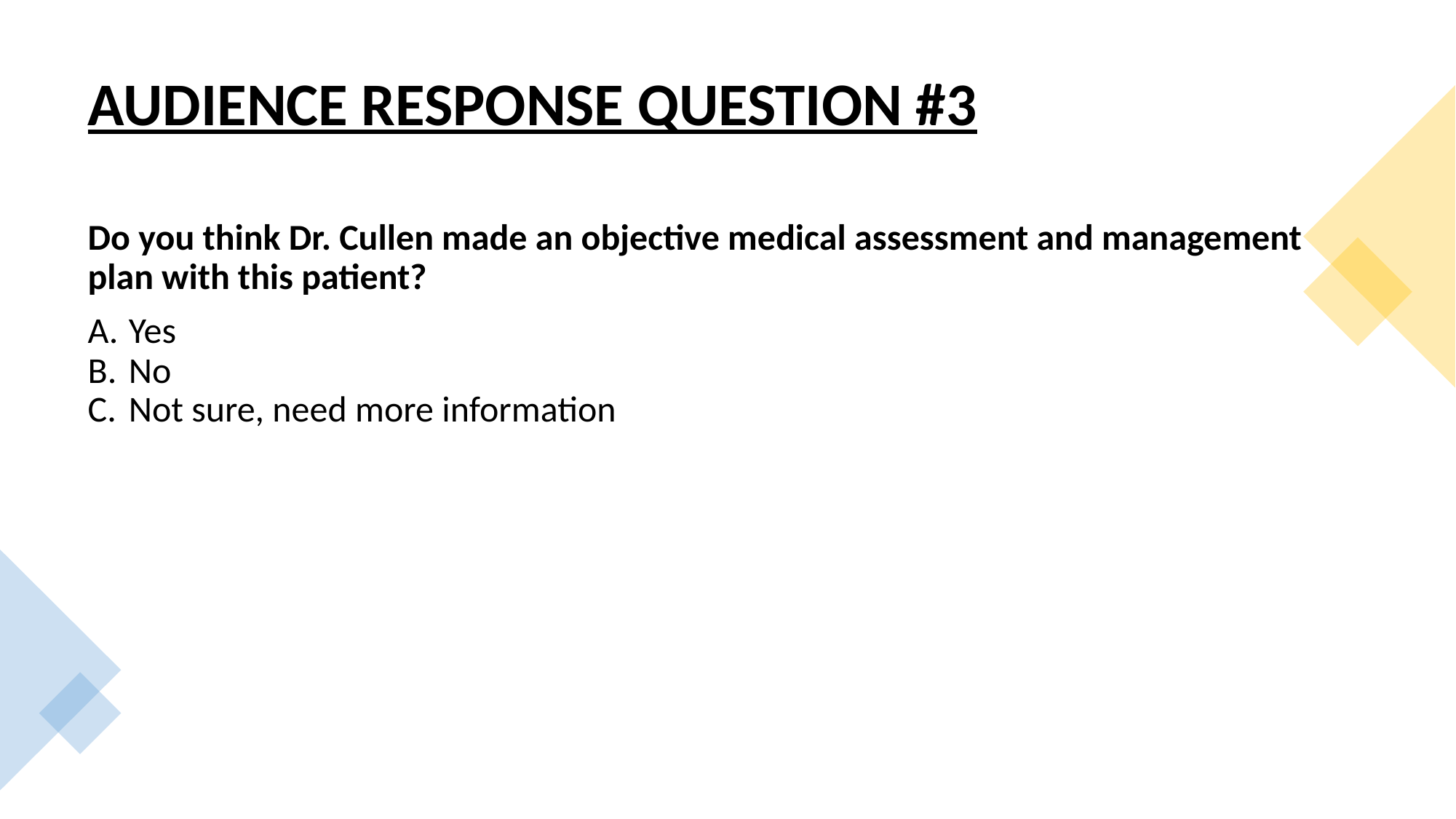

# AUDIENCE RESPONSE QUESTION #3
Do you think Dr. Cullen made an objective medical assessment and management plan with this patient?
Yes
No
Not sure, need more information

## Slide 15
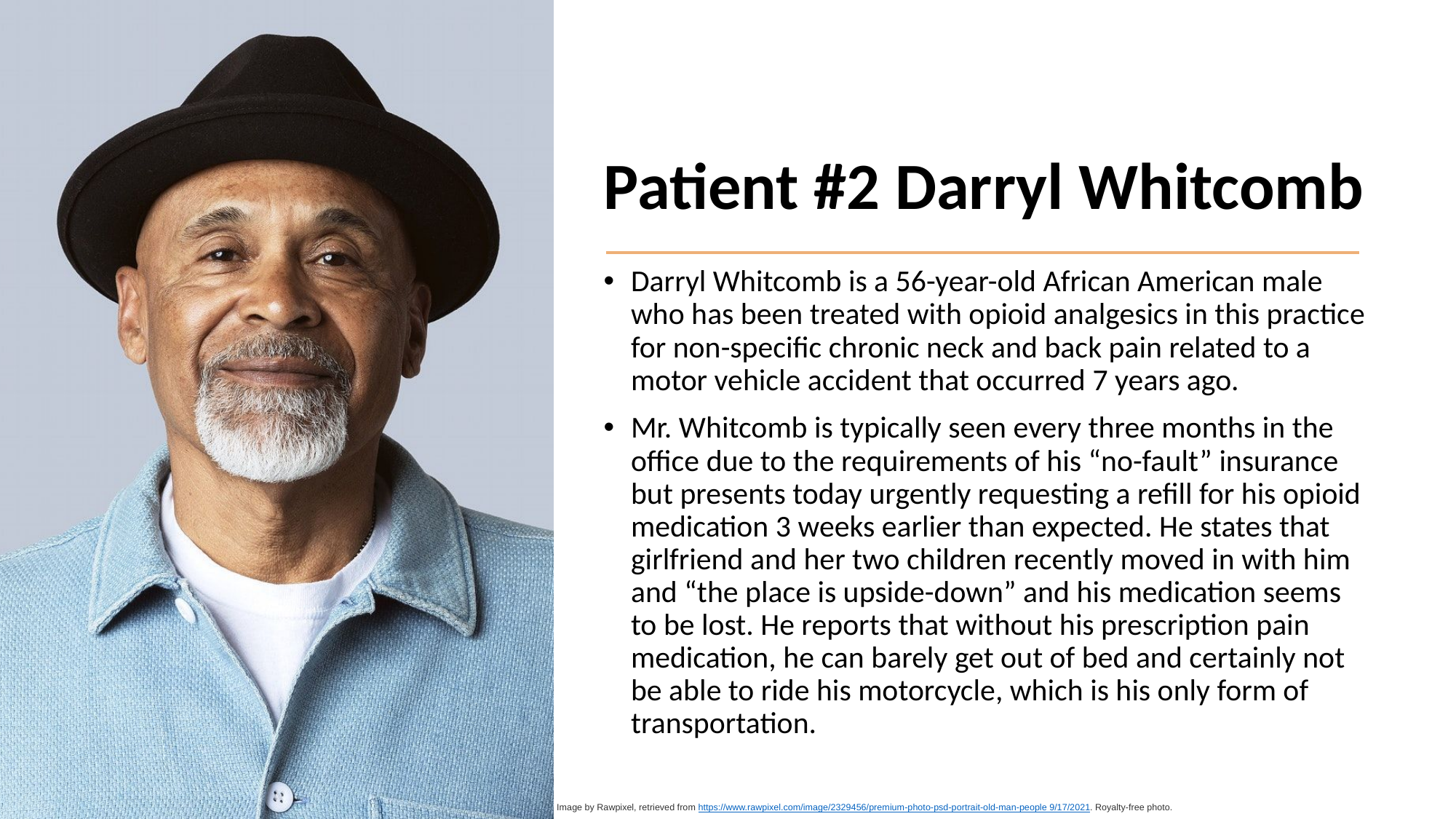

# Patient #2 Darryl Whitcomb
Darryl Whitcomb is a 56-year-old African American male who has been treated with opioid analgesics in this practice for non-specific chronic neck and back pain related to a motor vehicle accident that occurred 7 years ago.
Mr. Whitcomb is typically seen every three months in the office due to the requirements of his “no-fault” insurance but presents today urgently requesting a refill for his opioid medication 3 weeks earlier than expected. He states that girlfriend and her two children recently moved in with him and “the place is upside-down” and his medication seems to be lost. He reports that without his prescription pain medication, he can barely get out of bed and certainly not be able to ride his motorcycle, which is his only form of transportation.
Image by Rawpixel, retrieved from https://www.rawpixel.com/image/2329456/premium-photo-psd-portrait-old-man-people 9/17/2021. Royalty-free photo.

## Slide 16
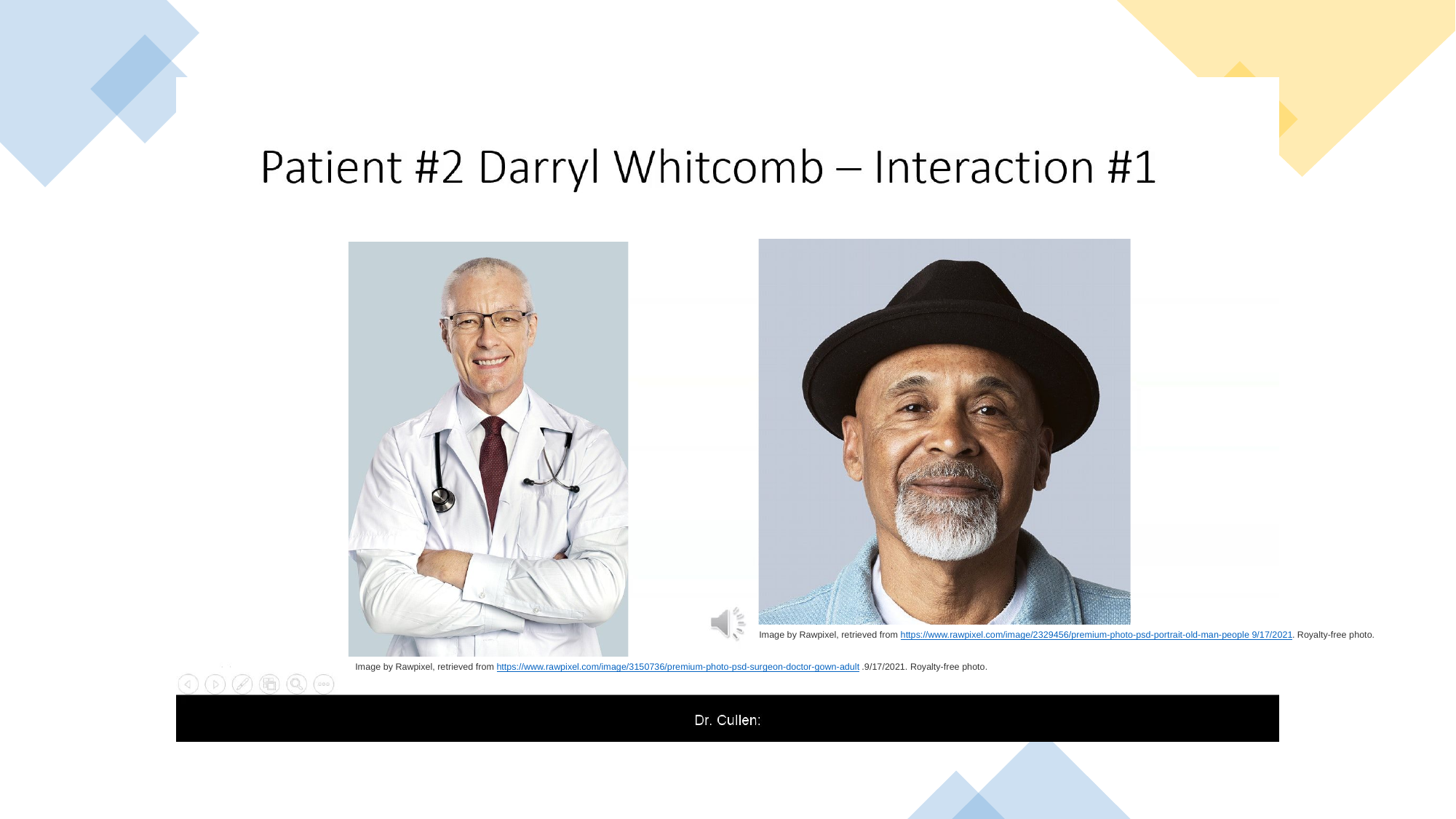

Image by Rawpixel, retrieved from https://www.rawpixel.com/image/2329456/premium-photo-psd-portrait-old-man-people 9/17/2021. Royalty-free photo.
Image by Rawpixel, retrieved from https://www.rawpixel.com/image/3150736/premium-photo-psd-surgeon-doctor-gown-adult .9/17/2021. Royalty-free photo.

## Slide 17
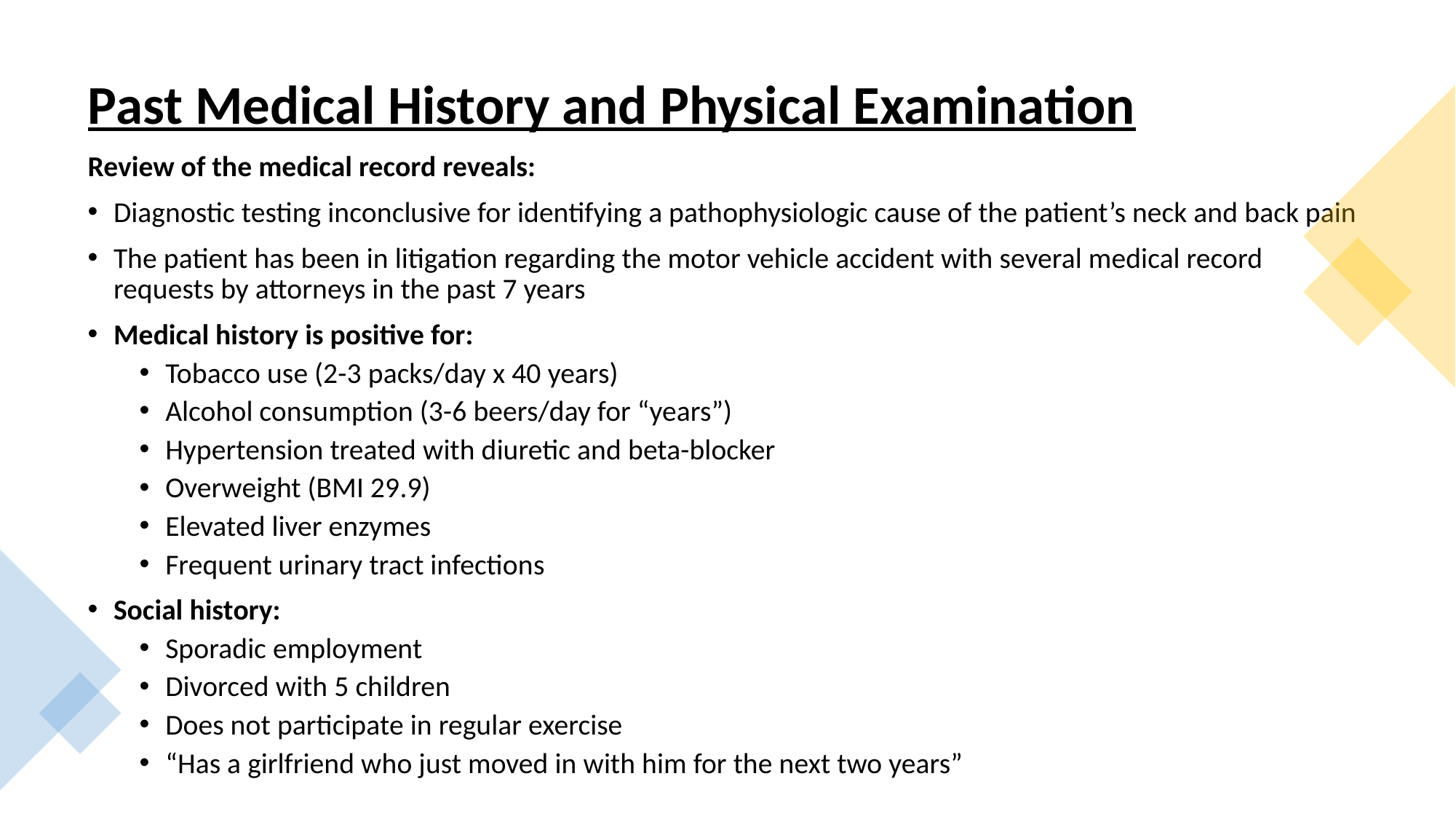

# Past Medical History and Physical Examination
Review of the medical record reveals:
Diagnostic testing inconclusive for identifying a pathophysiologic cause of the patient’s neck and back pain
The patient has been in litigation regarding the motor vehicle accident with several medical record requests by attorneys in the past 7 years
Medical history is positive for:
Tobacco use (2-3 packs/day x 40 years)
Alcohol consumption (3-6 beers/day for “years”)
Hypertension treated with diuretic and beta-blocker
Overweight (BMI 29.9)
Elevated liver enzymes
Frequent urinary tract infections
Social history:
Sporadic employment
Divorced with 5 children
Does not participate in regular exercise
“Has a girlfriend who just moved in with him for the next two years”

## Slide 18
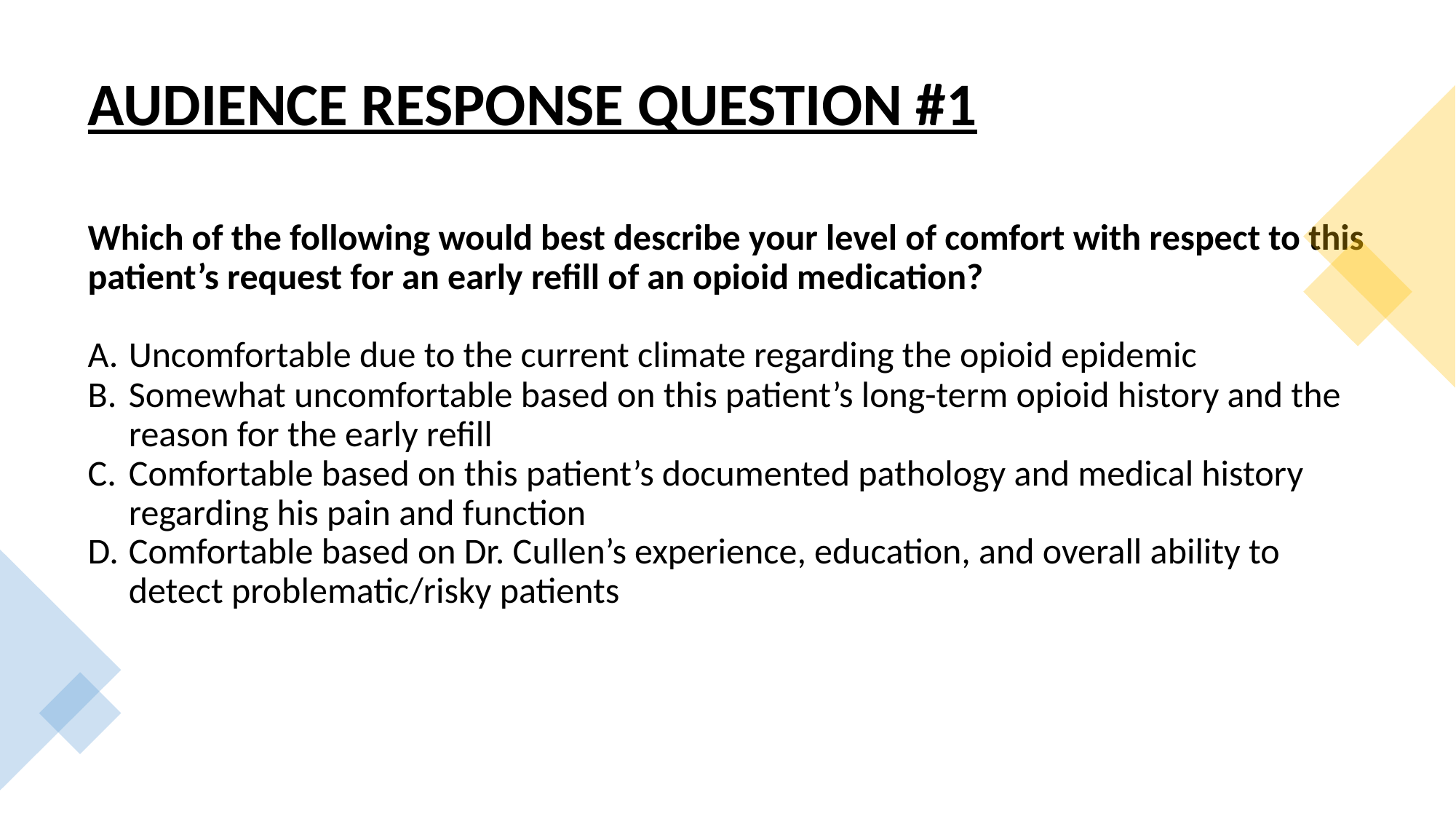

# AUDIENCE RESPONSE QUESTION #1
Which of the following would best describe your level of comfort with respect to this patient’s request for an early refill of an opioid medication?
Uncomfortable due to the current climate regarding the opioid epidemic
Somewhat uncomfortable based on this patient’s long-term opioid history and the reason for the early refill
Comfortable based on this patient’s documented pathology and medical history regarding his pain and function
Comfortable based on Dr. Cullen’s experience, education, and overall ability to detect problematic/risky patients

## Slide 19
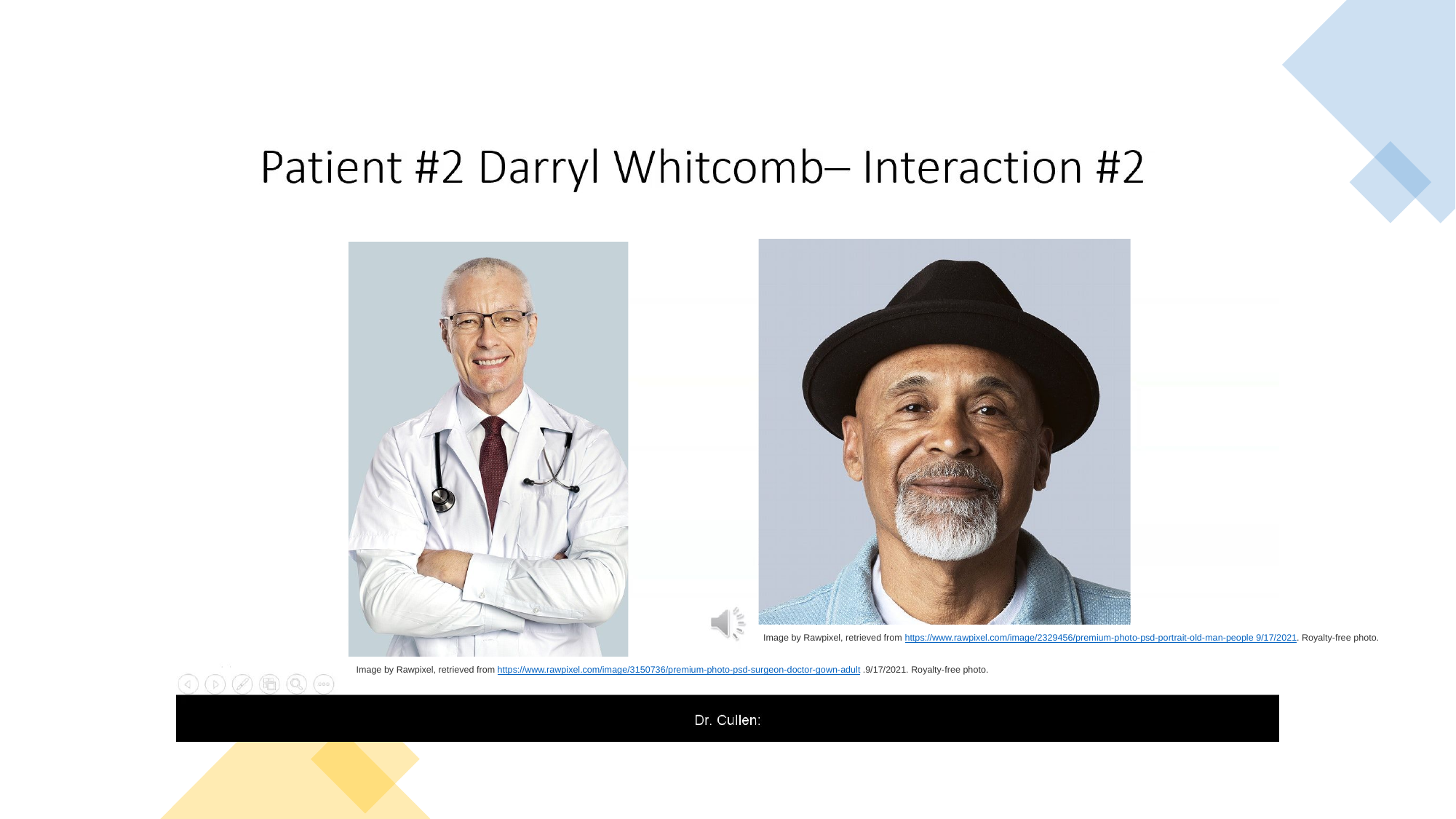

Image by Rawpixel, retrieved from https://www.rawpixel.com/image/2329456/premium-photo-psd-portrait-old-man-people 9/17/2021. Royalty-free photo.
Image by Rawpixel, retrieved from https://www.rawpixel.com/image/3150736/premium-photo-psd-surgeon-doctor-gown-adult .9/17/2021. Royalty-free photo.

## Slide 20
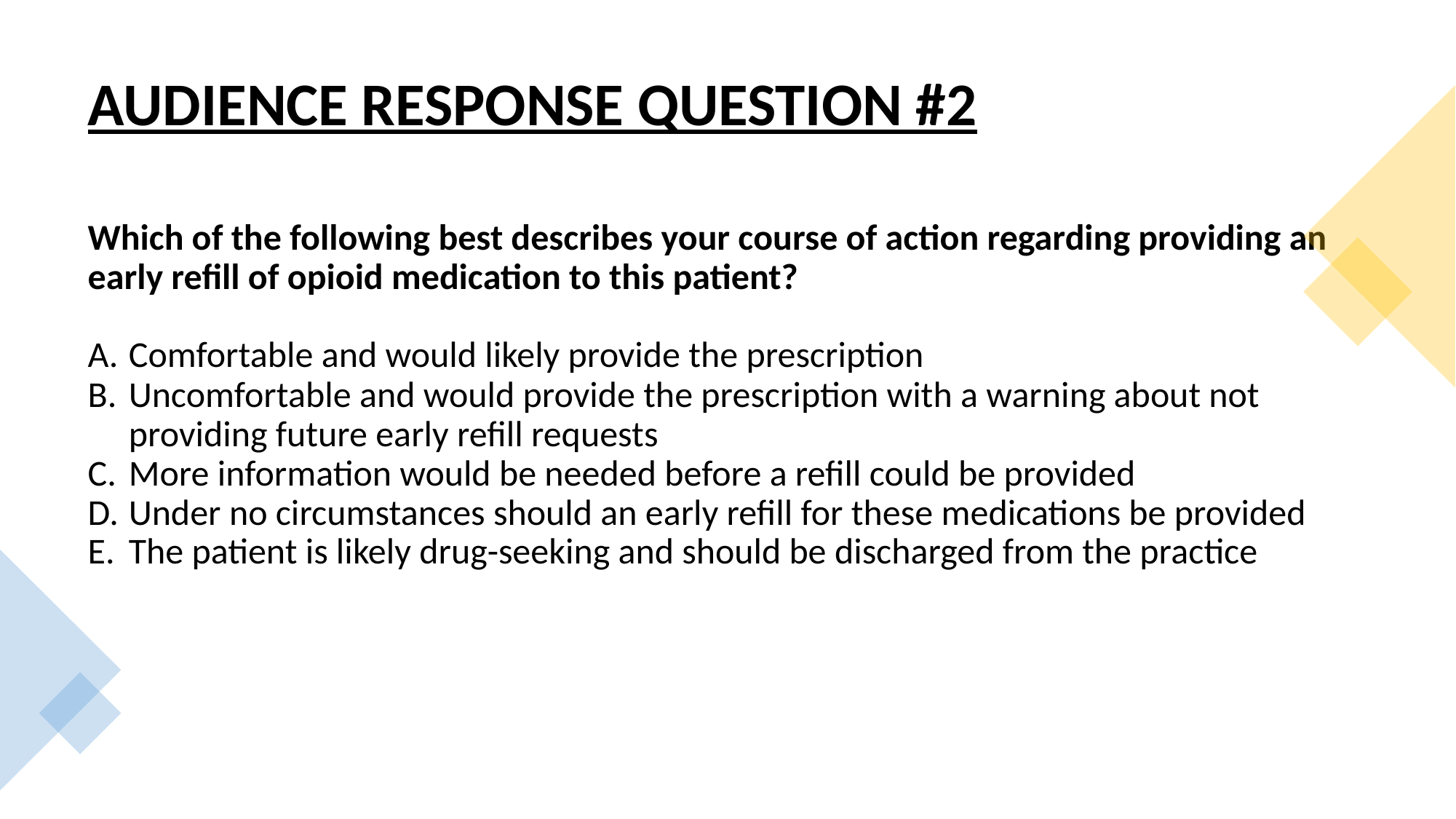

# AUDIENCE RESPONSE QUESTION #2
Which of the following best describes your course of action regarding providing an early refill of opioid medication to this patient?
Comfortable and would likely provide the prescription
Uncomfortable and would provide the prescription with a warning about not providing future early refill requests
More information would be needed before a refill could be provided
Under no circumstances should an early refill for these medications be provided
The patient is likely drug-seeking and should be discharged from the practice

## Slide 21
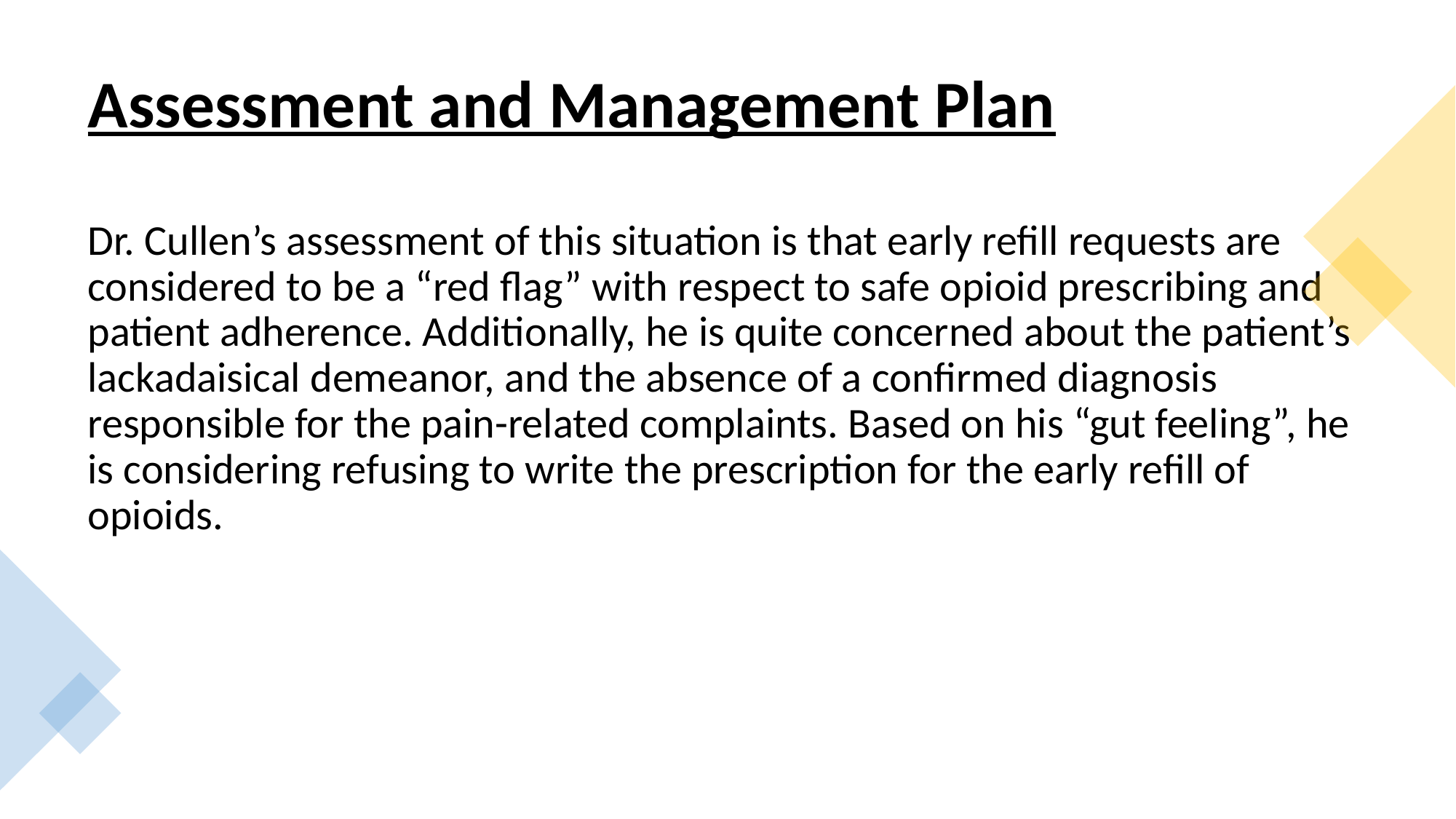

# Assessment and Management Plan
Dr. Cullen’s assessment of this situation is that early refill requests are considered to be a “red flag” with respect to safe opioid prescribing and patient adherence. Additionally, he is quite concerned about the patient’s lackadaisical demeanor, and the absence of a confirmed diagnosis responsible for the pain-related complaints. Based on his “gut feeling”, he is considering refusing to write the prescription for the early refill of opioids.

## Slide 22
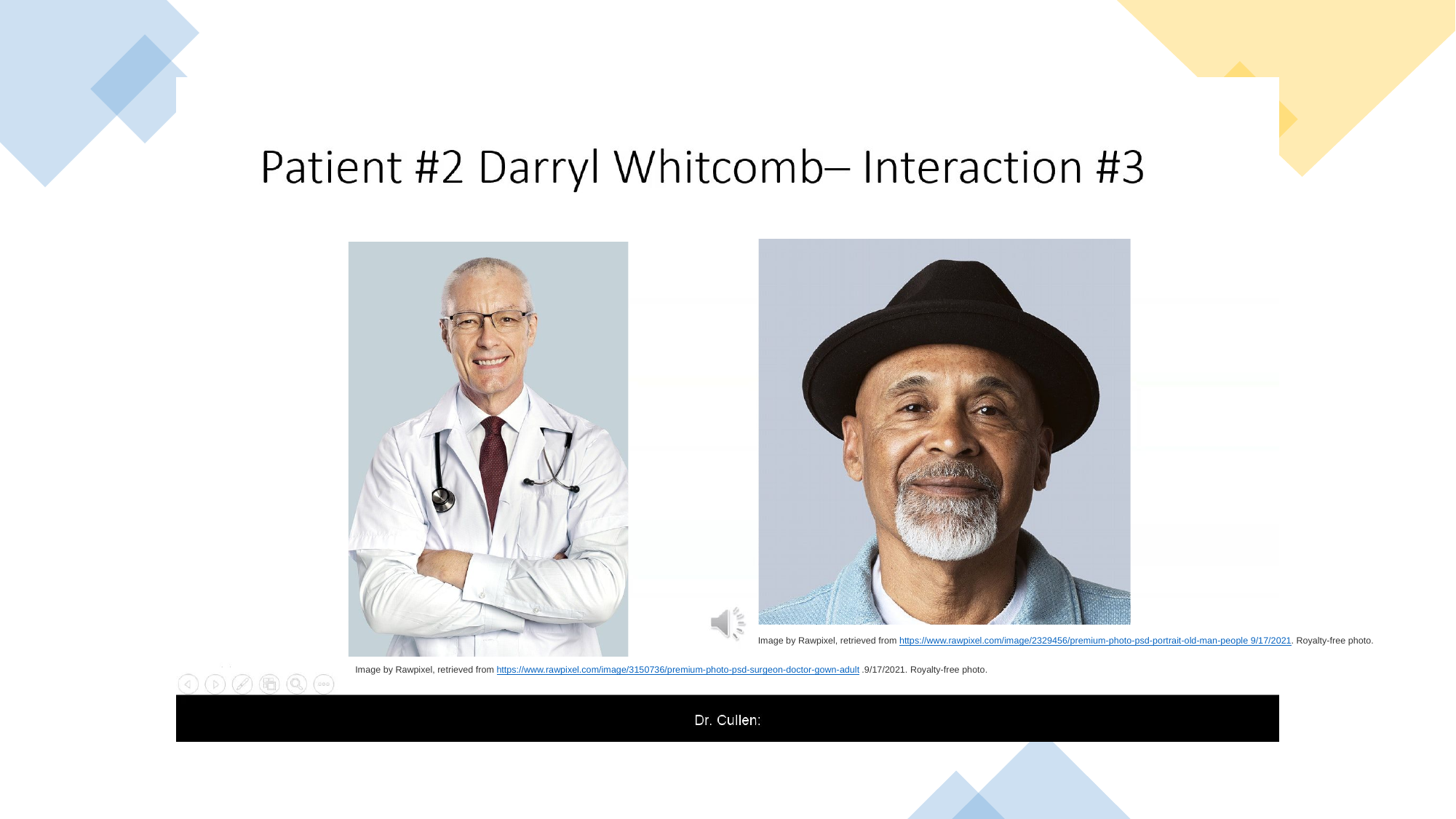

Image by Rawpixel, retrieved from https://www.rawpixel.com/image/2329456/premium-photo-psd-portrait-old-man-people 9/17/2021. Royalty-free photo.
Image by Rawpixel, retrieved from https://www.rawpixel.com/image/3150736/premium-photo-psd-surgeon-doctor-gown-adult .9/17/2021. Royalty-free photo.

## Slide 23
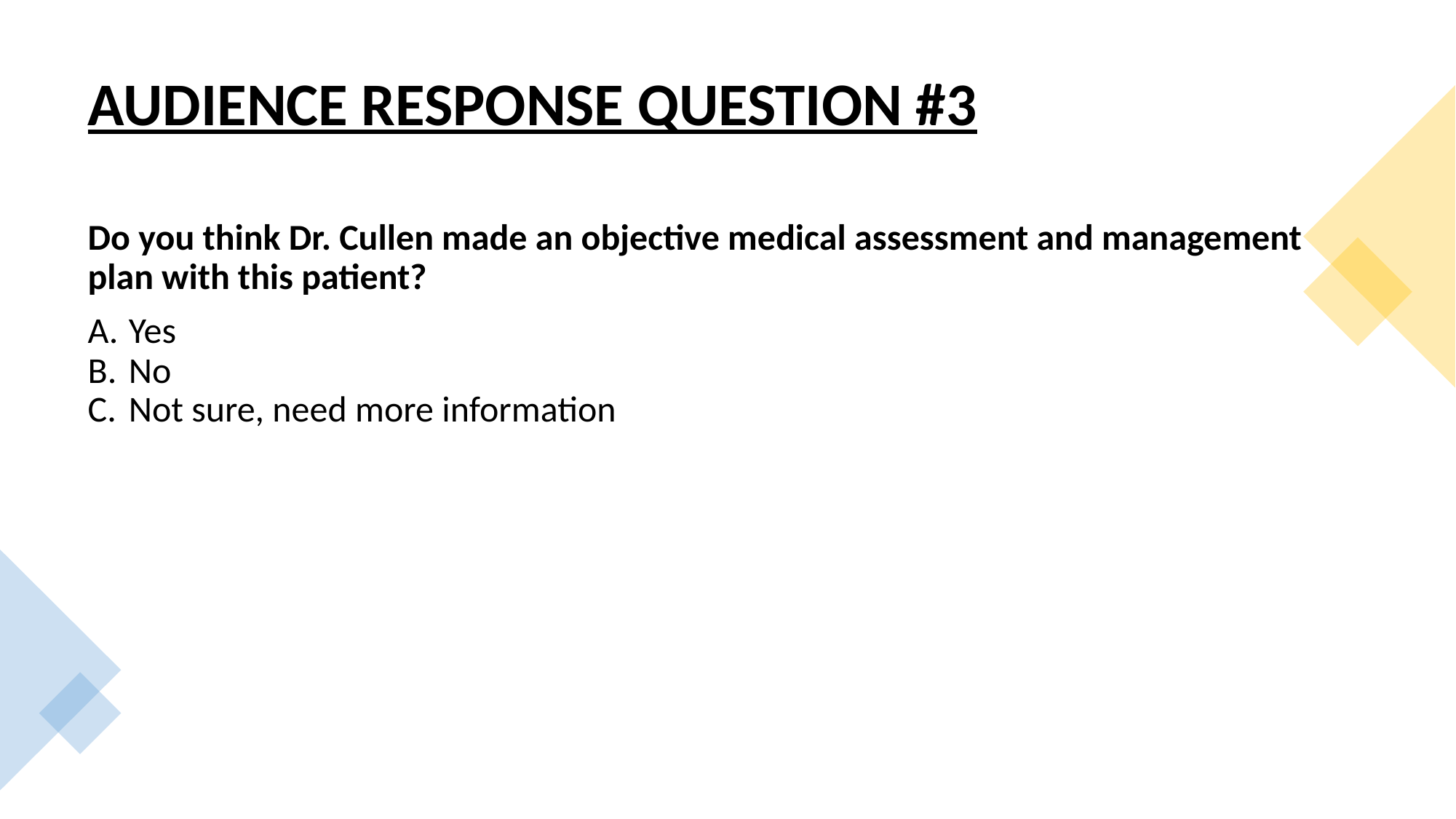

# AUDIENCE RESPONSE QUESTION #3
Do you think Dr. Cullen made an objective medical assessment and management plan with this patient?
Yes
No
Not sure, need more information

## Slide 24
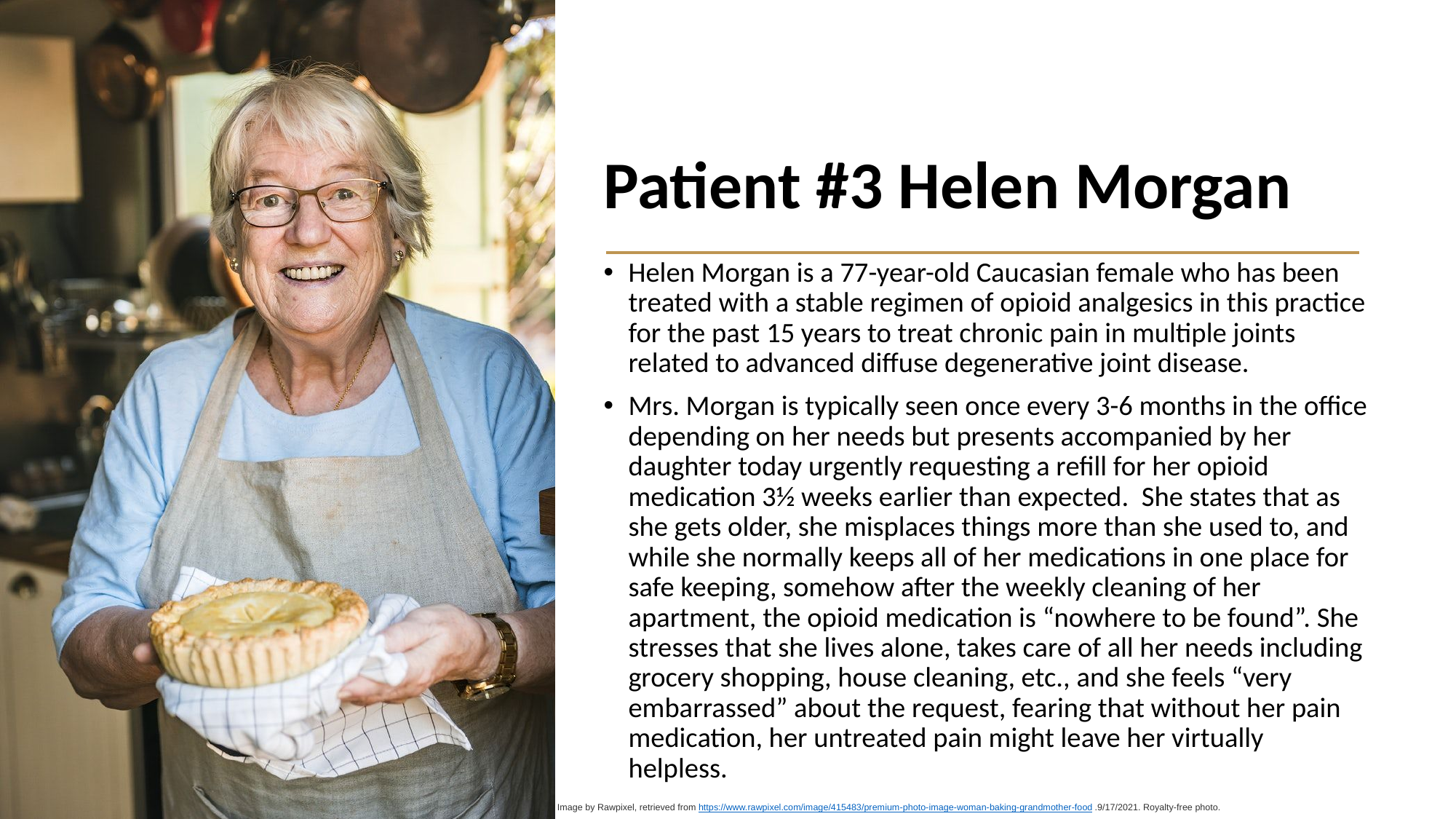

# Patient #3 Helen Morgan
Helen Morgan is a 77-year-old Caucasian female who has been treated with a stable regimen of opioid analgesics in this practice for the past 15 years to treat chronic pain in multiple joints related to advanced diffuse degenerative joint disease.
Mrs. Morgan is typically seen once every 3-6 months in the office depending on her needs but presents accompanied by her daughter today urgently requesting a refill for her opioid medication 3½ weeks earlier than expected. She states that as she gets older, she misplaces things more than she used to, and while she normally keeps all of her medications in one place for safe keeping, somehow after the weekly cleaning of her apartment, the opioid medication is “nowhere to be found”. She stresses that she lives alone, takes care of all her needs including grocery shopping, house cleaning, etc., and she feels “very embarrassed” about the request, fearing that without her pain medication, her untreated pain might leave her virtually helpless.
Image by Rawpixel, retrieved from https://www.rawpixel.com/image/415483/premium-photo-image-woman-baking-grandmother-food .9/17/2021. Royalty-free photo.

## Slide 25
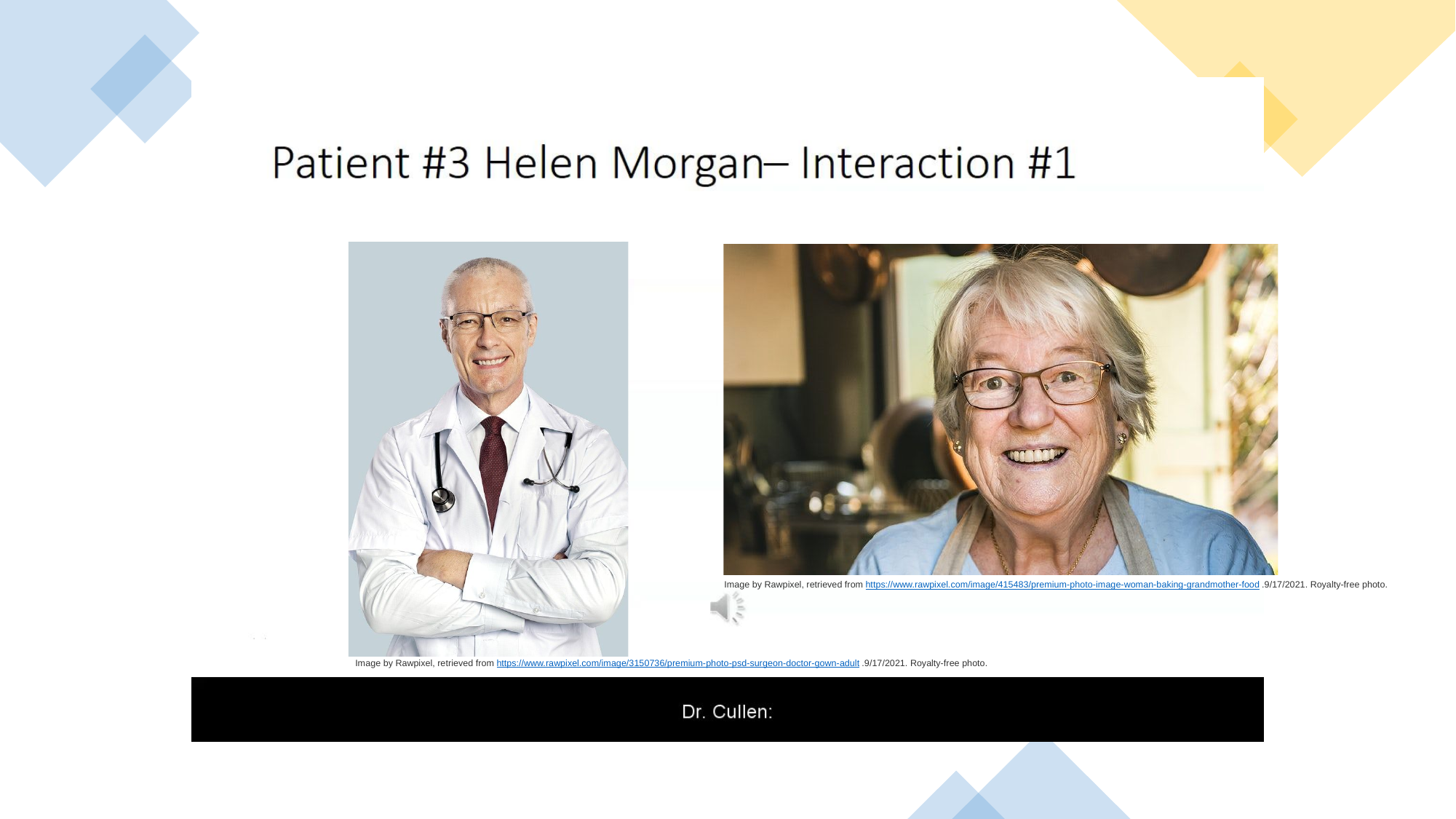

Image by Rawpixel, retrieved from https://www.rawpixel.com/image/415483/premium-photo-image-woman-baking-grandmother-food .9/17/2021. Royalty-free photo.
Image by Rawpixel, retrieved from https://www.rawpixel.com/image/3150736/premium-photo-psd-surgeon-doctor-gown-adult .9/17/2021. Royalty-free photo.

## Slide 26
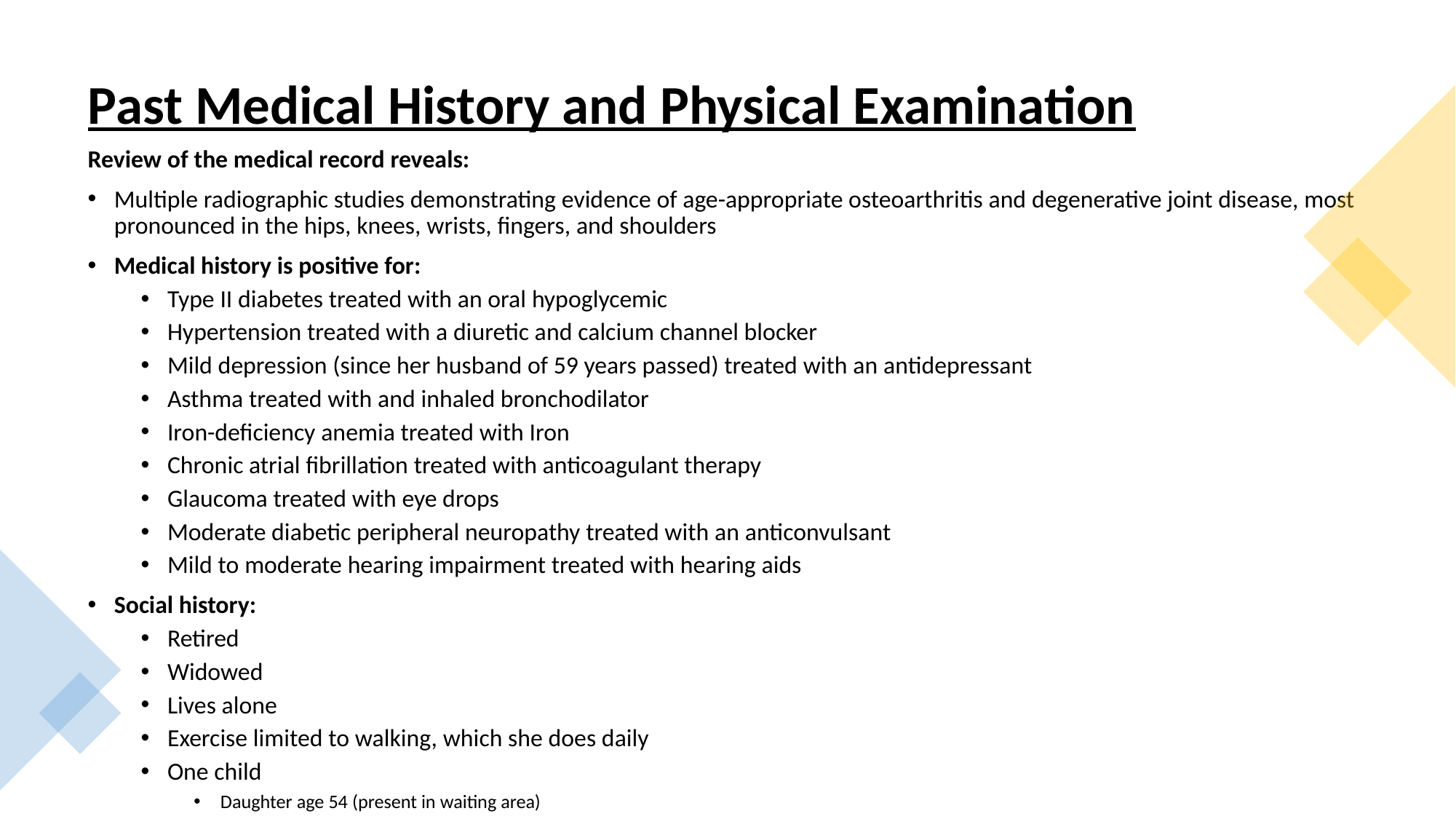

# Past Medical History and Physical Examination
Review of the medical record reveals:
Multiple radiographic studies demonstrating evidence of age-appropriate osteoarthritis and degenerative joint disease, most pronounced in the hips, knees, wrists, fingers, and shoulders
Medical history is positive for:
Type II diabetes treated with an oral hypoglycemic
Hypertension treated with a diuretic and calcium channel blocker
Mild depression (since her husband of 59 years passed) treated with an antidepressant
Asthma treated with and inhaled bronchodilator
Iron-deficiency anemia treated with Iron
Chronic atrial fibrillation treated with anticoagulant therapy
Glaucoma treated with eye drops
Moderate diabetic peripheral neuropathy treated with an anticonvulsant
Mild to moderate hearing impairment treated with hearing aids
Social history:
Retired
Widowed
Lives alone
Exercise limited to walking, which she does daily
One child
Daughter age 54 (present in waiting area)

## Slide 27
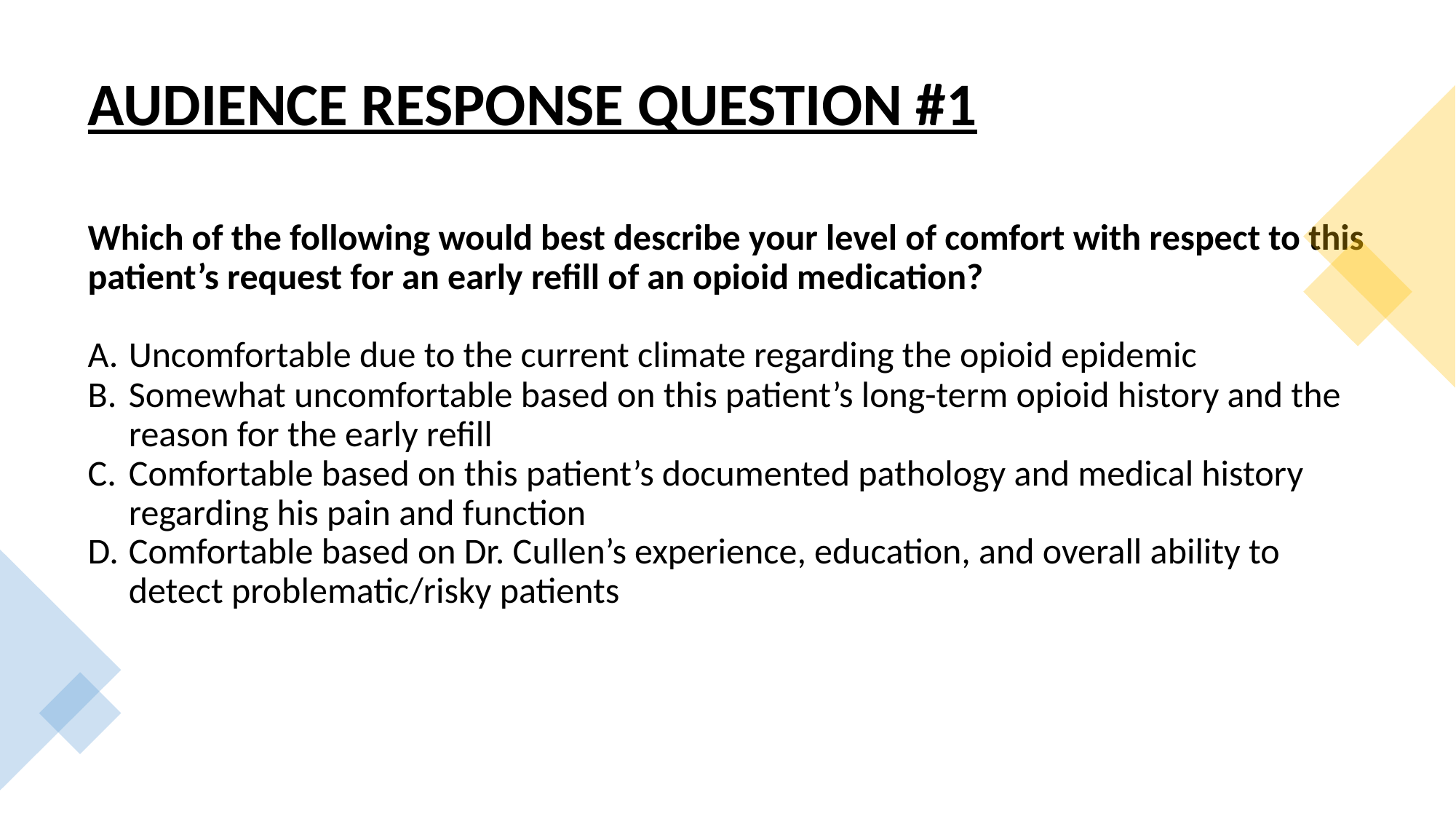

# AUDIENCE RESPONSE QUESTION #1
Which of the following would best describe your level of comfort with respect to this patient’s request for an early refill of an opioid medication?
Uncomfortable due to the current climate regarding the opioid epidemic
Somewhat uncomfortable based on this patient’s long-term opioid history and the reason for the early refill
Comfortable based on this patient’s documented pathology and medical history regarding his pain and function
Comfortable based on Dr. Cullen’s experience, education, and overall ability to detect problematic/risky patients

## Slide 28
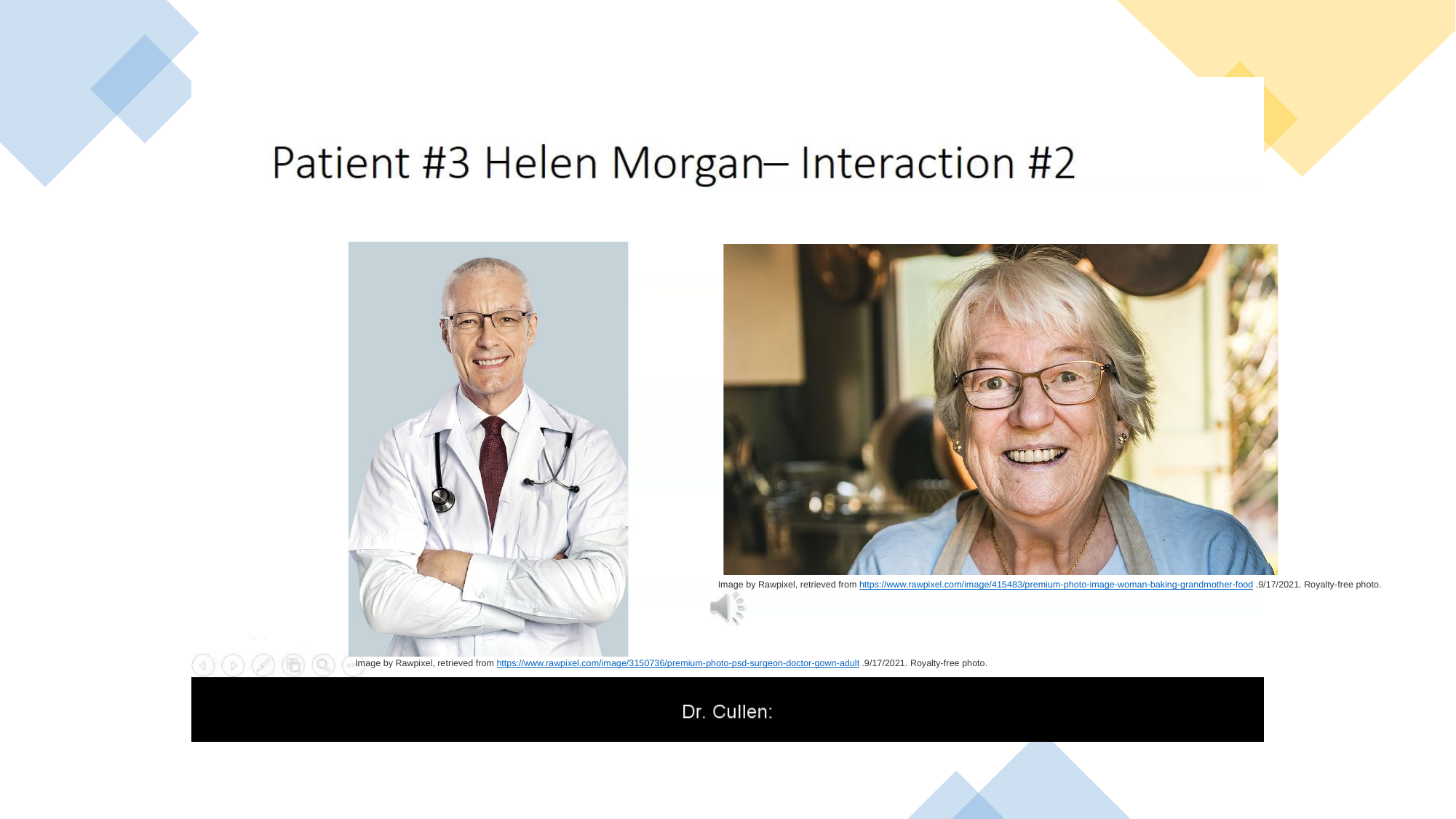

Image by Rawpixel, retrieved from https://www.rawpixel.com/image/415483/premium-photo-image-woman-baking-grandmother-food .9/17/2021. Royalty-free photo.
Image by Rawpixel, retrieved from https://www.rawpixel.com/image/3150736/premium-photo-psd-surgeon-doctor-gown-adult .9/17/2021. Royalty-free photo.

## Slide 29
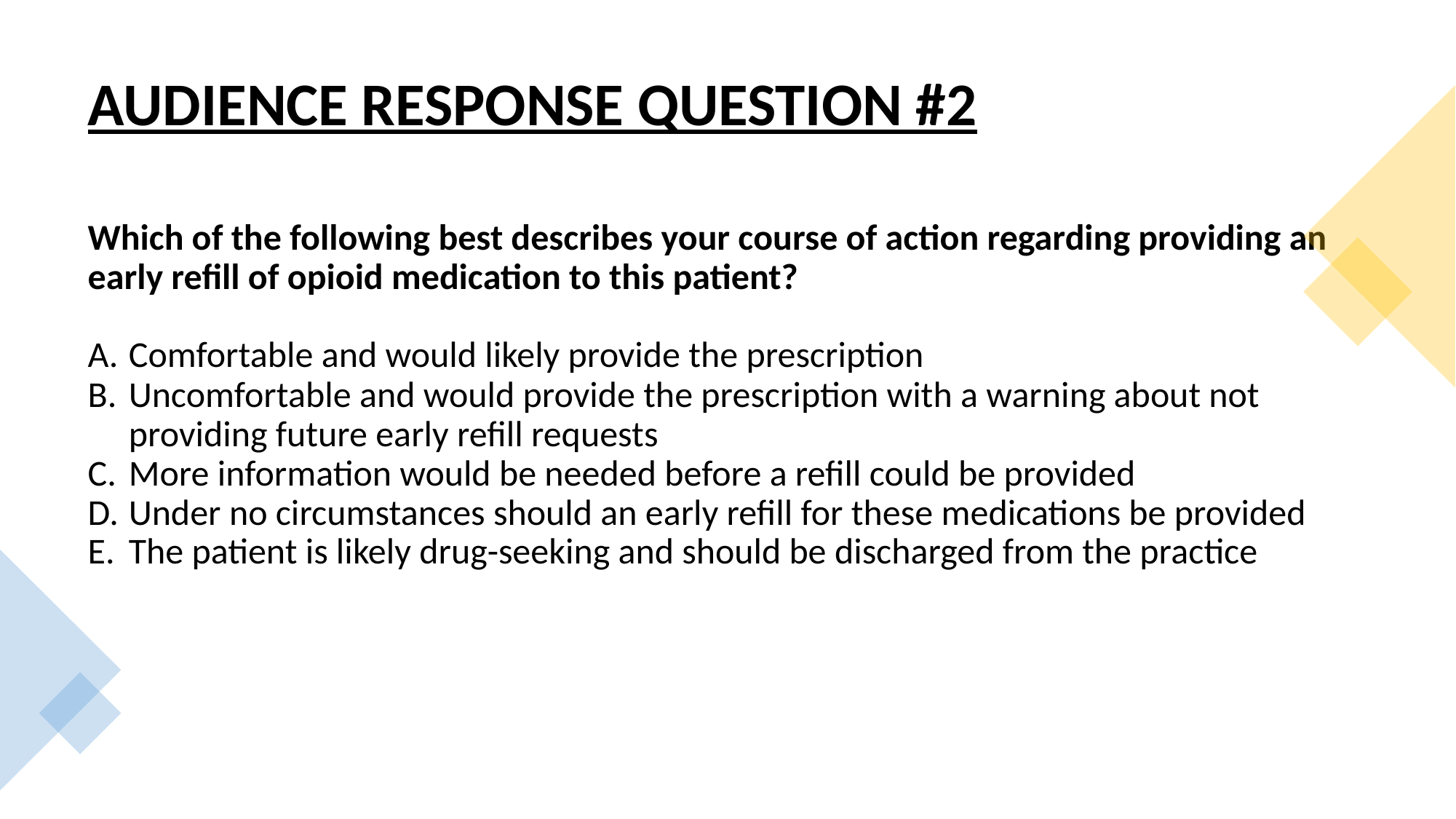

# AUDIENCE RESPONSE QUESTION #2
Which of the following best describes your course of action regarding providing an early refill of opioid medication to this patient?
Comfortable and would likely provide the prescription
Uncomfortable and would provide the prescription with a warning about not providing future early refill requests
More information would be needed before a refill could be provided
Under no circumstances should an early refill for these medications be provided
The patient is likely drug-seeking and should be discharged from the practice

## Slide 30
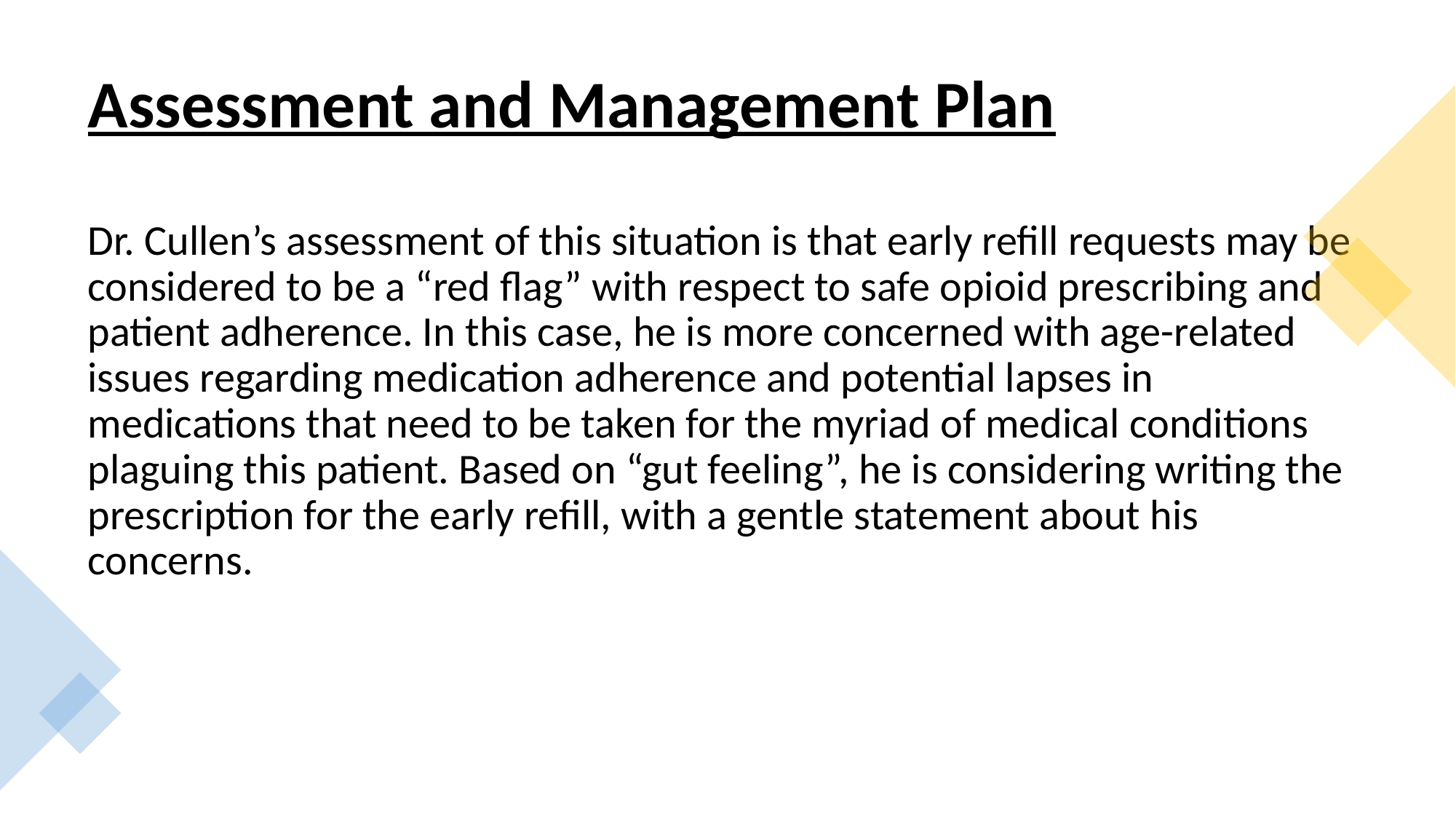

# Assessment and Management Plan
Dr. Cullen’s assessment of this situation is that early refill requests may be considered to be a “red flag” with respect to safe opioid prescribing and patient adherence. In this case, he is more concerned with age-related issues regarding medication adherence and potential lapses in medications that need to be taken for the myriad of medical conditions plaguing this patient. Based on “gut feeling”, he is considering writing the prescription for the early refill, with a gentle statement about his concerns.

## Slide 31
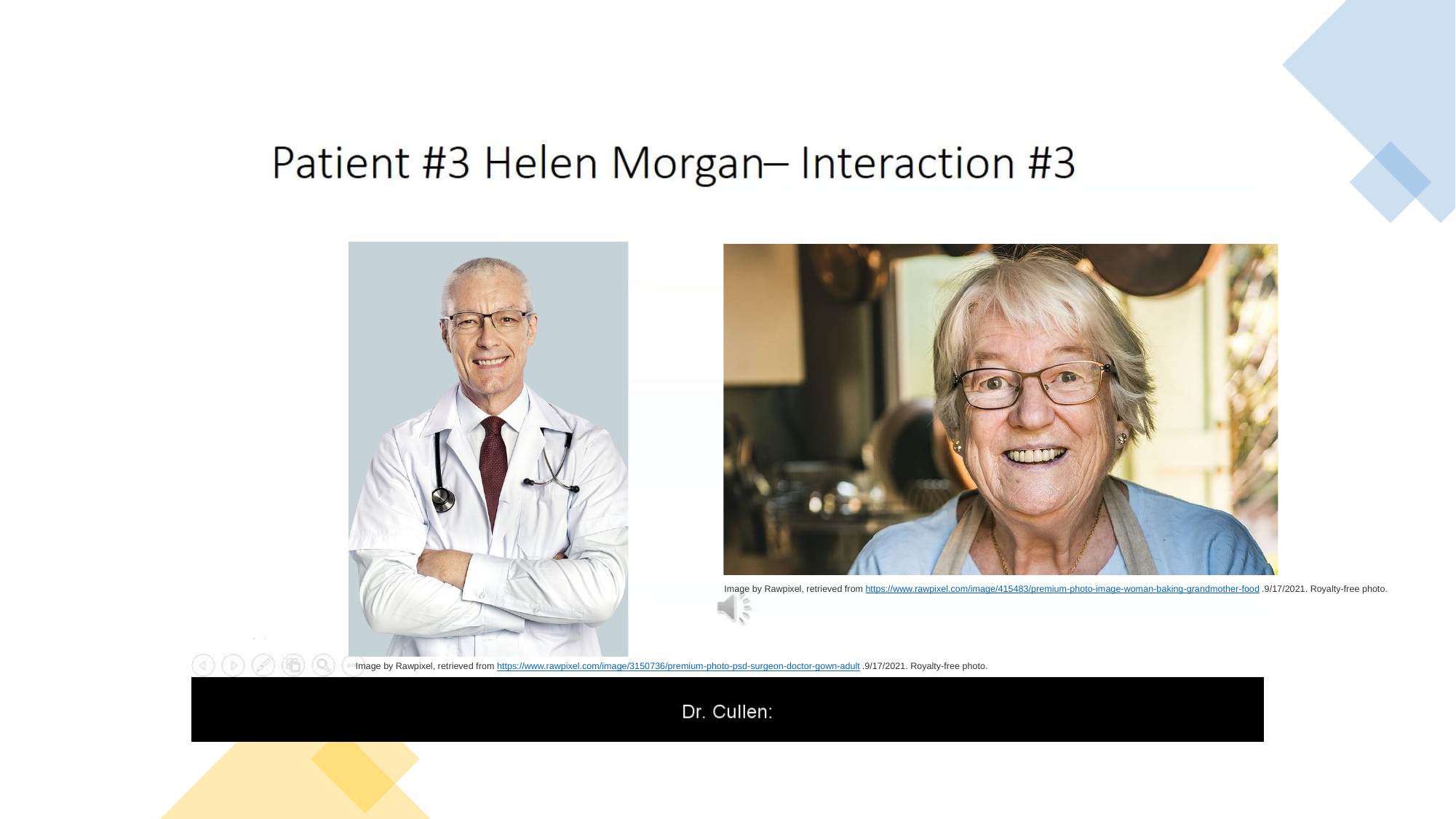

Image by Rawpixel, retrieved from https://www.rawpixel.com/image/415483/premium-photo-image-woman-baking-grandmother-food .9/17/2021. Royalty-free photo.
Image by Rawpixel, retrieved from https://www.rawpixel.com/image/3150736/premium-photo-psd-surgeon-doctor-gown-adult .9/17/2021. Royalty-free photo.

## Slide 32
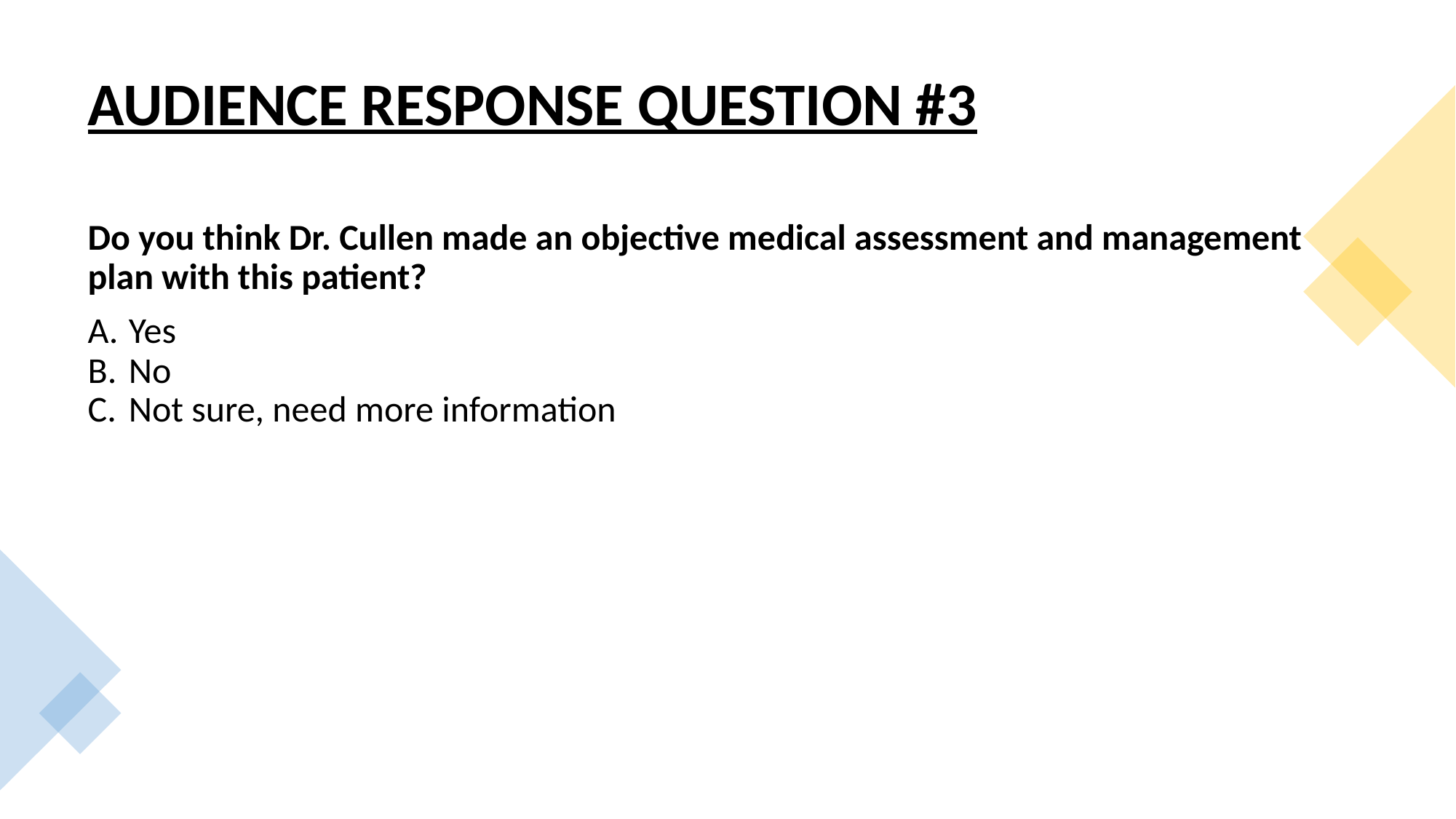

# AUDIENCE RESPONSE QUESTION #3
Do you think Dr. Cullen made an objective medical assessment and management plan with this patient?
Yes
No
Not sure, need more information

## Slide 33
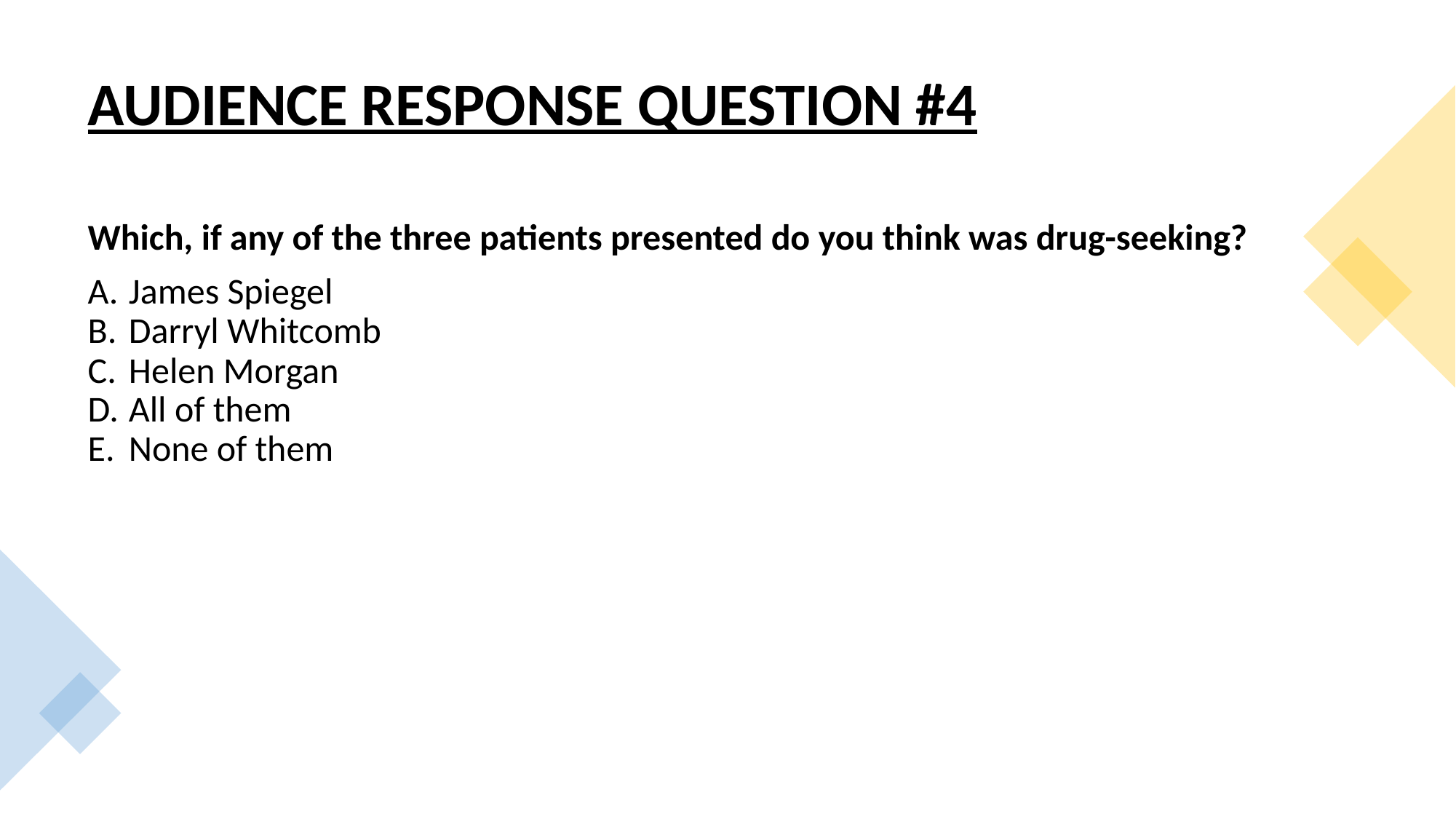

# AUDIENCE RESPONSE QUESTION #4
Which, if any of the three patients presented do you think was drug-seeking?
James Spiegel
Darryl Whitcomb
Helen Morgan
All of them
None of them

## Slide 34
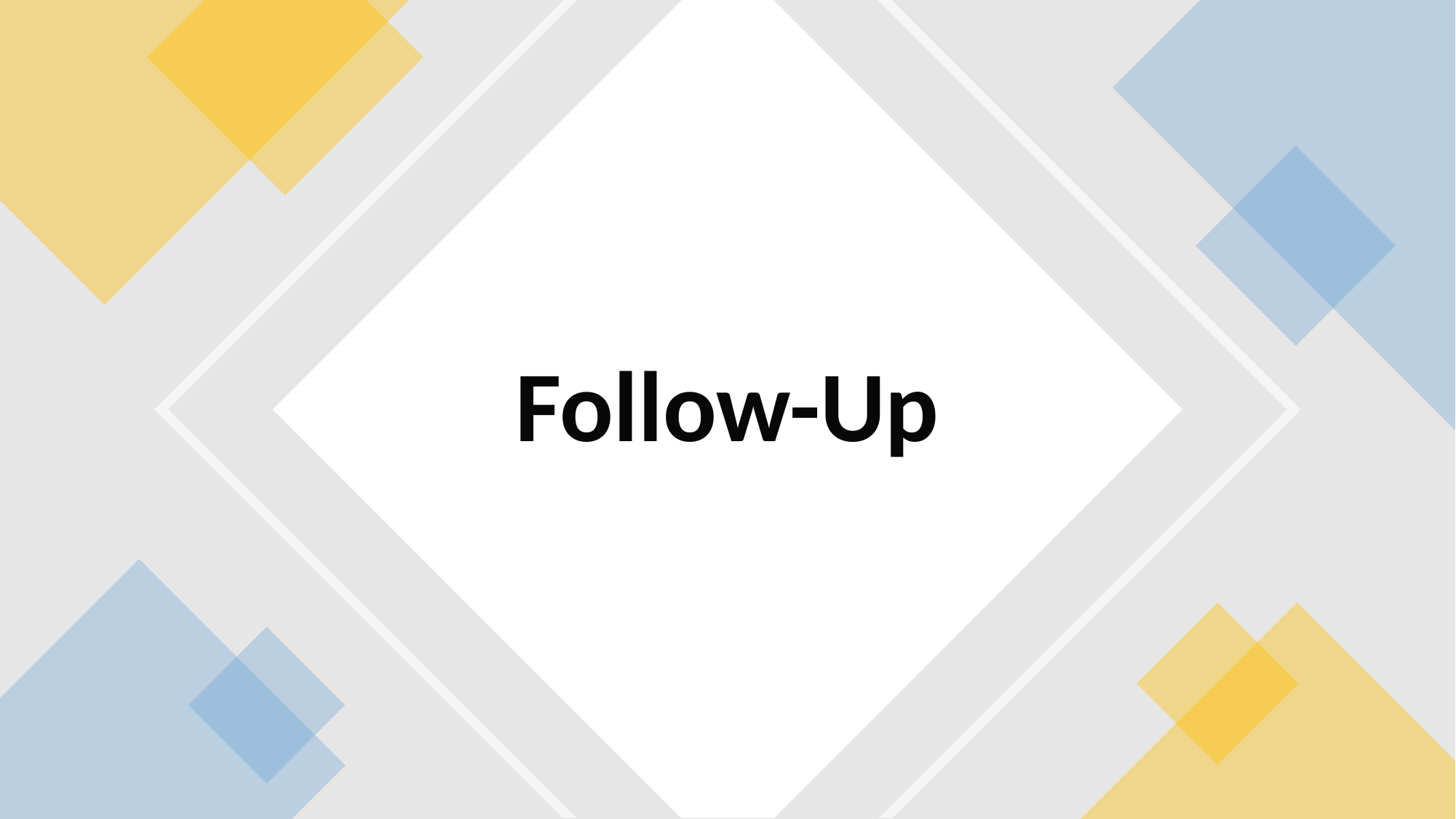

# Follow-Up

## Slide 35
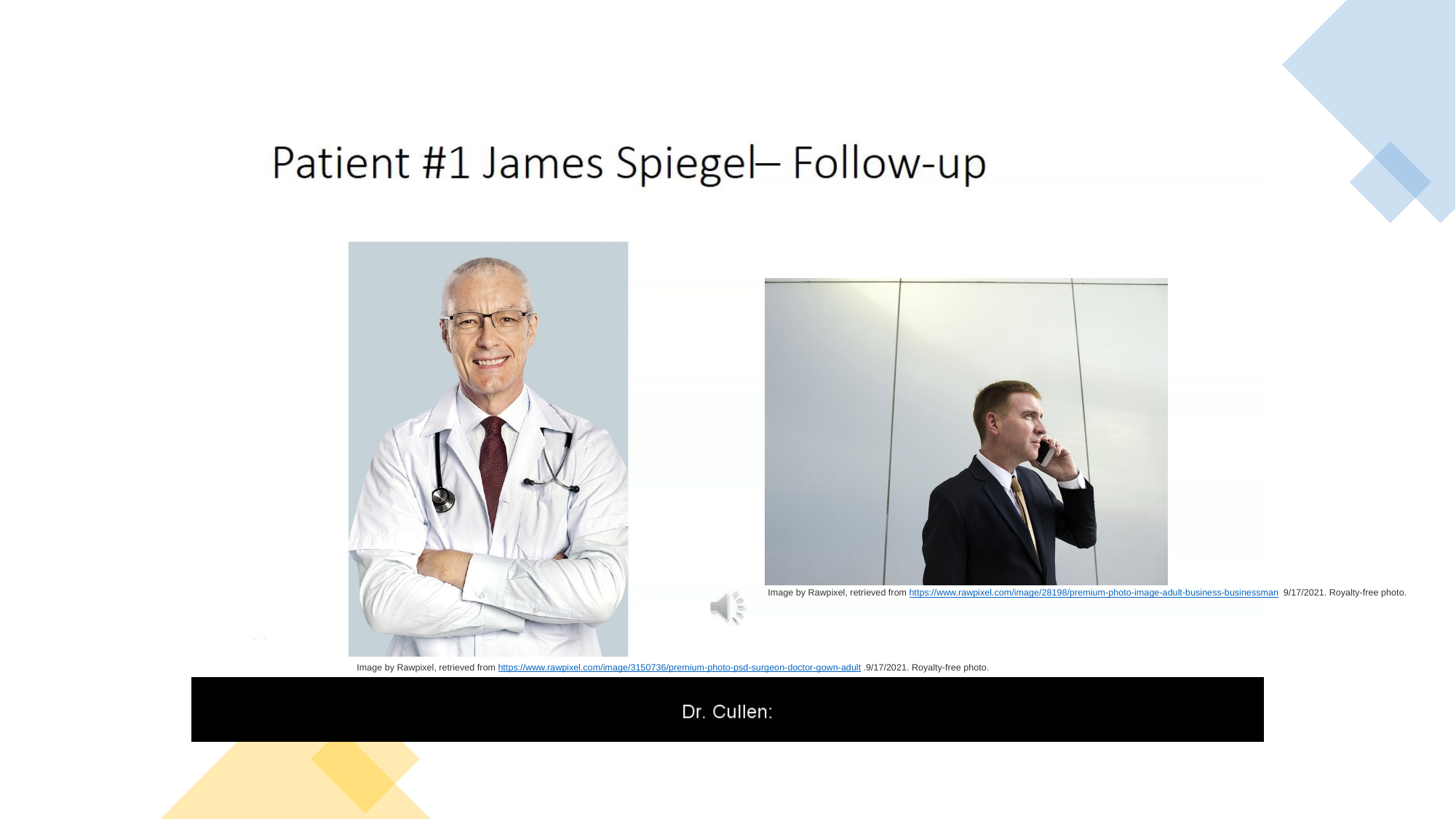

Image by Rawpixel, retrieved from https://www.rawpixel.com/image/28198/premium-photo-image-adult-business-businessman 9/17/2021. Royalty-free photo.
Image by Rawpixel, retrieved from https://www.rawpixel.com/image/3150736/premium-photo-psd-surgeon-doctor-gown-adult .9/17/2021. Royalty-free photo.

## Slide 36
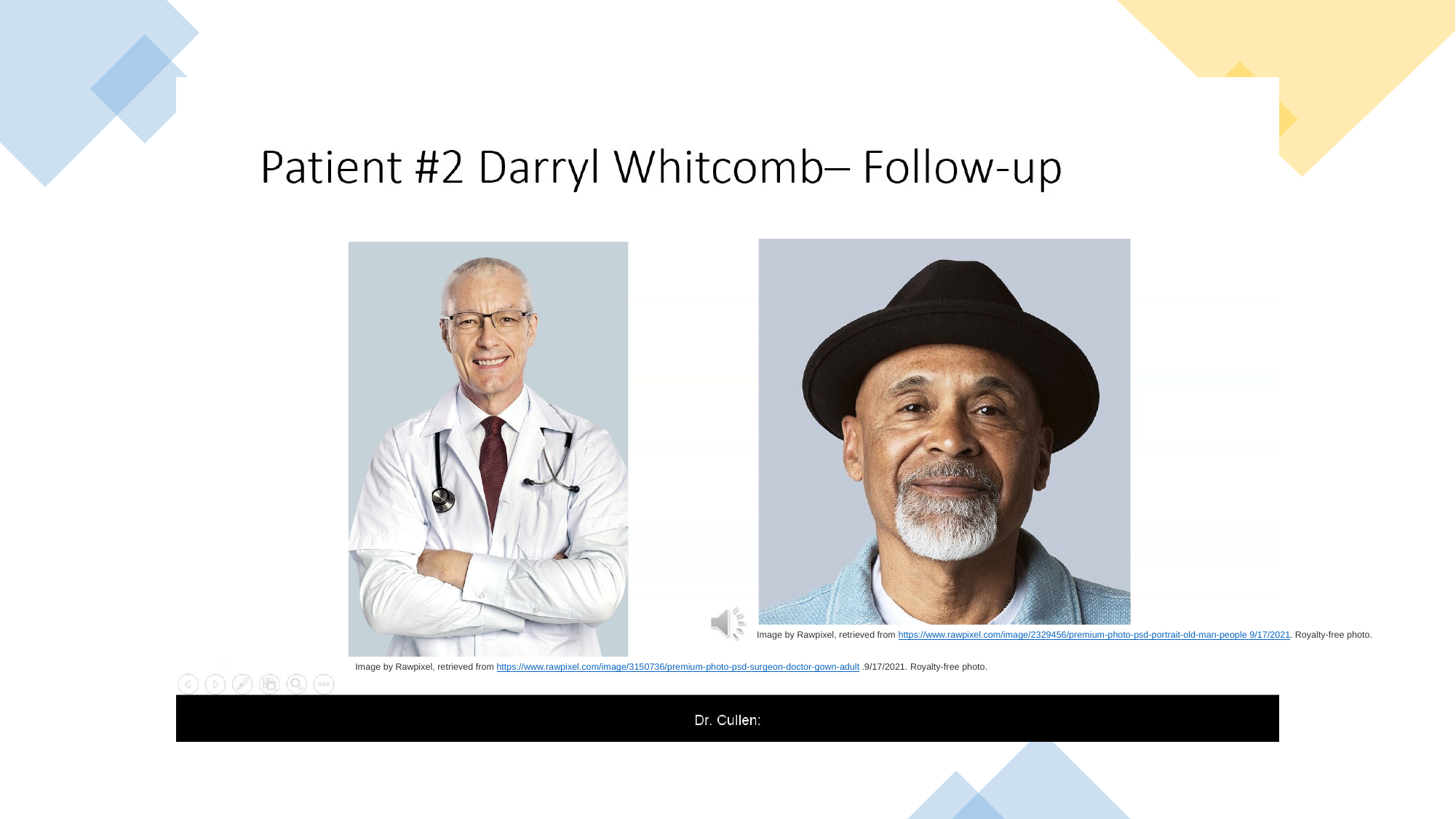

Image by Rawpixel, retrieved from https://www.rawpixel.com/image/2329456/premium-photo-psd-portrait-old-man-people 9/17/2021. Royalty-free photo.
Image by Rawpixel, retrieved from https://www.rawpixel.com/image/3150736/premium-photo-psd-surgeon-doctor-gown-adult .9/17/2021. Royalty-free photo.

## Slide 37
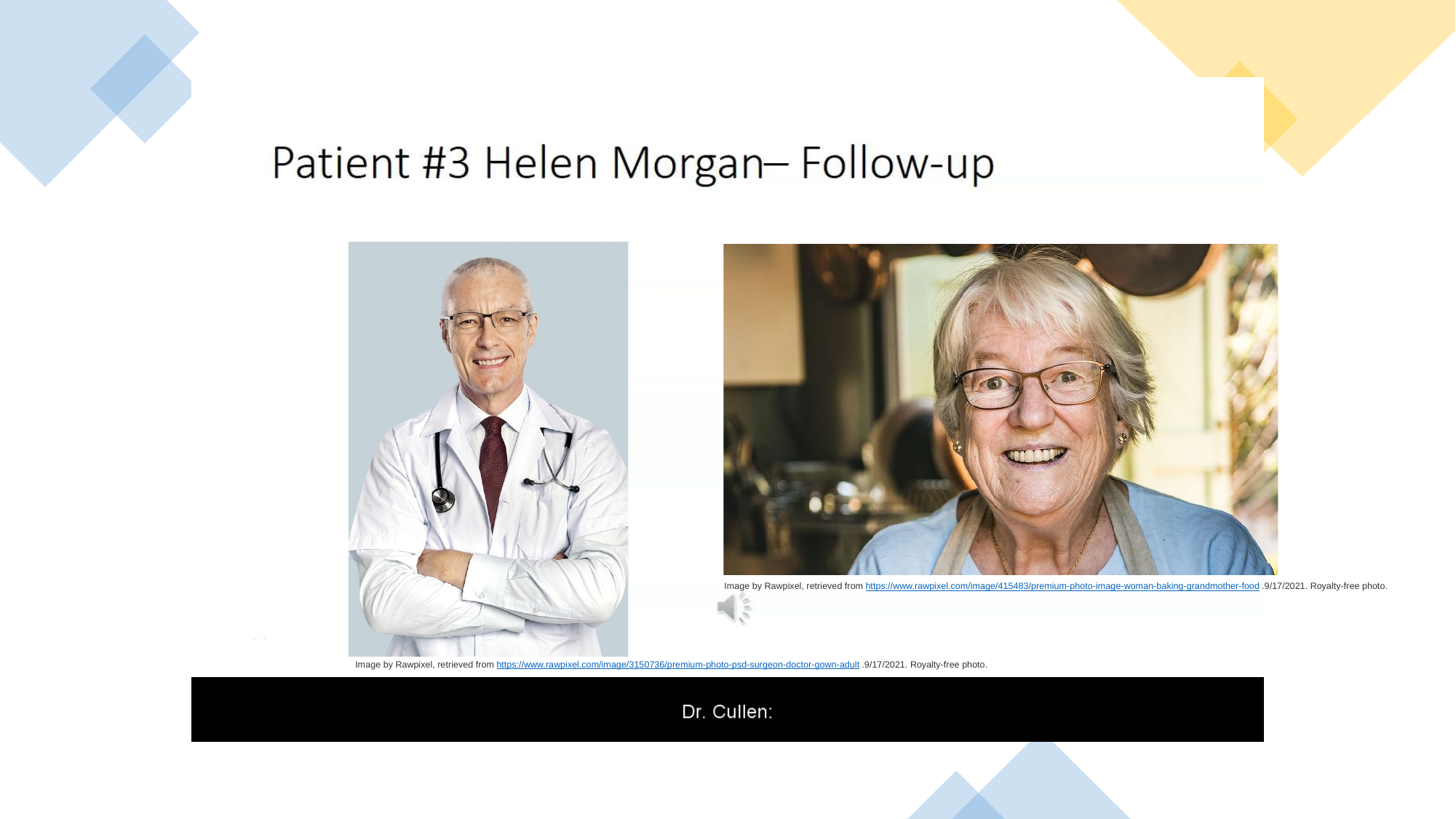

Image by Rawpixel, retrieved from https://www.rawpixel.com/image/415483/premium-photo-image-woman-baking-grandmother-food .9/17/2021. Royalty-free photo.
Image by Rawpixel, retrieved from https://www.rawpixel.com/image/3150736/premium-photo-psd-surgeon-doctor-gown-adult .9/17/2021. Royalty-free photo.

## Slide 38
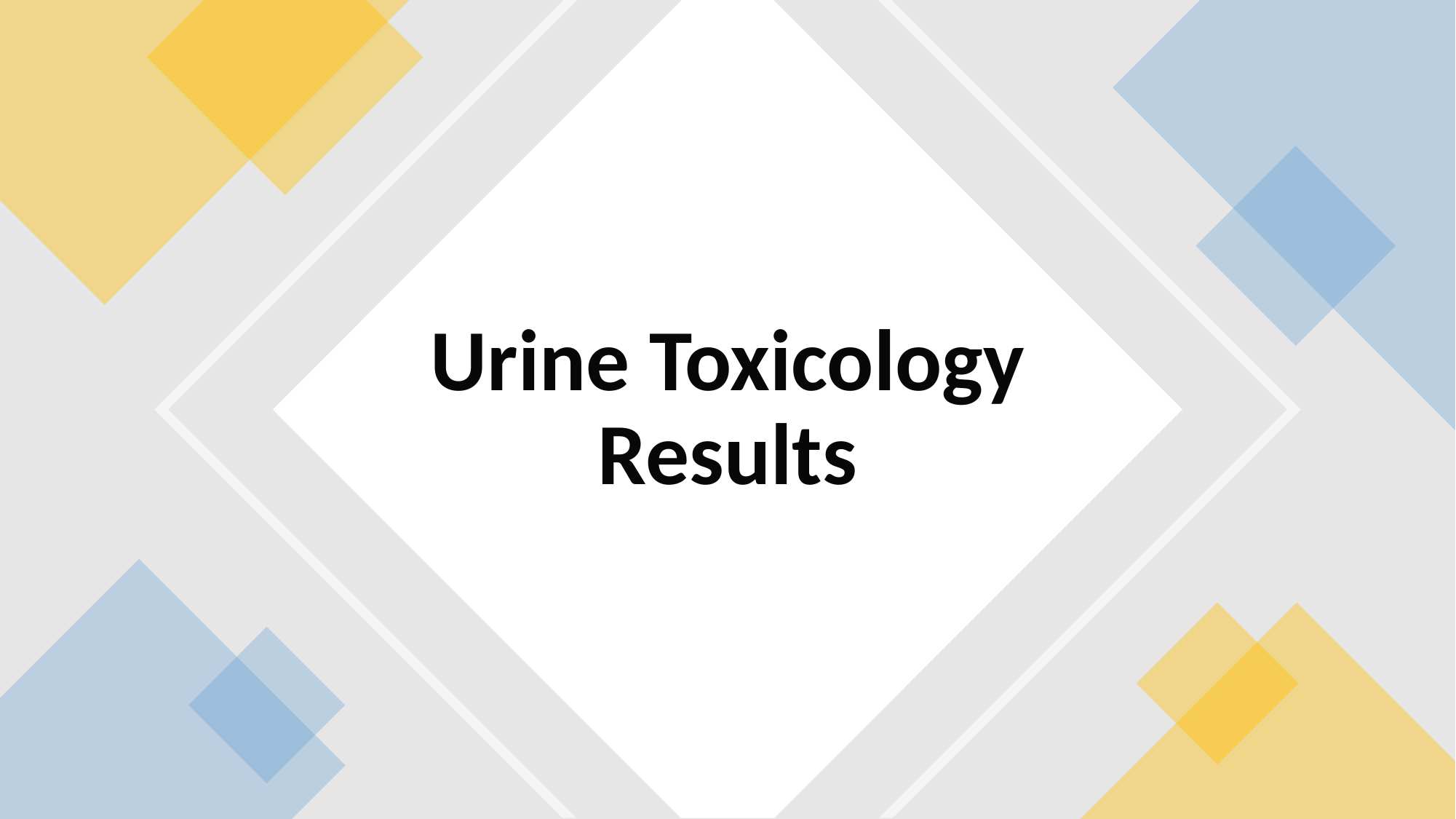

# Urine Toxicology Results

## Slide 39
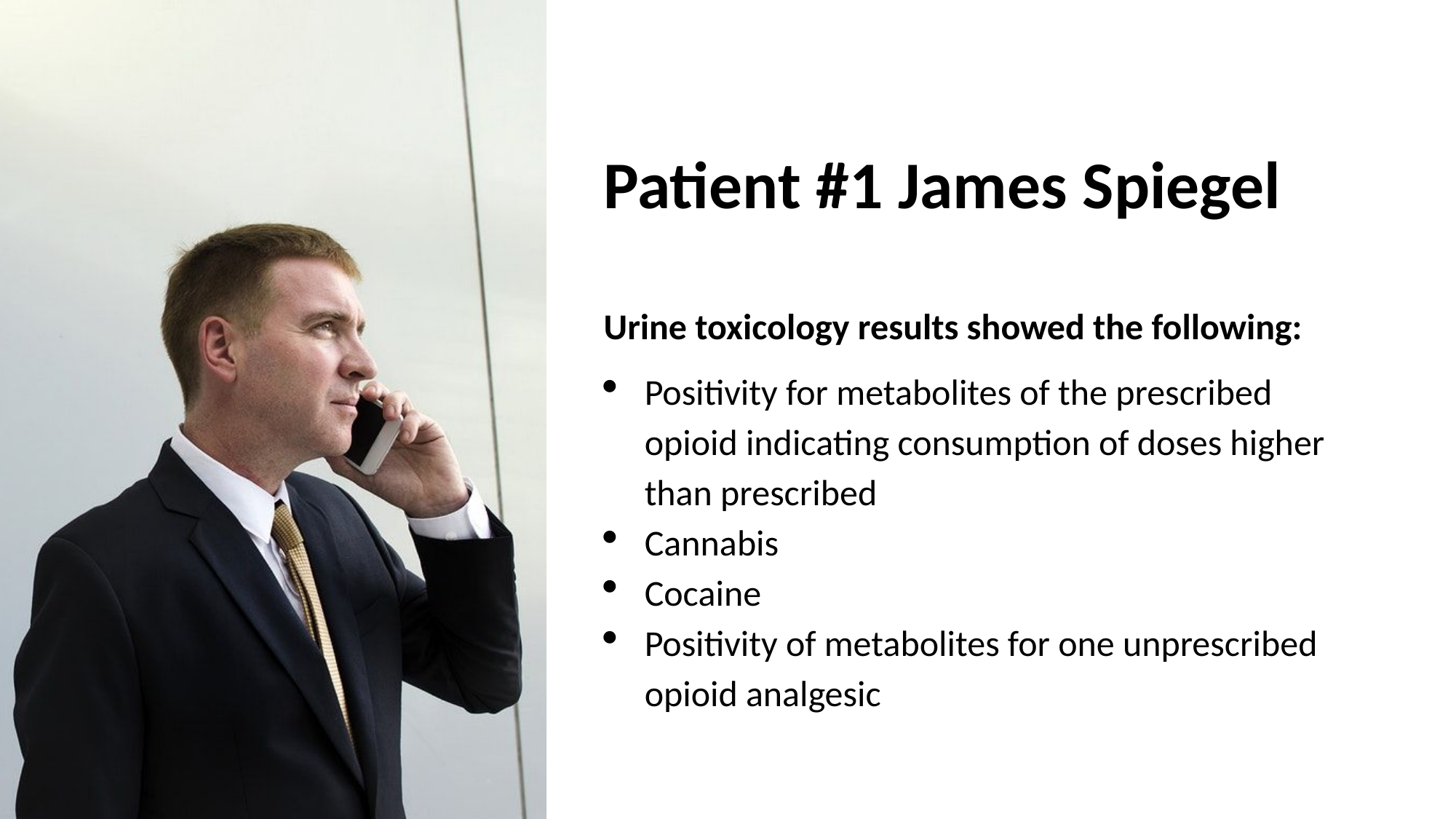

# Patient #1 James Spiegel
Urine toxicology results showed the following:
Positivity for metabolites of the prescribed opioid indicating consumption of doses higher than prescribed
Cannabis
Cocaine
Positivity of metabolites for one unprescribed opioid analgesic

## Slide 40
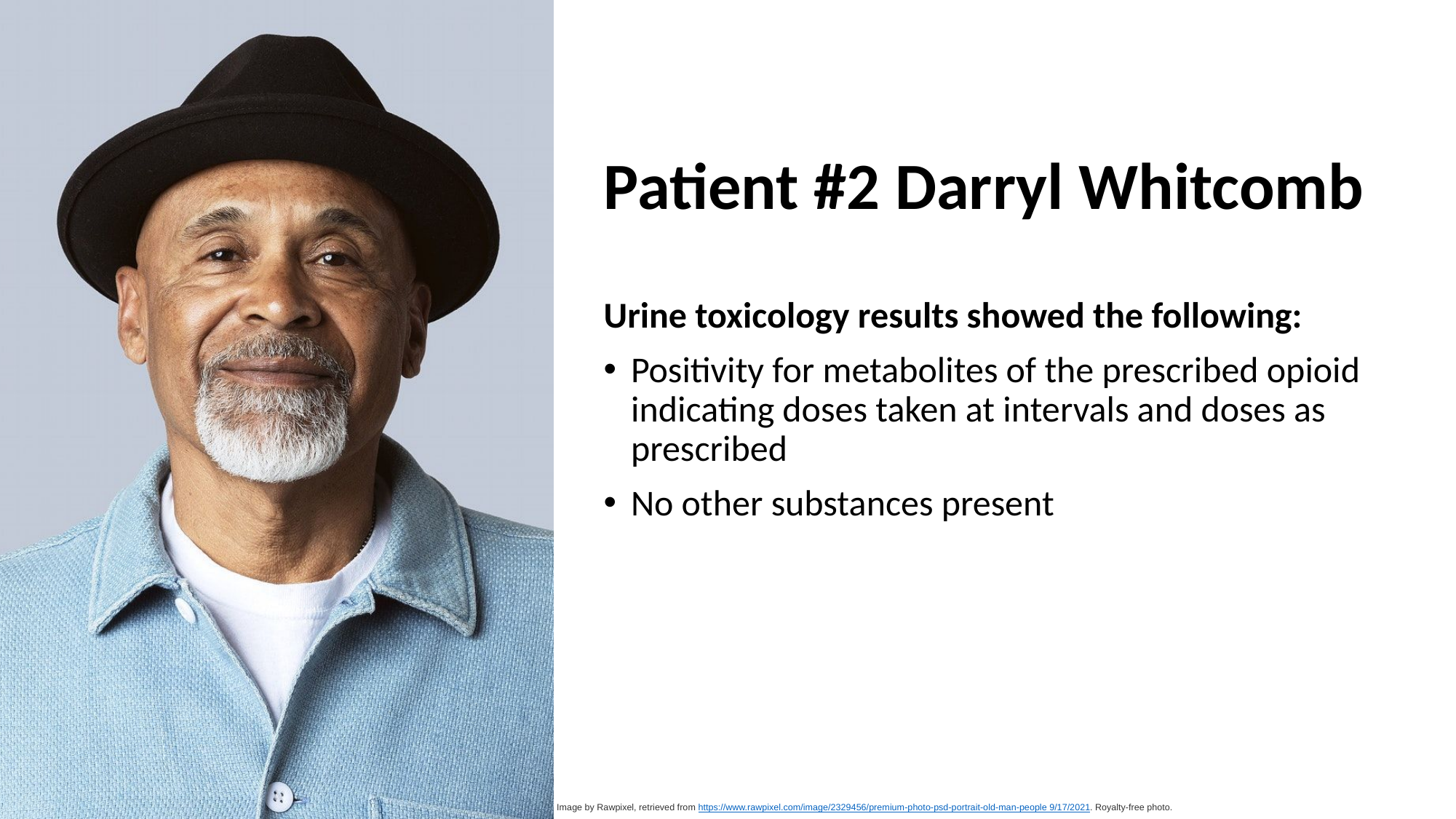

# Patient #2 Darryl Whitcomb
Urine toxicology results showed the following:
Positivity for metabolites of the prescribed opioid indicating doses taken at intervals and doses as prescribed
No other substances present
Image by Rawpixel, retrieved from https://www.rawpixel.com/image/2329456/premium-photo-psd-portrait-old-man-people 9/17/2021. Royalty-free photo.

## Slide 41
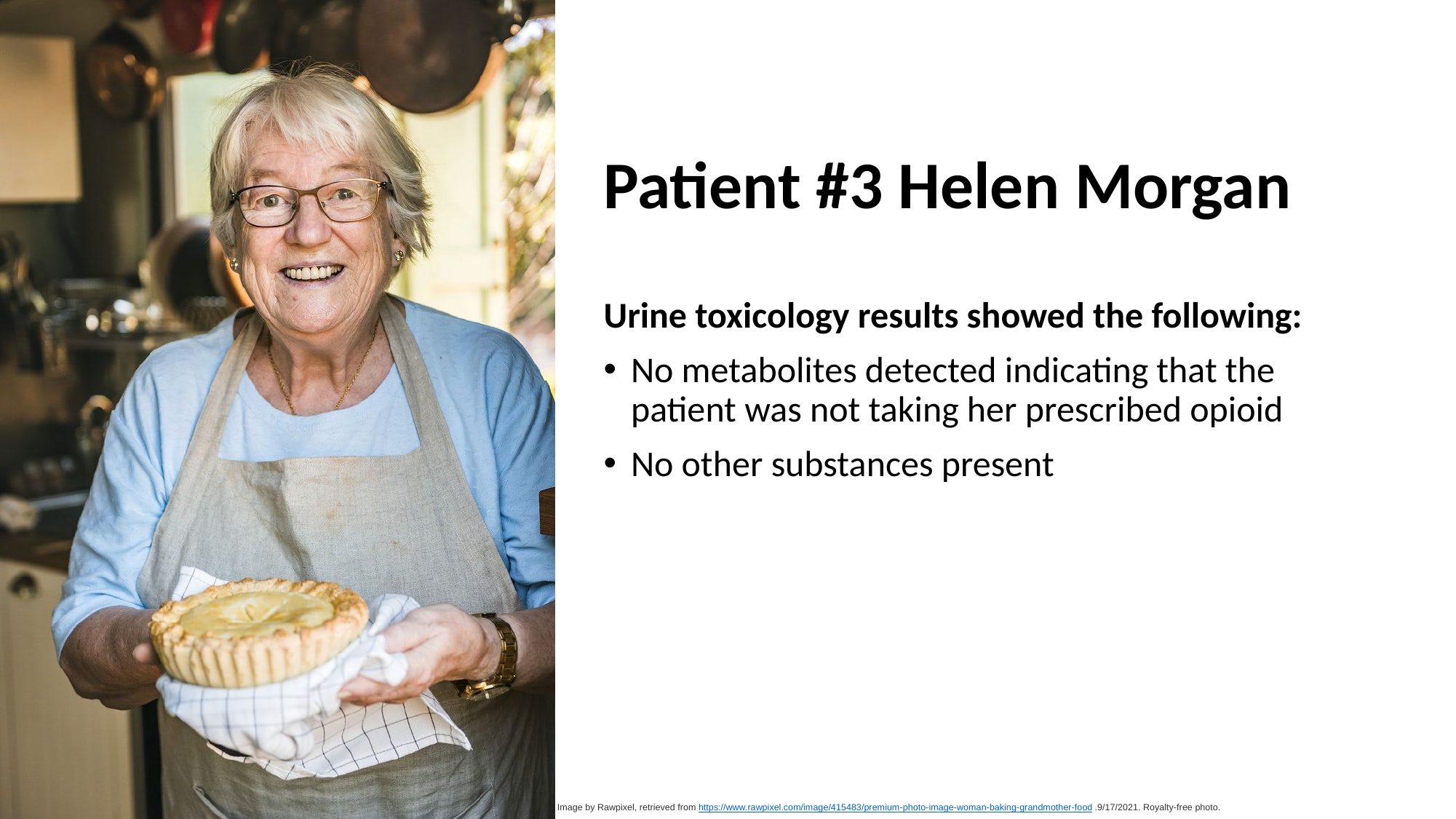

# Patient #3 Helen Morgan
Urine toxicology results showed the following:
No metabolites detected indicating that the patient was not taking her prescribed opioid
No other substances present
Image by Rawpixel, retrieved from https://www.rawpixel.com/image/415483/premium-photo-image-woman-baking-grandmother-food .9/17/2021. Royalty-free photo.

## Slide 42
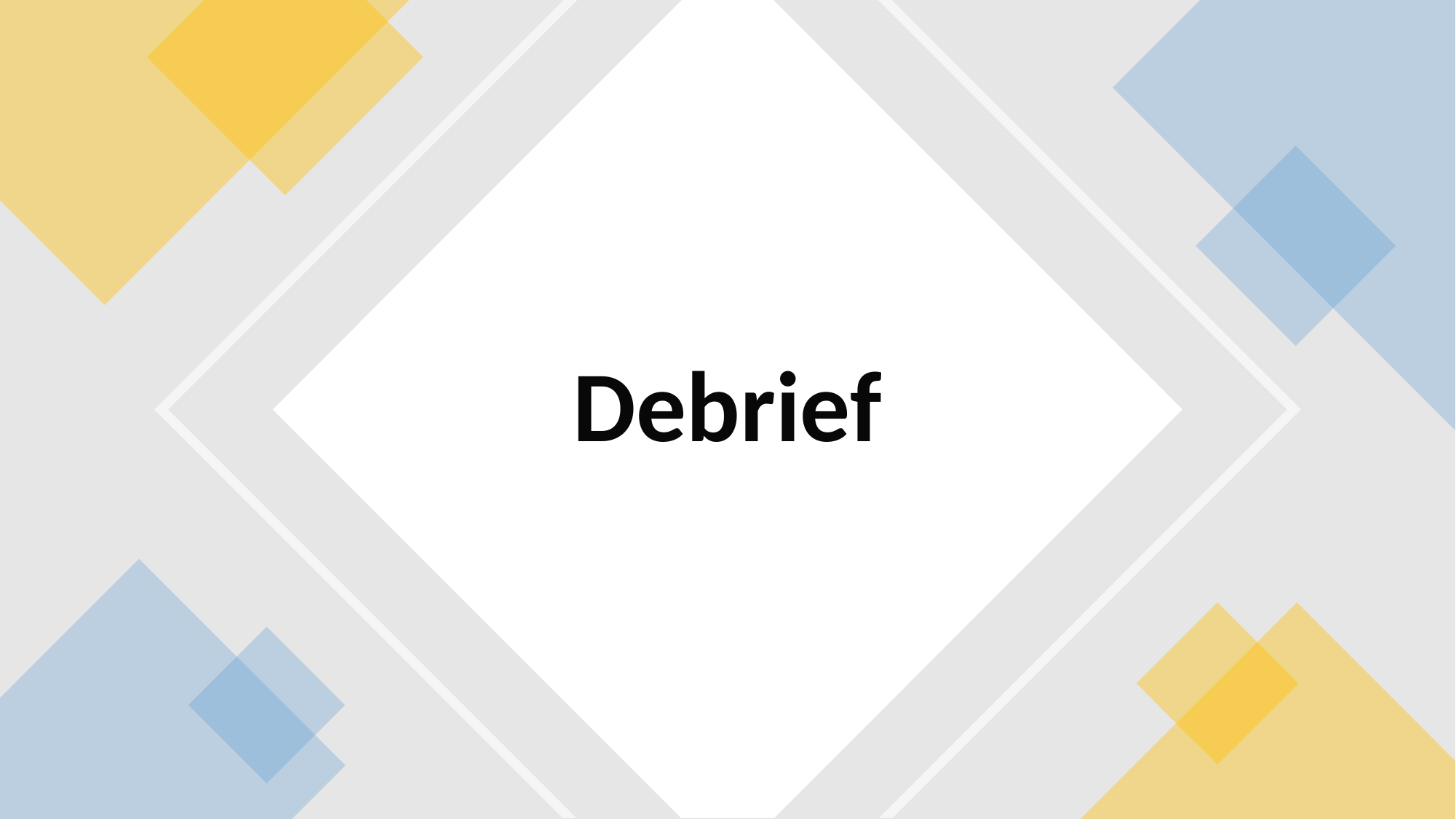

# Debrief

## Slide 43
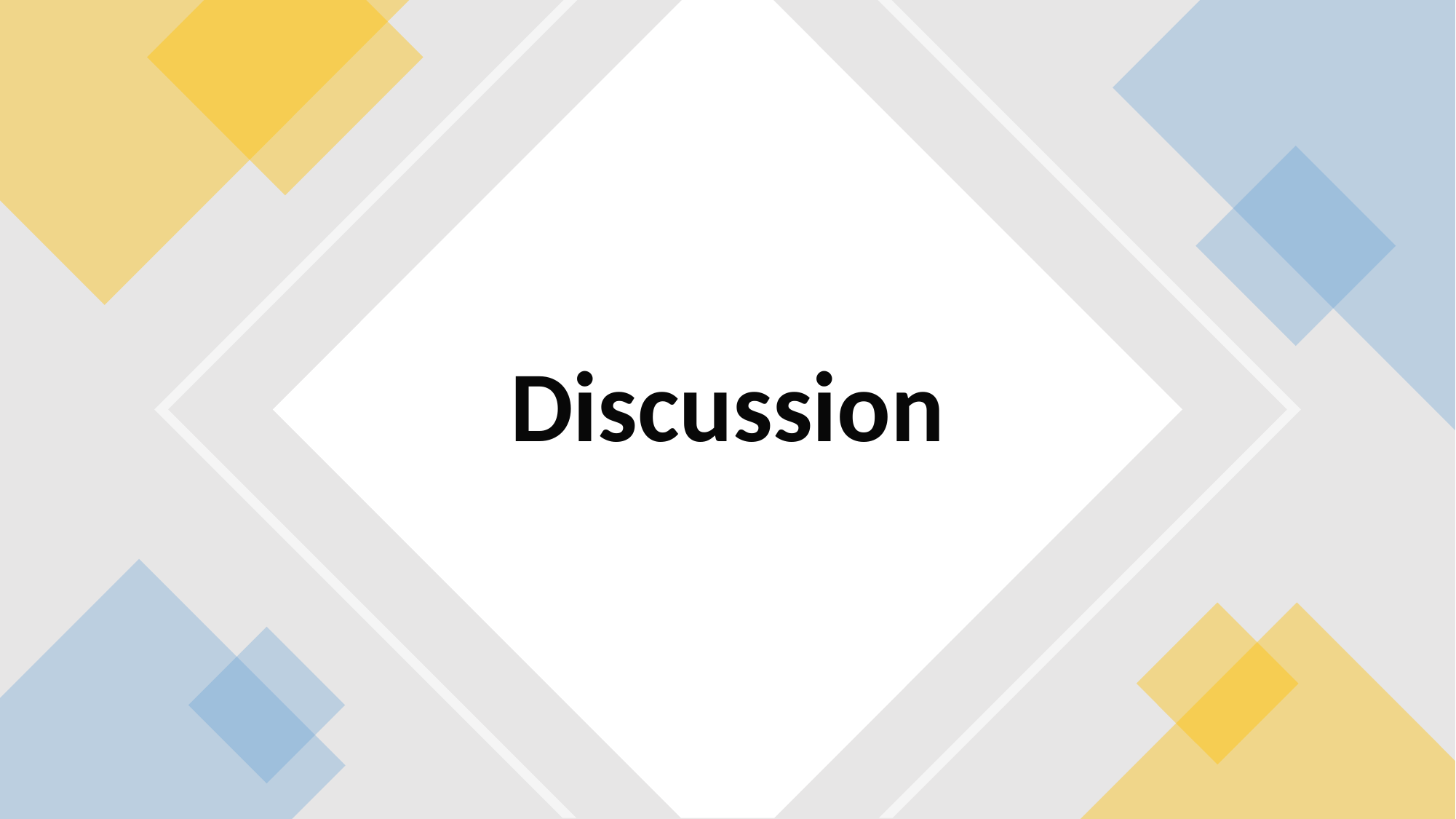

# Discussion

## Slide 44
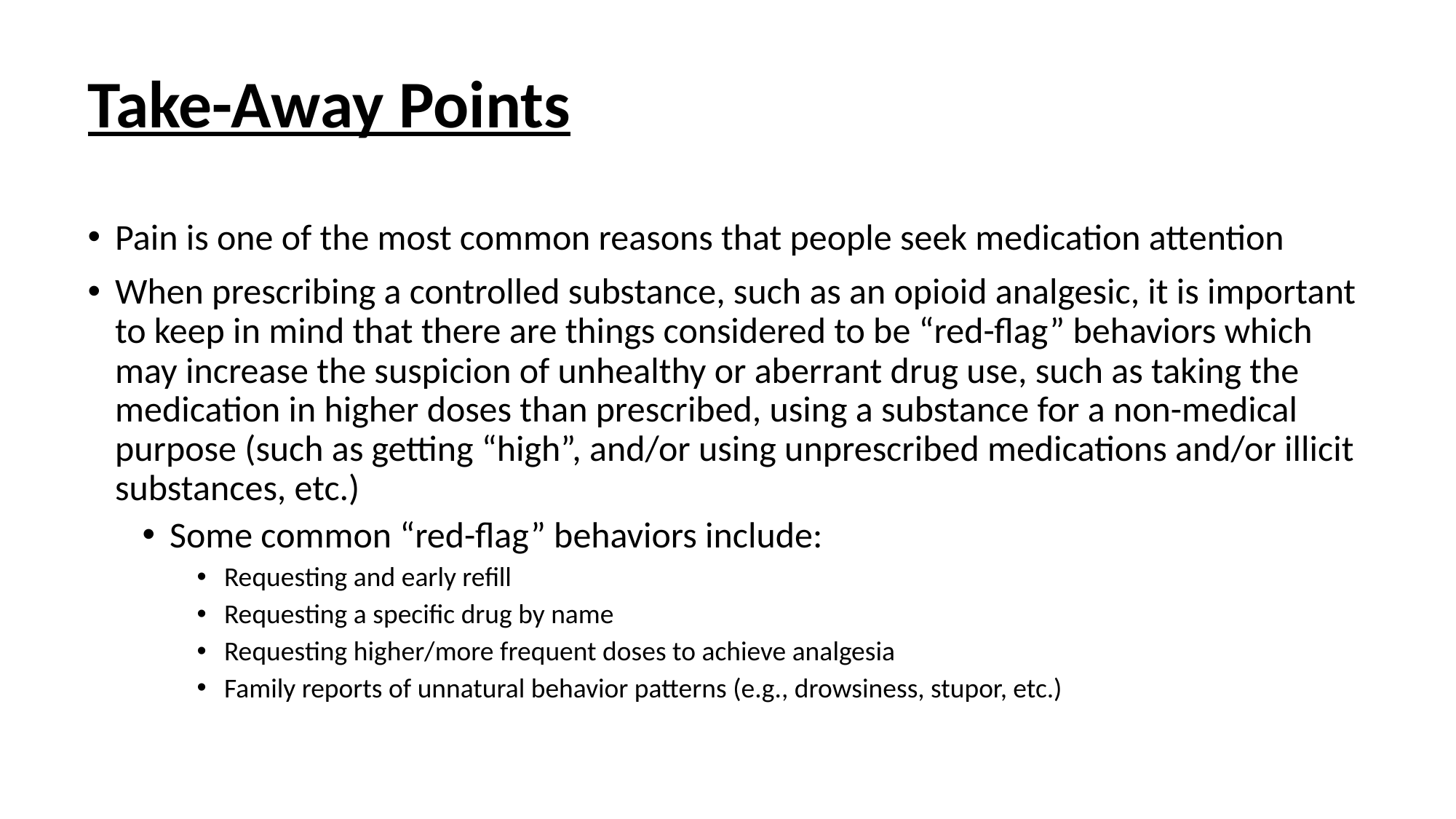

# Take-Away Points
Pain is one of the most common reasons that people seek medication attention
When prescribing a controlled substance, such as an opioid analgesic, it is important to keep in mind that there are things considered to be “red-flag” behaviors which may increase the suspicion of unhealthy or aberrant drug use, such as taking the medication in higher doses than prescribed, using a substance for a non-medical purpose (such as getting “high”, and/or using unprescribed medications and/or illicit substances, etc.)
Some common “red-flag” behaviors include:
Requesting and early refill
Requesting a specific drug by name
Requesting higher/more frequent doses to achieve analgesia
Family reports of unnatural behavior patterns (e.g., drowsiness, stupor, etc.)

## Slide 45
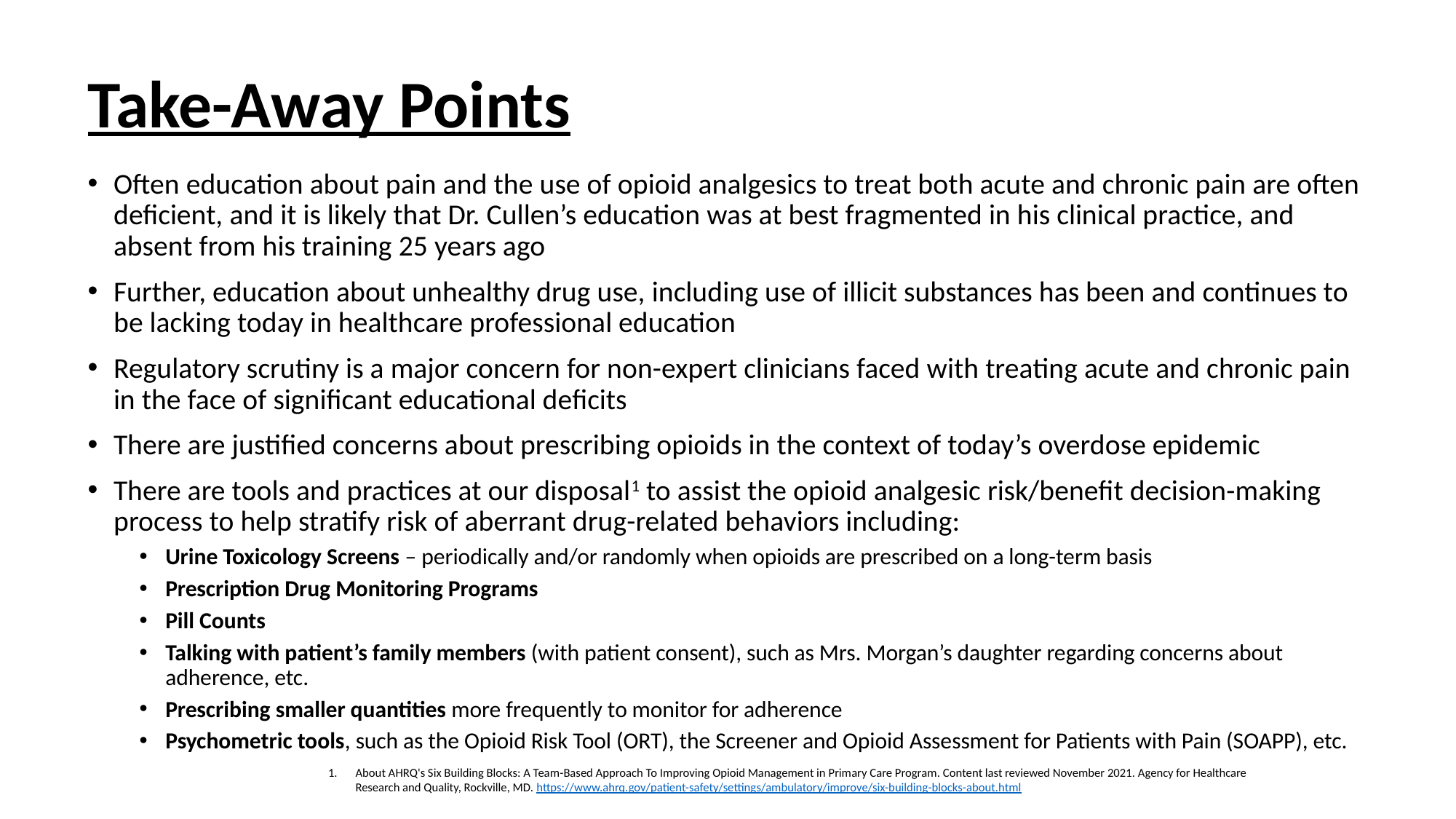

# Take-Away Points
Often education about pain and the use of opioid analgesics to treat both acute and chronic pain are often deficient, and it is likely that Dr. Cullen’s education was at best fragmented in his clinical practice, and absent from his training 25 years ago
Further, education about unhealthy drug use, including use of illicit substances has been and continues to be lacking today in healthcare professional education
Regulatory scrutiny is a major concern for non-expert clinicians faced with treating acute and chronic pain in the face of significant educational deficits
There are justified concerns about prescribing opioids in the context of today’s overdose epidemic
There are tools and practices at our disposal1 to assist the opioid analgesic risk/benefit decision-making process to help stratify risk of aberrant drug-related behaviors including:
Urine Toxicology Screens – periodically and/or randomly when opioids are prescribed on a long-term basis
Prescription Drug Monitoring Programs
Pill Counts
Talking with patient’s family members (with patient consent), such as Mrs. Morgan’s daughter regarding concerns about adherence, etc.
Prescribing smaller quantities more frequently to monitor for adherence
Psychometric tools, such as the Opioid Risk Tool (ORT), the Screener and Opioid Assessment for Patients with Pain (SOAPP), etc.
About AHRQ's Six Building Blocks: A Team-Based Approach To Improving Opioid Management in Primary Care Program. Content last reviewed November 2021. Agency for Healthcare Research and Quality, Rockville, MD. https://www.ahrq.gov/patient-safety/settings/ambulatory/improve/six-building-blocks-about.html

## Slide 46
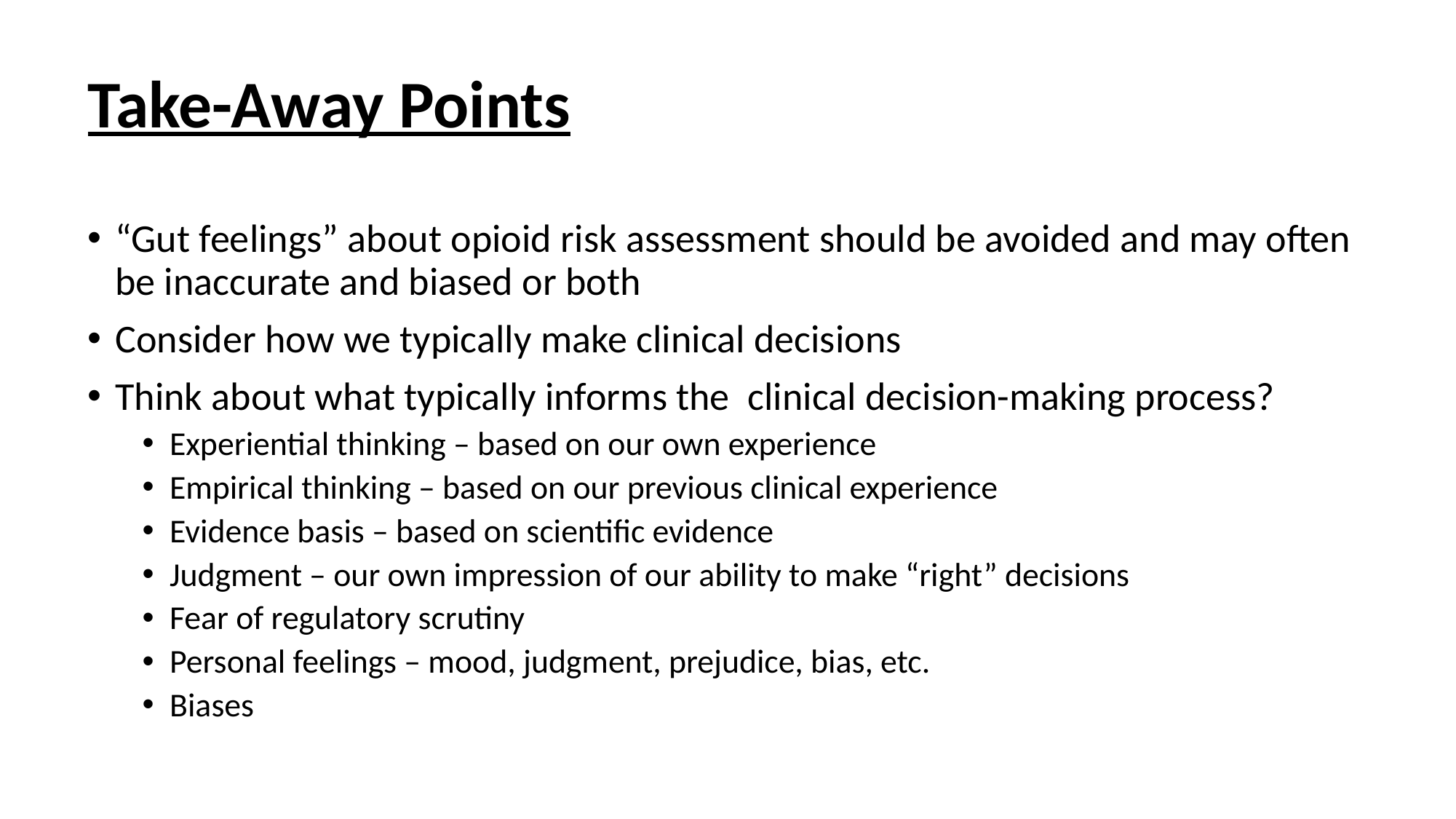

# Take-Away Points
“Gut feelings” about opioid risk assessment should be avoided and may often be inaccurate and biased or both
Consider how we typically make clinical decisions
Think about what typically informs the clinical decision-making process?
Experiential thinking – based on our own experience
Empirical thinking – based on our previous clinical experience
Evidence basis – based on scientific evidence
Judgment – our own impression of our ability to make “right” decisions
Fear of regulatory scrutiny
Personal feelings – mood, judgment, prejudice, bias, etc.
Biases

## Slide 47
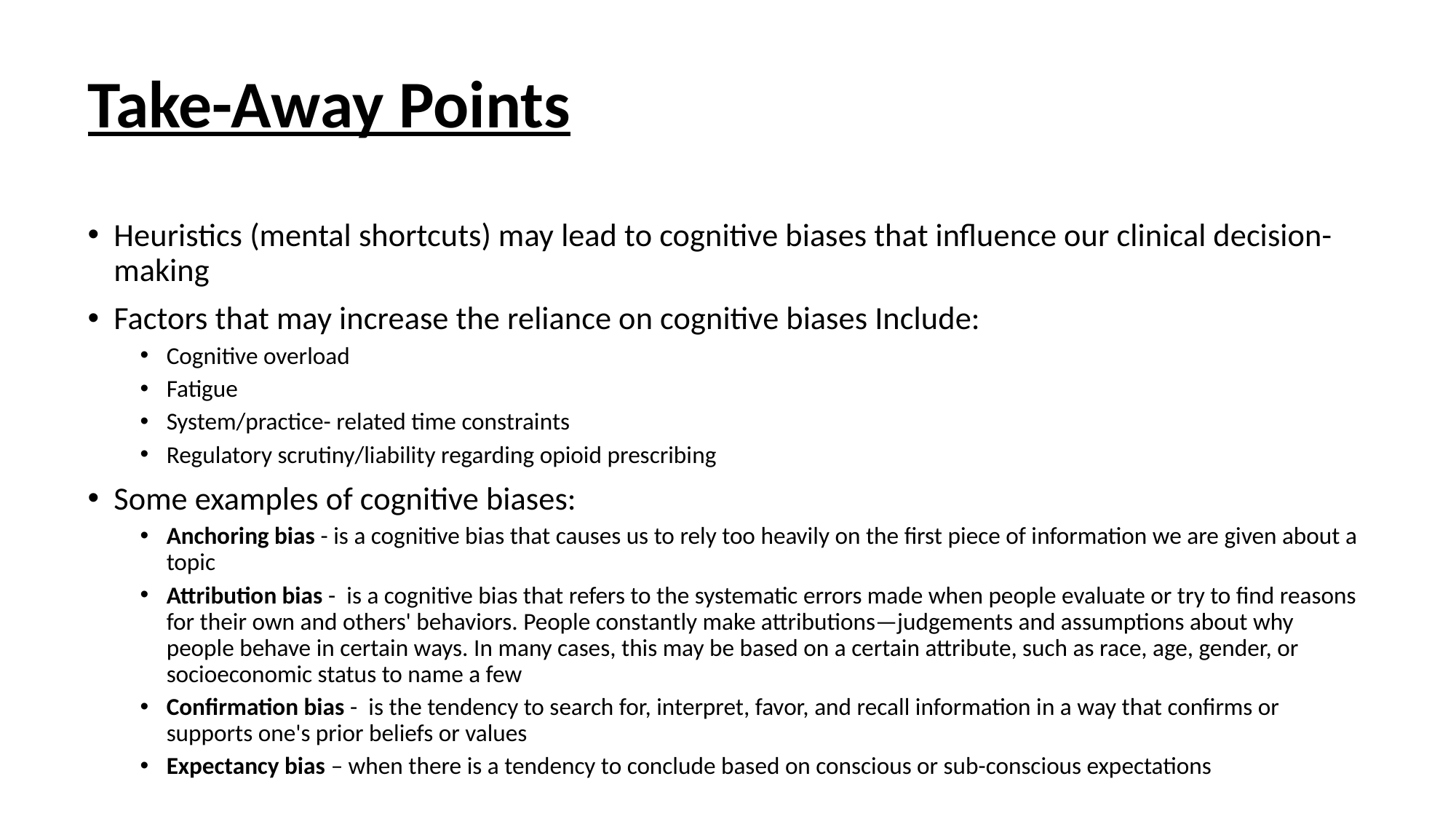

# Take-Away Points
Heuristics (mental shortcuts) may lead to cognitive biases that influence our clinical decision-making
Factors that may increase the reliance on cognitive biases Include:
Cognitive overload
Fatigue
System/practice- related time constraints
Regulatory scrutiny/liability regarding opioid prescribing
Some examples of cognitive biases:
Anchoring bias - is a cognitive bias that causes us to rely too heavily on the first piece of information we are given about a topic
Attribution bias - is a cognitive bias that refers to the systematic errors made when people evaluate or try to find reasons for their own and others' behaviors. People constantly make attributions—judgements and assumptions about why people behave in certain ways. In many cases, this may be based on a certain attribute, such as race, age, gender, or socioeconomic status to name a few
Confirmation bias - is the tendency to search for, interpret, favor, and recall information in a way that confirms or supports one's prior beliefs or values
Expectancy bias – when there is a tendency to conclude based on conscious or sub-conscious expectations

## Slide 48
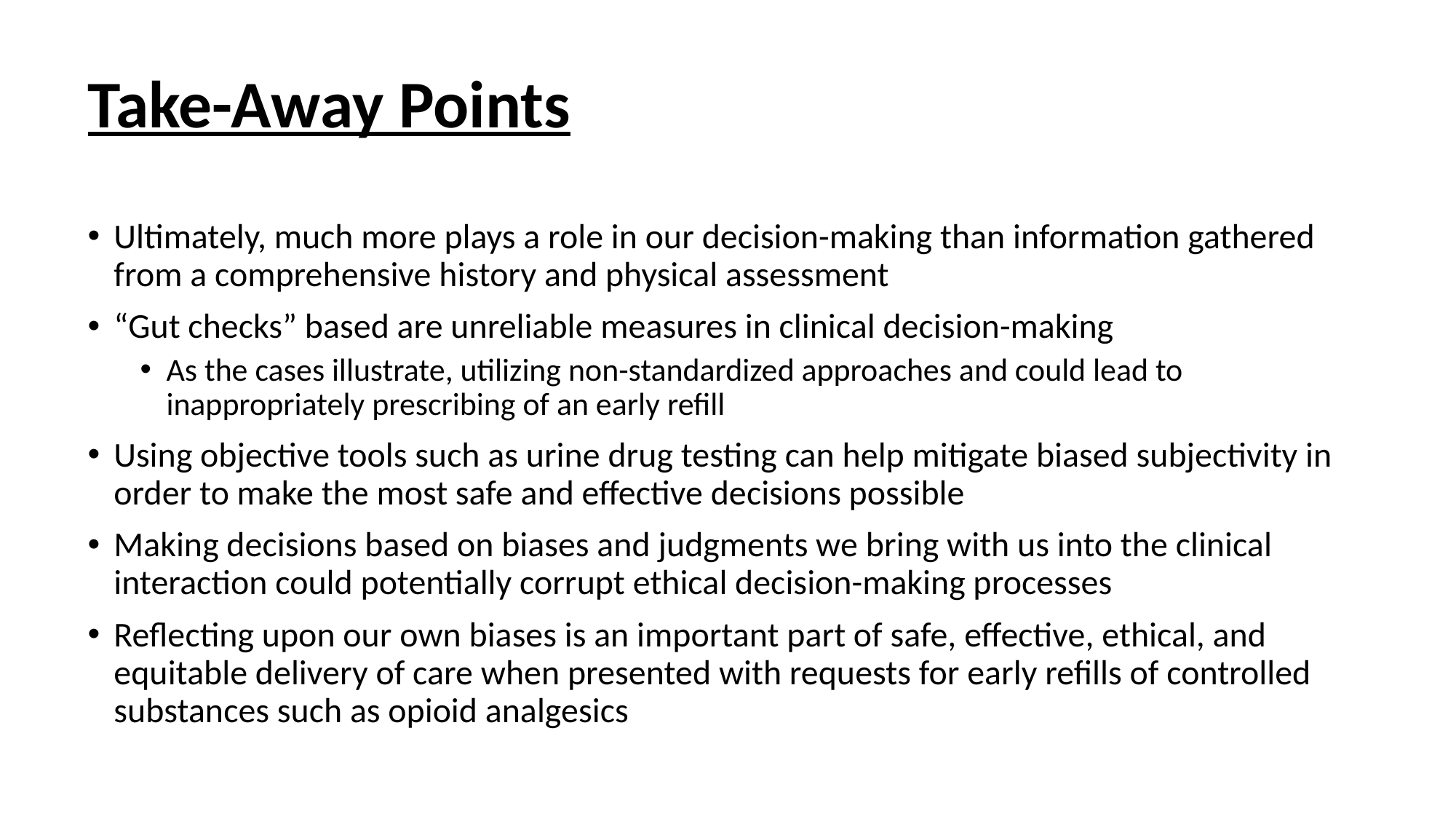

# Take-Away Points
Ultimately, much more plays a role in our decision-making than information gathered from a comprehensive history and physical assessment
“Gut checks” based are unreliable measures in clinical decision-making
As the cases illustrate, utilizing non-standardized approaches and could lead to inappropriately prescribing of an early refill
Using objective tools such as urine drug testing can help mitigate biased subjectivity in order to make the most safe and effective decisions possible
Making decisions based on biases and judgments we bring with us into the clinical interaction could potentially corrupt ethical decision-making processes
Reflecting upon our own biases is an important part of safe, effective, ethical, and equitable delivery of care when presented with requests for early refills of controlled substances such as opioid analgesics

## Slide 49
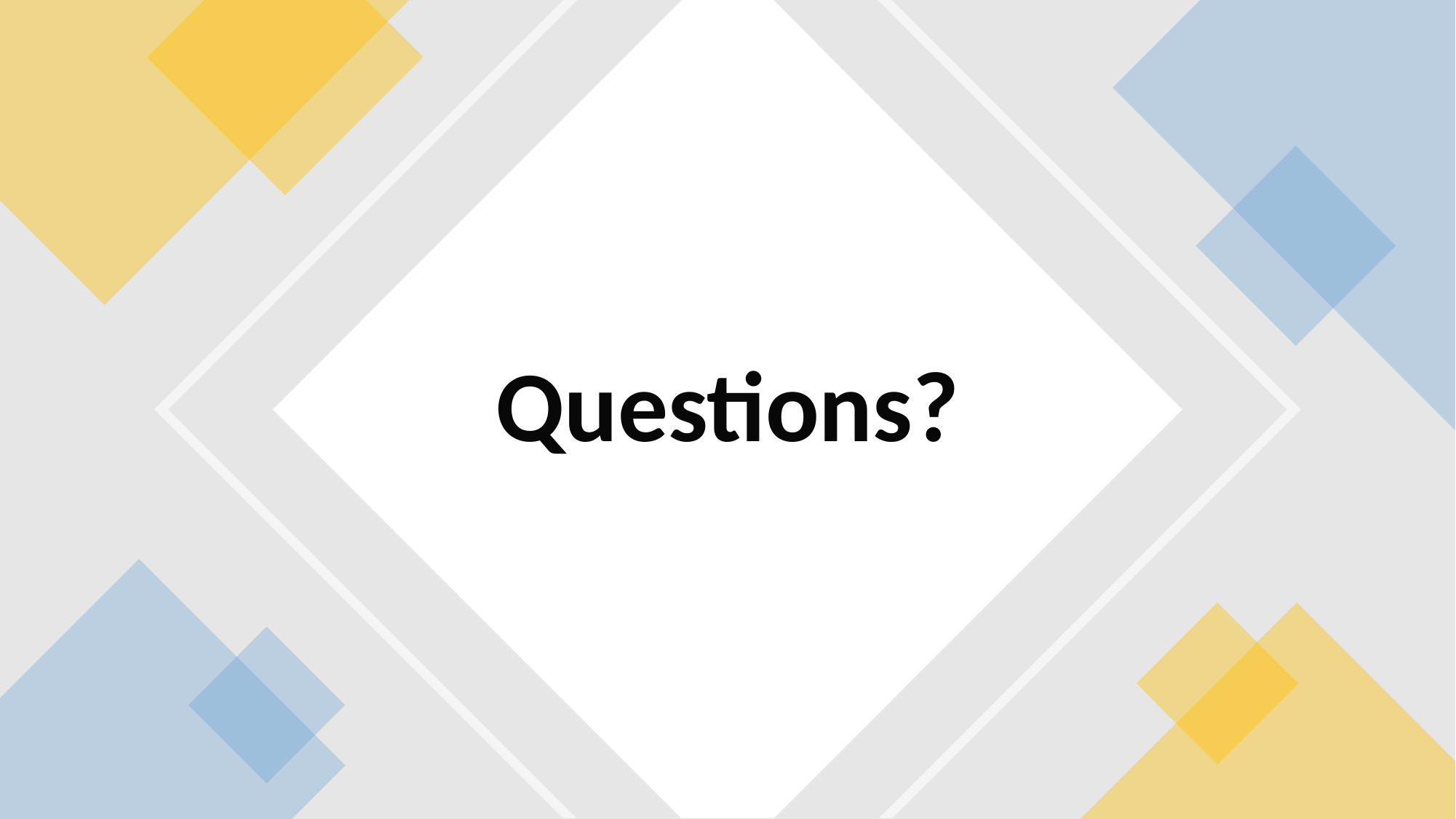

# Questions?
