# Supplementary material for: Early Refill of an Opioid Medication: Recognizing Personal Biases Through Clinical Vignettes and OSCEs
Source: MedEdPORTAL. 2022 Apr 7;18:11234. doi: 10.15766/mep_2374-8265.11234 (PMC8986891; doi:10.15766/mep_2374-8265.11234)
Supplement: Supplementary file 1 — MS 1 Clinical Vignettes & Follow-Up.pptxMS 1 Debrief.pptxSP James Spiegel - Case 1.docxSP Darryl Whitcomb - Case 2.docxSP Helen Morgan - Case 3.docxDoor Notes.docxLogistical Flow.docxFaculty Post-OSCE Debrief Discussion Guide.docxSP Encounter Checklist.docxSP Responses for Checklist Items.docxMS 3 Post-OSCE Survey.docx [file mep_2374-8265.11234-s001.zip › B. MS 1 Debrief.pptx]

## Slide 1
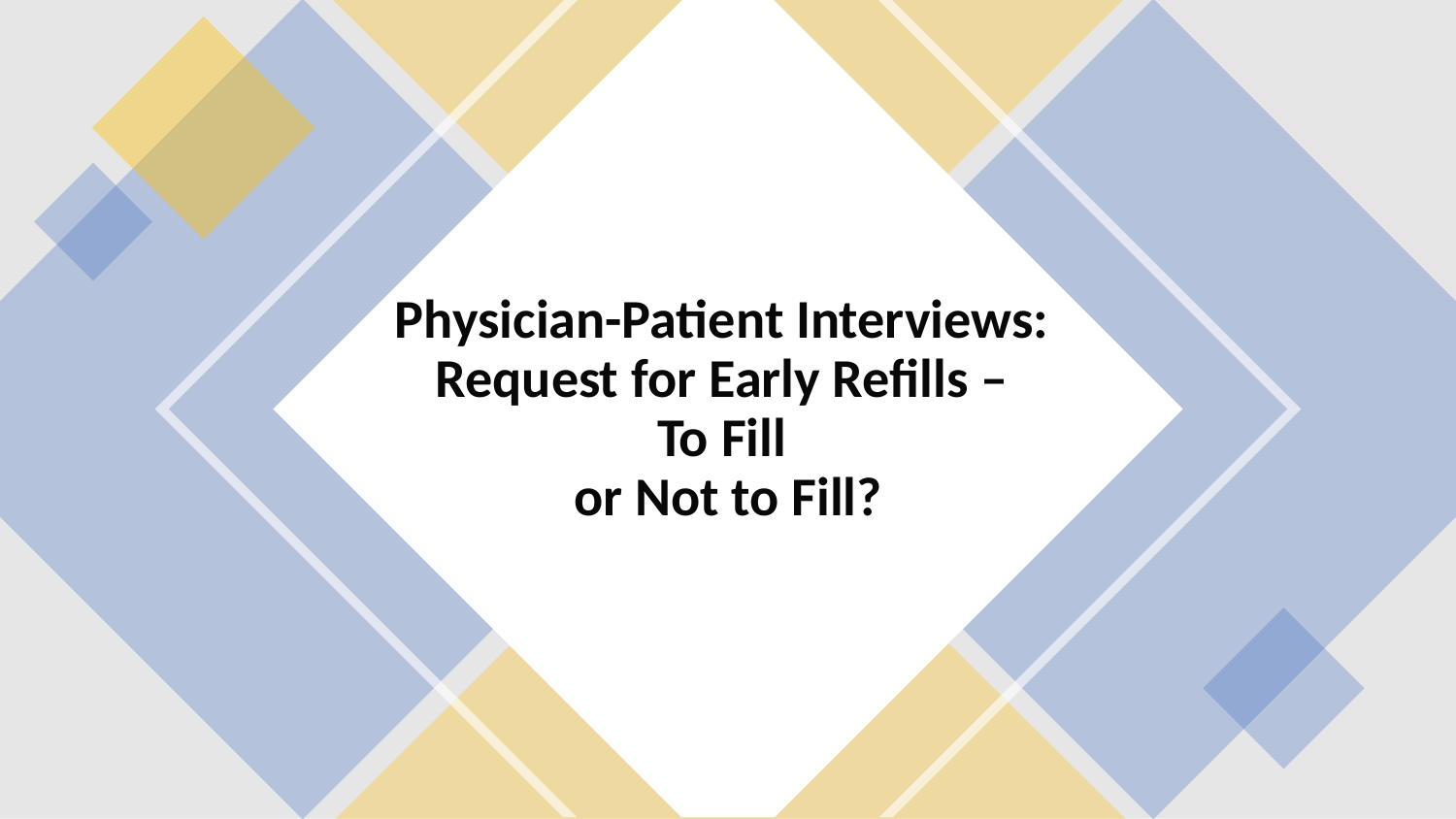

# Physician-Patient Interviews: Request for Early Refills – To Fill or Not to Fill?

## Slide 2
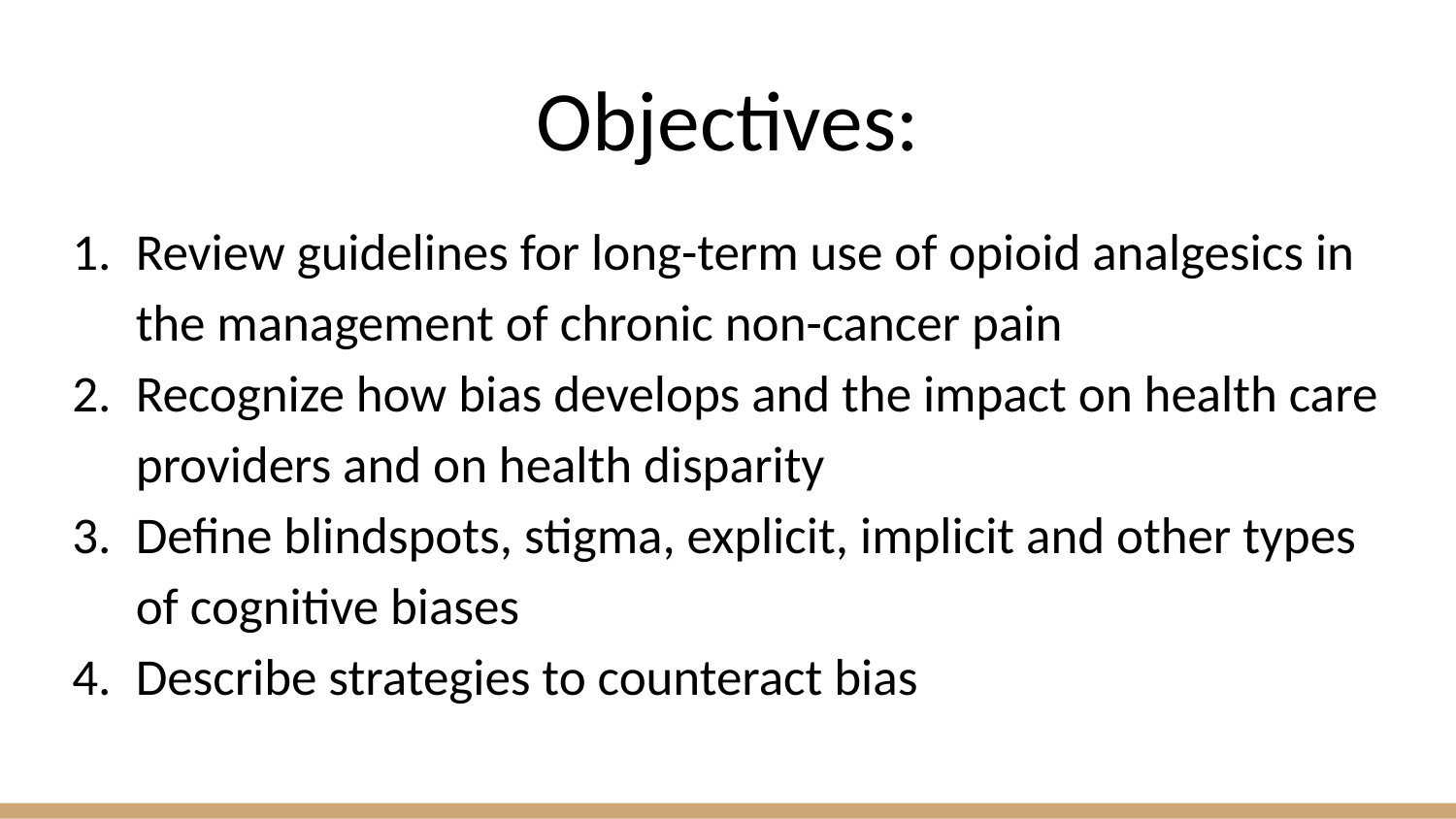

# Objectives:
Review guidelines for long-term use of opioid analgesics in the management of chronic non-cancer pain
Recognize how bias develops and the impact on health care providers and on health disparity
Define blindspots, stigma, explicit, implicit and other types of cognitive biases
Describe strategies to counteract bias

## Slide 3
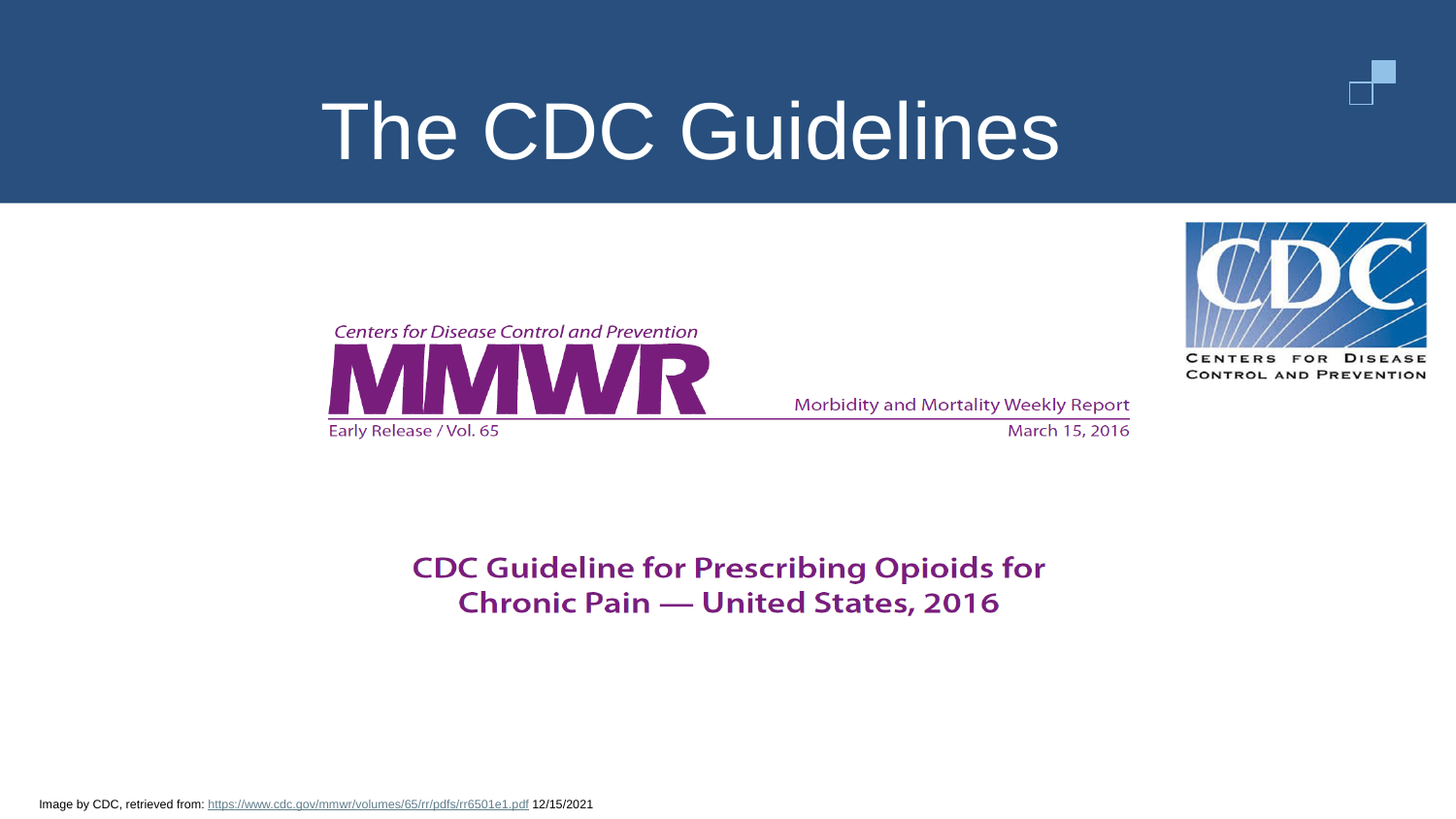

# The CDC Guidelines
Image by CDC, retrieved from: https://www.cdc.gov/mmwr/volumes/65/rr/pdfs/rr6501e1.pdf 12/15/2021

## Slide 4
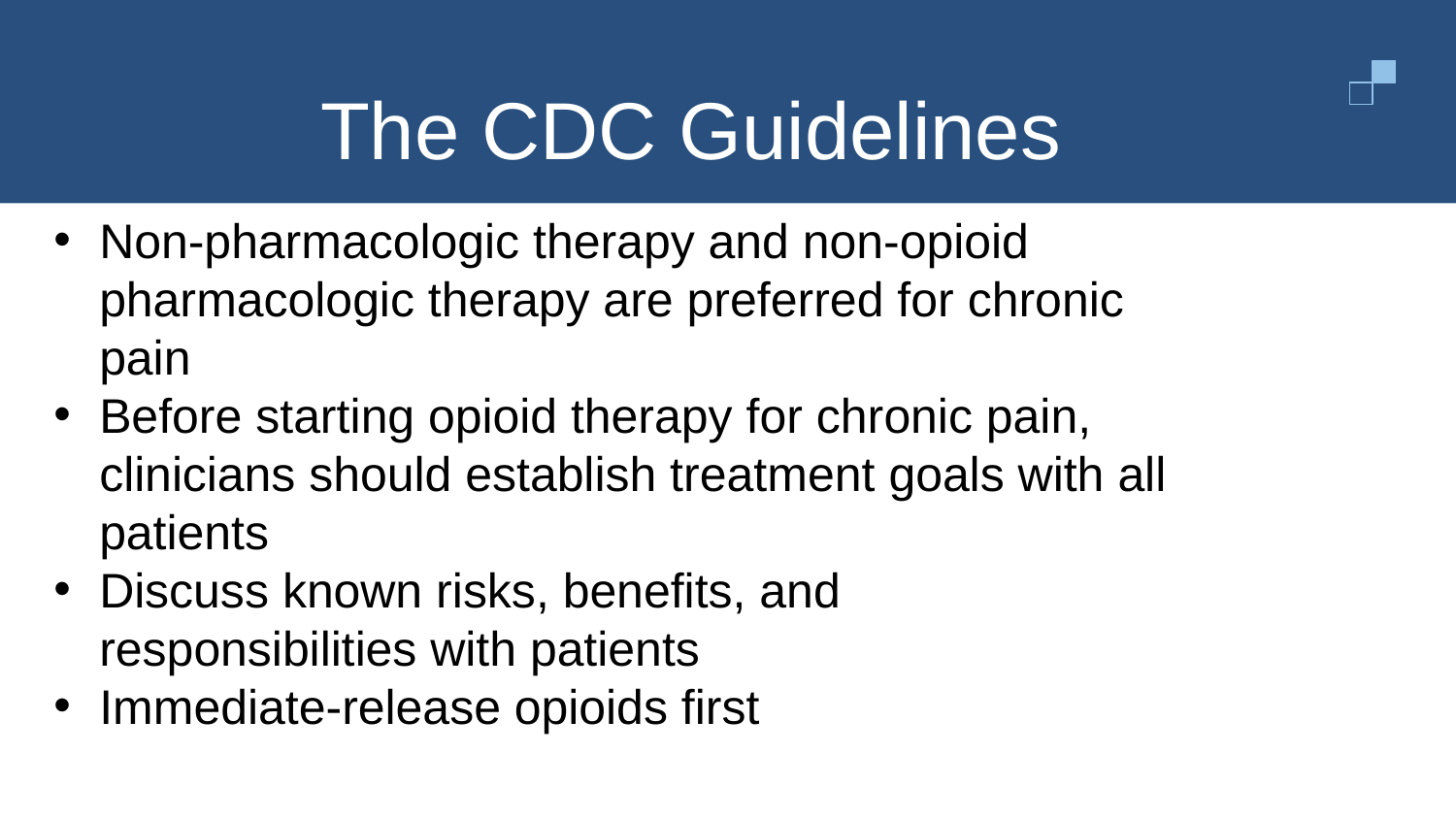

# The CDC Guidelines
Non-pharmacologic therapy and non-opioid pharmacologic therapy are preferred for chronic pain
Before starting opioid therapy for chronic pain, clinicians should establish treatment goals with all patients
Discuss known risks, benefits, and responsibilities with patients
Immediate-release opioids first

## Slide 5
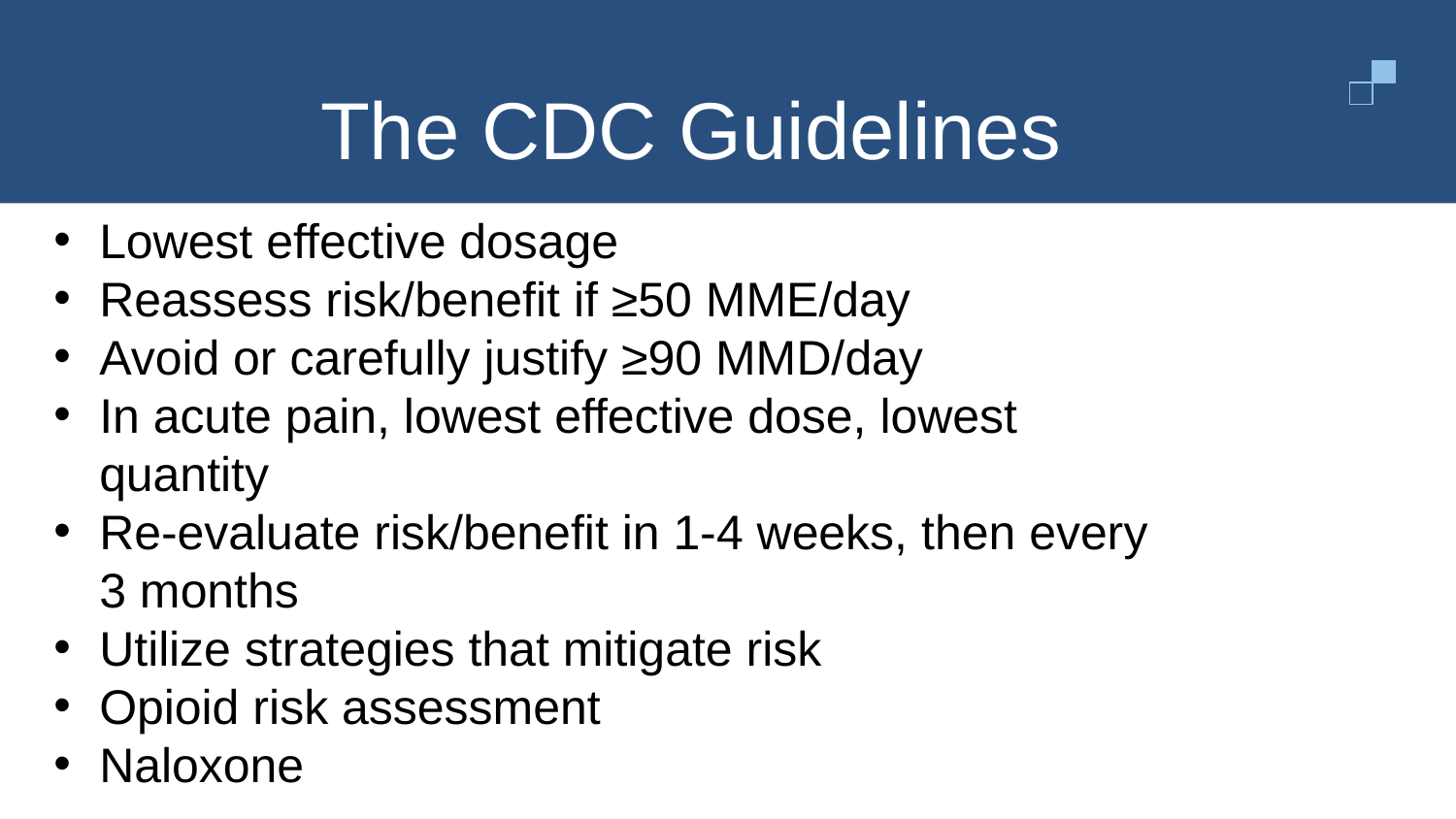

# The CDC Guidelines
Lowest effective dosage
Reassess risk/benefit if ≥50 MME/day
Avoid or carefully justify ≥90 MMD/day
In acute pain, lowest effective dose, lowest quantity
Re-evaluate risk/benefit in 1-4 weeks, then every 3 months
Utilize strategies that mitigate risk
Opioid risk assessment
Naloxone

## Slide 6
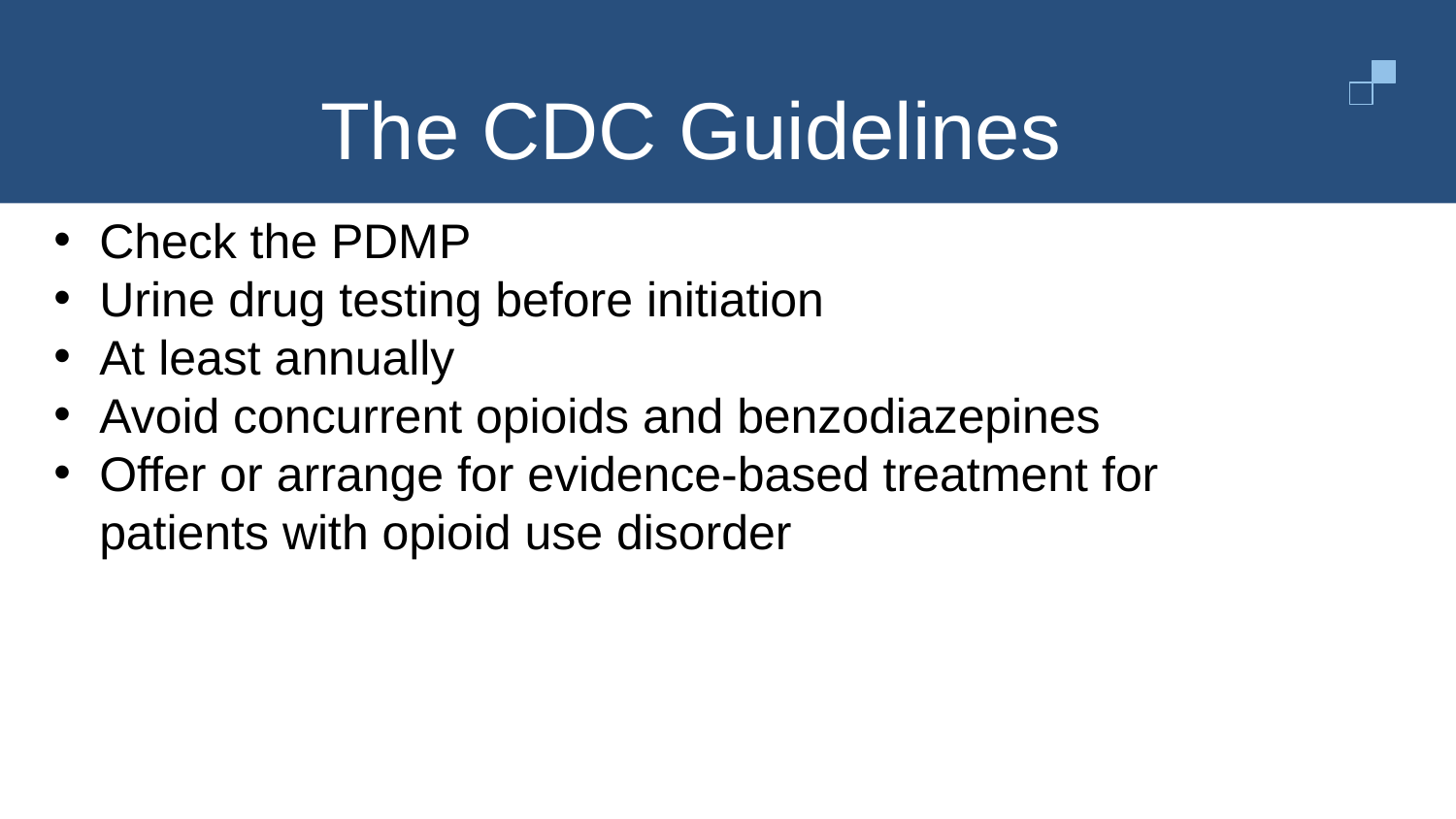

# The CDC Guidelines
Check the PDMP
Urine drug testing before initiation
At least annually
Avoid concurrent opioids and benzodiazepines
Offer or arrange for evidence-based treatment for patients with opioid use disorder

## Slide 7
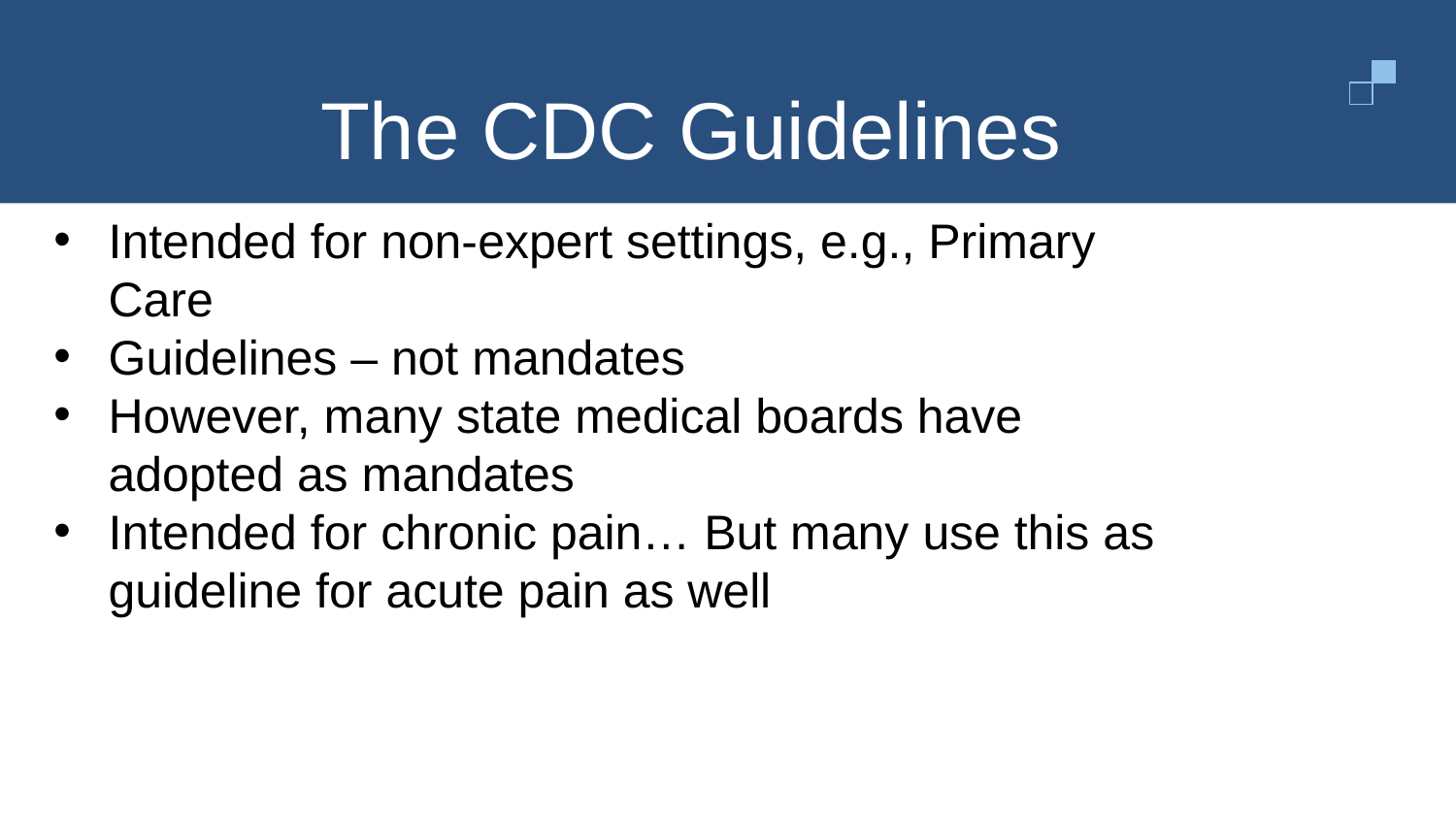

# The CDC Guidelines
Intended for non-expert settings, e.g., Primary Care
Guidelines – not mandates
However, many state medical boards have adopted as mandates
Intended for chronic pain… But many use this as guideline for acute pain as well

## Slide 8
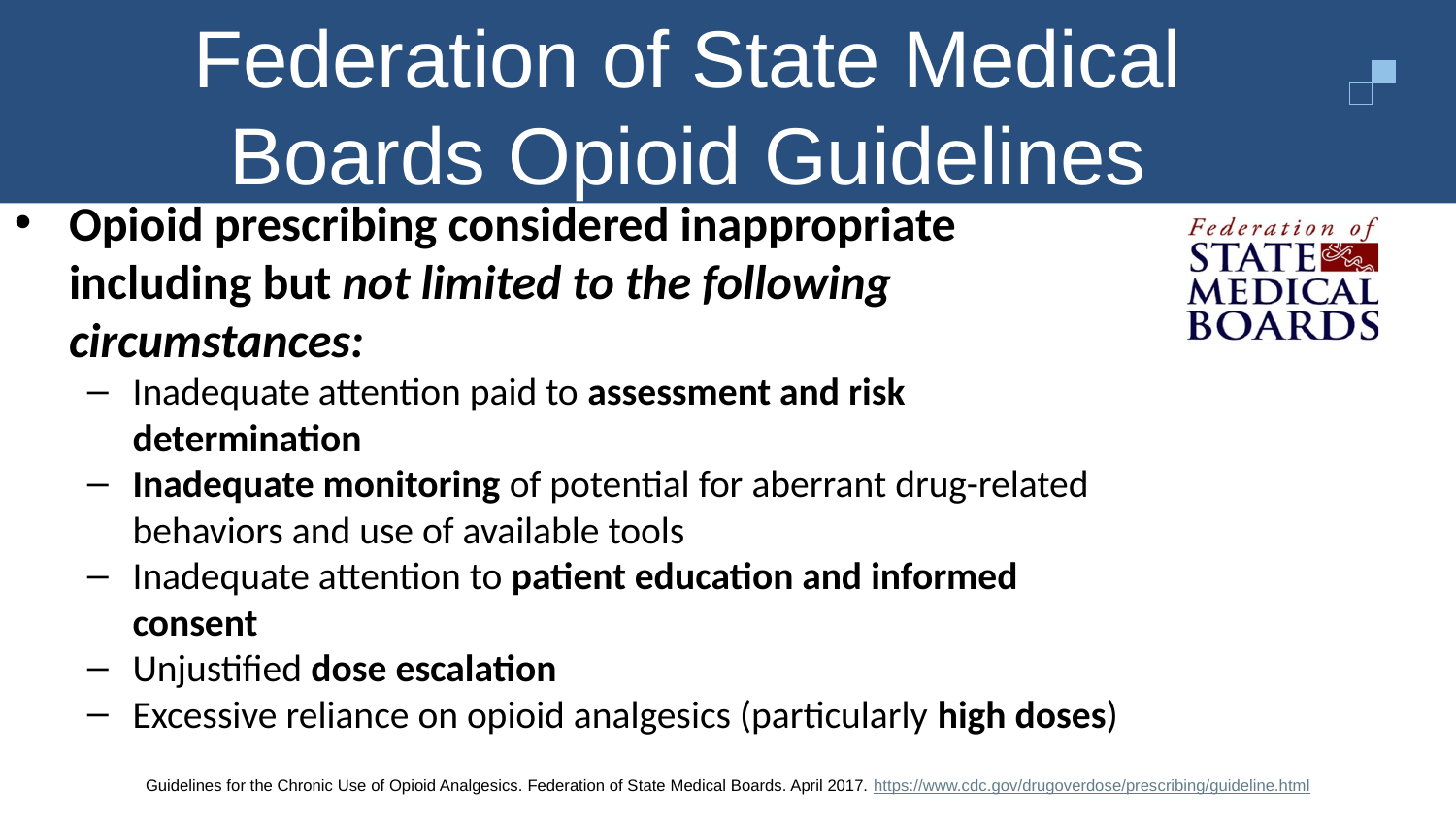

# Federation of State Medical Boards Opioid Guidelines
Opioid prescribing considered inappropriate including but not limited to the following circumstances:
Inadequate attention paid to assessment and risk determination
Inadequate monitoring of potential for aberrant drug-related behaviors and use of available tools
Inadequate attention to patient education and informed consent
Unjustified dose escalation
Excessive reliance on opioid analgesics (particularly high doses)
Guidelines for the Chronic Use of Opioid Analgesics. Federation of State Medical Boards. April 2017. https://www.cdc.gov/drugoverdose/prescribing/guideline.html

## Slide 9
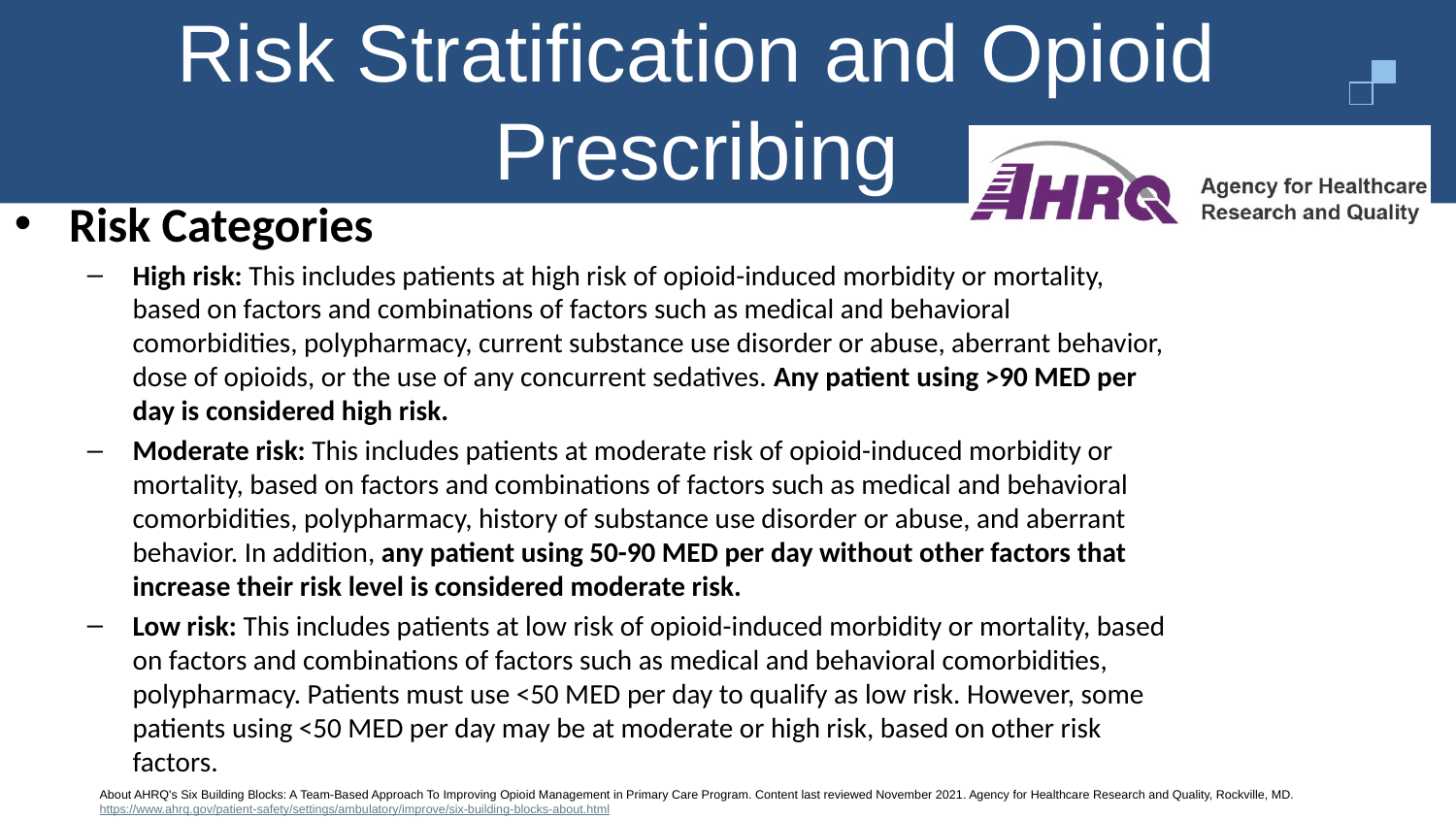

# Risk Stratification and Opioid Prescribing
Risk Categories
High risk: This includes patients at high risk of opioid-induced morbidity or mortality, based on factors and combinations of factors such as medical and behavioral comorbidities, polypharmacy, current substance use disorder or abuse, aberrant behavior, dose of opioids, or the use of any concurrent sedatives. Any patient using >90 MED per day is considered high risk.
Moderate risk: This includes patients at moderate risk of opioid-induced morbidity or mortality, based on factors and combinations of factors such as medical and behavioral comorbidities, polypharmacy, history of substance use disorder or abuse, and aberrant behavior. In addition, any patient using 50-90 MED per day without other factors that increase their risk level is considered moderate risk.
Low risk: This includes patients at low risk of opioid-induced morbidity or mortality, based on factors and combinations of factors such as medical and behavioral comorbidities, polypharmacy. Patients must use <50 MED per day to qualify as low risk. However, some patients using <50 MED per day may be at moderate or high risk, based on other risk factors.
About AHRQ's Six Building Blocks: A Team-Based Approach To Improving Opioid Management in Primary Care Program. Content last reviewed November 2021. Agency for Healthcare Research and Quality, Rockville, MD. https://www.ahrq.gov/patient-safety/settings/ambulatory/improve/six-building-blocks-about.html

## Slide 10
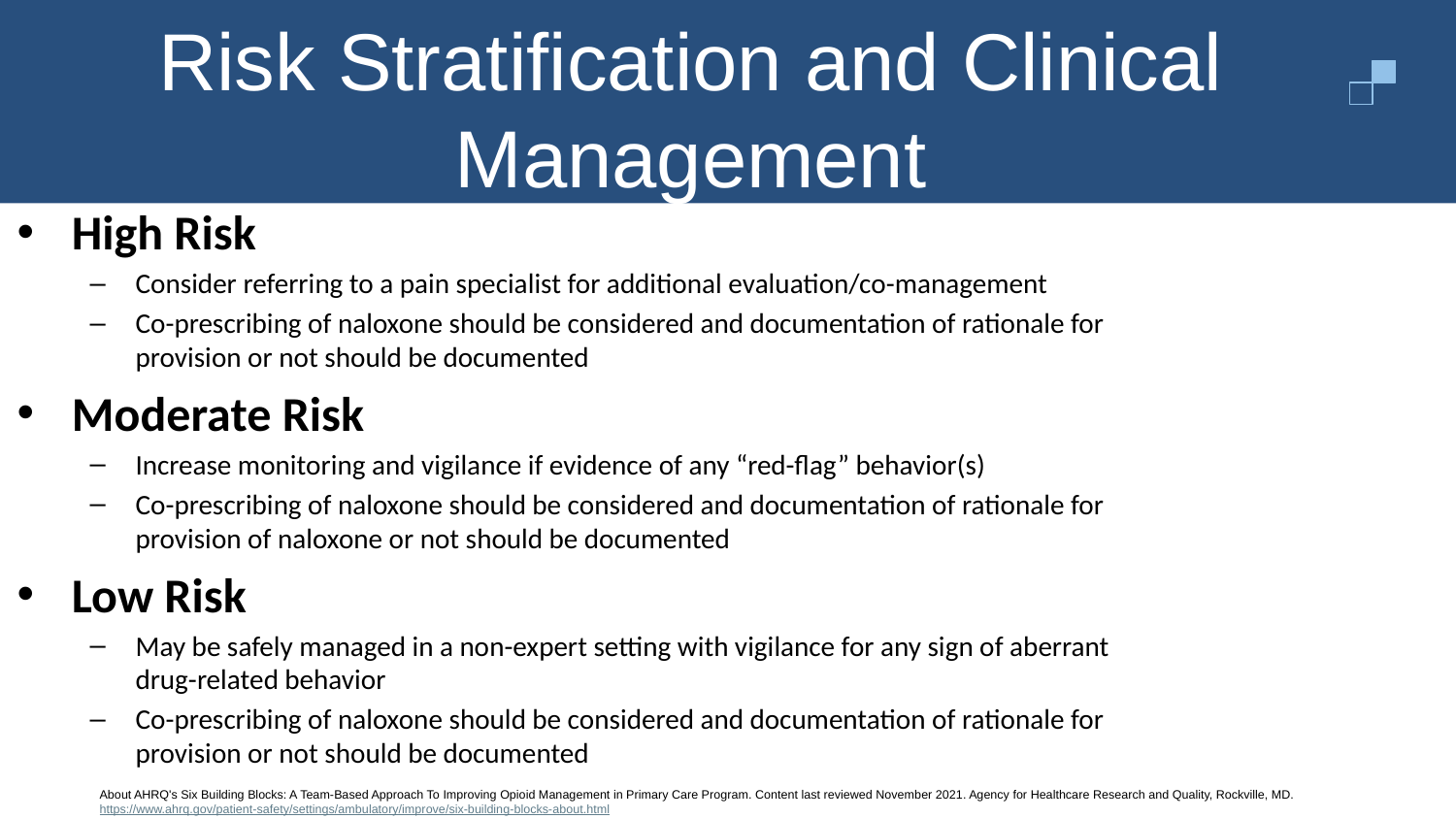

# Risk Stratification and Clinical Management
High Risk
Consider referring to a pain specialist for additional evaluation/co-management
Co-prescribing of naloxone should be considered and documentation of rationale for provision or not should be documented
Moderate Risk
Increase monitoring and vigilance if evidence of any “red-flag” behavior(s)
Co-prescribing of naloxone should be considered and documentation of rationale for provision of naloxone or not should be documented
Low Risk
May be safely managed in a non-expert setting with vigilance for any sign of aberrant drug-related behavior
Co-prescribing of naloxone should be considered and documentation of rationale for provision or not should be documented
About AHRQ's Six Building Blocks: A Team-Based Approach To Improving Opioid Management in Primary Care Program. Content last reviewed November 2021. Agency for Healthcare Research and Quality, Rockville, MD. https://www.ahrq.gov/patient-safety/settings/ambulatory/improve/six-building-blocks-about.html

## Slide 11
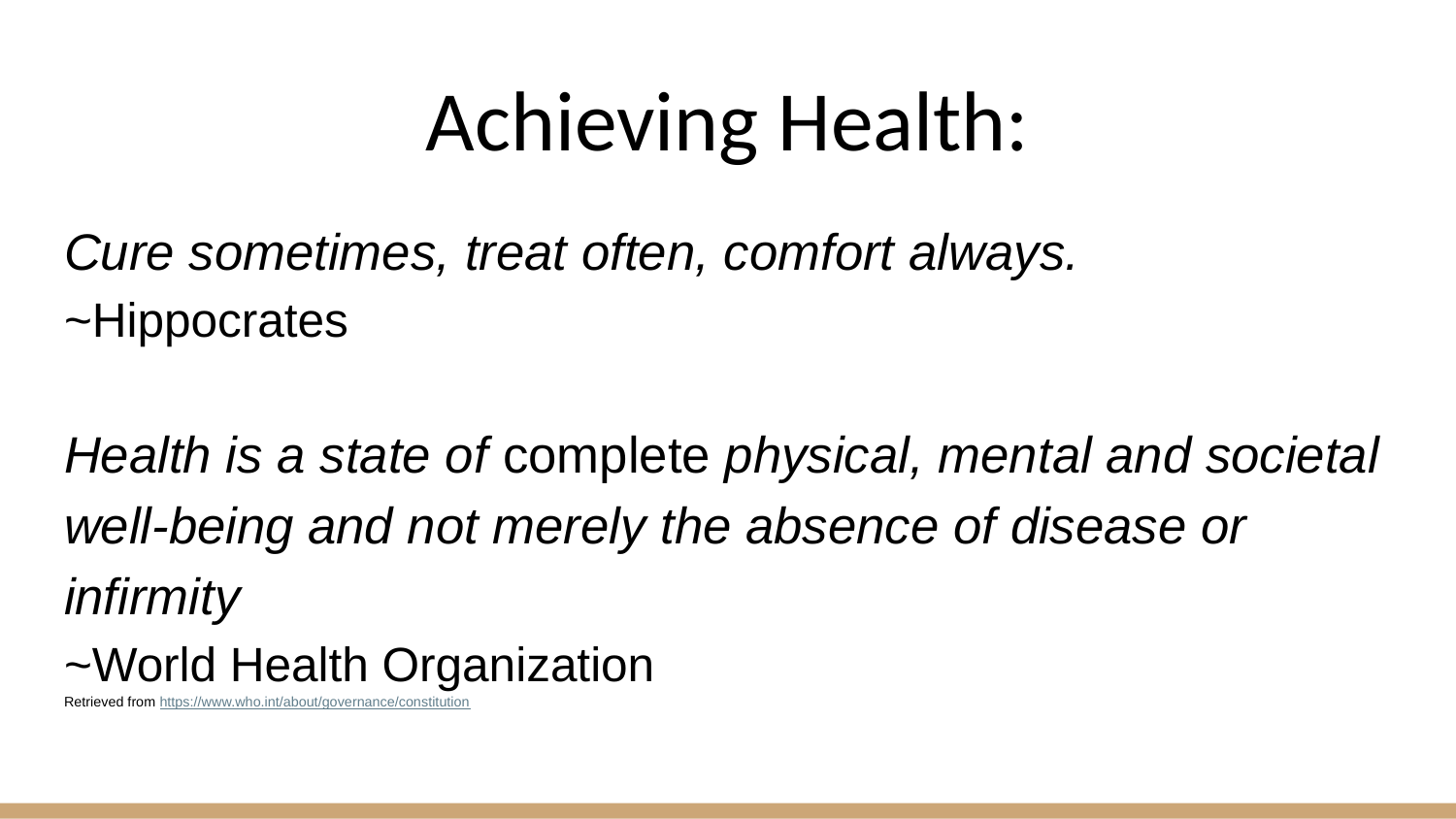

# Achieving Health:
Cure sometimes, treat often, comfort always.
~Hippocrates
Health is a state of complete physical, mental and societal well-being and not merely the absence of disease or infirmity
~World Health Organization
Retrieved from https://www.who.int/about/governance/constitution

## Slide 12
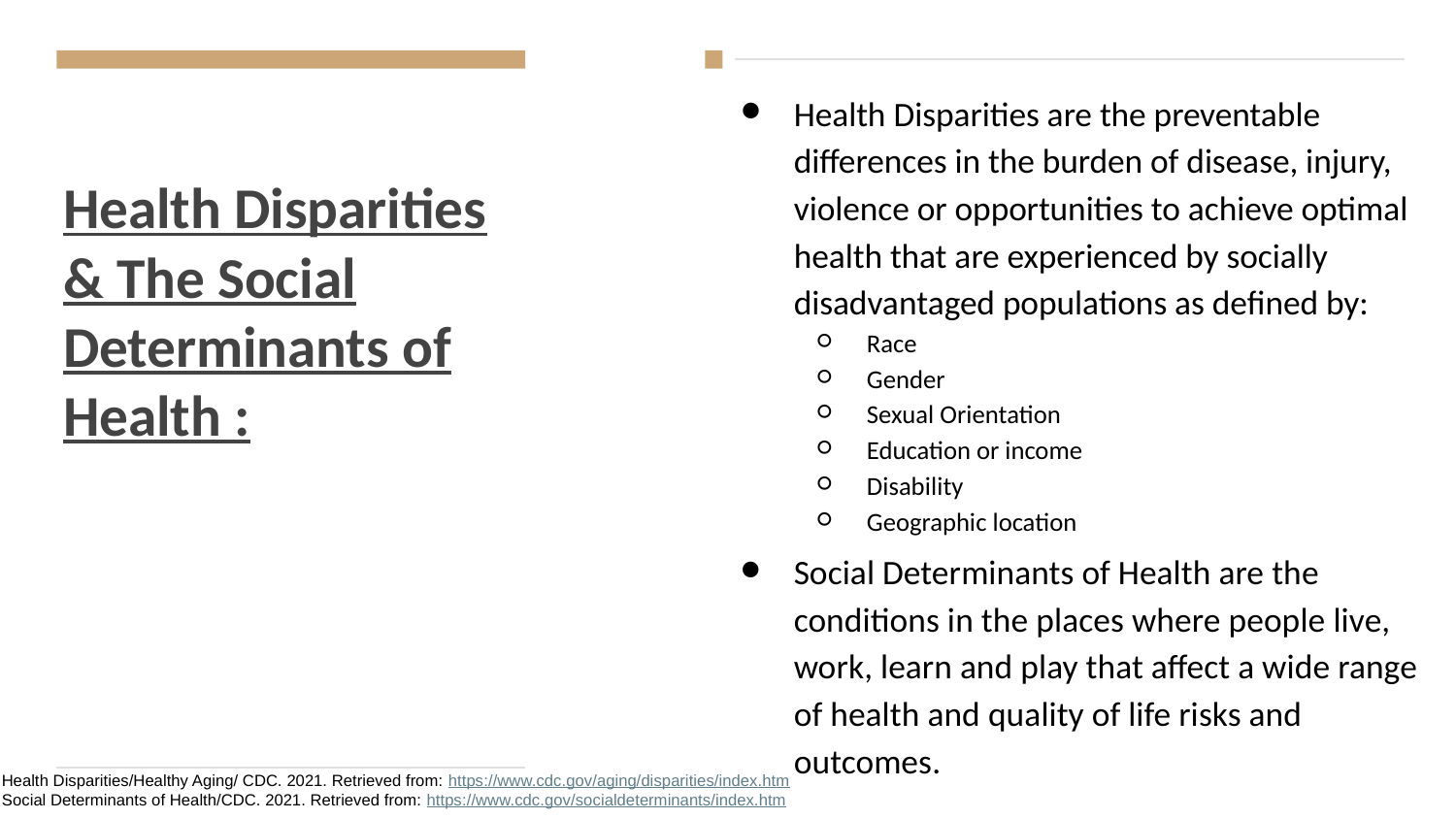

Health Disparities are the preventable differences in the burden of disease, injury, violence or opportunities to achieve optimal health that are experienced by socially disadvantaged populations as defined by:
Race
Gender
Sexual Orientation
Education or income
Disability
Geographic location
# Health Disparities & The Social Determinants of Health :
Social Determinants of Health are the conditions in the places where people live, work, learn and play that affect a wide range of health and quality of life risks and outcomes.
Health Disparities/Healthy Aging/ CDC. 2021. Retrieved from: https://www.cdc.gov/aging/disparities/index.htm
Social Determinants of Health/CDC. 2021. Retrieved from: https://www.cdc.gov/socialdeterminants/index.htm

## Slide 13
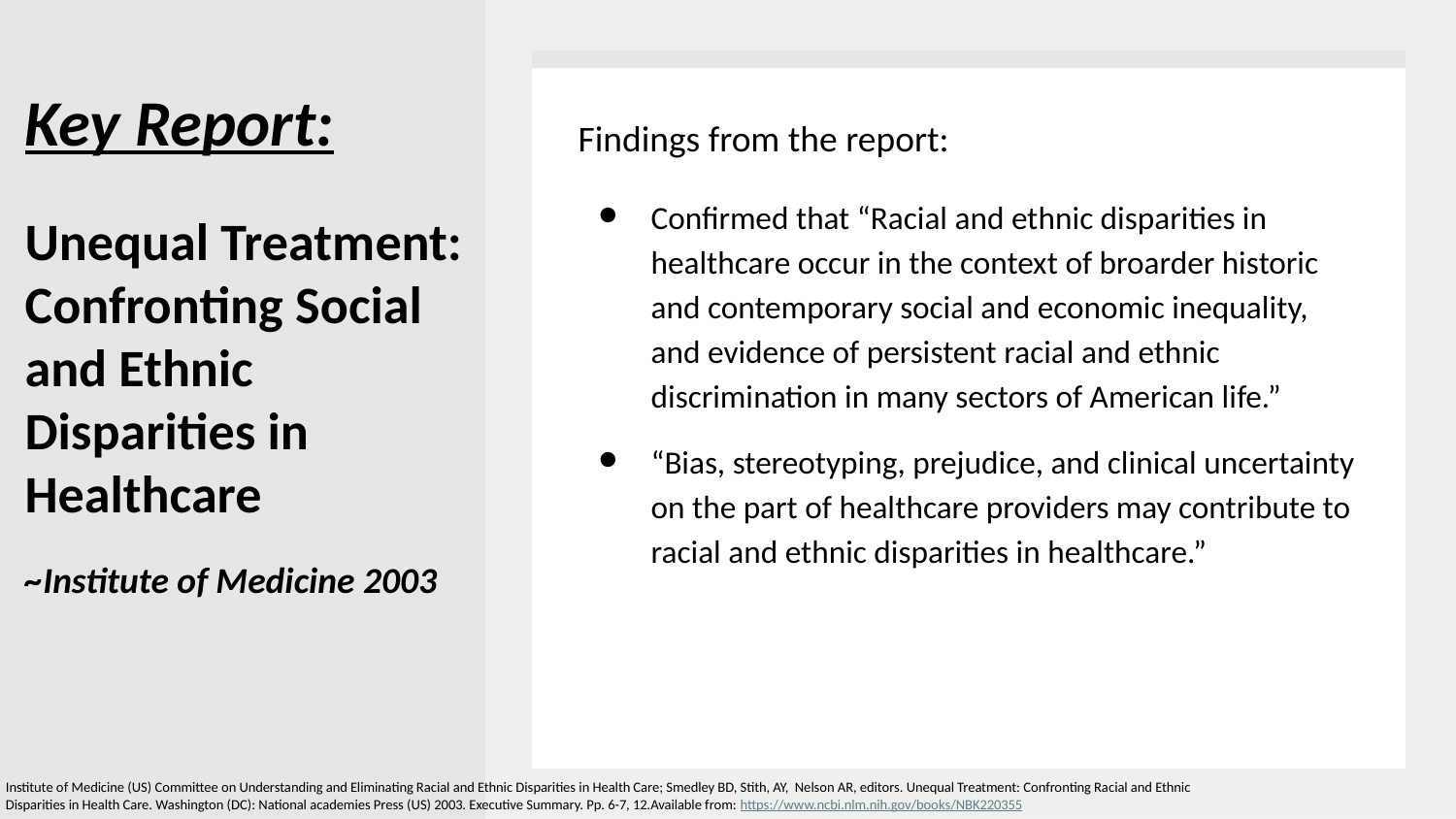

# Key Report:
Unequal Treatment:
Confronting Social and Ethnic Disparities in Healthcare
~Institute of Medicine 2003
Findings from the report:
Confirmed that “Racial and ethnic disparities in healthcare occur in the context of broarder historic and contemporary social and economic inequality, and evidence of persistent racial and ethnic discrimination in many sectors of American life.”
“Bias, stereotyping, prejudice, and clinical uncertainty on the part of healthcare providers may contribute to racial and ethnic disparities in healthcare.”
Institute of Medicine (US) Committee on Understanding and Eliminating Racial and Ethnic Disparities in Health Care; Smedley BD, Stith, AY, Nelson AR, editors. Unequal Treatment: Confronting Racial and Ethnic Disparities in Health Care. Washington (DC): National academies Press (US) 2003. Executive Summary. Pp. 6-7, 12.Available from: https://www.ncbi.nlm.nih.gov/books/NBK220355

## Slide 14
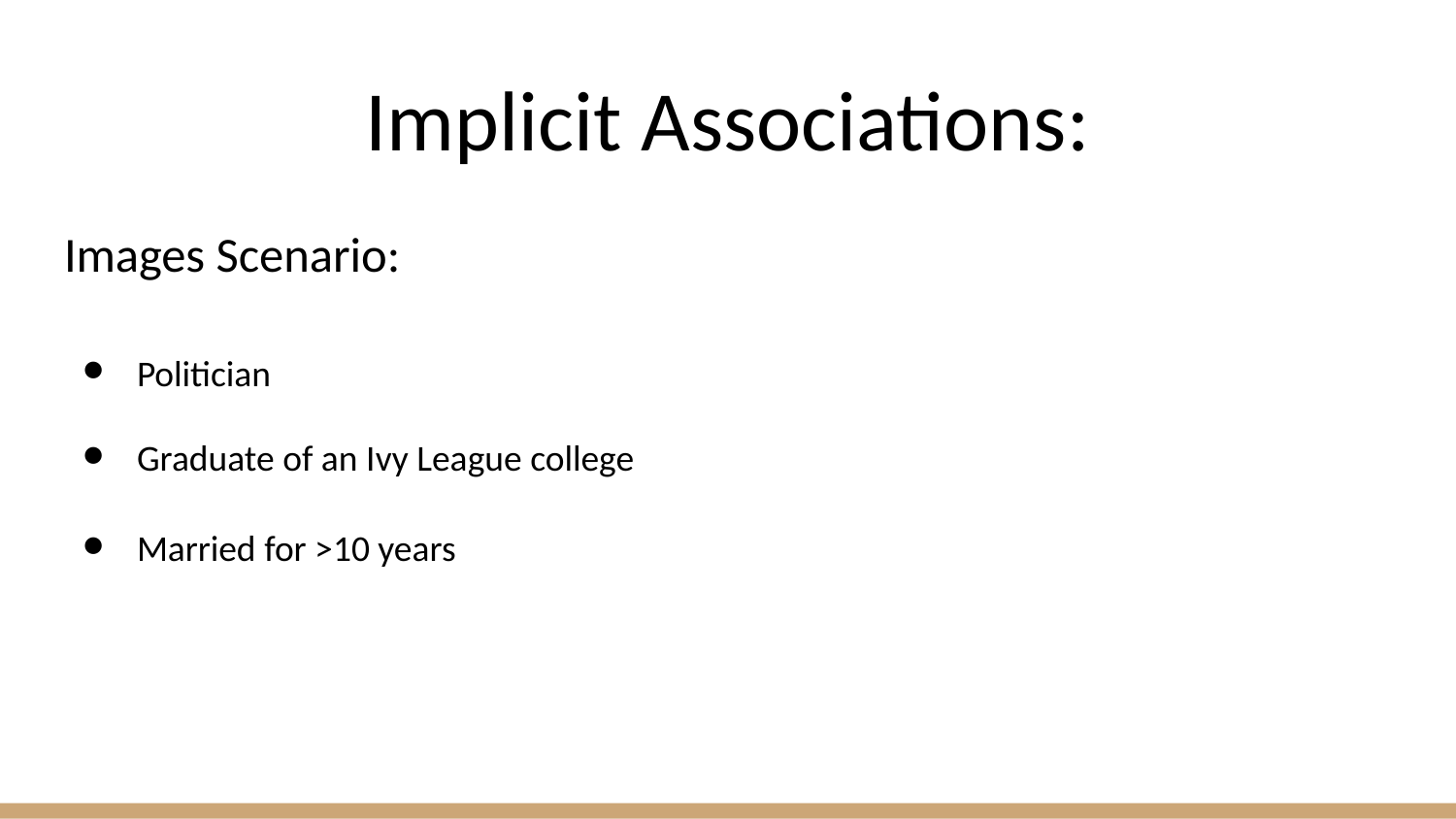

# Implicit Associations:
Images Scenario:
Politician
Graduate of an Ivy League college
Married for >10 years

## Slide 15
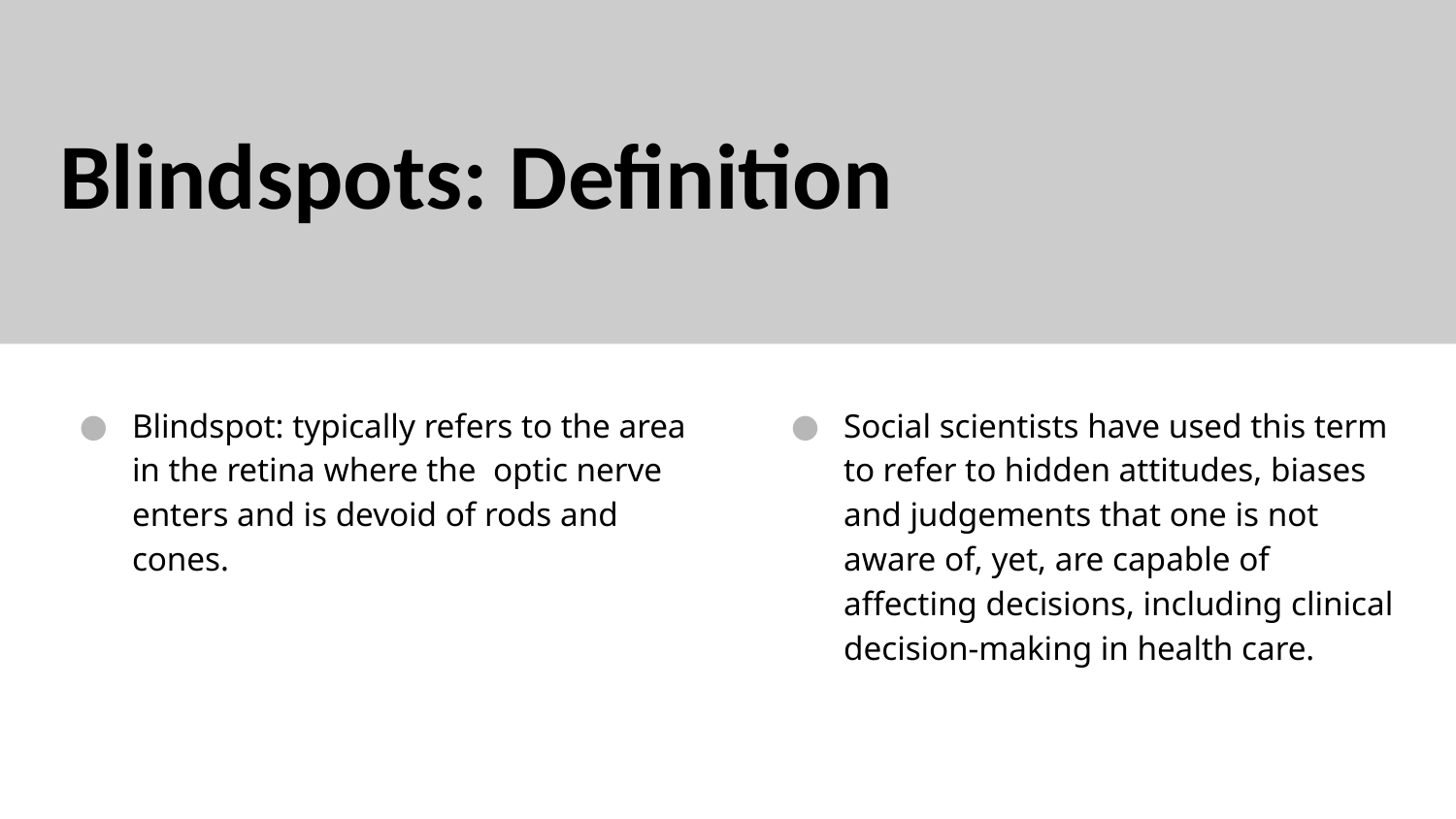

# Blindspots: Definition
Blindspot: typically refers to the area in the retina where the optic nerve enters and is devoid of rods and cones.
Social scientists have used this term to refer to hidden attitudes, biases and judgements that one is not aware of, yet, are capable of affecting decisions, including clinical decision-making in health care.

## Slide 16
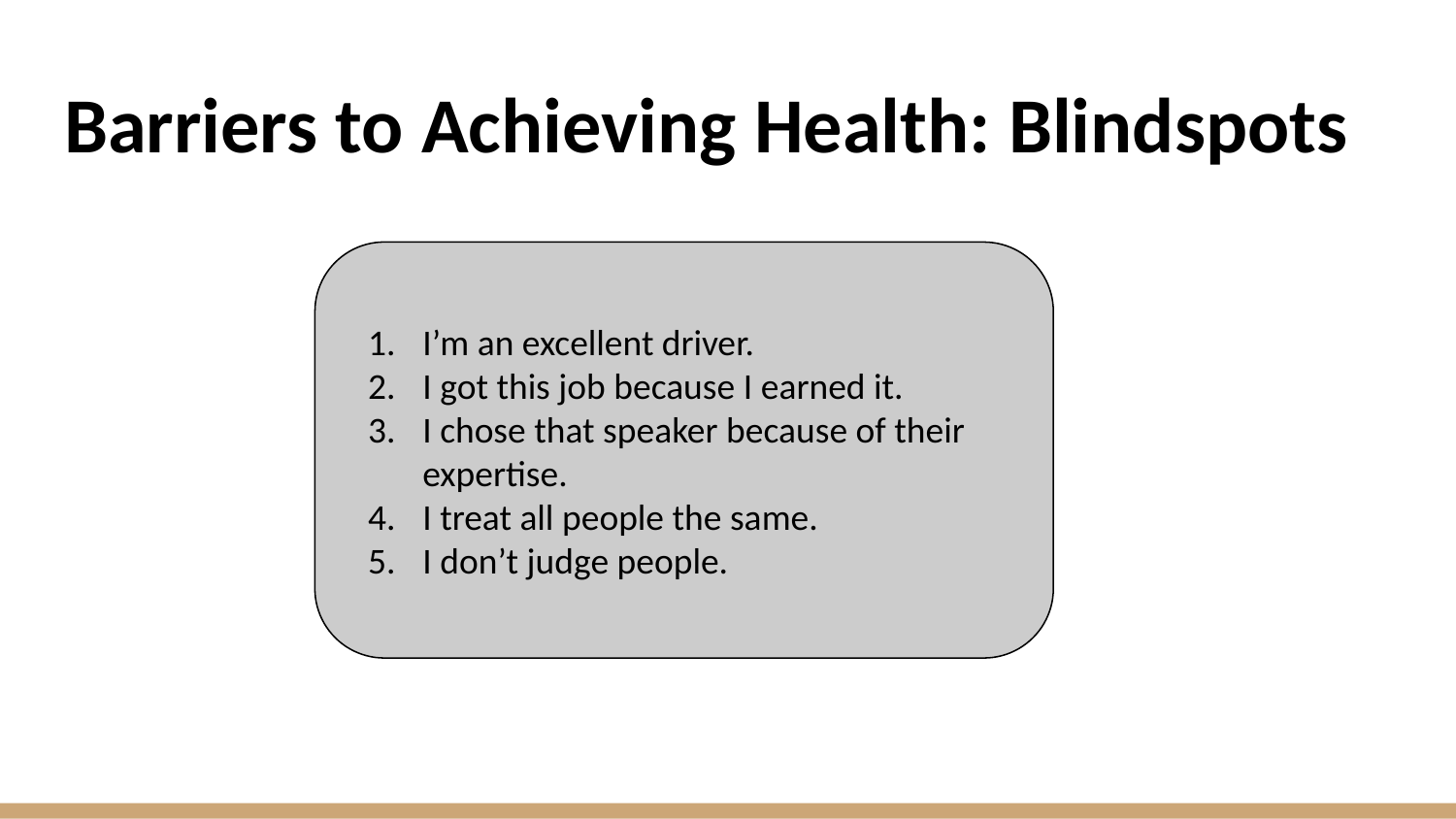

# Barriers to Achieving Health: Blindspots
I’m an excellent driver.
I got this job because I earned it.
I chose that speaker because of their expertise.
I treat all people the same.
I don’t judge people.

## Slide 17
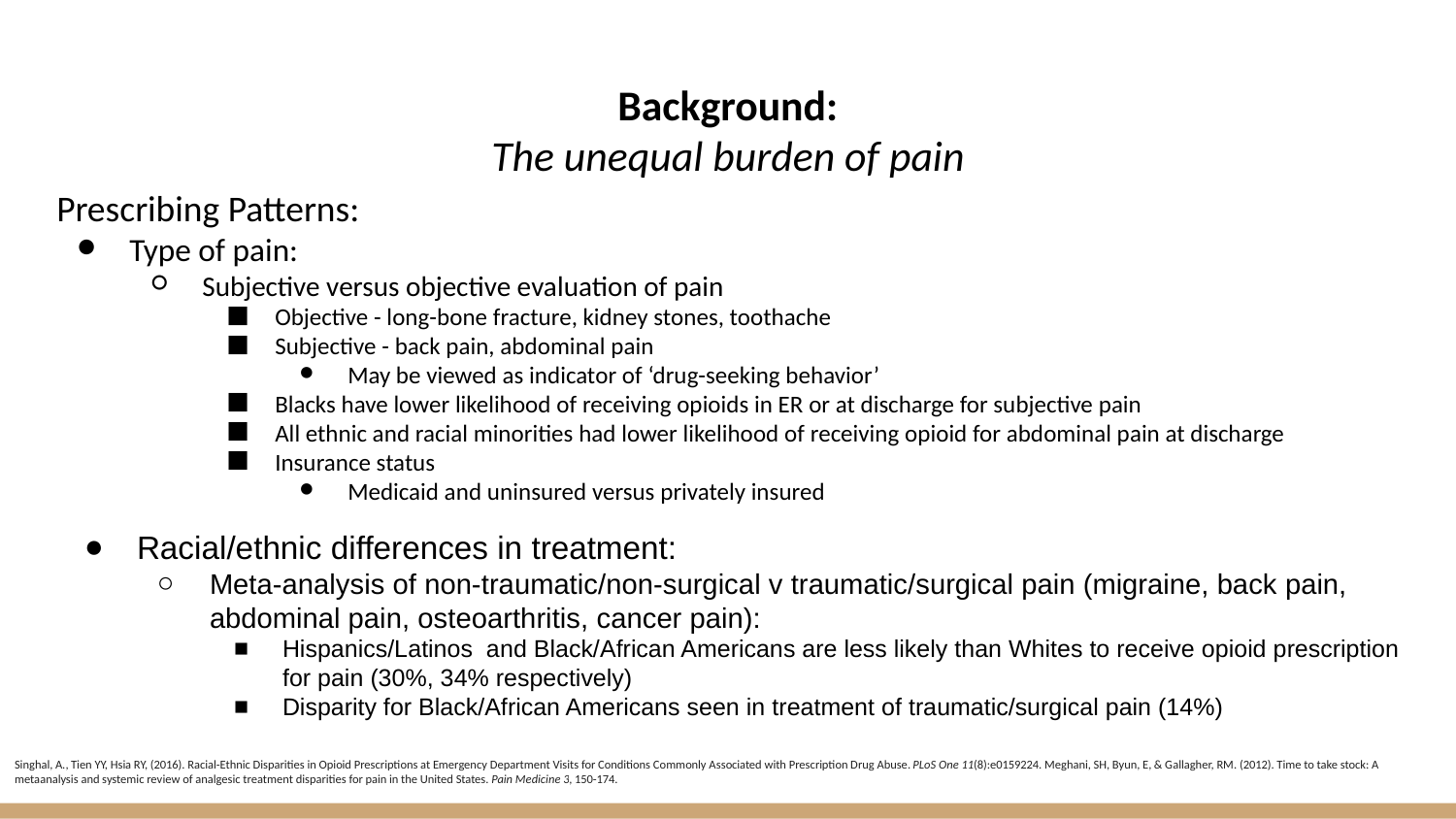

# Background:
The unequal burden of pain
Prescribing Patterns:
Type of pain:
Subjective versus objective evaluation of pain
Objective - long-bone fracture, kidney stones, toothache
Subjective - back pain, abdominal pain
May be viewed as indicator of ‘drug-seeking behavior’
Blacks have lower likelihood of receiving opioids in ER or at discharge for subjective pain
All ethnic and racial minorities had lower likelihood of receiving opioid for abdominal pain at discharge
Insurance status
Medicaid and uninsured versus privately insured
Racial/ethnic differences in treatment:
Meta-analysis of non-traumatic/non-surgical v traumatic/surgical pain (migraine, back pain, abdominal pain, osteoarthritis, cancer pain):
Hispanics/Latinos and Black/African Americans are less likely than Whites to receive opioid prescription for pain (30%, 34% respectively)
Disparity for Black/African Americans seen in treatment of traumatic/surgical pain (14%)
Singhal, A., Tien YY, Hsia RY, (2016). Racial-Ethnic Disparities in Opioid Prescriptions at Emergency Department Visits for Conditions Commonly Associated with Prescription Drug Abuse. PLoS One 11(8):e0159224. Meghani, SH, Byun, E, & Gallagher, RM. (2012). Time to take stock: A metaanalysis and systemic review of analgesic treatment disparities for pain in the United States. Pain Medicine 3, 150-174.

## Slide 18
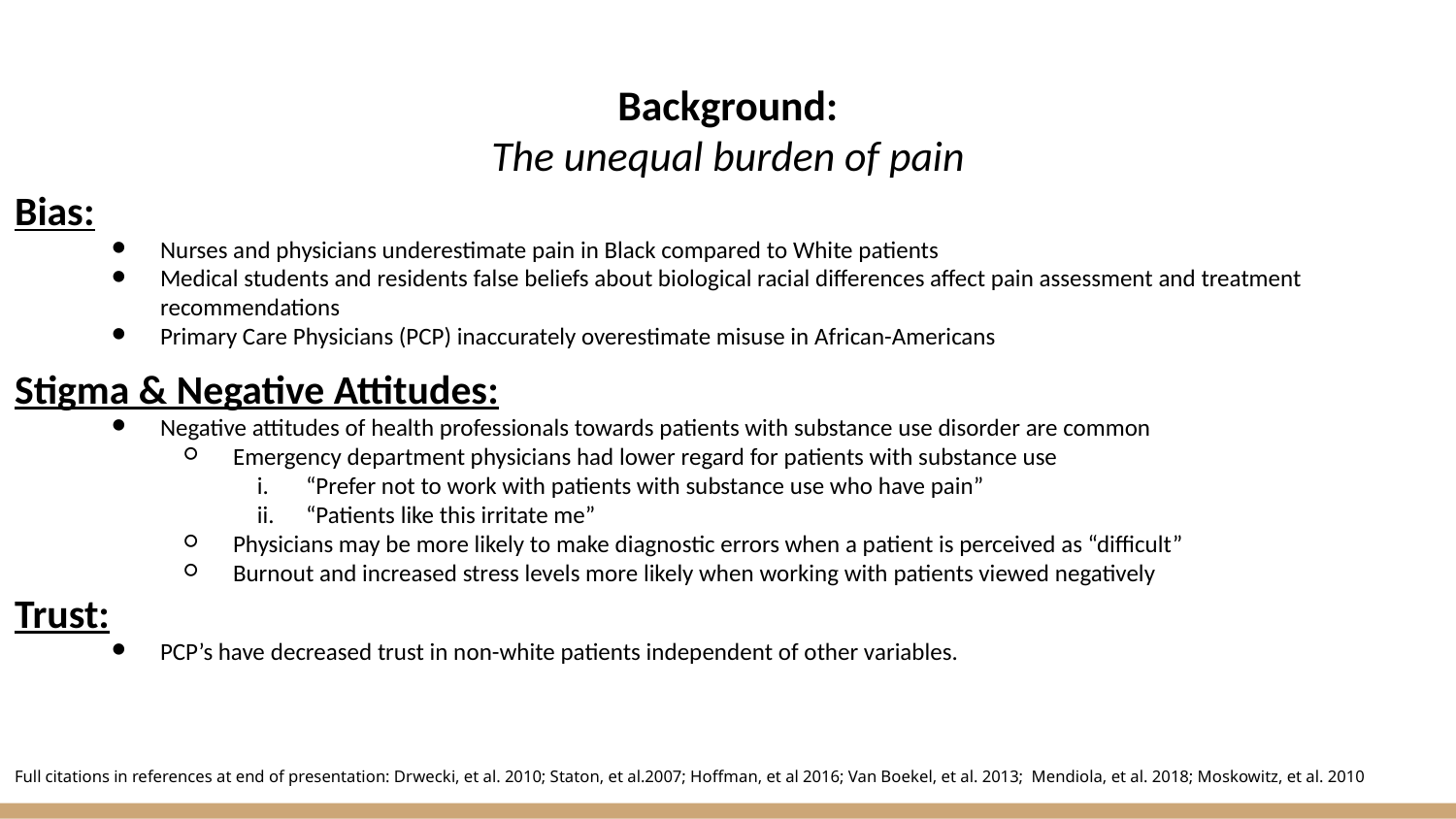

# Background:
The unequal burden of pain
Bias:
Nurses and physicians underestimate pain in Black compared to White patients
Medical students and residents false beliefs about biological racial differences affect pain assessment and treatment recommendations
Primary Care Physicians (PCP) inaccurately overestimate misuse in African-Americans
Stigma & Negative Attitudes:
Negative attitudes of health professionals towards patients with substance use disorder are common
Emergency department physicians had lower regard for patients with substance use
“Prefer not to work with patients with substance use who have pain”
“Patients like this irritate me”
Physicians may be more likely to make diagnostic errors when a patient is perceived as “difficult”
Burnout and increased stress levels more likely when working with patients viewed negatively
Trust:
PCP’s have decreased trust in non-white patients independent of other variables.
Full citations in references at end of presentation: Drwecki, et al. 2010; Staton, et al.2007; Hoffman, et al 2016; Van Boekel, et al. 2013; Mendiola, et al. 2018; Moskowitz, et al. 2010

## Slide 19
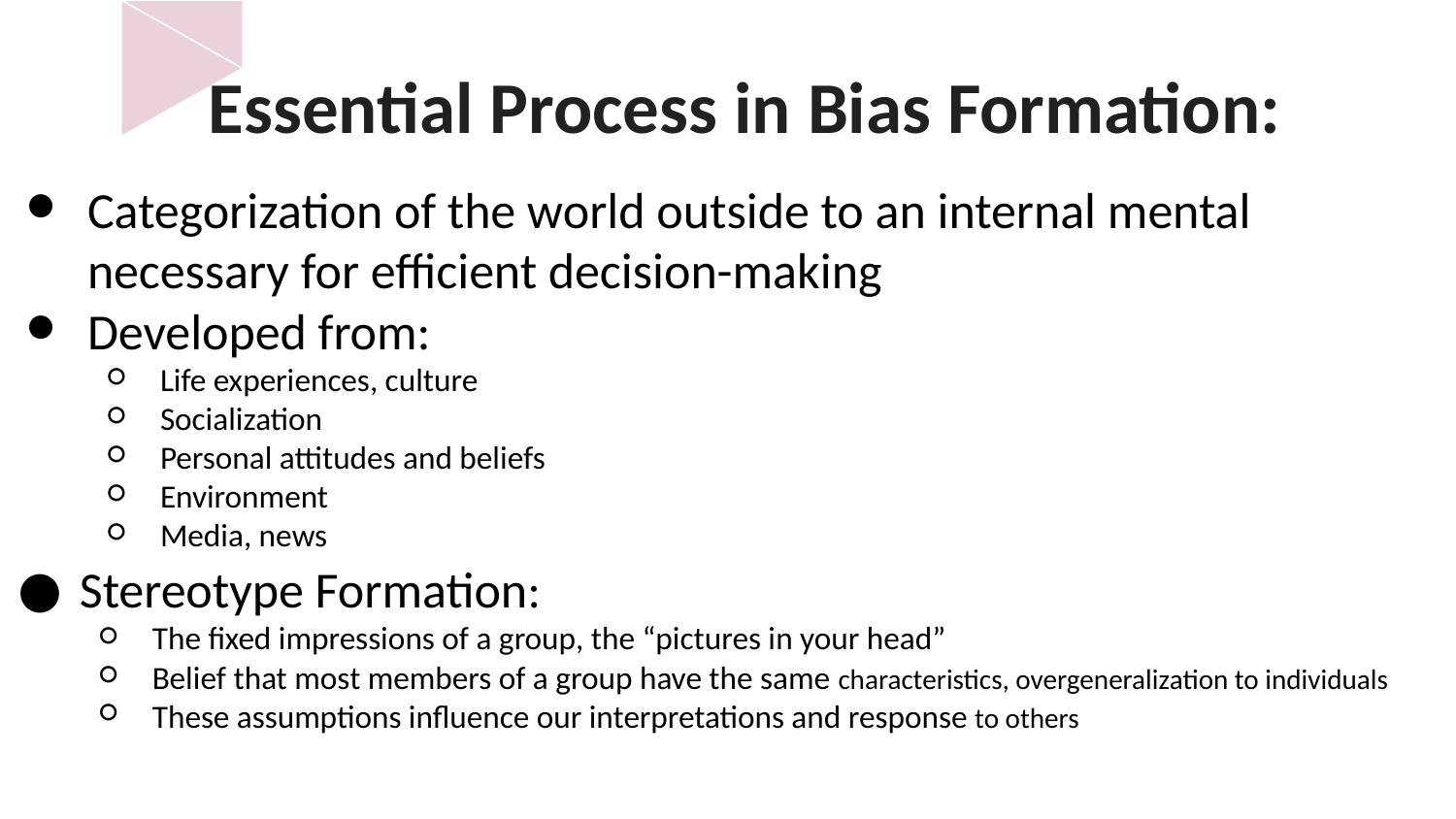

# Essential Process in Bias Formation:
Categorization of the world outside to an internal mental necessary for efficient decision-making
Developed from:
Life experiences, culture
Socialization
Personal attitudes and beliefs
Environment
Media, news
Stereotype Formation:
The fixed impressions of a group, the “pictures in your head”
Belief that most members of a group have the same characteristics, overgeneralization to individuals
These assumptions influence our interpretations and response to others

## Slide 20
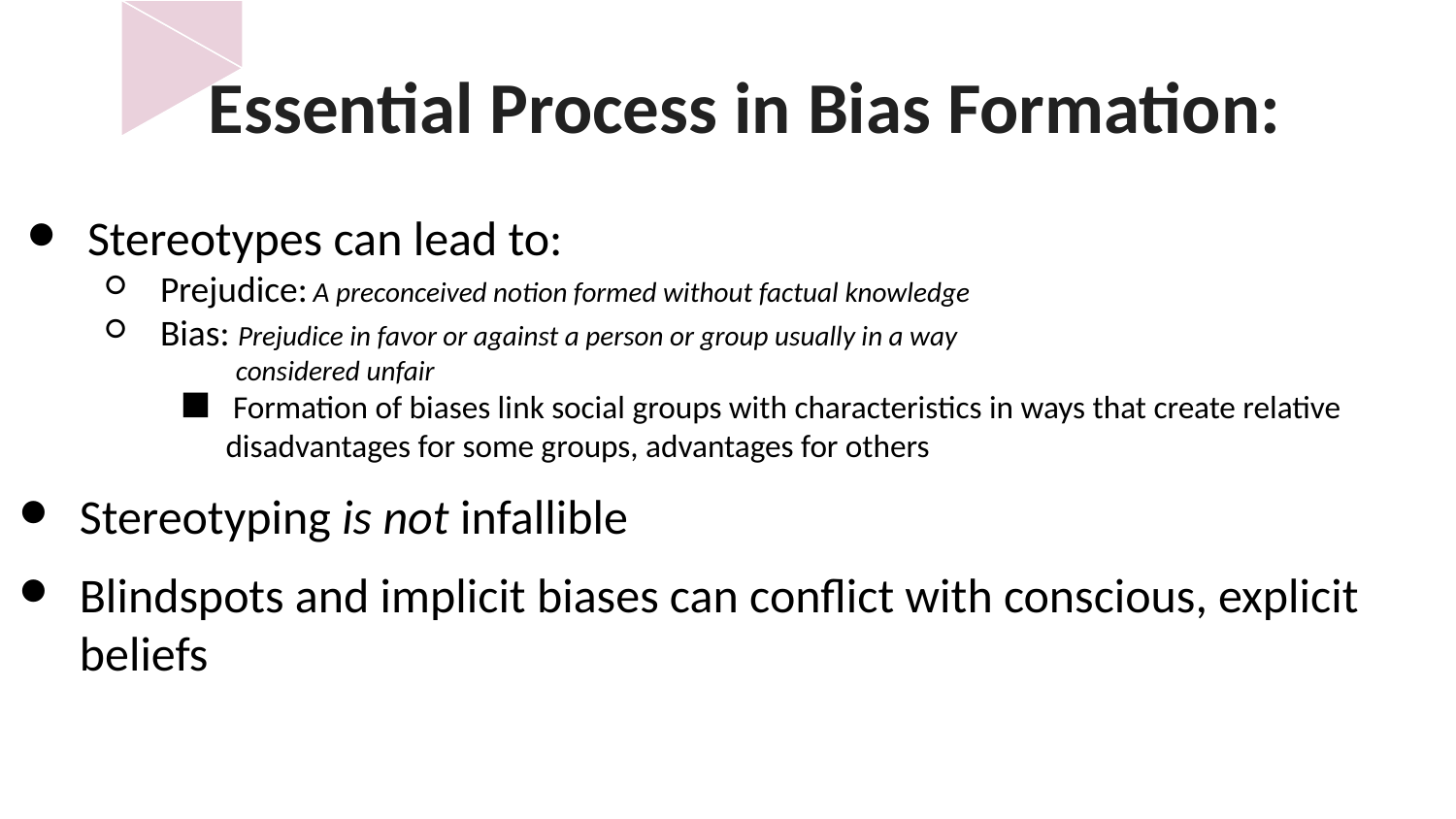

# Essential Process in Bias Formation:
Stereotypes can lead to:
Prejudice: A preconceived notion formed without factual knowledge
Bias: Prejudice in favor or against a person or group usually in a way
 considered unfair
Formation of biases link social groups with characteristics in ways that create relative
 disadvantages for some groups, advantages for others
Stereotyping is not infallible
Blindspots and implicit biases can conflict with conscious, explicit beliefs

## Slide 21
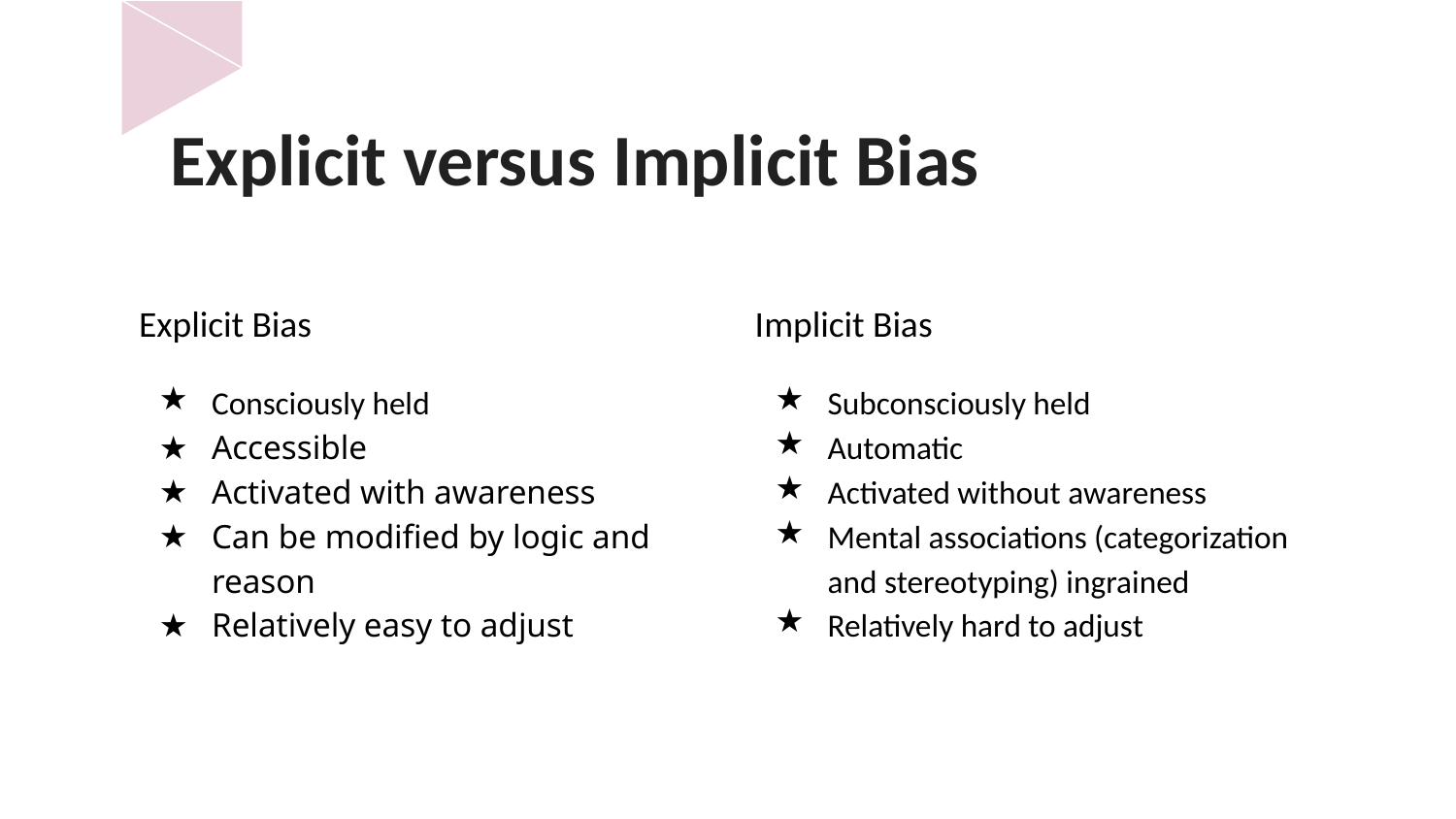

# Explicit versus Implicit Bias
Explicit Bias
Consciously held
Accessible
Activated with awareness
Can be modified by logic and reason
Relatively easy to adjust
Implicit Bias
Subconsciously held
Automatic
Activated without awareness
Mental associations (categorization and stereotyping) ingrained
Relatively hard to adjust

## Slide 22
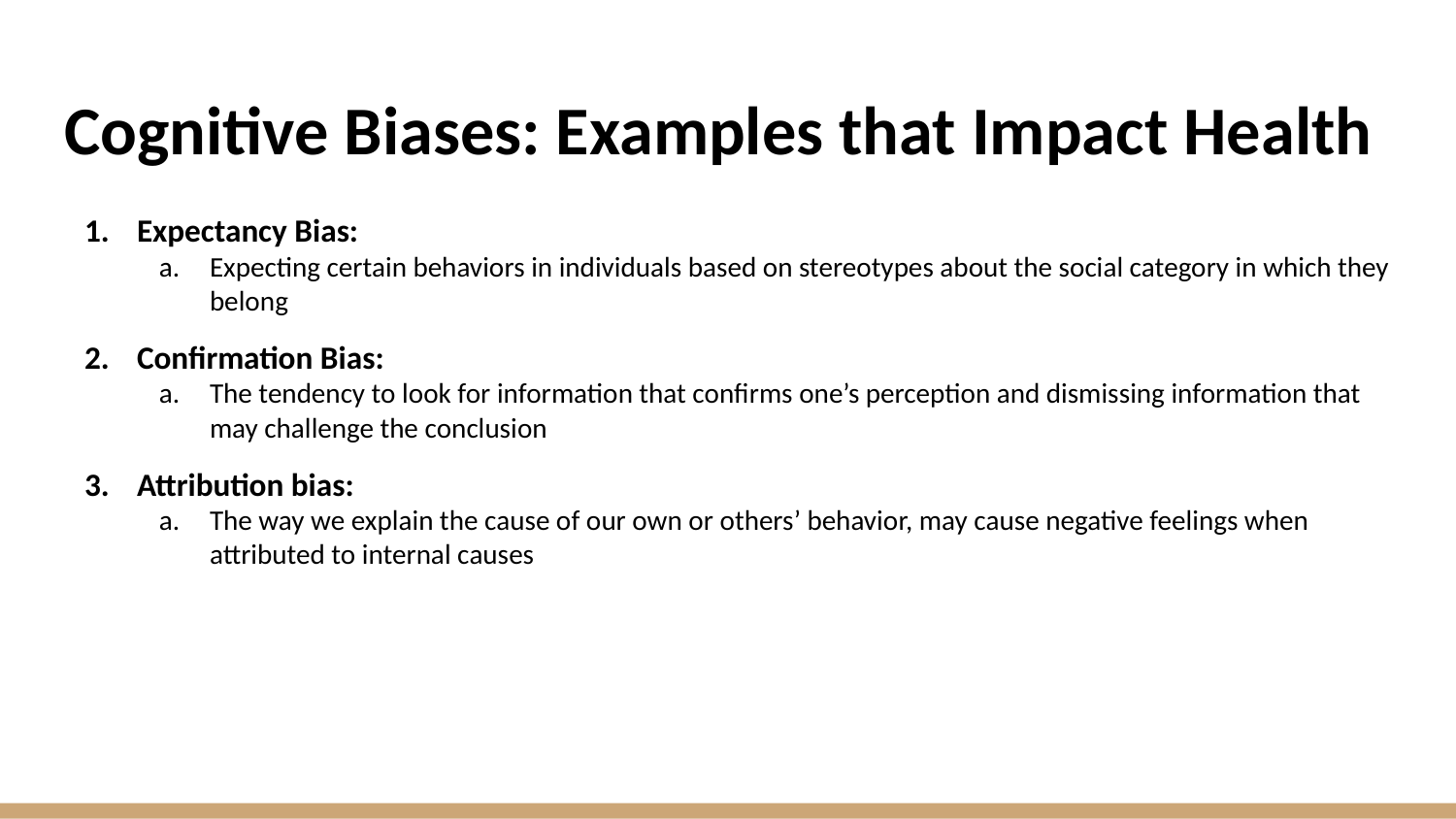

# Cognitive Biases: Examples that Impact Health
Expectancy Bias:
Expecting certain behaviors in individuals based on stereotypes about the social category in which they belong
Confirmation Bias:
The tendency to look for information that confirms one’s perception and dismissing information that may challenge the conclusion
Attribution bias:
The way we explain the cause of our own or others’ behavior, may cause negative feelings when attributed to internal causes

## Slide 23
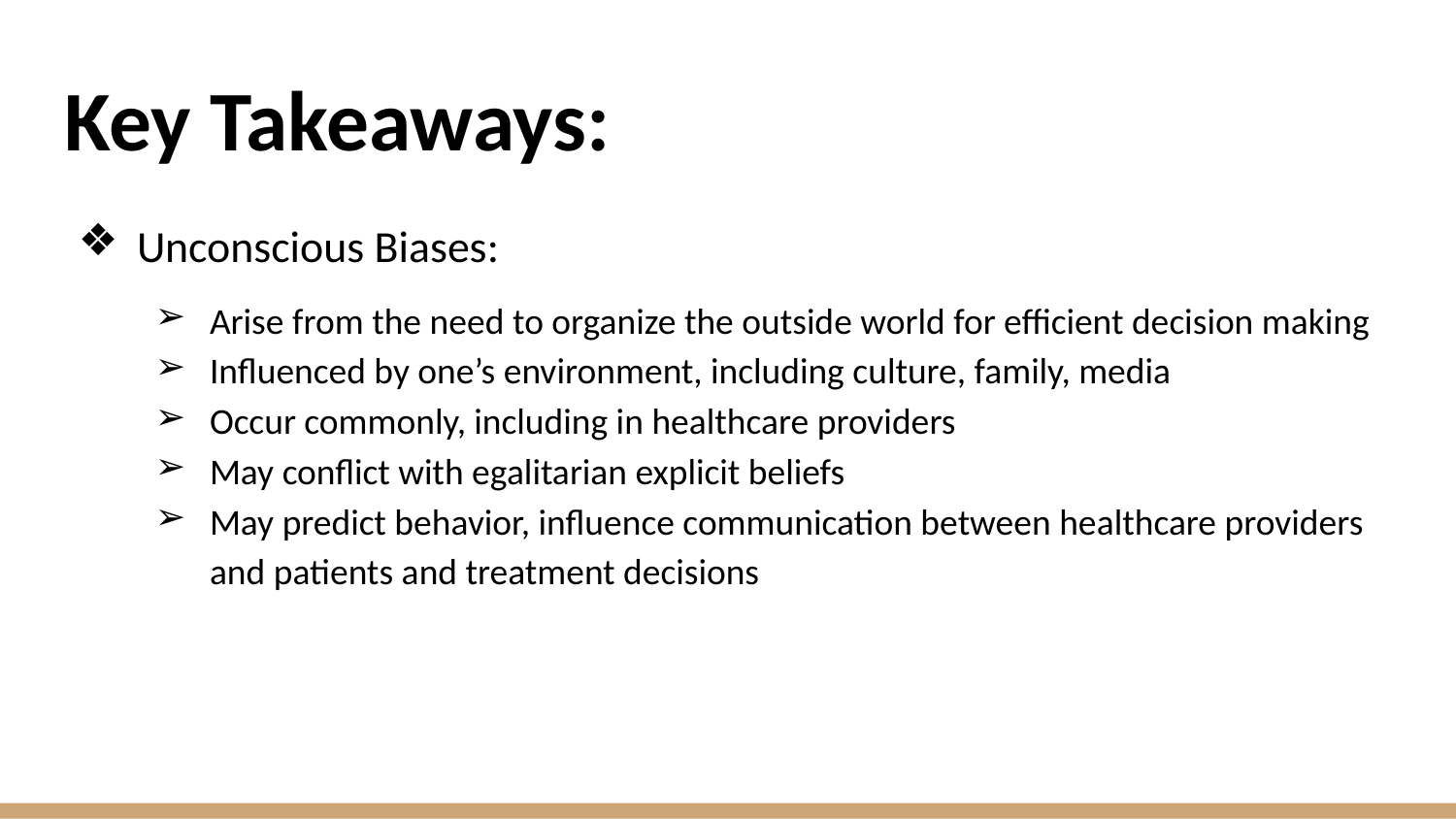

# Key Takeaways:
Unconscious Biases:
Arise from the need to organize the outside world for efficient decision making
Influenced by one’s environment, including culture, family, media
Occur commonly, including in healthcare providers
May conflict with egalitarian explicit beliefs
May predict behavior, influence communication between healthcare providers and patients and treatment decisions

## Slide 24
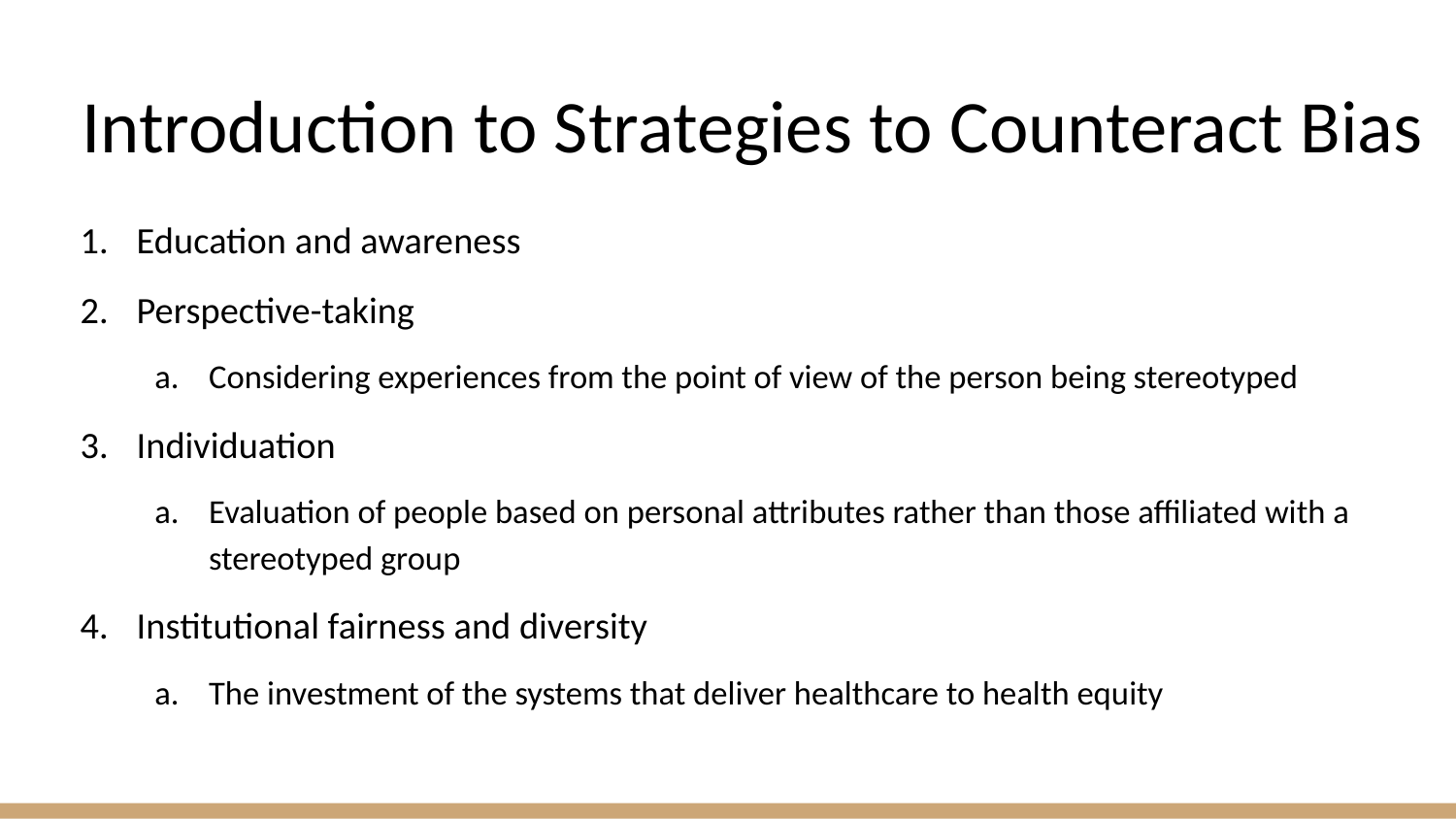

# Introduction to Strategies to Counteract Bias
Education and awareness
Perspective-taking
Considering experiences from the point of view of the person being stereotyped
Individuation
Evaluation of people based on personal attributes rather than those affiliated with a stereotyped group
Institutional fairness and diversity
The investment of the systems that deliver healthcare to health equity

## Slide 25
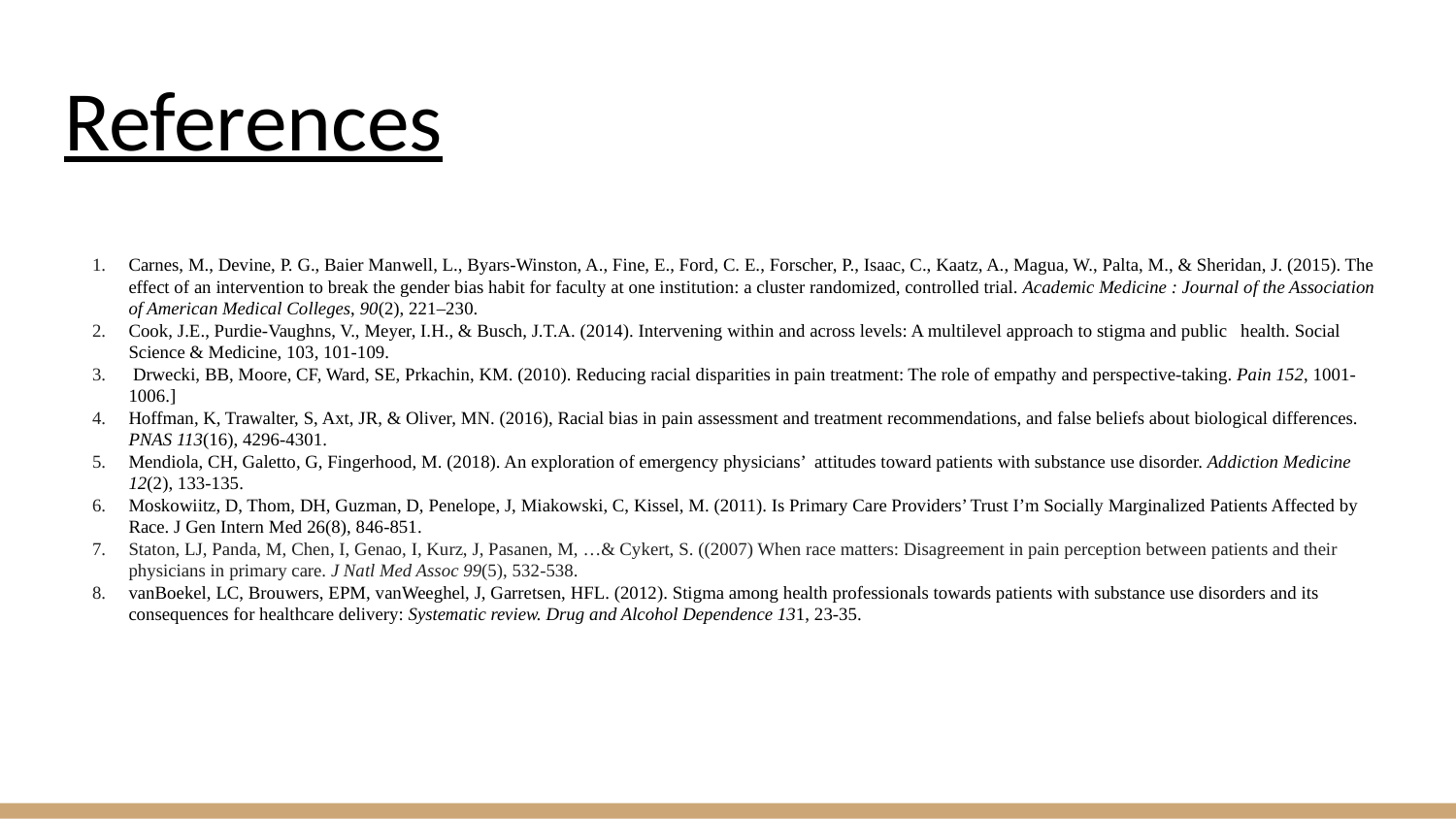

# References
Carnes, M., Devine, P. G., Baier Manwell, L., Byars-Winston, A., Fine, E., Ford, C. E., Forscher, P., Isaac, C., Kaatz, A., Magua, W., Palta, M., & Sheridan, J. (2015). The effect of an intervention to break the gender bias habit for faculty at one institution: a cluster randomized, controlled trial. Academic Medicine : Journal of the Association of American Medical Colleges, 90(2), 221–230.
Cook, J.E., Purdie-Vaughns, V., Meyer, I.H., & Busch, J.T.A. (2014). Intervening within and across levels: A multilevel approach to stigma and public health. Social Science & Medicine, 103, 101-109.
 Drwecki, BB, Moore, CF, Ward, SE, Prkachin, KM. (2010). Reducing racial disparities in pain treatment: The role of empathy and perspective-taking. Pain 152, 1001-1006.]
Hoffman, K, Trawalter, S, Axt, JR, & Oliver, MN. (2016), Racial bias in pain assessment and treatment recommendations, and false beliefs about biological differences. PNAS 113(16), 4296-4301.
Mendiola, CH, Galetto, G, Fingerhood, M. (2018). An exploration of emergency physicians’ attitudes toward patients with substance use disorder. Addiction Medicine 12(2), 133-135.
Moskowiitz, D, Thom, DH, Guzman, D, Penelope, J, Miakowski, C, Kissel, M. (2011). Is Primary Care Providers’ Trust I’m Socially Marginalized Patients Affected by Race. J Gen Intern Med 26(8), 846-851.
Staton, LJ, Panda, M, Chen, I, Genao, I, Kurz, J, Pasanen, M, …& Cykert, S. ((2007) When race matters: Disagreement in pain perception between patients and their physicians in primary care. J Natl Med Assoc 99(5), 532-538.
vanBoekel, LC, Brouwers, EPM, vanWeeghel, J, Garretsen, HFL. (2012). Stigma among health professionals towards patients with substance use disorders and its consequences for healthcare delivery: Systematic review. Drug and Alcohol Dependence 131, 23-35.
