# Supplementary material for: Early Refill of an Opioid Medication: Recognizing Personal Biases Through Clinical Vignettes and OSCEs
Source: MedEdPORTAL. 2022 Apr 7;18:11234. doi: 10.15766/mep_2374-8265.11234 (PMC8986891; doi:10.15766/mep_2374-8265.11234)
Supplement: Supplementary file 1 — MS 1 Clinical Vignettes & Follow-Up.pptxMS 1 Debrief.pptxSP James Spiegel - Case 1.docxSP Darryl Whitcomb - Case 2.docxSP Helen Morgan - Case 3.docxDoor Notes.docxLogistical Flow.docxFaculty Post-OSCE Debrief Discussion Guide.docxSP Encounter Checklist.docxSP Responses for Checklist Items.docxMS 3 Post-OSCE Survey.docx [file mep_2374-8265.11234-s001.zip › C. SP James Spiegel - Case 1.docx]

Appendix C. Standardized Patient James Spiegel Case

Date: 1/05/21

Primary Case Author: Kevin L. Zacharoff, MD, FACIP, FACPE, FAAP

Secondary Case Author: Perrilynn Baldelli, DNP, RN, CHSE

Standardized Patient Educator: Denise Antonelle-Mahoney, MS

Name of Case: Request for Early Refill of an Opioid Medication

Name of educational and or assessment activity: Transitions to Clinical Care (TCC) OSCE Case

Patient Name: James Speigel

Chief Complaint: Chronic back pain, requesting an early refill of prescription opioid medication

Most likely Diagnosis and Differential with rationale from history and/or physical exam:

R/O Discogenic back pain

R/O Musculoskeletal pain

R/O Medication-seeking behavior

R/O Aberrant Drug-related behavior

Challenge question: None

Domains: Check all that apply

Professionalism

Communication and Interpersonal skills

Medical History

☒ Physical exam

Shared Decision Making

☒ Patient Education

Clinical Reasoning

Documentation

Handoff

Presentation

Other:

Type and level of learner:

Case Objectives: please list specific objectives for each of the domains you have checked above:

1. Obtain a history and evaluate the patient
2. Perform an appropriate physical examination.
3. Arrive at a likely diagnosis for the patient’s back pain
4. Explore the possibility of aberrant drug-related behavior related to the request for an early refill of prescribed opioid analgesic

| SETTING: outpatient, in patient, ED, home, nursing home, rehab, group etc. | Outpatient |
| --- | --- |
| PATIENT PROFILE: Information about the “patient” that helps select an SP and helps the learner get an understanding of them as a person. SP will know more information about the patient than learner will ever ask but allows SP to portray a fully developed patient personality. If none of the items below are particulars for the case please write “all may be used.” | |
| Age range | 39-40 years old |
| Religious/spiritual background |  |
| Sex (e.g., male, female, intersex, transwoman, transman) | Male |
| Sexual Orientation (e.g., heterosexual, lesbian, gay, bisexual, pansexual, queer, asexual) | Heterosexual |
| Gender expression (e.g., man, woman, gender queer) | Man |
| Race/ethnicity: | Any |
| Physical description (e.g., BMI, height range) | Normal BMI |
| Physical limitations | Chronic Back Pain |
| Patient appearance (e.g., disheveled, hospital gown, business casual, casual) | Healthy/good physical condition. Professional/business attire (suit, blazer, tie). Well-groomed |
| Moulage + location (e.g., none, bruises, scars, body piercing, tattoos) | None |
| Affect (e.g., pleasant, cooperative) | Assertive, confident, rushed and somewhat fidgety as you are anxious to finish with the MD and get to your next meeting. |
| Family group (e.g., who is family, who they live with) | Lives alone.  Engaged to be married for 7 years to Tina (your fiancé works on Wall Street you are both very busy and committed to your work). |
| Education | College Graduate – Finance/Business Management |
| Level of health literacy | Proficient |
| Employment, if any - present and past, noting any current stresses | Employed as a hedge fund manager (states that he/she is very “successful”) for the past 10 years. This is your focus right now and they have little time for anything else. |
| Home/homeless - type of dwelling, number of stories, owned or rented | “Expensive” Co-op in city – owned. |
| Financial situation- any current stresses | Very successful businessman, no financial concerns/stresses |
| Insurance Status (e.g., un/under/insured, public/private, HMO/PPO) | Private insurance through company |
| Habits (i.e., diet, exercise, caffeine, smoking, alcohol, drugs) | Tobacco use (2 packs/day x 19 years) has tried to quit many times but cannot do it.  Alcohol consumption (between 2-5 drinks in an average day x 15 years) – usually wine with dinner (will finish a bottle) but will occasionally have a “night cap” of something stronger if you need to relax. This really helps you relax due to your job stress.  Recreational Marijuana use (via smoking ~3-4 times/wk.) Have been using marijuana for the past 10-15 years. Initially it was more social – 2-3 times/month, however it has steadily increased for the past 2 years you find yourself smoking it 3-4 times per week. Recreational Cocaine use (via snorting “socially” ~ 2 times/month) last use 2 days ago – your Cocaine usage began about 5 years ago.  Periodic insomnia treated with Ambien as needed.  Diet: healthy, balanced diet  Exercise : Gym 3-4 visits/wk. (Will take it easy if back is bothering  them but usually once their workout is over, their back is feeling  better). |
| Activities (i.e., hobbies, sports, clubs, friends) | None – “No time!” |
| Typical day - what is the usual daily routine | Up early – especially on days I go to the gym. Off to work, I work long hours. Usually home between 7-8 pm and have dinner. High amount of work related stress. Work long hours and travel often. |

| CASE INFORMATION | |
| --- | --- |
| Chief Concern: What the patient will say when greeted by the student. The patient’s primary reason for seeking medical care often stated in his/own words. | “I need a refill for my prescription pain medicine. For the past 5 years, I’m doing fine with my back pain on this medication but if I run out there is going to be a problem.” |
| Additional Concerns: Other, if any, concerns the patient has today (i.e., symptoms, requests, expectations, etc.) that will become part of set agenda. | Assertive, manipulative behavior just shy to the point of being pushy and overanxious. The patient *wants to be believed*, but also *wants to leave with an early refill* of his prescription pain medication. |
|  | |
| THE PATIENT STORY: The SP will be asked to tell their symptom story and the personal and emotion impact for each of their concerns. You will want to write this is the patient voice. The symptom story should be able to answer this question: “Tell me more about [chief concern/additional concern], starting at the beginning and bringing me up to now.”  The personal context should be able to answer questions concerning the broader personal/psychosocial context of symptoms, especially the patient beliefs/attributions.  The emotional context should be able to ask how are you doing with this, how does this make you feel, how has this affected you emotionally? IMPACT: How has this affected your life? How has this been for your family? | You are a 39-year-old who has been suffering with chronic back pain for the past 5 years. You are not new to this practice but the physician you are seeing today is new to you and this practice. Your main concern today is to obtain an early refill of your opioid pain medication (Oxycodone). You are feeling rushed and anxious as you want to make this quick as you have an important meeting you need to get to.  The story you will initially tell the new doctor is:  That you are usually very careful traveling with your medication, however, on a recent business trip (4 days ago) you were forced to check your bag at the last minute due to lack of overheard luggage space and they lost your luggage. You normally never check your luggage but you were late for your connecting flight and had no choice. You needed to make this flight and could not change your plans or you would have missed a very important business meeting. You will say that you were so flustered and aggravated by the situation that you never thought to take your medication out of the luggage before they took it. Now you just need the refill and don’t really have time for this appointment as you have another important meeting today, but you have no choice as you need your medication. The medication is especially important to get today as your next long-distance business trip is just 2 days away and you are going to need the medication to be able to tolerate the pain during the flight and make it through the meeting.  If the student asks how you have been managing your pain for the last 4 days without your medication you will respond: Luckily, I had a few doses at home, so I did not lose all of it in my luggage.  However, the truth of the situation is that you had developed a good relationship with your prior physician and are concerned about this “visit” today with this new doctor for a variety of reasons:   1. You have been taking the pain medication more frequently or in higher doses than prescribed, which has caused you to run out early. You are only supposed to take 1 Oxycodone every 12 hours however, you find some days you need 3 to get by. You only do this once or maybe twice a week but will not share this information unless asked if you are taking your medication as prescribed. If asked about how you are taking your medication, you will respond vaguely at first – for example:   If you are asked how much pain medication you are taking, you will respond with:  “The doctor prescribed 1 tablet every 12 hours.”  If asked if you are taking as prescribed or extra doses, you will respond with:  “Yes, when I need to.” (see checklist item #5)  If they follow up and ask additional questions regarding how much you are taking you will give them the information about the extra doses as above.   1. You have found that your social use of marijuana and cocaine, which have typically helped deal with your pain has gotten a little out of control lately. This will only be revealed if they ask you about recreational or illicit drug use. 2. You have been “borrowing” the opioid medication (a long-acting hydrocodone) from your fiancé which has now run out. You took the last does of this yesterday. This will only be revealed if you are asked if you are taking any other opioid pain or other prescription medications than the medications prescribed to you. 3. If they ask if you take any other pain medications, you can say you occasionally take Advil (2 tabs) or Tylenol (2 tabs) as needed – you take one of these 4-5 times a week. |
| HISTORY OF PRESENT ILLNESS: Although some of the HPI will be given in the patient’s symptom story, the learners will expand the story during the direct question section. Below describe the detailed history, usually about the chief concern, which the student must develop in order to make a useful assessment of the problem: | |
|  | |
| Onset (when; gradual or sudden) | Your pain has always been in your lower back and varies significantly from day to day. Started after you slipped and fell in your shower at home. |
| Setting (what was going on or where was patient when symptoms first noticed?) | Your understanding is that your original back pain problem was caused by either bulging discs (identified in an MRI done 5 years ago) in your lower spine, or muscle spasms in your lower back. You have been offered injections, other interventional procedures, and physical therapy to treat the pain instead of opioids, but you really don’t have time for this – you need to be able to get your prescription so you can get on to your next meeting. |
| Duration (how long) | 5 years. |
| Time relationships (frequency, constant or intermittent) | Depending on your use of your prescribed medication, along with marijuana and cocaine, your pain is usually around a 4-5 (0-10, 0 being no pain and 10 being pain as bad as it can be) on average, but you seem to be losing ground taking more than prescribed, and even borrowing a different opioid from your fiancé to keep it at an 8 at best. |
| Location | Lower back. Your right lower back usually bothers you more than your left, and you can usually point to a spot on just to the right of your lower spine about 2-3 inches above your buttocks where it is most tender. |
| Radiation | None |
| Quality | Aching |
| Amount | The current pain as an 8 on a numerical pain rating scale of 0 to 10 (O being no pain at all and 10 being the worst pain imaginable). You took your last Oxycodone at 8 am this morning. |
| Aggravated by what | Prolonged periods of sitting, and their occupation involves frequent air travel |
| Relieved by what | The opioids help |
| Associated with what |  |
| Attitude (what does the patient think is the problem, and how does he/she feel about it) | You want to convey to the Doctor that your pain management is very stable and that you are able to control your back pain with this medication. However, if you cannot get a refill you fear that it will become very difficult for you to manage your pain  The more time that goes by, the more ground you seem to be losing, and are very concerned that balancing the need for your medication, the demand of your high-pressure job, the stress is going to bring everything crashing down. You know that the Doctor may be reluctant to prescribe and early refill and plan to convince him/her to provide it by stressing the demands of your job and upcoming travel |
| Overall course |  |
| REVIEW OF SYSTEMS: Significant positives and negatives | |
| Aching, non-radiating lower back pain (worse on right lower back) |  |
| No radiation of pain |  |
| No leg weakness |  |
| No leg numbness |  |
|  | |
| Past medical history |  |
| Medication allergies (Name and reaction) | None |
| Environmental allergies (Name and reaction) | None |
| Illnesses | Chronic low back pain for 5 years. |
| Vaccinations | Up to date. |
| Surgeries | None. |
| Accidents/ injuries/ trauma | Chronic back pain after falling in shower 5 years ago. |
| Hospitalization | None. |
|  | |
| Inclusive sexual and reproductive history | |
| Sexual practices  Sexual partners  Protection: Use of safer sex practices  Use of birth control if appropriate  Risk of intimate partner violence | Monogamous with finance (Tina) for past 10 years. Tina has an IUD, you do not use condoms. |
| Ob/GYN HISTORY | Age of onset of menses Not applicable – male patient  Age of menopause  Number of pregnancies  Number of live births  Number of miscarriages  Number of abortions |
| Medications | Prescription/dose/reason  Oxycodone 60 mg tablet (by mouth) every 12 hours (this is how it is prescribed). However, you sometimes (maybe 2-3 times a week) you will take a third one to help you “get by” for back pain.  Ambien 10 mg pill by mouth at bedtime as needed (Take this 4-5 nights a week) for sleep.  Over the counter/dose/reason  Advil (2 tabs) or Tylenol (2 tabs) as needed – take one of these 4-5 times a week for back pain.  Herbs/supplements/dose/reason  Other: You have also on occasion taken opioid medication (a long-acting hydrocodone) that was prescribed for your fiancé |
| Immunizations | - Tetanus - Flu - Hepatitis - Pneumovax - HPV - Other |
| Tobacco products:  Cigarettes   - Cigar - Pipe - Chew - E-cigarettes | - Never - Past- year started/year quit   Current   - - Quantity- 2 packs per day   - # of years – 19 years |
| Alcohol   - Beer   Wine  Liquor   - Other | - Never - Past- year started/year quit   Current   - - Quantity 2-5 drinks per day   - # of years 10-12 years |
| Drugs  Weed  Cocaine   - Heroin - Meth - Other - IV - Inhalants - Other | - Never - Past- year started/year quit   Current   - - Quantity - # of years   Recreational Marijuana use (via smoking ~3-4 times/wk.) Have been using marijuana for the past 10-15 years. Initially it was more social – 2-3 times/month, however it has steadily increased for the past 2 years you find yourself smoking it 3-4 times per week. Recreational Cocaine use (via snorting “socially” ~ 2 times/month) last use 2 days ago – your Cocaine usage began about 5 years ago. |
| Diet (describe) | Healthy, balanced |
| Exercise (describe) | Gym 3-4 visits/wk. (Will take it easy if back is bothering  them but usually once their workout is over, their back is feeling  better). |
| List any other important social history or information important to this case | High stress, work-related  Significant amount of long-distance air travel |
| Family history |  |
| Mother, Father, Siblings, Grandparents, and other significant findings. | Father Hx of COPD, Hypertension, Alcohol abuse. Deceased at75  Mother Hx of Uterine Cancer, Deceased at 71  Siblings None  Children None  Other Blood Relatives Not significant. |
|  |  |
| Physical Exam- List exam maneuvers expected for this case and any abnormal findings that SP will simulate. (tenderness, hyper-hypo reflex, rebound, weakness etc. )   1. Location of the pain: You can stand up and indicate an area on your back just to the right of your lower spine about 2-3 inches above your buttocks where it is most tender 2. “Range of motion” of your back: May ask you to stand up and bend forward, and touch your toes (or as close as you can), which you can do but it does cause some increased pain in your back (mainly in the area you just referred to above). 3. Balance: May ask you to stand on one leg and then the other – you can do this without any trouble and both legs have equal strength and no pain when you do this. It also does not increase your back pain when you do this. | |
| PHYSICAL EXAM FINDINGS |  |
| 1. Written in layman’s terms | See above. |
| 1. General appearance- affect, appearance, position of patient at opening (i.e. sitting, laying down, holding abdomen etc.) | Seated in street clothes (professional business attire). |
| 1. Vital signs | Temperature 98.6  Blood Pressure 130/85  Pulse 85  Respiration 14 |
| 1. Specific findings and affect |  |
| 1. Response to certain physical movements | May ask you to stand up and bend forward, and touch your toes (or as close as you can), which you can do but it does cause some increased pain in your back  May ask you to stand on one leg and then the other – you can do this without any trouble and both legs have equal strength and no pain when you do this. It also does not increase your back pain when you do this. |
|  |  |
| DIAGNOSIS AND DIFFERENTIAL |  |
| Diagnosis with support from positive and negative history and PE findings | Chronic back pain for 5 years duration. Discogenic findings confirmed by MRI 5 years ago. History and physical exam consistent with chief complaint. |
| Differential with support from positive and negative history and PE findings | R/O Discogenic back pain  R/O Musculoskeletal pain  R/O Medication-seeking behavior  R/O Aberrant Drug-related behavior  R/O Medication-seeking behavior |
|  |  |
| MANAGEMENT OR DIAGNOSTIC PLAN | - Urine drug screen - Diagnostic imaging at a later date - Discussion at a later date regarding opioid tapering - Reinforcement of the importance of safe storage of controlled substances - Reinforcement of the importance of adhering to the prescription regimen - Encourage decreasing ETOH consumption |
|  |  |
| PROFESSIONALISM ISSUES OR CHALLENGES: | - Patient request for an early refill of opioid medication could be considered to be a “red-flag” for unhealthy drug use - Safe and appropriate prescribing of opioids and meeting patient’s needs - Fear of regulatory scrutiny |
