# Supplementary material for: Early Refill of an Opioid Medication: Recognizing Personal Biases Through Clinical Vignettes and OSCEs
Source: MedEdPORTAL. 2022 Apr 7;18:11234. doi: 10.15766/mep_2374-8265.11234 (PMC8986891; doi:10.15766/mep_2374-8265.11234)
Supplement: Supplementary file 1 — MS 1 Clinical Vignettes & Follow-Up.pptxMS 1 Debrief.pptxSP James Spiegel - Case 1.docxSP Darryl Whitcomb - Case 2.docxSP Helen Morgan - Case 3.docxDoor Notes.docxLogistical Flow.docxFaculty Post-OSCE Debrief Discussion Guide.docxSP Encounter Checklist.docxSP Responses for Checklist Items.docxMS 3 Post-OSCE Survey.docx [file mep_2374-8265.11234-s001.zip › D. SP Darryl Whitcomb - Case 2.docx]

Appendix D. Standardized Patient Darryl Whitcomb Case

Date: 1/05/21

Primary Case Author: Kevin L. Zacharoff, MD, FACIP, FACPE, FAAP

Secondary Case Author: Perrilynn Baldelli, DNP, RN, CHSE

Standardized Patient Educator: Denise Antonelle-Mahoney, MS

Name of Case: Request for Early Refill of an Opioid Medication

Name of educational and or assessment activity: Transitions to Clinical Care (TCC) OSCE Case

Patient Name: Darryl Whitcomb

Chief Complaint: Chronic back pain, requesting an early refill of prescription opioid medication

Most likely Diagnosis and Differential with rationale from history and/or physical exam:

R/O Discogenic back pain

R/O Musculoskeletal pain

R/O Medication-seeking behavior

R/O Aberrant Drug-related behavior

Challenge question: None

Domains: Check all that apply

Professionalism

Communication and Interpersonal skills

Medical History

☒ Physical exam

Shared Decision Making

☒ Patient Education

Clinical Reasoning

Documentation

Handoff

Presentation

Other:

Type and level of learner:

Case Objectives: please list specific objectives for each of the domains you have checked above:

1. Obtain a history and evaluate the patient
2. Perform an appropriate physical examination.
3. Arrive at a likely diagnosis for the patient’s back pain
4. Explore the possibility of aberrant drug-related behavior related to the request for an early refill of prescribed opioid analgesic

| SETTING: outpatient, in patient, ED, home, nursing home, rehab, group etc. | Outpatient |
| --- | --- |
| PATIENT PROFILE: Information about the “patient” that helps select an SP and helps the learner get an understanding of them as a person. SP will know more information about the patient than learner will ever ask but allows SP to portray a fully developed patient personality. If none of the items below are particulars for the case please write “all may be used.” | |
| Age range | 54- 58years old |
| Religious/spiritual background |  |
| Sex (e.g., male, female, intersex, transwoman, transman) | Male |
| Sexual Orientation (e.g., heterosexual, lesbian, gay, bisexual, pansexual, queer, asexual) | Heterosexual |
| Gender expression (e.g., man, woman, gender queer) | Man |
| Race/ethnicity: | Any |
| Physical description (e.g., BMI, height range) | Normal BMI |
| Physical limitations | Chronic Back Pain |
| Patient appearance (e.g., disheveled, hospital gown, business casual, casual) | Sloppily dressed, poorly manicured beard and/or ponytail. Biker bandana on his head. |
| Moulage + location (e.g., none, bruises, scars, body piercing, tattoos) | Can add tattoos |
| Affect (e.g., pleasant, cooperative) | Assertive, outspoken and blunt. Cooperative and willing to answer questions. |
| Family group (e.g., who is family, who they live with) | Divorced for 10 years. Currently girlfriend and her 2 children just moved in with you for the next 2 years. |
| Education | High School Diploma |
| Level of health literacy | Basic |
| Employment, if any - present and past, noting any current stresses | Currently unemployed; sporadically employed, when you can find “odd jobs” in construction and your back pain permits. |
| Home/homeless - type of dwelling, number of stories, owned or rented | Owns a small house in an average neighborhood. |
| Financial situation- any current stresses | Money is “tight” as it usually is. Waiting for your next “odd” job but you don’t stress about it. |
| Insurance Status (e.g., un/under/insured, public/private, HMO/PPO) | Medicaid |
| Habits (i.e., diet, exercise, caffeine, smoking, alcohol, drugs) | Tobacco use (2 packs/day x 40 years) – Not going to quit, “I’ve been smoking since I was a teenager”  Alcohol consumption (between 3-6 beers in per day for many years).  No illicit/recreational drug use.  Diet: not healthy – pancakes for breakfast, likes fast food for lunch (especially pizza) and meat and potatoes for dinner.  Exercise : None |
| Activities (i.e., hobbies, sports, clubs, friends) | Riding his motorcycle |
| Typical day - what is the usual daily routine | Depends on the day. If I have work and my back is feeling good, I am out the door early after breakfast. Otherwise, I sleep in alittle and then have breakfast. If I’m not working, I enjoy a ride on my motorcycle and out to lunch. Usually home for dinner with girlfriend and her kids. |

| CASE INFORMATION | |
| --- | --- |
| Chief Concern: What the patient will say when greeted by the student. The patient’s primary reason for seeking medical care often stated in his/own words. | “I can’t find my pain meds and it’s been total hell for me without them.” |
| Additional Concerns: Other, if any, concerns the patient has today (i.e., symptoms, requests, expectations, etc.) that will become part of set agenda. | Lackadaisical, laid-back behavior just shy of not taking things seriously including responsible use/storage of his opioid medication. The patient portrays *a somewhat indignant* attitude, but also *wants to leave with an early refill* of his prescription pain medication. |
|  | |
| THE PATIENT STORY: The SP will be asked to tell their symptom story and the personal and emotion impact for each of their concerns. You will want to write this is the patient voice. The symptom story should be able to answer this question: “Tell me more about [chief concern/additional concern], starting at the beginning and bringing me up to now.”  The personal context should be able to answer questions concerning the broader personal/psychosocial context of symptoms, especially the patient beliefs/attributions.  The emotional context should be able to ask how are you doing with this, how does this make you feel, how has this affected you emotionally? IMPACT: How has this affected your life? How has this been for your family? | You are a 56-year-old who has been suffering with chronic back and neck pain for the past 7 years. You are not new to this practice but the physician you are seeing today is new to you and this practice. Your main concern today is to obtain an early refill of your opioid pain medication (Oxycodone). You are feeling relaxed and matter of fact about this, and not worried at all that your early refill request will be denied.  The story you will initially tell the new doctor is:  That your girlfriend recently (5 days ago) just moved into your house with her kids (for about the next 2 years) and even though it’s not normally neat and orderly, “The place looks like a hurricane hit it”. The problem is that right now you cannot find your prescription pain medication – it seems to be “lost”. You tried to get an earlier appointment, as the pain started to get worse after missing just one dose. You are a bit frustrated that it took you 3 days to get an appointment! You want to stress to the Doctor that it has “been hell for you”, and you need a refill for your opioid medication “like yesterday” because your pain is now unbearable, making it difficult to ride your motorcycle, which is your only form of transportation.  If the student asks how you have been managing your pain for the last 4 days without your medication you will respond: “A lot of beer and cigarettes.”  You had developed a good relationship with your prior physician (who is now retired) and are not at all concerned about this “visit” today with this new doctor for a variety of reasons:   1. You have been taking the prescription pain medication as prescribed, and don’t feel that your request is unusual. If asked about how you are taking your medication, you will respond succinctly – for example:   If you are asked how much pain medication you are taking, you will respond with:  “The same way I have for the past 7 years.”  If asked if you are taking as prescribed or extra doses, you will respond with:  “Nope.”   1. You never used illicit substances such as marijuana or cocaine. This will only be revealed if they ask you about recreational or illicit drug use. 2. You have never “borrowed” anyone else’s prescription pain medication. This will only be revealed if you are asked if you are taking any other opioid pain or other prescription medications than the medications prescribed to you. 3. If they ask if you take any other pain medications, you can say you occasionally take Advil (2 tabs) or Tylenol (2 tabs) – maybe 1-2 times a week. However, for the past 5 days you have been taking both Advil and Tylenol a couple of times a day to help with the pain.   You convey to the Doctor that your pain control is normally tolerable on your medication, but you have been miserable for the past 4 days. |
| HISTORY OF PRESENT ILLNESS: Although some of the HPI will be given in the patient’s symptom story, the learners will expand the story during the direct question section. Below describe the detailed history, usually about the chief concern, which the student must develop in order to make a useful assessment of the problem: | |
|  | |
| Onset (when; gradual or sudden) | Your pain has always been generalized in your neck and spine and varies from day to day. Your neck is especially stiff in the morning on awakening, your back hurts pretty much all of the time, and you point to the base of your neck and down the back of the entire spine when prompted about the location of your pain. |
| Setting (what was going on or where was patient when symptoms first noticed?) | This pain started after you were involved in a motorcycle accident. Originally, your understanding is that no one has come to a specific conclusion about the cause of your neck and back pain (evaluated with neck and spine MRI and X-ray studies). You have been offered injections, other interventional procedures, and physical therapy to treat the pain instead of opioids, but you “ain’t letting anyone stick needles in you” and think the other options are “baloney”. |
| Duration (how long) | 7 years. |
| Time relationships (frequency, constant or intermittent) | You have pain every day and pretty much all of the time. |
| Location | Entire spine and the base of your neck. |
| Radiation | None |
| Quality | Aching. |
| Amount | The average pain over the past month has been 4-5 on some days, and 8-9 other days on a numerical pain rating scale of 0 to 10 (O being no pain at all and 10 being the worst pain imaginable).  . |
| Aggravated by what | The pain is aggravated by weather, high levels of physical activity, and sudden turning of the neck in either direction. |
| Relieved by what | The opioids help |
| Associated with what |  |
| Attitude (what does the patient think is the problem, and how does he/she feel about it) | You want to convey to the Doctor that your pain management is very stable and that you are able to control your back pain with this medication. “Doc, this just boils down to one thing for me, and that is, since my accident the only thing that matters is that I have my medication. I just don’t know what I would do without it. I hear all that mumbo-jumbo you’re saying, but I have to tell you, that’s the deal.”  You “want to get out of the Doctor’s hair” and get back to helping with the unpacking to get back to “the normal level of chaos”. |
| Overall course |  |
| REVIEW OF SYSTEMS: Significant positives and negatives | |
| Aching, non-radiating neck, upper, and lower back pain |  |
| No radiation of pain |  |
| No leg weakness |  |
| No leg numbness |  |
|  | |
| Past medical history |  |
| Medication allergies (Name and reaction) | None |
| Environmental allergies (Name and reaction) | None |
| Illnesses | Chronic neck and back pain for 7 years.  Hypertension for 15 years  Elevated liver enzymes (reason unknown)  Frequent urinary tract infections |
| Vaccinations | Up to date including flu shot. |
| Surgeries | None. |
| Accidents/ injuries/ trauma | Two prior hospitalizations for concussions (overnight stays). One involving a “bar fight” and the other was a construction job-related injury.  Motorcycle accident 7 years ago – started neck and back pain but not hospitalized. |
| Hospitalization | Two concussions (see above). |
|  | |
| Inclusive sexual and reproductive history | |
| Sexual practices  Sexual partners  Protection: Use of safer sex practices  Use of birth control if appropriate  Risk of intimate partner violence | Monogamous with girlfriend (Annette) for past 5 years. Annette has an IUD, you do not use condoms. |
| Ob/GYN HISTORY | Age of onset of menses Not applicable – male patient  Age of menopause  Number of pregnancies  Number of live births  Number of miscarriages  Number of abortions |
| Medications | Prescription/dose/reason  Oxycodone 60 mg tablet (by mouth) every 12 hours (this is how it is prescribed). You take this only as prescribed.  Over the counter/dose/reason  Advil (2 tabs) or Tylenol (2 tabs) as needed – take one of these 1-2 times a week for back/neck pain.  Hydrochlorothiazide (HCTZ) [50mg (by mouth) once a day for high blood pressure].  Atenolol [25mg (by mouth) twice a day for high blood pressure]. |
| Immunizations | - Tetanus   X Flu   - Hepatitis - Pneumovax - HPV - Other |
| Tobacco products:  Cigarettes   - Cigar - Pipe - Chew - E-cigarettes | - Never - Past- year started/year quit   Current   - - Quantity- 2 packs per day   - # of years – 40 years |
| Alcohol  Beer  Wine   - Liquor - Other | - Never - Past- year started/year quit   Current   - - Quantity 3-6 beers per day   - # of years – for “many” years |
| Drugs  Weed  Cocaine   - Heroin - Meth - Other - IV - Inhalants - Other | Never   - Past- year started/year quit   Current   - - Quantity - # of years |
| Diet (describe) | Diet: not healthy – pancakes for breakfast, likes fast food for lunch (especially pizza) and meat and potatoes for dinner. |
| Exercise (describe) | None. |
| List any other important social history or information important to this case | Divorced for 10 years  Girlfriend (48 y/o) and 2 children (16 & 18) just moved in with you for the next two years.  Children (from marriage): 5 Kids: 2 Boys (Andy age 36 and Roy age 30) and 3 Girls (Stacy age 34, Michelle age 32, and Ashley age 28) |
| Family history |  |
| Mother, Father, Siblings, Grandparents, and other significant findings. | Father: Hx of COPD, Hypertension, Alcohol abuse. Deceased at  75 natural cases  Mother: Hx of Uterine Cancer, Hypertension. Deceased at 71 natural causes.  Siblings: Brother, medical history unknown (has not had contact in over 20 years)  Children:5 (2 boys, 3 girls), no significant medical history  Other Blood Relatives Not significant. |
|  |  |
| Physical Exam- List exam maneuvers expected for this case and any abnormal findings that SP will simulate. (tenderness, hyper-hypo reflex, rebound, weakness etc. )   1. Location of the pain: You can stand up and indicate an area from the middle portion of the base of the neck and entire length of the back 2. “Range of motion” of your neck: May ask you to look as far as you can to the left, and then to the right 3. Ask to check the “range of motion” of your back – ask you to stand up and bend forward, and touch your toes (or as close as you can attempt), which you can do but it does cause some increased pain in your back (mainly in the area you just referred to above) 4. Balance: May ask you to stand on one leg and then the other – you can do this without any trouble and both legs have equal strength and no pain when you do this. It also does not increase your back pain when you do this. | |
| PHYSICAL EXAM FINDINGS |  |
| 1. Written in layman’s terms | See above. |
| 1. General appearance- affect, appearance, position of patient at opening (i.e. sitting, laying down, holding abdomen etc.) | Seated in street clothes (sloppily dressed – tee shirt, jeans with poorly manicured appearance – beard and/or ponytail. Biker bandana on head, may have tattoos). |
| 1. Vital signs | Temperature 98.4  Blood Pressure 140/90  Pulse 90  Respiration 12 |
| 1. Specific findings and affect |  |
| 1. Response to certain physical movements | May ask you to stand up and bend forward, and touch your toes (or as close as you can attempt), which you can do but it does cause some increased pain in your back (mainly in the area you just referred to above)  May ask you to stand on one leg and then the other – you can do this without any trouble and both legs have equal strength and no pain when you do this. It also does not increase your back pain when you do this. |
|  |  |
| DIAGNOSIS AND DIFFERENTIAL |  |
| Diagnosis with support from positive and negative history and PE findings | Chronic back pain for 7 years duration. Radiologic testing 7 years ago inconclusive. History and physical exam consistent with chief complaint. |
| Differential with support from positive and negative history and PE findings | R/O Discogenic back pain  R/O Musculoskeletal pain  R/O Medication-seeking behavior  R/O Aberrant Drug-related behavior  R/O Medication-seeking behavior |
|  |  |
| MANAGEMENT OR DIAGNOSTIC PLAN | - Urine drug screen - Diagnostic imaging to be considered at a later date - Discussion at a later date regarding opioid tapering - Reinforcement of the importance of safe storage of controlled substances - Reinforcement of the importance of adhering to the prescription regimen - Encourage decreasing ETOH consumption |
|  |  |
| PROFESSIONALISM ISSUES OR CHALLENGES: | - Patient request for an early refill of opioid medication could be considered to be a “red-flag” for unhealthy drug use - Safe and appropriate prescribing of opioids and meeting patient’s needs - Fear of regulatory scrutiny |
