# Supplementary material for: Early Refill of an Opioid Medication: Recognizing Personal Biases Through Clinical Vignettes and OSCEs
Source: MedEdPORTAL. 2022 Apr 7;18:11234. doi: 10.15766/mep_2374-8265.11234 (PMC8986891; doi:10.15766/mep_2374-8265.11234)
Supplement: Supplementary file 1 — MS 1 Clinical Vignettes & Follow-Up.pptxMS 1 Debrief.pptxSP James Spiegel - Case 1.docxSP Darryl Whitcomb - Case 2.docxSP Helen Morgan - Case 3.docxDoor Notes.docxLogistical Flow.docxFaculty Post-OSCE Debrief Discussion Guide.docxSP Encounter Checklist.docxSP Responses for Checklist Items.docxMS 3 Post-OSCE Survey.docx [file mep_2374-8265.11234-s001.zip › E. SP Helen Morgan - Case 3.docx]

Appendix E. Standardized Patient Helen Morgan Case

Date: 1/05/21

Primary Case Author: Kevin L. Zacharoff, MD, FACIP, FACPE, FAAP

Secondary Case Author: Perrilynn Baldelli, DNP, RN, CHSE

Standardized Patient Educator: Denise Antonelle-Mahoney, MS

Name of Case: Request for Early Refill of an Opioid Medication

Name of educational and or assessment activity: Transitions to Clinical Care (TCC) OSCE Case

Patient Name: Helen Morgan

Chief Complaint: Chronic back pain, requesting an early refill of prescription opioid medication

Most likely Diagnosis and Differential with rationale from history and/or physical exam:

R/O Spinal stenosis vs Discogenic back pain

R/O Osteoarthritis – related back pain

R/O Medication-seeking behavior

R/O Aberrant Drug-related behavior

Challenge question: None

Domains: Check all that apply

Professionalism

Communication and Interpersonal skills

Medical History

☒ Physical exam

Shared Decision Making

☒ Patient Education

Clinical Reasoning

Documentation

Handoff

Presentation

Other:

Type and level of learner:

Case Objectives: please list specific objectives for each of the domains you have checked above:

1. Obtain a history and evaluate the patient
2. Perform an appropriate physical examination.
3. Arrive at a likely diagnosis for the patient’s back pain
4. Explore the possibility of aberrant drug-related behavior related to the request for an early refill of prescribed opioid analgesic

| SETTING: outpatient, in patient, ED, home, nursing home, rehab, group etc. | Outpatient |
| --- | --- |
| PATIENT PROFILE: Information about the “patient” that helps select an SP and helps the learner get an understanding of them as a person. SP will know more information about the patient than learner will ever ask but allows SP to portray a fully developed patient personality. If none of the items below are particulars for the case please write “all may be used.” | |
| Age range | 75 - 78years old |
| Religious/spiritual background |  |
| Sex (e.g., male, female, intersex, transwoman, transman) | Female |
| Sexual Orientation (e.g., heterosexual, lesbian, gay, bisexual, pansexual, queer, asexual) | Heterosexual |
| Gender expression (e.g., man, woman, gender queer) | woman |
| Race/ethnicity: | Any |
| Physical description (e.g., BMI, height range) | Normal BMI |
| Physical limitations | Chronic Back Pain |
| Patient appearance (e.g., disheveled, hospital gown, business casual, casual) | Immaculately dressed, Wearing makeup, earrings, pearl necklace, (or any costume jewelry) and “granny glasses,” etc. (Do not need to wear glasses if you usually do not wear them) . “Salon-level” hairdo |
| Moulage + location (e.g., none, bruises, scars, body piercing, tattoos) | None |
| Affect (e.g., pleasant, cooperative) | Almost meek/shy. Soft-spoken, articulate  A patient, focused listener who nods with most comments by the Doctor |
| Family group (e.g., who is family, who they live with) | Widowed 16 years ago after husband of 41 years died suddenly of heart attack. Lives alone in small apartment. |
| Education | College Graduate |
| Level of health literacy | Proficient. |
| Employment, if any - present and past, noting any current stresses | Retired high school librarian. Retired at age 70 after working for 35 years. |
| Home/homeless - type of dwelling, number of stories, owned or rented | Rents a small apartment for past 20 years. |
| Financial situation- any current stresses | Lives on fixed income of social security and her small pension. “I get by but it’s tough sometimes”. |
| Insurance Status (e.g., un/under/insured, public/private, HMO/PPO) | Has Medicare. |
| Habits (i.e., diet, exercise, caffeine, smoking, alcohol, drugs) | No tobacco use – never has.  Alcohol consumption – rare glass of wine on special occasion.  No illicit/recreational drug use.  Diet: healthy.  Exercise : Tries to walk in neighborhood a couple of times a week. |
| Activities (i.e., hobbies, sports, clubs, friends) | Reading. |
| Typical day - what is the usual daily routine | Usually get up around 8 am and have breakfast. I usually watch the news or read the newspaper after breakfast. I eat lunch around 1 pm and take a short nap. In the afternoon, I may visit with neighbors, read, take a walk or do something around the apartment or run an errand/shop. |

| CASE INFORMATION | |
| --- | --- |
| Chief Concern: What the patient will say when greeted by the student. The patient’s primary reason for seeking medical care often stated in his/own words. | “I feel so foolish about this, but since I cleaned my apartment the other day, I just can’t seem to find my pain medicine.” |
| Additional Concerns: Other, if any, concerns the patient has today (i.e., symptoms, requests, expectations, etc.) that will become part of set agenda. | Polite, mild-mannered, soft-spoken, proper, calm, and endearing. The patient portrays respectful and attentive attitude, but also wants to leave with an early *refill* of her prescription pain medication. |
|  | |
| THE PATIENT STORY: The SP will be asked to tell their symptom story and the personal and emotion impact for each of their concerns. You will want to write this is the patient voice. The symptom story should be able to answer this question: “Tell me more about [chief concern/additional concern], starting at the beginning and bringing me up to now.”  The personal context should be able to answer questions concerning the broader personal/psychosocial context of symptoms, especially the patient beliefs/attributions.  The emotional context should be able to ask how are you doing with this, how does this make you feel, how has this affected you emotionally? IMPACT: How has this affected your life? How has this been for your family? | You are a 77-year-old who has been suffering with chronic back pain for the past 15 years. You are not new to this practice but the physician you are seeing today is new to you and this practice. Your main concern today is to obtain an early refill of your opioid pain medication (Oxycodone). You are feeling a bit apprehensive and hopeful that your early refill request will be approved.  The story you will initially tell the new doctor is:  That as you get older, you try very hard to be regimented about organizing things more than you used to, and while you normally keep all your medications in one place for safe keeping, somehow after the weekly cleaning (2 days ago) of your apartment (which you do meticulously), the opioid medication is “nowhere to be found”. You stress that you live alone, are proud that you take care of all your needs and responsibilities including grocery shopping, house cleaning, etc., and you feel “very embarrassed” about the early refill request, fearing that without your pain medication, your untreated pain might leave you virtually helpless and lose your independence.  If the student asks how you have been managing your pain for the last 2 days without your medication you will respond: “I’ve been lucky that the pain just hasn’t been that bad for the past couple of days.”  You had developed a good relationship with your prior physician (who has retired) and are concerned about this “visit” today with this new doctor for a variety of reasons:   1. You have avoided taking the medication unless absolutely necessary, to stockpile it and sell it to help pay for your other prescription medications because they are so expensive.   You take the medication an average of 2 days a week. The other days you just “power” through the pain as you are trying to stockpile the medicine to sell (but you will not reveal this unless they push you and say that it does not account for the amount of medication you are prescribed versus the amount you should have left over). You do this for the extra money to help pay for your living expenses and the expensive other prescriptions you need for your other health conditions.   1. If asked about how you are taking your medication, you will respond succinctly – for example:   If you are asked how much pain medication you are taking, you will respond with:  “Exactly the same way I have for the past 15 years.”  If asked if you are ever taking extra doses, you will respond with:  “No Doctor, I would never do that.”   1. You have never used illicit substances such as marijuana or cocaine. This will only be revealed if they ask you about recreational or illicit drug use. 2. You have never “borrowed” anyone else’s prescription pain medication. This will only be revealed if you are asked if you are taking any other opioid pain or other prescription medications than the medications prescribed to you. 3. If the student asks if you take any other pain medications, you can say that you try to take as few pain medications as possible, so you almost never use over the counter medications such as Advil or Tylenol. |
| HISTORY OF PRESENT ILLNESS: Although some of the HPI will be given in the patient’s symptom story, the learners will expand the story during the direct question section. Below describe the detailed history, usually about the chief concern, which the student must develop in order to make a useful assessment of the problem: | |
|  | |
| Onset (when; gradual or sudden) | Your pain has always been generalized in your lower back and both legs and varies in intensity from day to day. You are especially stiff in the morning on awakening, your back hurts pretty much all the time, and you point to the base of your lower back when prompted about the location of your pain. |
| Setting (what was going on or where was patient when symptoms first noticed?) | Your understanding is that your back pain is due to a combination of arthritis and spinal stenosis (evaluated with spine MRI and X-ray studies) You have been offered injections, other interventional procedures, and physical therapy to treat the pain instead of opioids, but you are “Deathly terrified of needles and scared of being left paralyzed” and feel that the other options are “Not likely to succeed as my problems are age-related”. |
| Duration (how long) | 15 years. |
| Time relationships (frequency, constant or intermittent) | Your pain varies in intensity from day to day. You are especially stiff in the morning on awakening and your back hurts pretty much all of the time. |
| Location | Lower back |
| Radiation | Down both legs. |
| Quality | Aching and stiffness |
| Amount | The current pain is a 6 on a numerical pain rating scale of 0 to 10 (O being no pain at all and 10 being the worst pain imaginable)  The average pain over the past month has been 4-5 on some days, and 8-9 other days on a numerical pain rating scale of 0 to 10 (O being no pain at all and 10 being the worst pain imaginable) |
| Aggravated by what | The pain is aggravated by weather, sitting for prolonged periods of time (such as long car rides) and sudden movement of the legs or hips |
| Relieved by what | The opioids help |
| Associated with what |  |
| Attitude (what does the patient think is the problem, and how does he/she feel about it) | You convey to the Doctor that you just want to make sure that you have your pain medication on hand, and that you are fearful that without it, you might not be able to take care of yourself and retain your independence if your pain should flare and worsen and become disabling to you.  You want to portray yourself as somewhat embarrassed and worthy of sympathy as possible, without creating any fuss and leave with your early refill. |
| Overall course |  |
| REVIEW OF SYSTEMS: Significant positives and negatives | |
| Aching, lower back pain and stiffness |  |
| Pain radiates down both legs |  |
| No leg weakness |  |
| Some numbness in both feet (likely due to diabetes) |  |
|  | |
| Past medical history |  |
| Medication allergies (Name and reaction) | None |
| Environmental allergies (Name and reaction) | None |
| Illnesses | Chronic back pain for 15 years  Hypertension for 35 years  Adult-onset Diabetes for 17 years  Atrial fibrillation (A-fib) for 5 years  Osteoarthritis in fingers, hands, hips, and knees for 15 years. |
| Vaccinations | Up to date including flu shot. |
| Surgeries | Cholecystectomy (Gall Bladder removed) at 25 years of age |
| Accidents/ injuries/ trauma | None |
| Hospitalization | Cholecystectomy (Gall Bladder removed) at 25 years of age  Cardioversion for atrial fibrillation (A-fib) twice; 28 years ago, and 5 years ago. You still have A-fib even after having the Cardioversion twice. |
|  | |
| Inclusive sexual and reproductive history | |
| Sexual practices  Sexual partners  Protection: Use of safer sex practices  Use of birth control if appropriate  Risk of intimate partner violence | Abstinent since husband’s passing. |
| Ob/GYN HISTORY | Age of onset of menses 14  Age of menopause 54  Number of pregnancies 1  Number of live births 1  Number of miscarriages 0  Number of abortions 0 |
| Medications | Prescription/dose/reason  Oxycodone [30 mg tablet (by mouth) every 12 hours (this is how it is prescribed). You are taking this medication only when you need it (you will only reveal this if they ask you how often you take it). If they ask this you will respond that you take it about 2 days a week.]  Note: if they ask are you taking it as prescribed the response will be: “Exactly the same way I have for the past 15 years.”  Hydrochlorothiazide (HCTZ) [50mg (by mouth) once a day for high blood pressure].  Atenolol [25mg (by mouth) twice a day for high blood pressure].  Metformin [500mg (by mouth) twice a day for adult-onset diabetes]. Last A1C 6.2. Check fingerstick once a day in the evening and it is usually around 130.  Eliquis [5 mg (by mouth) twice a day for atrial fibrillation]. (Expensive Blood Thinner medication) |
| Immunizations | - Tetanus   X Flu   - Hepatitis - Pneumovax - HPV - Other |
| Tobacco products:  Cigarettes   - Cigar - Pipe - Chew - E-cigarettes | Never   - Past- year started/year quit   Current   - - Quantity   - # of years |
| Alcohol  Beer  Wine   - Liquor - Other | - Never - Past- year started/year quit   Current   - - Quantity – rare glass of wine at special occasion   - # of years |
| Drugs  Weed  Cocaine   - Heroin - Meth - Other - IV - Inhalants - Other | Never   - Past- year started/year quit   Current   - - Quantity - # of years |
| Diet (describe) | Diet: healthy – tea and cereal for breakfast, sandwich and fruit for lunch and chicken or pasta with vegetables for dinner. “I stay away from red meat”. |
| Exercise (describe) | None. |
| List any other important social history or information important to this case | Important for patient to remain “independent”. She prides herself on being able to care for herself and this is very important to her. |
| Family history |  |
| Mother, Father, Siblings, Grandparents, and other significant findings. | Father: Hx of COPD, Hypertension, Alcohol abuse. Deceased at  75 natural cases  Mother: Hx of Uterine Cancer, Hypertension. Deceased at 71 natural causes.  Siblings: Brother with hypertension, coronary artery disease, and died of pancreatic cancer at age 58.  Children: 1 daughter age 51, no significant medical history  Other Blood Relatives Not significant. |
|  |  |
| Physical Exam- List exam maneuvers expected for this case and any abnormal findings that SP will simulate. (tenderness, hyper-hypo reflex, rebound, weakness etc. )   1. Location of the pain: You can stand up and indicate a general area around both hips and buttocks 2. “Range of motion” of your back: May ask you to stand up and bend forward and touch your toes (or as close as you can attempt), which you can do to a very limited degree due to stiffness and age. 3. Balance: May ask you to stand on one leg and then the other – you can do this while holding onto something to help support and stabilize you (such as a chair or counter). It does not increase your back pain when you do this. | |
| PHYSICAL EXAM FINDINGS |  |
| 1. Written in layman’s terms | See above. |
| 1. General appearance- affect, appearance, position of patient at opening (i.e. sitting, laying down, holding abdomen etc.) | Seated in street clothes. Immaculately dressed, Wearing makeup, earrings, pearl necklace, (or any costume jewelry) and “granny glasses,” etc. (Do not need to wear glasses if you usually do not wear them) . “Salon-level” hairdo |
| 1. Vital signs | Temperature 97.9  Blood Pressure 148/90  Pulse 82  Respiration 12 |
| 1. Specific findings and affect |  |
| 1. Response to certain physical movements | May ask you to stand up and bend forward and touch your toes (or as close as you can attempt), which you can do to a very limited degree due to stiffness and age.  May ask you to stand on one leg and then the other – you can do this while holding onto something to help support and stabilize you (such as a chair or counter). It does not increase your back pain when you do this. |
|  |  |
| DIAGNOSIS AND DIFFERENTIAL |  |
| Diagnosis with support from positive and negative history and PE findings | Chronic back pain for 15 years duration. History and physical exam consistent with chief complaint. |
| Differential with support from positive and negative history and PE findings | R/O Spinal stenosis vs Discogenic back pain  R/O Osteoarthritis – related back pain  R/O Medication-seeking behavior  R/O Aberrant Drug-related behavior  R/O Medication-seeking behavior |
|  |  |
| MANAGEMENT OR DIAGNOSTIC PLAN | - Urine drug screen - Diagnostic imaging to be considered at a later date - Reinforcement of the importance of safe storage of controlled substances - Reinforcement of the importance of adhering to the prescription regimen |
|  |  |
| PROFESSIONALISM ISSUES OR CHALLENGES: | - Patient request for an early refill of opioid medication could be considered to be a “red-flag” for unhealthy drug use - Safe and appropriate prescribing of opioids and meeting patient’s needs - Fear of regulatory scrutiny |
