# Supplementary material for: Early Refill of an Opioid Medication: Recognizing Personal Biases Through Clinical Vignettes and OSCEs
Source: MedEdPORTAL. 2022 Apr 7;18:11234. doi: 10.15766/mep_2374-8265.11234 (PMC8986891; doi:10.15766/mep_2374-8265.11234)
Supplement: Supplementary file 1 — MS 1 Clinical Vignettes & Follow-Up.pptxMS 1 Debrief.pptxSP James Spiegel - Case 1.docxSP Darryl Whitcomb - Case 2.docxSP Helen Morgan - Case 3.docxDoor Notes.docxLogistical Flow.docxFaculty Post-OSCE Debrief Discussion Guide.docxSP Encounter Checklist.docxSP Responses for Checklist Items.docxMS 3 Post-OSCE Survey.docx [file mep_2374-8265.11234-s001.zip › I. SP Encounter Checklist.docx]

**STANDARDIZED PATIENT CHECKLIST: Request for Early Refill Case**

Please assess the learner performance during your encounter utilizing the observable behaviors listed below. Place a check in the **“YES”** column if it occurred or **“NO” if** it did not. A comment is **required** for items marked **“No”.**

Patient Case Name: _____________________ Learn Name: ________________________

| **History-Taking: The student asked** | **Yes** | **No** |
| --- | --- | --- |
| 1. Do you use any recreational drugs? OR   Do you use any illicit drugs? OR  Do you use any illegal drugs? |  |  |
| 1. Do you use any drugs not prescribed to you? OR   Do you ever use any other medications for your pain? |  |  |
| 1. Have you ever needed an early refill before? OR   Has anything ever happened like this before where you needed an early refill? OR  Do you find that you ever run out of your medication early? |  |  |
| 1. How many times have you needed an early refill? |  |  |
| 1. Have you ever taken your prescription pain medicine in a way other than prescribed? OR   Have you ever taken more prescription pain medicine other than prescribed? OR  Have you ever taken any extra doses of prescribed pain medicine? OR  Are you taking more than 1 tablet every 12 hours? |  |  |
| 1. How long has it been since someone performed testing to re-evaluate your neck/back pain? OR   When was the last time that you had any diagnostic tests (x-ray, MRI, etc.) to evaluate your neck/back pain? OR  When was the last time that someone did a “work-up” for your neck/back pain? |  |  |
| 1. Does anyone in your family have a history of substance abuse? OR   Has anyone in your family ever had a problem with alcohol or drugs? |  |  |
| **Information Sharing/Patient Education** | **Yes** | **No** |
| 1. The student instructed me that prescription medication should always be stored in a safe and secure location. |  |  |
| 1. The student reinforced that this medication should always be taken as directed. |  |  |
| 1. The student informed me that taking a controlled medication involves taking responsibility for safe storage practices. |  |  |
| **Comments:** | | |

| **Patient-Centered Communication Skills** | | |
| --- | --- | --- |
| 1. **Appropriate Greeting** | **Yes** | **No** |
| 1. **Introduced** themselves and showed ID. |  |  |
| 1. Explained their **role/title** |  |  |
| 1. Addressed the **patient by name** |  |  |
| 1. Referred to patient by asking preference (how would you like to be called) |  |  |
| 1. **Building the Relationship** | **Yes** | **No** |
| 1. **Showed interest** in the patient, as a person, not just their condition |  |  |
| 1. Demonstrated a Professional **Demeanor.** |  |  |
| 1. Appeared **Professional** |  |  |
| 1. **Attentive Listening** | **Yes** | **No** |
| 1. **Non-verbal** expression of interest/concern: body language, nodding, eye contact |  |  |
| 1. **Summarized** information and/or repeated information back to the patient |  |  |
| 1. The examinee **did not** unnecessarily **interrupt** the patient. |  |  |
| 1. **Information Gathering** | **Yes** | **No** |
| 1. Information was collected in an **organized** fashion |  |  |
| 1. Asked a combination of **open-ended questions** and **closed-ended questions** |  |  |
| 1. Asked questions in a **clear, articulate**, **straightforward** manner. |  |  |
| 1. **Provided Information** | **Yes** | **No** |
| 1. Informed the patient of a differential diagnosis (DDx) in an understandable way. |  |  |
| 1. Spoke to the patient using clear statements and language |  |  |
| 1. Explained the next steps and the reasons behind them |  |  |
| 1. **Assisted Patient in Decision Making** | **Yes** | **No** |
| 1. Assessed the patient’s willingness and ability to carry out the next steps |  |  |
| 1. Encouraged questions by asking patient if they had any questions |  |  |
| 1. Understands, determines, or asks if patient has concerns |  |  |
| 1. **Supported Emotions** | **Yes** | **No** |
| 1. Demonstrated genuineness, caring, concern, and respect. |  |  |
| 1. Used statements of empathy, understanding, support or validation of patient’s feelings/perspective. |  |  |
| 1. **Return for Future Care** | **Yes** | **No** |
| 1. The patient would return to the examinee for future care. |  |  |
| 1. **Had a Good Presence During the Telemedicine Visit. (Only used during Telemedicine Virtual OSCEs)** | **Yes** | **No** |
| 1. The examinee positioned themselves adequately on screen |  |  |
| 1. The examinee made an effort to look at the camera to maintain eye contact. |  |  |
| 1. The examinee was aware of the potential for slight delays in videoconferencing and appropriately waited to continue speaking |  |  |
| 1. Spoke naturally as if you were in the exam room. |  |  |
| 1. The examinee ensured a professional environment |  |  |
| **Comments:** | | |

**SP Guide to the Checklist (Case: James/Jaime Spiegel)**

1. **I use marijuana and a little cocaine.**

“Do you use any recreational drugs?”

“Do you use any illicit drugs?”

“Do you use any illegal drugs?”

1. **Sometimes I use some of my fiancé’s pain medication if I am having a bad day.**

“Do you use any drugs not prescribed to you?”

“Do you ever use any other prescribed or prescription medications for your pain?”

3. **Yes.**

“Have you ever needed an early refill before?”

“Has anything ever happened like this before where you needed an early refill?”

“Do you find that you often run out of your medication early?”

1. **I don’t know, maybe 10 times over the past 5 years.**

“How many times have you needed an early refill?”

1. **Yes, when I need to.**

“Have you ever taken your prescription pain medicine in a way other than prescribed?”

“Have you ever taken any extra doses of prescribed pain medicine?”

“Are you taking more than 1 tablet every 12 hours?”

1. **It’s been at least 5 years.**

“How long has it been since someone has performed testing to re-evaluate your back pain?”

“When was the last time that you had any diagnostic tests (x-ray, MRI, etc.) to evaluate your back pain?”

“When was the last time that someone did a “work-up” for your back pain?”

1. **My father was an alcoholic.**

“Does anyone in your family have a history of substance abuse?”

“Has anyone in your family ever had a problem with alcohol or drugs?”

1. **The student instructed me that prescription medication should never be in checked luggage.**

The student should indicate to you that prescription medication should never be placed in checked luggage.

1. **The student reinforced that this medication should always be taken as directed.**

The student discussed that any prescribed mediation should only be taken as directed/prescribed.

1. **The student informed me that taking a controlled medication prescribed for someone else is against the law.**

The student should inform you that taking a controlled medication such as this which is prescribed for someone else is illegal.

**SP Guide to the Checklist (Case: Darryl Whitcomb)**

1. **No. I like my beer and cigarettes but don’t touch anything else.**

“Do you use any recreational drugs?”

“Do you use any illicit drugs?”

“Do you use any illegal drugs?”

1. **Nothing other than some Advil or Tylenol.**

“Do you use any drugs not prescribed to you?”

“Do you ever use any other medications for your pain?”

3. **Yes.**

“Have you ever needed an early refill before?”

“Has anything ever happened like this before where you needed an early refill?”

“Do you find that you ever run out of your medication early?”

1. **I don’t know, maybe 2 times over the past 7 years.**

“How many times have you needed an early refill?”

1. **Nope.**

“Have you ever taken your prescription pain medicine in a way other than prescribed?”

“Have you ever taken any extra doses of prescribed pain medicine?”

“Are you taking more than 1 tablet every 12 hours?”

1. **It’s been at least 5 years.**

“How long has it been since someone has performed testing to re-evaluate your neck/back pain?”

“When was the last time that you had any diagnostic tests (x-ray, MRI, etc.) to evaluate your neck/back pain?”

“When was the last time that someone did a “work-up” for your neck/back pain?”

1. **My father was an alcoholic.**

“Does anyone in your family have a history of substance abuse?”

“Has anyone in your family ever had a problem with alcohol or drugs?”

1. **The student instructed me that prescription medication should always be stored in a safe and secure location.**

The student instructed me that prescription medication should always be stored in a safe and secure location.

1. **The student reinforced that this medication should always be taken as directed.**

The student discussed that any prescribed mediation should only be taken as directed/prescribed.

1. **The student informed me that taking a controlled medication involves taking responsibility for safe storage practices.**

The student should inform you that being prescribed a controlled substance like an opioid medication requires responsible behavior, always requiring safe and secure storage.

**SP Guide to the Checklist (Case: Helen Morgan)**

1. **Oh my, no. I have a rare drink on special occasions.**

“Do you use any recreational drugs?”

“Do you use any illicit drugs?”

“Do you use any illegal drugs?”

1. **No Doctor, I do not think that would be safe, especially since I’m taking so many different medicines.**

“Do you use any drugs not prescribed to you?”

“Do you ever use any other medications for your pain?”

3. **Yes.**

“Have you ever needed an early refill before?”

“Has anything ever happened like this before where you needed an early refill?”

“Do you find that you often run out of your medication early?”

1. **Every so often, when the pills get “lost” maybe once a year.**

“How many times have you needed an early refill?”

1. **Oh no, never.**

“Have you ever taken more prescription pain medicine other than prescribed?”

“Have you ever taken any extra doses of prescribed pain medicine?”

“Are you taking more than 1 tablet every 12 hours?”

1. **It’s been at least 10 years.**

“How long has it been since someone has performed testing to re-evaluate your back pain?”

“When was the last time that you had any diagnostic tests (x-ray, MRI, etc.) to evaluate your back pain?”

“When was the last time that someone did a **“work-up”** for your back pain?”

1. **My father did like his scotch and sodas.**

“Does anyone in your family have a history of substance abuse?”

“Has anyone in your family ever had a problem with alcohol or drugs?”

1. **The student instructed me that prescription medication should always be stored in a safe and secure location.**

The student instructed me that prescription medication should always be stored in a safe and secure location.

1. **The student reinforced that this medication should always be taken as directed.**

The student discussed that any prescribed mediation should only be taken as directed/prescribed.

1. **The student informed me that taking a controlled medication involves taking responsibility for safe storage practices.**

The student should inform you that being prescribed a controlled substance like an opioid medication requires responsible behavior, always requiring safe and secure storage.

Use this guide to assist in your checklist assessment of **Patient-Centered Communication Skills**.

The examples listed are only a reference and not the exact language the learner must use. Each bulleted example is NOT required for credit. There can be additional behaviors/language used to receive credit.

1. **Appropriate Greeting**
2. **Introduced themselves and showed ID badge.**

- Examinee can use their first or last name or both to introduce themselves.
- Examinee showed their ID Badge to the camera. **Note:** If the examinee forgets to show their ID you will still give credit but make a reference in the comment box and you will ask: **“I am so sorry, I have never met you, could you show me your ID so I can confirm it’s you?”**

1. **Explained their role/title.**

- Indicates their role/title, (ex. medical/nursing student, first year, student doctor, Dr. Smith). They do not need to state their tasks, (ex. “I am here to take a history/physical exam.”).

1. **Addressed the patient by name.**

- Must verify the patient’s name using their first or last name or both.
- May ask the patient to state their name and date of birth. (DOB is not required).

1. **Referred to patient using first name, last name or both.**

- Choose “first name” if the student address you only using your first name.
- Choose “last name” if the student address you only using “Mr.”, “Mrs.” Or “Ms.” and your last name.
- Choose “asked preference” if the student asks you what name you prefer that they use
- Choose “N/A” if the student never addresses you by name to does not do one of the above choices and then please list a comment. ***Modified**

1. **Building the Relationship**
2. **Showed interest in the patient, as a person, not just their condition.**

- By asking about personal life, home situation, support system, or concerns.
- Examinee could express concern over unhealthy habits, ex. smoking, alcohol/drug use or ask if you were injured due to a fall.
- Determined how the health issue is affecting the patient’s life. May directly ask “How has this affected or impacted your life?” or “Did you miss work because of this?”, “Has this caused any changes in your normal routine?”, “Can you drive/climb stairs?”, “Do you have help at home?”

1. **Demonstrated a Professional Demeanor.**

- The examinee did not talk down to the patient. This is subjective/related to tone.
- Friendly, personable, appropriate pace (not too fast or too slow), word choice.
- Listened with full attention. Did not appear distracted, (ex. consistently looking at their watch/clock/timer).

1. **Appeared Professional.**

- Professional dress (clean, neat, business casual attire), clean white lab coat.

1. **Attentive Listening**
2. **Non-verbal expression of interest/concern: body language, nodding, eye contact.**

- Any body language that indicates or expresses an encouragement to speak. As compared to a closed body language like crossed arms, standing very far away or avoiding eye contact.
- Note-taking was balanced with conversation and eye contact. (Do not penalize the examinee if they do or do not take notes).

1. **Summarized information and/or repeated information back to the patient.**
   - **Repeating one thing** the patient said back to the patient will suffice. This can occur at any time during the encounter. **Note:** If the examinee repeats incorrect information you should correct them. Multiple corrections should not occur. (3 or more)
2. **The examinee did not unnecessarily interrupt the patient.**

- Allowed patient to fully answer questions asked.
- They may interrupt appropriately to clarify information, however, continuous unnecessary interruptions should not occur.

1. **Information Gathering**
2. **Information was collected in an organized fashion.**

- There was a flow to the questions. Did not jump abruptly from subject to subject.

**Note:** The examinee can go back to an earlier topic if they verbalize there is additional information they need or should have asked.

1. **Asked a combination of open-ended questions and closed-ended questions.**

- Allows the patient to explain their needs in their own words, ex. “What brings you in?”, “Tell me more.” “Can you expand on that?” Followed by closed-ended more direct questioning, ex. yes/no or direct response type questions.

1. **Asked questions in a clear, articulate, straightforward manner.**

- Did not ask multiple leading questions or multiple stacked questions. (3 or more)
- Avoided repeating questions multiple times. (3 or more)

1. **Provided Information**
2. **Informed the patient of a differential diagnosis (DDx) in an understandable way.**

- Must tell/discuss more than one possible diagnosis. This does not always happen at the end of the encounter, it may happen earlier during the history taking. **Important**: The DDX does not have to be correct.

**Note:** Examinee may rule out a differential diagnosis by stating, “I don’t believe this is cancer.” and this would also be acceptable.

1. **Spoke to the patient using clear statements and language.**

- Avoided medical jargon OR explained any technical terms in plain language. Medical terms are allowed if followed by a clear explanation OR the learner confirms the patient’s understanding**.**
- If *you* the SP (not the person you are playing) truly does not know how to accurately answer a question because of jargon you will say: “I don’t know what you mean.” If you know the medical word, you will answer the question, but will still give a “no” for this item if medical jargon was not clarified.

1. **Explained the next steps and the reasons behind them.**

- Clearly explains the next steps, reason for them and assesses the patients understanding of the next steps.
- Simply saying “I will speak to my preceptor”, or “I am going to run some “TESTS.” is NOT adequate to receive credit. Do NOT ask “What tests?” - It is their job to explain what tests without your asking.

1. **Assisted Patient in Decision Making**
2. **Assessed the patient’s willingness and ability to carry out the next steps.**

- “How do you feel about that?” They should check to see if you “buy in” to the plan.
- They may simply say “Okay?” after they have discussed the plan. As long as you realize they are asking if the plan sounds “Okay” credit is given.
- Be aware of your own behavior and avoid consistent head nodding throughout the encounter. This can be perceived as you “agreeing” with everything being said and the student may never inquire about your “buy in”.

1. **Encouraged questions by asking the patient if they had any questions.**

- Asked if you had any questions. (This could occur at any point during the encounter.)
- Asked if you understood or had any questions regarding the DDX and/or the plan.

1. **Understands, determines or asks if patient has concerns.**

- Develops an understanding of the patient’s expectations and priorities of the visit**.**
- Addresses/asks patient’s concerns pertaining to their reason for the visit.
- May directly ask: “Do you have any concerns?”, “I can see you are concerned about…”

**Note Re #27 & 28:** If the examinee stacks the question “Any questions or concerns?” answer the last thing correctly and make a note in the comment box if they do not go back and clarify the other question. Be aware they may ask if you have “any questions/concerns” at any time during the interview.

1. **Supported Emotions**
2. **Demonstrated genuineness, caring, concern and respect.**

- Respectful and non-judgmental of patient’s feelings.
- Sensitive to discussing difficult subjects such as, questions regarding sexual preferences or practices, questions about drinking/drug use. ex. “I ask this of all my patients…”

1. **Used statements of empathy, understanding, support or validation of patient’s feelings/ perspective.**

- Correctly interprets how the patient is feeling. (ex. nervous, scared, sad, frustrated, annoyed).
- Acknowledgement of concern/pain or attempts/offers to give relief.
- Made supportive or reflective statements: ex. “This must be very upsetting or scary for you.”, “You came to the right place.”, “We will get you the help you need.”, “I can understand how you are feeling.”, “You seem so sad.”, “That sounds difficult or painful.”, “I can see you are in pain.” “I’m sorry to hear this.”, “You look uncomfortable.”, “I’m concerned about you.”, “I’m so sorry to hear of your loss.” **Note**: “I’m sorry” someone passed is not the only empathic statement you should be looking for. However, it may be relevant based on the specific case details.

1. **Return for Future Care**
2. **The patient would return to the examinee for future care.**

- If a **“No”** is given for this item a comment is required. If you are **undecided or “on the fence”** as to whether or not you would return you should give the examinee a **“No”** and explain. A “no” for this question is **rare** and must be supported with comments regarding the examinee’s performance.
- Personal biases and preferences should not sway your decision. It should be based solely on the student’s communication skills during the encounter.

1. **Had a Good Presence During the Telemedicine Visit. [ONLY USED DURING VIRTUAL OSCEs]**
2. **The examinee** **positioned themselves adequately on screen with minimal distractions.**

- Positioned and centered in front of their electronic device.
- The background was relatively neutral with minimal distractions.

1. **The examinee made an effort to look at the camera to maintain eye contact.**

- There was a balance between looking at the camera and looking at the patient on the screen, when speaking and listening to the patient.

1. **The examinee was aware of the potential for slight delays in videoconferencing and appropriately waited to continue speaking.**

- The examinee was patient when lag times interfered between questions and responses.

1. **Spoke naturally as if you were in the exam room.**

- The examinee spoke in a natural tone of voice as they would in a face-to-face visit.

1. **The examinee ensured a professional environment.**

- The examinee’s background was appropriate – ex. neutral or with minimal distractions.
- The examinee was in a private location without distractions or others present (ex. No pets, family members, etc...
- The examinee spoke in a natural tone of voice as they would in a face-to-face visit.
