# Supplementary material for: Early Refill of an Opioid Medication: Recognizing Personal Biases Through Clinical Vignettes and OSCEs
Source: MedEdPORTAL. 2022 Apr 7;18:11234. doi: 10.15766/mep_2374-8265.11234 (PMC8986891; doi:10.15766/mep_2374-8265.11234)
Supplement: Supplementary file 1 — MS 1 Clinical Vignettes & Follow-Up.pptxMS 1 Debrief.pptxSP James Spiegel - Case 1.docxSP Darryl Whitcomb - Case 2.docxSP Helen Morgan - Case 3.docxDoor Notes.docxLogistical Flow.docxFaculty Post-OSCE Debrief Discussion Guide.docxSP Encounter Checklist.docxSP Responses for Checklist Items.docxMS 3 Post-OSCE Survey.docx [file mep_2374-8265.11234-s001.zip › K. MS 3 Post-OSCE Survey.docx]

| 1. Which patient case did you just participate in? | |
| --- | --- |
| ⃝ | James Spiegel |
| ⃝ | Darryl Whitcomb |
| ⃝ | Helen Morgan |
| 1. Which of the following would best describe your **level of comfort** with respect to this patient's request for an early refill of an opioid medication? | |
| ⃝ | Uncomfortable due to the current climate regarding the opioid epidemic. |
| ⃝ | Somewhat uncomfortable based on this patient's long-term opioid history and the reason for the early refill. |
| ⃝ | Comfortable based on this patient's documented pathology and medical history regarding his/her pain and function. |
| ⃝ | Comfortable based my interaction with the patient. |
| 1. Which of the following best describes your **course of action** regarding providing an early refill of opioid medication to this patient? | |
| ⃝ | Comfortable and would likely provide the prescription. |
| ⃝ | Uncomfortable and would provide the prescription with a warning about not providing future early refill requests. |
| ⃝ | More information would be needed before a refill could be provided. |
| ⃝ | Under no circumstances should an early refill for the opioid medication be provided. |
| ⃝ | The patient is likely drug-seeking and should be discharged from practice. |
| 1. Additional thoughts and comments? | |
|  | |
