# Supplementary material for: Early Refill of an Opioid Medication: Recognizing Personal Biases Through Clinical Vignettes and OSCEs
Source: MedEdPORTAL. 2022 Apr 7;18:11234. doi: 10.15766/mep_2374-8265.11234 (PMC8986891; doi:10.15766/mep_2374-8265.11234)
Supplement: Supplementary file 1 — MS 1 Clinical Vignettes & Follow-Up.pptxMS 1 Debrief.pptxSP James Spiegel - Case 1.docxSP Darryl Whitcomb - Case 2.docxSP Helen Morgan - Case 3.docxDoor Notes.docxLogistical Flow.docxFaculty Post-OSCE Debrief Discussion Guide.docxSP Encounter Checklist.docxSP Responses for Checklist Items.docxMS 3 Post-OSCE Survey.docx [file mep_2374-8265.11234-s001.zip › Educational_Summary_Report_11234.pdf]

MedEdPORTAL® The AAMC Journal of Teaching and Learning Resources

Original Publication

Open Access

Early Refill of an Opioid Medication: Recognizing Personal Biases Through Clinical Vignettes and OSCEs

Wei-Hsin Lu, PhD*, Perrilynn Baldelli, DNP, RN, Phyllis Migdal, MD, MA, Richard Iuli, PhD, Lisa Strano-Paul, MD, Kevin L. Zacharoff, MD

*Corresponding author: wei-hsin.lu@stonybrook.edu

Abstract

Introduction: Efforts to improve pain education and knowledge about prescription opioid misuse and opioid/substance use disorder in undergraduate medical education continue to be inadequate. To advance educational practices and address training needs to counter the opioid epidemic, we created a longitudinal pain and addiction curriculum that includes three patient vignettes in which the patient requests an early refill of opioid medication. The goal was to introduce students to the potential impact of personal biases on health care delivery and medical decision-making with patients who have pain and/or substance use disorders. **Methods:** Three clinical vignettes were presented to early matriculating medical students (MS 1s) using a progressive case disclosure approach in the format of a PowerPoint presentation with embedded audio interactions and follow-up audience response system questions. The same vignettes were converted into OSCEs for early clinical clerkship students (MS 3s). **Results:** A total of 180 MS 1s participated in the case presentations, and 124 MS 3s participated in the OSCE session. There was a significant difference between students' level of comfort and individual patient requests for early prescription refills in both student cohorts. MS 1s were significantly more likely to provide the early refill to the elderly female patient compared to the two middle-age male patients, whereas a majority of MS 3s wanted more information. **Discussion:** This module can be presented to medical students who have little clinical exposure and to health care trainees at other levels of clinical exposure.

Keywords
Substance Use Disorder, Bias, Clinical Skills Assessment/OSCEs, Standardized Patient, Substance Abuse/Addiction, Opioids, Addiction, Pain

Educational Objectives

By the end of this activity, learners will be able to:

1. Perform a (virtual) patient history to make/confirm a diagnosis.
2. Demonstrate knowledge and competency for identifying substance use disorder (SUD).
3. Appropriately assess a request for early refill of a prescription opioid analgesic.
4. Recognize the potential impact of biases on clinical decision-making and the assessment and treatment of pain in the potential context of aberrant drug-related behaviors (e.g., misuse, abuse, diversion) and/or SUDs.

Citation:
Lu WH, Baldelli P, Migdal P, Iuli R, Strano-Paul L, Zacharoff KL. Early refill of an opioid medication: recognizing personal biases through clinical vignettes and OSCEs. *MedEdPORTAL.* 2022;18:11234.
https://doi.org/10.15766/mep_2374-8265.11234

Introduction

Health care providers, regulatory agencies, medical educators, patients, and other key stakeholders in the US face a number of significant challenges today. Among the most pressing is the tide of opioid-related deaths, which are approaching, on average, 130 lives lost every day.[1] Intricately connected to opioid use is the management of patients who suffer from chronic pain. Safe, effective pain management by health care providers requires substantive knowledge of the principles of opioids as therapeutic agents as well as effective assessment of the risks for unhealthy use, addiction, and diversion associated with opioid use both individually and as a medication class.[2] It is clear that no single strategy or target for managing the opioid use crisis is likely to succeed on its own. Rather, what is needed is an integrated, multilevel, multiprong approach involving medical education and training, therapeutic alliances, and evidence-based interventions that enlist all involved medical specialties and health care providers to achieve the desired outcomes—treating patients in need while mitigating risks and saving lives.[3,4]

Copyright © 2022 Lu et al. This is an open-access publication distributed under the terms of the Creative Commons Attribution license.

Additionally, biases and stigmatized ideas held by health care providers, patients, patients' family members, and the general population are common challenges encountered in managing patients with chronic pain and/or substance use disorders (SUDs). Implicit bias, also known as implicit social cognition, refers to the attitudes or stereotypes that affect an individual's knowledge, perceptions, actions, and decisions in an unconscious manner.[5] The literature points out that health care professionals admit to negative attitudes towards patients with SUDs, and correlating evidence indicates that these biases likely influence diagnoses, treatment decisions, and levels of care.[6,7] Unfortunately, these biases are difficult to explicitly identify. Even so, there is evidence suggesting that addiction specialists who undergo enhanced training and education in SUDs are able to lessen their biases towards patients with SUDs.[8] Although there are curricula published in *MedEdPORTAL* related to either prescribing opioid medication[9] or the impact of implicit biases on patient care,[10] there is a lack of published curricula at the intersection of both topics in the context of early refill requests in which the target learners are early-stage medical students.

The Renaissance School of Medicine at Stony Brook University designed and developed the Integrated Pain and Addiction Curriculum (iPAC) focusing on opioid-related education and training. The iPAC program is integrated longitudinally across our existing LEARN (Learner-focused, Experiential, Adaptive, Rigorous, and Novel) curriculum, which first establishes baseline knowledge and skills and then gradually builds on this foundation through application and practice. Through iPAC, our students also learn to recognize the impact that physician biases have on patient care and medical decision-making, especially in the assessment and care of patients with pain and/or SUDs for whom opioid analgesics may be determined to be an appropriate component of therapy. Students have opportunities to become aware of personal biases, which are naturally inherent in a physician's perspective, and to utilize self-reflection as a method for understanding and mitigating their biases. Students are also encouraged to express their thoughts and emotions on issues and/or patient care experiences related to pain management, addiction, and opioid overdose.

This project describes three patient vignettes we developed as part of iPAC. The goal of these vignettes was to introduce medical students to the potential impact of personal biases on health care delivery and medical decision-making with patients who have pain and/or SUDs within the context of patient early refill requests. This module can be presented to early-stage medical students who have very little clinical exposure as well as to health care trainees with varying levels of clinical exposure.

Methods

The target learners for the patient vignettes included preclerkship and clerkship students. Our LEARN curriculum comprised three distinct phases: Phase I (18-month foundational phase), Phase II (12-month primary clinical phase), and Phase III (16-month advanced clinical phase). Three required transition courses were offered at key junctures of the medical training continuum: Transition to Medical and Dental School (TMDS), a 2-week course for matriculating medical students at the beginning of Phase I; Transition to Clinical Care (TCC), a 2-week course for mid-level medical students preparing to begin Phase II clinical clerkships; and Transition to Residency, a 2-week course for Phase III medical students 3-4 months before graduation. We presented three patient vignettes to first-year medical students (MS 1s) in the TMDS course and then again to third-year medical students (MS 3s) in the TCC course.

Preclinical Implementation

During a 2-hour session in the TMDS course, we started by presenting the three clinical vignettes using a progressive case disclosure approach. We used a PowerPoint presentation with embedded audio recordings for the three-part interactions between a physician and three separate patients, James Spiegel, Darryl Whitcomb, and Helen Morgan (Appendix A). Each vignette took approximately 15-20 minutes to present and discuss the audience response system questions used to poll students on their thoughts as the case unfolded.

We began by introducing learners to the physician, Dr. Melvin Cullen, who was portrayed as a distinguished White/possibly multiracial male. This was intentional to maintain the focus of MS 1 learners (who had little to no clinical exposure) during the exercise on the aspects of bias, judgment, and precognitive thinking in considering requests for an early opioid refill.

All three patients were being treated with chronic opioid therapy and had requested an early prescription refill from Dr. Cullen. Each vignette started with a door note, which included the context of the patient's visit; then, the visit was presented in three parts, each including a physician-patient interaction recording, learner poll question, and discussion. We designed all questions to prompt students to think about patient care and medical decision-making, especially with regard to patients who were dealing with chronic pain and who might be at risk for substance abuse. In terms of outcome measures related to bias, it was not

Copyright © 2022 Lu et al. This is an open-access publication distributed under the terms of the Creative Commons Attribution license.

the objective of these exercises to change our students' biases but rather to have them recognize that implicit biases do exist and that these biases could affect clinical decision-making.

Visit part 1: We began with a 1- to 1.5-minute physician-patient interaction at the start of the patient's visit. This recording included the request from the patient for an early refill of an opioid medication.

We then paused the physician-patient interaction and presented the patient's medical history and physical examination results. We posed a question to the students regarding their level of comfort with respect to this patient's request for an early refill of an opioid medication and asked them to respond via the audience response system (Question 1). We did not define the meaning of the term *comfort* or expect it to be the same for all of our learners, as we understood that the meaning would vary based on each individual's prior experiences, attitudes, and beliefs. In fact, this was the very reason why we created such an exercise—we wanted our students to realize that an individual's preferences and experiences influence their thoughts (consciously or unconsciously) and feelings (e.g., comfort level). Hence, as a first step, it was important to be able to recognize and understand that one's own biases, particularly unconscious biases, could affect one's decisions.

Visit part 2: We resumed the physician-patient interaction, and the vignette unfolded to reveal critical information to stimulate students to think about personal and professional biases and possible rationales for these biases. The second audience response question asked learners about their intended course of action (Question 2). As noted earlier, our medical students in the MS 1 TMDS course were in the early stages of their training, with little to no clinical exposure. Rather than asking them to indicate whether or not they would agree to the patients' requests for an early refill of an opioid medication or identify the risks/concerns of making such decisions, we wanted to focus more on how they felt about these situations based on the different patient vignettes and their perceptions of the physician-patient interactions. We then provided students with a summary of the physician's assessment and management plan.

Visit part 3: After concluding the physician-patient interaction, we asked students whether they thought the physician in the clinical vignettes made an objective medical assessment and management plan for each of the three patients (Question 3). After presenting all three vignettes, we asked whether the students thought any, all, or none of the three patients presented were drug-seeking (Question 4).

Debrief: After reviewing the three vignettes, we conducted an hour-long follow-up debrief and didactic presentation (Appendices A and B). The purpose of the follow-up debrief was to address Educational Objective 4 and provide takeaway points.

Clinical Implementation

In the TCC course, we converted the same three clinical vignettes presented during TMDS into standardized patient (SP) OSCE cases (Appendices C-E; door notes in Appendix F). This was a full-day event, with groups of MS 3 students rotating through our Clinical Skills Center every 30 minutes until all students had completed one of the three OSCE cases, as detailed in Appendix G. We followed each group OSCE session with a 30-minute, faculty-facilitated debrief session that provided students with another opportunity to address Educational Objective 4. The faculty debriefers were trained during a live 1-hour meeting with the subject matter expert using a developed debrief discussion guide (Appendix H). Due to the COVID-19 pandemic, we delivered the OSCE encounters remotely through Zoom, but the OSCE cases and the logistical flow were the same regardless of the format.

SP training: The development of the OSCE required the standardization and training of the SPs portraying each case. Training sessions were conducted by an experienced SP educator with consultation of a subject matter expert. Each training was a 2-hour session that consisted of a review of the SP case materials (Appendices C-E) and the SP encounter checklist and responses (Appendices I and J). After the review of the case materials, we gave clarifications for any outstanding questions regarding them. A role-play exercise was also completed in order to ensure standardization of patient portrayal, completion of the SP encounter checklist, and consistency in SP responses to the checklist items.

Assessment: We assessed the MS 3 students using a 37-item SP encounter checklist (Appendix I). The checklist was developed by the subject matter expert and an experienced SP case developer by determining the critical action items that needed to be completed by a health care provider in normal clinical practice with the patient cases (standard practice for learner evaluation in our Clinical Simulation Center). The checklist included observable action items/behaviors in three categories: history taking (seven items), information sharing/patient education (three items), and patient-centered communication skills (27 items). The patient-centered communication skills category was further broken down into nine subcategories: appropriate greeting, building the relationship, attentive listening, information gathering, provided

Copyright © 2022 Lu et al. This is an open-access publication distributed under the terms of the Creative Commons Attribution license.

information, assisted patient in decision-making, supported emotions, return for future care, and had a good presence during the telemedicine visit (only used during virtual teleOSCEs). For each item, the SPs checked off whether the student had performed/demonstrated each action/behavior (yes/no). One point was given for each item checked yes, resulting in a total of up to 37 points (Appendix J). The SPs completed the checklist evaluations electronically using our simulation software system.

We also asked the MS 3 students to complete a brief post-OSCE survey (Appendix K) at the beginning of the debrief session before the facilitators started the discussion. The post-OSCE survey consisted of the same two questions asked of MS 1 students regarding the student's level of comfort with respect to the patient's request for an early refill of an opioid medication (Question 1) and the student's intended course of action regarding providing an early refill of opioid medication to this patient (Question 2).

Evaluation

We compared MS 1 and MS 3 students' responses to Questions 1 and 2 to determine if there was a difference in level of comfort and intended course of action at different levels of training. A chi-square test was performed to compare both MS 1 and MS 3 students' comfort level with the requests for an early refill of an opioid medication among the three patient cases and their intended course of action. We reviewed completed SP checklists to evaluate MS 3 students' OSCE performance.

Results

A total of 180 students (136 medical, 44 dental) participated in the TMDS course, and all MS 1 students responded to the audience response questions for each of the three patient vignettes. There was a significant difference between level of comfort and individual patient request ($\chi^2 = 180.7$, $p < .001$; Table 1). MS 1 TMDS students were more comfortable with Helen Morgan's early refill request (Case 3) compared to the requests from James Spiegel (Case 1) and Darryl Whitcomb (Case 2).

Regarding a student's intended course of action, MS 1 students were more likely to indicate they needed additional information before providing the prescription for James Spiegel (Case 1) and Darryl Whitcomb (Case 2) than for Helen Morgan (Case 3; $\chi^2 = 110.5$, $p < .001$; Table 2).

When MS 1 students were asked if they thought any of the three patients presenting were drug-seeking, 34% ($n = 59$) indicated they did not think any of the patients were drug-seeking, whereas 16% ($n = 28$) indicated they thought all of the patients were drug-seeking. Of MS 1 students, 30% ($n = 53$) indicated that they thought James Spiegel (Case 1) was drug-seeking, 14% ($n = 25$) thought that Darryl Whitcomb (Case 2) was drug-seeking, and 5% ($n = 9$) believed that Helen Morgan (Case 3) was drug-seeking.

A total of 124 MS 3 students participated in the TCC course. MS 3 students were randomly assigned to complete one of the three OSCEs: James Spiegel (Case 1; $n = 41$), Darryl Whitcomb (Case 2; $n = 42$), and Helen Morgan (Case 3; $n = 41$). When asked about their level of comfort following the OSCE encounter, MS 3 students were significantly more likely to be either uncomfortable or somewhat uncomfortable (Table 1) with James Spiegel's request for an early refill (Case 1; $\chi^2 = 8.0$, $p = .02$; Table 1), whereas there was no significant difference with the students' intended courses of action for the three individual patients ($\chi^2 = 4.1$, $p = .66$; Table 2). A majority of the MS 3 students wanted more information before providing an early refill to any of the three patients: James Spiegel (Case 1), 57%; Darryl Whitcomb (Case 2), 68%; and Helen Morgan (Case 3), 61%.

SP Checklist

For the history-taking checklist items, most MS 3 students ($n = 88$, 74%) asked, "Do you use any recreational/illicit/illegal drugs?" (Table 3). MS 3 students on average completed three of the seven items, with two students not completing any of the checklist items and seven students completing six of the seven items. No student performed all seven checklist items. Regarding MS3 students' information sharing/patient education, 82%

Table 1. Comparison of MS 1 TMDS and MS 3 TCC Students' Responses to Question 1 for the Three Patient Cases

Class	Level of Comfort	No. (%)			χ^2 (p)
		James Spiegel (Case 1)[a]	Darryl Whitcomb (Case 2)[b]	Helen Morgan (Case 3)[c]	
MS 1 TMDS	Uncomfortable/somewhat uncomfortable	146 (83)	174 (96)	64 (36)	180.7 (<.001)
	Comfortable	29 (17)	7 (4)	116 (64)	
MS 3 TCC	Uncomfortable/somewhat uncomfortable	29 (83)	22 (58)	19 (47)	8.0 (.02)
	Comfortable	6 (17)	16 (42)	17 (53)	

Abbreviations: MS 1, first-year medical student; MS 3, third-year medical student; TCC, Transition to Clinical Care course; TMDS, Transition to Medical and Dental School course.
[a]MS 1 TMDS, $N = 175$; MS 3 TCC, $N = 35$.
[b]MS 1 TMDS, $N = 181$; MS 3 TCC, $N = 38$.
[c]MS 1 TMDS, $N = 180$; MS 3 TCC, $N = 36$.

Copyright © 2022 Lu et al. This is an open-access publication distributed under the terms of the Creative Commons Attribution license.

Table 2. Comparison of MS 1 TMDS and MS 3 TCC Students' Responses to Question 2 for the Three Patient Cases

Class	Intended Course of Action	No. (%) James Spiegel (Case 1)[a]	Darryl Whitcomb (Case 2)[b]	Helen Morgan (Case 3)[c]	χ^2 (p)
MS 1 TMDS	Comfortable providing the prescription	3 (2)	2 (1)	42 (24)	110.5 (<.001)
	Uncomfortable but would provide the prescription with a warning about not providing future early refill requests	65 (37)	45 (25)	81 (45)	
	More information needed before a refill could be provided	103 (59)	121 (67)	54 (30)	
	Should not provide/drug-seeking	5 (3)	12 (7)	2 (1)	
MS 3 TCC	Comfortable providing the prescription	6 (17)	8 (21)	9 (25)	4.1 (.66)
	Uncomfortable but would provide the prescription with a warning about not providing future early refill requests	8 (23)	3 (8)	4 (11)	
	More information needed before a refill could be provided	20 (57)	26 (68)	22 (61)	
	Should not provide/drug-seeking	1 (3)	1 (3)	1 (3)	

Abbreviations: MS 1, first-year medical student; MS 3, third-year medical student; TCC, Transition to Clinical Care course; TMDS, Transition to Medical and Dental School course.
[a]MS 1 TMDS, $N = 176$; MS 3 TCC, $N = 35$.
[b]MS 1 TMDS, $N = 180$; MS 3 TCC, $N = 38$.
[c]MS 1 TMDS, $N = 179$; MS 3 TCC, $N = 36$.

($n = 98$) did not perform any of the three checklist items. Eleven students (9%) performed one checklist item, eight students (7%) performed two items, and three students (3%) performed all three items. The item completed by the most students ($n = 16$, 13%; Table 3) was "the student reinforced that this medication should always be taken as directed."

For the 27 checklist items targeting patient-centered communication skills, MS 3 students on average completed 25 items. The three checklist items with the lowest percentages of student completions included (1) asked if the patient has concerns ($n = 81$, 68%), (2) encouraged patient to ask questions ($n = 86$, 72%), and (3) referred to patient by asking preference (e.g., "How would you like to be called?"; $n = 86$, 72%).

Discussion

We developed three patient vignettes regarding an early refill request of an opioid medication. The patient vignettes were first introduced to MS 1s during their TMDS course using a progressive case disclosure approach in the format of a PowerPoint presentation with embedded audio recording interactions between a physician and each of the three patients. The same three vignettes were presented to MS 3s during the TCC course through SP OSCE encounters. Due to COVID-19 restrictions, both sessions were offered virtually via Zoom; they were originally designed to be implemented in person.

MS 1s in TMDS were significantly more comfortable with the request for an early refill by Helen Morgan (Case 3), a 77-year-old White female patient with chronic pain related to advanced diffuse degenerative joint disease, compared to the requests for an early refill by the other two patients (Case 1: James Spiegel—a 39-year-old White male with chronic back pain, and Case 2: Darryl Whitcomb—a 56-year-old African American male with chronic neck and back pain). In a follow-up question regarding the MS 1 students' intended course of action, over two-thirds (69%) indicated they would have provided the prescription to Helen Morgan (Case 3), although nearly half of them (45%) indicated they would be uncomfortable doing so and would provide the prescription with a warning about future early refill

Table 3. Third-Year Medical Students' Standardized Patient Checklist Performance ($N = 124$)

Checklist Items	No. (%)
History taking—the student asked:	
Do you use any recreational/illicit/illegal drugs?	88 (74)
Do you use any drugs not prescribed to you?	63 (53)
Have you ever needed an early refill before? Or: Do you find you often run out of your medication early?	32 (26)
How many times have you needed an early refill?	18 (15)
Have you ever taken your prescription pain medication in a way other than prescribed or taken extra doses?	41 (34)
How long has it been since someone has performed testing to reevaluate your back pain? Or: When was the last time you had a diagnostic test (X-ray, MRI, etc.) to evaluate your back pain?	41 (34)
Does anyone in your family have a history of substance abuse (problem with alcohol or drugs)?	66 (55)
Information sharing/patient education	
The student instructed me that prescription medication should always be stored in a safe and secure location.	10 (8)
The student reinforced that this medication should always be taken as directed.	16 (13)
The student informed me that taking a controlled medication involves taking responsibility for safe storage practices.	10 (8)

Copyright © 2022 Lu et al. This is an open-access publication distributed under the terms of the Creative Commons Attribution license.

requests. Nevertheless, more MS 1 students were willing to provide Helen Morgan (Case 3) with the prescription relative to the other two patients.

Although MS 3s in TCC exhibited significant differences in their level of comfort with the three patients' early refill requests, we saw a more consistent intended course of action for all three patients: requiring more information before a refill could be provided. It is our hope that as our students begin their clinical clerkship rotations, they will take a similar approach to a patient's early refill request regardless of age, gender, and/or reason for the request.

The lower-than-expected percentage of students completing the history-taking and information sharing/patient education items on the SP encounter checklist was surprising. It prompted us to rethink how we train our students to ask patients questions and educate patients on important topics, such as drug prescription usage. Our goal is for at least 90% of students to complete every item on the SP encounter checklist.

Challenges and Lessons Learned

As noted, the COVID-19 pandemic required us to deliver this curriculum differently than we had originally planned. Nevertheless, we successfully implemented the curriculum virtually and believe that it can be used in either the virtual or in-person format. Additionally, we were prepared to create video recordings of the physician-patient interactions of the three vignettes for the MS 1 students in TMDS, but we were not able to produce the videos due to social distancing regulations. However, we found that the embedded audio recordings of the physician-patient interactions worked well and achieved our goals and objectives for the session. When we are able, we may proceed with creating the videos for future use.

It is important to note that because of our limited SP pool, not all the SPs who portrayed Darryl Whitcomb (Case 2) during MS 3 TCC were African American, although, during the MS 1 TMDS presentation, Darryl Whitcomb was presented as African American. Therefore, we were not able to determine if the SP's race was an influencing factor in students' OSCE performance and follow-up responses to the survey questions. Having a diverse pool of SPs, especially with regard to race and ethnicity, has been a continual challenge for us. To circumvent this challenge, instead of developing OSCE cases with patients representing different racial and ethnic backgrounds, we will focus more broadly on other social determinants of health, such as socioeconomic status, age, gender identity, sexual orientation, and health literacy.

Lastly, we did not assess or collect data from students regarding perceptions or awareness of their own biases and whether participation in iPAC helped them become more aware of and confident in identifying personal biases, especially when providing patient care. Development of such evaluation and data-collection tools will be considered for the next iteration of iPAC. These evaluation and data-collection tools should be administered not only after the delivery of iPAC but also after students are immersed in their clinical rotations to examine whether or not there is sustained effect and impact.

Future Directions and Adaptations

We plan to implement longitudinal tracking and monitoring of students' performance as they progress through iPAC. The MS 1s who participated in the TMDS session will soon participate in the TCC session. At that time, not only will we be able to examine whether there are changes in students' level of comfort and intended course of action with early refill requests by patients, we will also be able to compare OSCE performance between the two groups of TCC students—one group that had the TMDS session and one that did not. Although it was intentional to use the same three patient vignettes for both the MS 1 TMDS and MS 3 TCC courses, we may modify the cases based on the OSCE performances of the MS 3 students who were introduced to the three patient vignettes as MS 1s. While we believe it is advantageous for students to first experience the patient cases in more of an observatory format as MS 1s and then revisit the same clinical cases as MS 3s through hands-on, application activities (such as OSCEs), we could instead consider having the patient cases unfold over time. Rather than using the same case of an early refill request of an opioid medication, we could consider creating new OSCE cases that simulate follow-up visits by the same three patients presenting with other issues related to pain management and potential SUDs.

Finally, OSCE cases that relate to prescription refills for advanced clinical learners in iPAC should include an item on the SP encounter checklist regarding whether the student indicated they would provide the prescription to the patient. It is understandable for students in their early clinical training to tell a patient that they need to talk to their attending or to ask the patient for more information before they agree to provide a prescription refill, since they are less experienced and less confident. However, as students progress in their training, they should be expected to demonstrate more advanced knowledge, skills, and insight in simulated environments by making more refined clinical decisions such that educators are able to assess the

Copyright © 2022 Lu et al. This is an open-access publication distributed under the terms of the Creative Commons Attribution license.

students' clinical decision-making skills. Nevertheless, structured self-reflection should be emphasized more during the faculty-facilitated debrief sessions so that students are compelled to think about personal biases and the impact these biases may have on the decisions they make in caring for patients in the clinical environment.

Appendices

A. MS 1 Clinical Vignettes & Follow-Up.pptx
B. MS 1 Debrief.pptx
C. SP James Spiegel - Case 1.docx
D. SP Darryl Whitcomb - Case 2.docx
E. SP Helen Morgan - Case 3.docx
F. Door Notes.docx
G. Logistical Flow.docx
H. Faculty Post-OSCE Debrief Discussion Guide.docx
I. SP Encounter Checklist.docx
J. SP Responses for Checklist Items.docx
K. MS 3 Post-OSCE Survey.docx

All appendices are peer reviewed as integral parts of the Original Publication.

Wei-Hsin Lu, PhD: Director of Assessment and Evaluation and Research Assistant Professor of Preventive Medicine, Renaissance School of Medicine at Stony Brook University; ORCID: https://orcid.org/0000-0002-5559-8850

Perrilynn Baldelli, DNP, RN: Director, Clinical Simulation Center, Renaissance School of Medicine at Stony Brook University

Phyllis Migdal, MD, MA: Clinical Assistant Professor, Department of Family, Population, and Preventive Medicine, Renaissance School of Medicine at Stony Brook University

Richard Iuli, PhD: Director, Pathways to Success, Office of Academic Affairs, Renaissance School of Medicine at Stony Brook University

Lisa Strano-Paul, MD: Clinical Professor of Medicine and Assistant Dean for Clinical Education, Renaissance School of Medicine at Stony Brook University

Kevin L. Zacharoff, MD: Clinical Instructor and Distinguished Visiting Scholar in Medical Humanities, Compassionate Care, and Preventive Medicine, Department of Family, Population, and Preventive Medicine, Renaissance School of Medicine at Stony Brook University

Disclosures

None to report.

Funding/Support

This project is funded by the Association of American Medical Colleges grant for "Responding to the Training and Development Needs of Academic Medical Centers."

Prior Presentations

Zacharoff K, Migdal P, Baldelli P, Iuli R, Lu WH. Conscious efforts to address unconscious biases in health care delivery training: the iPAC (Integrated Pain and Addiction Curriculum) program at Stony Brook Renaissance School of Medicine. Poster presented virtually at: Association of American Medical Colleges Group on Educational Affairs Regional Spring Meeting; April 20-22, 2021.

Ethical Approval

The Stony Brook University Institutional Review Board deemed further review of this project not necessary.

References

1. CDC WONDER. Centers for Disease Control and Prevention. Accessed May 29, 2019. http://wonder.cdc.gov
2. Hirsh AT, Hollingshead NA, Ashburn-Nardo L, Kroenke K. The interaction of patient race, provider bias, and clinical ambiguity on pain management decisions. *J Pain*. 2015;16(6):558-568. https://doi.org/10.1016/j.jpain.2015.03.003
3. Institute of Medicine. *Relieving Pain in America: A Blueprint for Transforming Prevention, Care, Education, and Research.* National Academies Press; 2011.
4. National Academies of Sciences, Engineering, and Medicine. *Pain Management and the Opioid Epidemic: Balancing Societal and Individual Benefits and Risks of Prescription Opioid Use.* National Academies Press; 2017.
5. Payne BK, Gawronski B. A history of implicit social cognition: where is it coming from? Where is it now? Where is it going? In: Gawronski B, Payne BK, eds. *Handbook of Implicit Social Cognition: Measurement, Theory, and Applications.* Guilford Press; 2010:1-15.
6. van Boekel LC, Brouwers EPM, van Weeghel J, Garretsen HFL. Stigma among health professionals towards patients with substance use disorders and its consequences for healthcare delivery: systematic review. *Drug Alcohol Depend*. 2013; 131(1-2):23-35. https://doi.org/10.1016/j.drugalcdep.2013.02.018
7. FitzGerald C, Hurst S. Implicit bias in healthcare professionals: a systematic review. *BMC Med Ethics*. 2017;18:19. https://doi.org/10.1186/s12910-017-0179-8
8. van Boekel LC, Brouwers EPM, van Weeghel J, Garretsen HFL. Healthcare professionals' regard towards working with patients with substance use disorders: comparison of primary care, general psychiatry and specialist addiction services. *Drug Alcohol Depend*. 2014;134:92-98. https://doi.org/10.1016/j.drugalcdep.2013.09.012

Copyright © 2022 Lu et al. This is an open-access publication distributed under the terms of the Creative Commons Attribution license.

9. Vettese TE, Thati N, Roxas R. Effective chronic pain management and responsible opioid prescribing: aligning a resident workshop to a protocol for improved outcomes. *MedEdPORTAL*. 2018;14:10756. https://doi.org/10.15766/mep_2374-8265.10756
10. Gonzalez CM, Nava S, List J, Liguori A, Marantz PR. How assumptions and preferences can affect patient care: an introduction to implicit bias for first-year medical students. *MedEdPORTAL*. 2021;17:11162. https://doi.org/10.15766/mep_2374-8265.11162

Received: September 20, 2021
Accepted: January 6, 2022
Published: April 7, 2022

Copyright © 2022 Lu et al. This is an open-access publication distributed under the terms of the Creative Commons Attribution license.
